# Ruthenium‐Catalyzed C−H Arylation of Benzoic Acids and Indole Carboxylic Acids with Aryl Halides

**DOI:** 10.1002/chem.201605068

**Published:** 2016-12-05

**Authors:** Marco Simonetti, Diego M. Cannas, Adyasha Panigrahi, Szymon Kujawa, Michal Kryjewski, Pan Xie, Igor Larrosa

**Affiliations:** ^1^ School of ChemistryUniversity of ManchesterOxford RoadManchesterM13 9PLUK; ^2^ Department of Inorganic and Analytical ChemistryPoznan University of Medical SciencesGrunwaldzka 660-780PoznanPoland

**Keywords:** arylation, benzoic acids, C−H activation, indole carboxylic acids, ruthenium

## Abstract

Herein we report the first Ru‐catalyzed C−H arylation of benzoic acids with readily available aryl (pseudo)halides. The reaction, which does not require the use of silver salt additives, allows the arylation of previously challenging hindered benzoic acids and the use of generally unreactive *ortho*‐substituted halorarenes. Furthermore, our new protocol can efficiently be applied to indole carboxylic acids, thus allowing access to C7‐, C6‐, C5‐ and C4‐arylated indole compounds, a departure from the classical enhanced reactivity of the C2 and C3 positions of indole.

The development of new methods for the production of biologically and industrially relevant compounds, while retaining a high level of atom‐economy, still remains a big challenge in the field of organic synthesis. The biaryl motif is a privileged scaffold found in natural products, pharmaceuticals, and organic functional materials.^1^ C−H arylation, the coupling of an arene (C_Ar−H_) with an aryl halide (C_Ar−X_) is developing as a greener, more efficient and atom‐economical route for the construction of biaryls compared to traditional methods.^2^ Particularly, the presence of Lewis basic directing groups (DG) within the substrate has been identified as the most versatile approach to bind the metal catalyst and selectively deliver it to a proximal C−H bond in an intramolecular fashion.^2^ However effective, directing groups are rarely a necessity after the C−H functionalization event, in which case, their removal decreases the overall atom‐economy of the process.^3^ On the other hand, the use of carboxylates as DGs brings several advantages as benzoic acids are non‐toxic, shelf‐stable, cheap and readily available. Moreover, the carboxylic unit can be exploited for selective transformations through decarboxylative pathways,^4^ including tandem *ortho*‐functionalization/decarboxylation processes.^5^ Nearly a decade ago, the groups of Daugulis^6^ and Yu^7^ pioneered the Pd‐catalyzed carboxylate‐directed C−H arylation of benzoic acids with aryl halides and aryl boron reagents (Scheme 1), respectively. Subsequent contributions from our group and those of Zhou, Zhao and Su have further developed the scope and efficiency of these Pd‐catalyzed processes.^8^, ^9^, ^10^, ^11^ Lan, You and co‐workers also described a Rh‐catalyzed oxidative C−H arylation of benzoic acids with heteroaromatic compounds.^12^ More recently, Gooßen reported that aryl diazonium salts are suitable electrophiles when using an Ir‐catalyst.^13^ Most of these methods require the use of stoichiometric Ag‐salt additives and present important substrate scope limitations, in particular, with respect to *ortho*‐substituted aryl electrophiles, hindered benzoic acids, and heteroaromatic carboxylic acids. Despite the tremendous progress over the last few years on the development of C−H arylation methods catalyzed by the much cheaper transition metal ruthenium,2h, 14 there are no reports of the successful direct arylation of a benzoic acid. On the other hand, couplings with alkenes and alkynes have been reported.5h–5j, 15 For these reasons, we set out to investigate the possibility of developing a Ru‐catalyzed arylation of benzoic acids employing readily available aryl halides as the coupling partners. Herein we report a new method that allows the arylation of benzoic acids with Ru‐catalyst loadings as low as 0.5 mol %, and allows the use of *ortho*‐substituted aryl donors and hindered benzoic acids. Furthermore, we report the first examples of a carboxylic acid directed C−H arylation of indoles at the fused benzene core, which allow regioselective arylations at C4, C5, C6 and C7 positions.

**Scheme 1:**
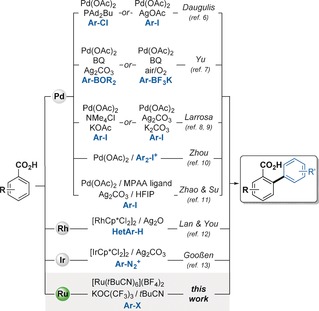
Carboxylate‐directed C−H arylation of benzoic acids under Pd, Ir, Rh and Ru catalysis.

Initially we investigated the reaction of *o*‐toluic acid, **1 a**, with 4‐iodoanisole, **2 a**, with [RuCl_2_(*p*‐cymene)]_2_, K_2_CO_3_ and 1,4‐dioxane at 100 °C (Table 1), classical conditions used in Ru‐catalyzed C−H arylation. Under these conditions, the cross‐coupled product **3 aa** was formed in 2 % yield. In view of our mechanistic studies on the nature of the Ru^II^ species involved in the C−H arylation of fluoroarenes,^16^ we hypothesized that a *p*‐cymene‐free catalyst would provide better reactivity. Indeed, the η^6^‐arene‐free Ru^II^ catalyst [Ru(*t*BuCN)_6_](BF_4_)_2_ recently developed in our group^16^ provided **3 aa** in 7 % yield (entry 2). A solvent screening revealed that more coordinating solvents increased the formation of the cross‐coupled adduct.^17^ The most effective one was *t*BuCN, which afforded **3 aa** in 41 % yield (entry 3). Examination of other alkali carbonate bases, as well as phosphines, pyridines and NHC‐type ligands did not result in any improvement in the yield of **3 aa**.^17^ The addition of KOPiv (entry 4) further increased the formation of **3 aa** to 51 %, possibly by assisting the C−H activation via a concerted metalation‐deprotonation (CMD) type pathway.14e, 16,14f, 15a, 18 Interestingly, when potassium perfluoro *tert*‐butoxide was used instead of KOPiv, **3 aa** was formed in 61 % yield (entry 5). We recently disclosed the higher activity of polyfluorinated alkoxide salts over more classical bases commonly employed to facilitate the metalation step.^16^ The ability of polyfluoroalcohols in promoting hydrogen‐bonding in the presence of a suitable proton acceptor,^19^, ^20^ while maintaining a low concentration of acidic protons in solution in view of their relative low boiling points, might provide a reasonable explanation behind their exceptional reactivity. When the temperature was raised to 140 °C, **3 aa** was yielded quantitatively (entry 6). Lastly, reducing the Ru‐catalyst loading to 3 mol % and *t*BuCN to 8.0 equivalents (entry 7) was possible, while maintaining the excellent yield.

**Table 1:** Optimization of the Ru‐catalyzed C−H arylation of **1 a** with **2 a**.

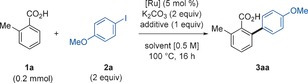				
Entry	Ru	Solvent	Additive	**3 aa^[a]^**
1	[RuCl_2_(*p‐*cymene)]_2_	1,4‐dioxane		2
2	[Ru(*t*BuCN)_6_](BF_4_)_2_	1,4‐dioxane		7
3	[Ru(*t*BuCN)_6_](BF_4_)_2_	*t*BuCN		41
4	[Ru(*t*BuCN)_6_](BF_4_)_2_	*t*BuCN	KOPiv	51
5	[Ru(*t*BuCN)_6_](BF_4_)_2_	*t*BuCN	KOC(CF_3_)_3_	61
6^[b]^	[Ru(*t*BuCN)_6_](BF_4_)_2_	*t*BuCN	KOC(CF_3_)_3_	>99
7^[b,c]^	[Ru(*t*BuCN)_6_](BF_4_)_2_	*t*BuCN	KOC(CF_3_)_3_	>99

[a] Yield determined by ^1^H‐NMR using 1,3‐dinitrobenzene as internal standard. [b] Reaction carried out at 140 °C. [c] [Ru(*t*BuCN)_6_](BF_4_)_2_ (3 mol %), *t*BuCN (8 equiv).

With the optimal reaction conditions in hand (Table 1, entry 7) we explored the compatibility of our protocol with a variety of functionalities on the aryl iodide coupling partner for the arylation of *o*‐toluic acid **1 a** (Table 2). The reaction tolerates a wide range of substituents at the *ortho*, *meta*, and *para* positions of the aryl iodide, affording the corresponding biaryl products **3 aa**–**3 ax** in moderate to excellent yields. Generally, electron‐poor aryl iodides (**3 ab**, **3 ao**–**s**, **3 av**) are less reactive than electron‐rich ones (**3 aa**, **3 af**–**g**, **3 at**). Notably, *ortho*‐substituted aryl halides, which are unreactive under Pd catalysis,^6^, ^7^, ^8^, ^9^, ^10^, ^11^ were compatible with our Ru system (**3 ac**–**d, 3 ah**, **3 am** and **3 au**). Remarkably, halogen substituents were tolerated while maintaining excellent yields (**3 ai**‐**n**). Particularly, the tolerance of bromo and iodo substituents open up new avenues towards further coupling reactions. Electrophiles bearing sensitive functional groups such as CO_2_Me (**3 bo**), COMe (**3 bn**), SMe (**3 bl**) reacted smoothly in the protocol. In addition to aryl iodides, the procedure was applicable to aryl bromides, chlorides, as well as pseudohalide PhOTf, although with a reduced efficiency. Conversely, PhOTs was unreactive in the system (**3 ae**). Additionally, heteroaromatic iodides containing the indole and thiophene core were successfully employed under identical conditions (**3 av**–**3 aw**). Finally, iodoarenes containing nitro‐ (**3 ay**) and ‐CHO substituents (**3 az**) failed to react in this system.

**Table 2:** Scope of the Ru‐catalyzed arylation of **1 a** with haloarenes **2 a**–**2 z**.^[a]^

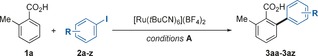
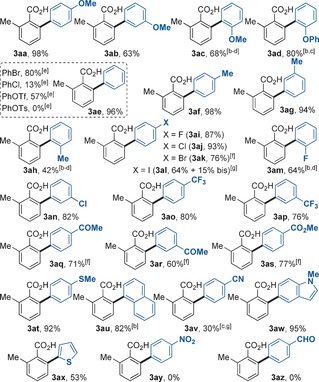

[a] *Reaction conditions* **A**: **1 a** (0.3 mmol), **2 a**–**z** (2.0 equiv), [Ru(*t*BuCN)_6_](BF_4_)_2_ (3 mol %), K_2_CO_3_ (2.0 equiv), KOC(CF_3_)_3_ (1.0 equiv) and *t*BuCN (8.0 equiv) stirred under Ar in a closed vessel at 140 °C for 16 h. Yields are of pure, isolated products. [b] [Ru(*t*BuCN)_6_](BF_4_)_2_ (6 mol %). [c] Isolated as the corresponding methyl ester after derivatization with MeI. [d] 3.0 equiv of H_2_O were added. [e] Yield evaluated by ^1^H NMR with 1,3‐dinitrobenzene as internal standard. [f] Reaction time 3 h. [g] Reaction time 1 h.

We then turned our attention to the generality of this method with respect to the benzoic acid partner (Table 3). Benzoic acids bearing a variety of electronically different substituents displayed excellent reactivity. Although *ortho*‐substituted benzoic acids reacted under standard conditions **A** (Table 1, entry 7 and Table 2), *meta*‐ and *para*‐substituted ones required the addition of 3.0 equivalents of H_2_O and, in the cases in which bis‐arylation was occurring, an adjustment of the relative stoichiometry of the reagents (conditions **B**). The beneficial effect of H_2_O might be due to an improved solubilization of the poorly soluble 3‐ and 4‐substituted potassium benzoates, which are generated in situ during the reaction. Weakly coordinating groups such as esters and ketones, which act as directing groups for a variety of TM‐catalyzed C−H functionalizations^21^ including Ru catalysis,15b, 22 were overridden by the carboxylic acid leading to the *ortho*‐aryl adducts **3 ea** and **3 ma** with complete regioselectivity. Remarkably, sterically hindered benzoic acids were compatible with this system, yielding **3 ga**–**3 ja** in modest to good yields. All previously reported methods have failed to incorporate an aryl group in such an encumbered environment,^6^, ^7^, ^8^, ^9^, ^10^, ^11^, ^12^, ^13^ further highlighting another unique feature of this system. When *meta*‐substituted benzoic acids **3 k**–**m** were tested, the least hindered position selectively reacted. On the other hand, 3‐fluoro benzoic acid only provided the bis‐arylated adduct **3 la**. This difference in regioselectivity suggests that the preference for the least hindered site can be in some cases less pronounced. Indeed, when two *ortho*‐C−H bonds possessing not exceedingly diverse steric impediment are competing, a mixture of mono‐ and bis‐ arylation was obtained (benzoic acids **3 n, 3 p,q**). In the latter cases, in order to selectively obtain bis‐arylation, a higher loading of catalyst, potassium perfluoro *tert*‐butoxide and *t*BuCN, along with an extended reaction time, were required (conditions **B**). Finally, *para*‐substituted benzoic acids, as well as the parent benzoic acid, selectively furnished the corresponding bis‐arylated products, without even traces of mono‐arylation (**3 ra**–**3 za**). This represents another element of distinction from the previously reported methods. For example, the Ir‐catalyzed system led to a mixture of mono‐ and bis‐arylation^13^ and the Pd protocols^6^, ^7^, ^8^, ^9^, ^10^, ^11^ can be seen as orthogonal methods since mono‐arylation was achieved in all cases.^23^

**Table 3:** Scope of the Ru‐catalyzed arylation of benzoic acids **1 b**–**1 x** with 4‐iodoanisole **2 a**.^[a]^

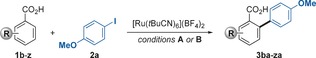
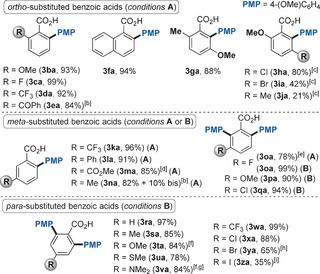

[a] *Reaction conditions* **A**: **1 b**–**n** (0.3 mmol), **2 a** (2.0 equiv), [Ru(*t*BuCN)_6_](BF_4_)_2_ (3 mol %), K_2_CO_3_ (2.0 equiv), KOC(CF_3_)_3_ (1.0 equiv) and *t*BuCN (8.0 equiv) stirred under Ar in a closed vessel at 140 °C for 16 h. Yields are of pure, isolated products. *Reaction conditions* **B**: **1 o**–**z** (0.3 mmol), **2 a** (4.0 equiv), [Ru(*t*BuCN)_6_](BF_4_)_2_ (6 mol %), K_2_CO_3_ (3.0 equiv), KOC(CF_3_)_3_ (1.5 equiv), *t*BuCN (12.0 equiv) and H_2_O (3.0 equiv) stirred under Ar in a closed vessel at 140 °C for 24 h. Yields are of pure, isolated products. [b] Isolated as the corresponding methyl ester after derivatization with MeI. [c] [Ru(*t*BuCN)_6_](BF_4_)_2_ (6 mol %). [d] Reaction time 3 h. [e] Yield evaluated by ^1^H NMR with 1,3‐dinitrobenzene as internal standard. [f] [Ru(*t*BuCN)_6_](BF_4_)_2_ (10 mol %). [g] Reaction time 1 h. [h] Reaction time 12 h, no H_2_O was added. [i] Reaction time 4 h.

To demonstrate the utility of our Ru‐catalyzed arylation, we scaled the reaction more than 150 times while lowering the catalyst loading to 0.5 mol % and *t*BuCN to 3.0 equivalents. After a simple acid‐base work up, followed by a recrystallization in MeOH/H_2_O, product **3 aa** was afforded in 96 % yield (11.61 g, Scheme 2).

**Scheme 2:**
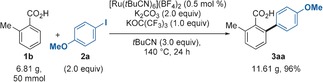
Multi‐gram scale synthesis of **3 aa**.

The indole ring is present in a myriad of natural products found in terrestrial and marine environments. Indole derivatives are among the most studied class of heterocyclic compounds in drug‐discovery because of their exceptional activity towards microbes, cancer cells, receptors involved in the chemistry of the brain, and several other disorders in the human body.^24^ For these reasons, the development of new strategies for selectively installing molecular complexity on the indole core is of significant interest. Particularly, the C4, C5, C6 and C7 positions are highly challenging sites to C−H functionalize due to the intrinsic higher reactivity of the fused pyrrole ring positions C2 and C3.^25^ Indeed, single‐step functionalization protocols in which both C2 and C3 positions are unblocked are extremely rare.^26^, ^27^ Very recently the group of Shi discovered that *N*‐P(O)*t*Bu_2_ protected indoles can be arylated at the C7 position under Pd catalysis with boronic acids and stoichiometric amounts of Cu^II^ and Ag^I^ salts,26a or at C6 under Cu catalysis employing bisaryliodonium salts.26b

By applying similar reaction conditions to those developed for the arylation of benzoic acids, 4‐, 5‐, 6‐, and 7‐indole carboxylic acids reacted exclusively at the carboxylic acid *ortho* position(s), without any side‐arylation products at either C2 or C3 positions (Table 4). A wide array of functional groups is tolerated at the *ortho, para* or *meta* positions of the aryl iodide coupling partner, including electron‐rich, electron‐poor and halogen‐containing ones. Interestingly, the *N*‐methyl protection of the 7‐indole carboxylic was required to prevent the carboxylate directed N‐H arylation (**5 ba**). On the contrary, 1*H*‐indole‐6‐carboxylic acids regioselectively provided bis‐arylation at C7 and C5 positions, without detecting coupling with the N atom (**5 ea**, **5 fa**). Particularly, these two examples constitute the first C−H functionalizations at the fused benzene ring of the indole without protecting at least one site of its pyrrole core. Instead of C5+C7 bisarylation, 1‐methyl‐1*H*‐indole‐6‐carboxylic acid **4 c** provided exclusively mono‐arylation at C5, likely due to steric reasons. Also the drug‐like 6‐indole carboxylic acid derivative **4 d**, en route to its *N*,*N*‐dimethyltryptamine analogue,^28^ was efficiently arylated (**5 da**, 75 %). Moreover, the C5 position, which has never exclusively been accessed before, can also be tackled from 1‐methyl‐1*H*‐indole‐4‐carboxylic acid **4 g**.^29^ Unfortunately, indole 2 and 3 carboxylic acids **4 k**–**n**, as well as unprotected 4 and 5 indole carboxylic acids (**4 h**, **4 j**), could not yet be converted. Finally, 1‐methyl‐1*H*‐indole‐5‐carboxylic acid **5 i** was efficiently bis‐arylated at C4 and C6 positions.

**Table 4:** Scope of the Ru‐catalyzed arylation of indole carboxylic acids **4 a**–**n** with iodoarenes **2**.^[a]^

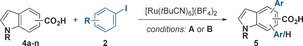
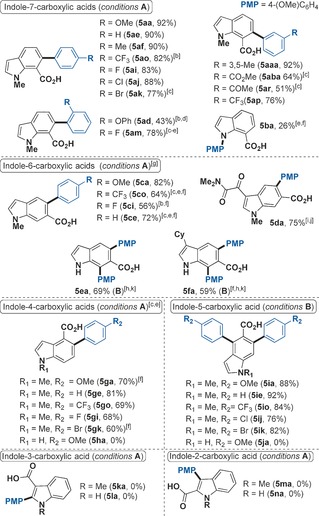

[a] *Reaction conditions* **A**: **4** (0.3 mmol), **2** (2.0 equiv), [Ru(*t*BuCN)_6_](BF_4_)_2_ (3 mol %), K_2_CO_3_ (2.0 equiv), KOC(CF_3_)_3_ (1.0 equiv) and *t*BuCN (8.0 equiv) stirred under Ar in a closed vessel at 140 °C for 16 h. Yields are of pure, isolated products. *Reaction conditions* **B**: **4** (0.3 mmol), **2** (4.0 equiv), [Ru(*t*BuCN)_6_](BF_4_)_2_ (6 mol %), K_2_CO_3_ (3.0 equiv), KOC(CF_3_)_3_ (1.5 equiv), *t*BuCN (12.0 equiv) and H_2_O (3.0 equiv) stirred under Ar in a closed vessel at 140 °C for 16 h. Yields are of pure, isolated products. [b] Reaction time 8 h. [c] Reaction time 3 h. [d] [Ru(*t*BuCN)_6_](BF_4_)_2_ (6 mol %). [e] 3.0 equiv of H_2_O were added. [f] Isolated as the corresponding methyl ester after derivatization with MeI. [g] [Ru(*t*BuCN)_6_](BF_4_)_2_ (5 mol %). [h] No H_2_O was added. [i] Reaction time 5 h. [j] Isolated as the corresponding benzyl ester after derivatization with BnCl. [k] Reaction time 12 h.

In conclusion, we developed the first Ru‐catalyzed C−H arylation of benzoic acids with aryl (pseudo)halides that does not require any Ag^I^ or Cu^II^ salts as halide scavenger and/or oxidant. Electron‐rich, electron‐poor, as well as halogen‐containing aryl iodides displayed excellent reactivity in the presented method. Contrarily to Pd catalysis, *ortho*‐substituted aryl halides were suitable coupling partners. Sterically encumbered benzoic acids, which have always failed to react with previous methods, were successfully arylated. *para*‐Substituted benzoic acids exclusively provided bis‐arylation, offering an alternative to Pd‐catalyzed procedures that selectively deliver mono‐arylation. The process can be easily scaled up with a remarkably low catalyst loading. Furthermore, 4‐, 5‐, 6‐ and 7‐indole carboxylic acids were regioselectively *ortho*‐arylated, overriding the classically more reactive C2 and C3 positions. Particularly, the C5 position was for the first time selectively accessed, further highlighting the novelty of this method.

## Supporting information

As a service to our authors and readers, this journal provides supporting information supplied by the authors. Such materials are peer reviewed and may be re‐organized for online delivery, but are not copy‐edited or typeset. Technical support issues arising from supporting information (other than missing files) should be addressed to the authors.

Supplementary
