# Supplementary material for: Ruthenium‐Catalyzed C−H Arylation of Benzoic Acids and Indole Carboxylic Acids with Aryl Halides
Source: Chemistry. 2016 Dec 5;23(3):549–53. doi: 10.1002/chem.201605068 (PMC5412846; doi:10.1002/chem.201605068)
Supplement: Supplementary file 1 — Supplementary [file CHEM-23-549-s001.pdf]

CHEMISTRY
A European Journal

Supporting Information

Ruthenium-Catalyzed C−H Arylation of Benzoic Acids and Indole Carboxylic Acids with Aryl Halides

Marco Simonetti+,[a] Diego M. Cannas+,[a] Adyasha Panigrahi+,[a] Szymon Kujawa,[a]
Michal Kryjewski,[b] Pan Xie,[a] and Igor Larrosa*[a]

chem_201605068_sm_miscellaneous_information.pdf

General information .... **2.**

General Procedure A .... **2.**

General Procedure B .... **3.**

Procedure for the arylation of 1a with 2a (50 mmol scale) .... **4.**

Procedure for the preparation of 5-indole carboxylic acid derivative 4d .... **4.**

Procedure for the preparation of $KOC(CF_3)_3$ .... **6.**

Experimental characterization data for products .... **9.**

^{1}H NMR, ^{13}C NMR and ^{19}F NMR spectra .... **46.**

References .... **149.**

General information

Unless otherwise indicated, all reactions were carried out in a glove box using reagents obtained from commercial sources and used without further purification. All solid reagents were dried in vacuum oven at 60 °C for 24 h prior to use and stored in a glove box, unless otherwise stated. All other starting materials and solvents were purchased from Acros, Aldrich, Alfa Aesar, Fluorochem, Apollo Scientific and Manchester Organics, and used without further purification, unless otherwise stated. $[Ru(^{t}BuCN)_6](BF_4)_2$,[1] 1-iodo-2-phenoxybenzene (**2d**)[2] and 5-iodo-1-methyl-1H-indole (**2w**)[3] were prepared according to reported methods. Column chromatography was carried out on silica gel, particle size 40-63 μm, using flash techniques. Melting points were obtained using a Stuart SMP11 apparatus and are uncorrected. IR spectra were recorded using a Thermo Scientific Nicolet iS5 FTIR machine, relevant bands are quoted in cm^{-1}. High resolution mass spectra were performed by the School of Chemistry Mass Spectrometry Service (University of Manchester) employing a Thermo Finnigan MAT95XP spectrometer. ^{1}H NMR, ^{19}F NMR and ^{13}C NMR spectra were recorded at 400 or 500 MHz on Bruker machines. ^{1}H NMR are referenced to the residual solvent peak at 7.26 ppm ($CDCl_3$), 2.05 ppm ($(CD_3)_2CO$), 1.94 (CD_3CN) and quoted in ppm to 2 decimal places with coupling constants (J) to the nearest 0.1 Hz. ^{13}C NMR spectra, recorded at 101 MHz or 126 MHz, are referenced to the solvent peak at 77.16 ppm ($CDCl_3$), 29.84 ppm ($(CD_3)_2CO$) and quoted in ppm to 1 decimal place with coupling constants (J) to the nearest 0.1 Hz. ^{19}F NMR spectra were recorded at 376 or 471 MHz in $CDCl_3$, $(CD_3)_2CO$ and quoted in ppm to 2 decimal places and with coupling constants (J) to the nearest 0.1 Hz.

General Procedure A

All solid reagents, except for $[Ru(^{t}BuCN)_6](BF_4)_2$, were dried at 60 °C in a vacuum oven over night. All liquid reagents were dried over 4 Å molecular sieves and degassed with 3 freeze-pump-thaw cycles. In a glove box, an appropriate carboxylic acid (1.0 equiv, 0.30 mmol), (pseudo)haloarene (2.0 equiv, 0.60 mmol), $[Ru(^{t}BuCN)_6](BF_4)_2$ (7.0 mg, 3 mol %), K_2CO_3 (82.9 mg, 2.0 equiv, 0.60 mmol), $KOC(CF_3)_3$ (82.2 mg, 1.0 equiv, 0.30 mmol) and $^{t}BuCN$ (265.3 μL, 8.0 equiv, 2.40 mmol) were loaded in a crimp-cap microwave vial. If needed, the vial was sealed,

taken outside the box and H_2O (16.2 µL, 3.0 equiv, 0.90 mmol) was added *via* syringe. Then, the vial was taken back inside the box, resealed with a new cap and heated at 140 °C for 16 h. Upon completion, the reaction mixture was cooled to room temperature and 1.0 mL of tartaric acid (10% in H_2O) was added. The crude was filtered through a short plug of silica, eluted with EtOAc (3 × 10 mL), $(CH_3)_2CO$ (2 × 10 mL) and the mixture was evaporated to dryness. If needed, MeI (93.4 µL, 5.0 equiv, 1.50 mmol) or BnCl (172.6 µL, 5.0 equiv, 1.50 mmol), K_2CO_3 (82.9 mg, 2.0 equiv, 0.60 mmol) and MeCN (2.0 mL) were loaded inside the flask and stirred at 60 °C for 2 h. After removal of all the solvents, the crude was loaded on a silica column for flash chromatography to afford the corresponding biaryl.

General Procedure B

All solid reagents, except for $[Ru(^tBuCN)_6](BF_4)_2$, were dried at 60 °C in a vacuum oven over night. All liquid reagents were dried over 4 Å molecular sieves and degassed with 3 freeze-pump-thaw cycles. In a glove box, an appropriate carboxylic acid (1.0 equiv, 0.30 mmol), iodoarene (4.0 equiv, 1.20 mmol), $[Ru(^tBuCN)_6](BF_4)_2$ (6 mol %, 13.9 mg), K_2CO_3 (124.4 mg, 3.0 equiv, 0.90 mmol), $KOC(CF_3)_3$ (123.4 mg, 1.5 equiv, 0.45 mmol) and ^tBuCN (398.0 µL, 12.0 equiv, 3.60 mmol) were loaded in a crimp-cap microwave vial. The vial was sealed, taken outside the box and H_2O (16.2 µL, 3.0 equiv, 0.90 mmol) was added *via* syringe. Then, the vial was taken back inside the box, resealed with a new cap and heated at 140 °C for 24 h. Upon completion, the reaction mixture was cooled to room temperature and 1.0 mL of tartaric acid (10% in H_2O) was added. The crude was filtered through a short plug of silica, eluted with EtOAc (3 × 10 mL), $(CH_3)_2CO$ (2 × 10 mL) and the mixture was evaporated to dryness. If needed, MeI (93.4 µL, 5.0 equiv, 1.50 mmol), K_2CO_3 (82.9 mg, 2.0 equiv, 0.60 mmol) and MeCN (2.0 mL) were loaded inside the flask and stirred at 60 °C for 2 h. After removal of all the solvents, the crude was loaded on a silica column for flash chromatography to afford the corresponding biaryl.

Procedure for the arylation of 1a with 2a (50 mmol scale)

All solid reagents, except for $[Ru(^tBuCN)_6](BF_4)_2$, 2-methylbenzoic acid and 4-iodoanisole were dried at 60 °C in a vacuum oven over night. All liquid reagents were dried over 4 Å molecular sieves and degassed with 3 freeze-pump-thaw cycles. 2-

methylbenzoic acid (6.81 g, 1.0 equiv, 50.0 mmol), 4-iodoanisole (23.40 g, 2.0 equiv, 100.0 mmol) and [Ru(^{t}BuCN)$_6$](BF$_4$)$_2$ (193.4 mg, 0.5 mol %) were loaded in a 200 mL Ace® pressure tube. Then, the vial was brought in a glove box and K_2CO_3 (13.82 g, 2.0 equiv, 100.0 mmol), KOC(CF$_3$)$_3$ (13.71 g, 1.0 equiv, 50.0 mmol) and ^{t}BuCN (16.6 mL, 3.0 equiv, 150.0 mmol) were weighed into the pressure tube and heated at 140 °C for 24 h. Upon completion, the reaction mixture was diluted with H_2O (500 mL), EtOAc (300 mL) and the aqueous layer phase washed with EtOAc (2 × 300 mL). The combined organic layers were extracted again with H_2O (1 × 200 mL). Then, the joint aqueous phases were carefully acidified to pH 1 with HCl 37%, extracted with EtOAc (3 × 350 mL), washed with brine (2 × 300 mL), dried over $MgSO_4$ and evaporated to dryness to afford a pale yellow solid. Recrystallization from MeOH/H_2O gave **3aa** as a white solid (11.61 g, 96%).

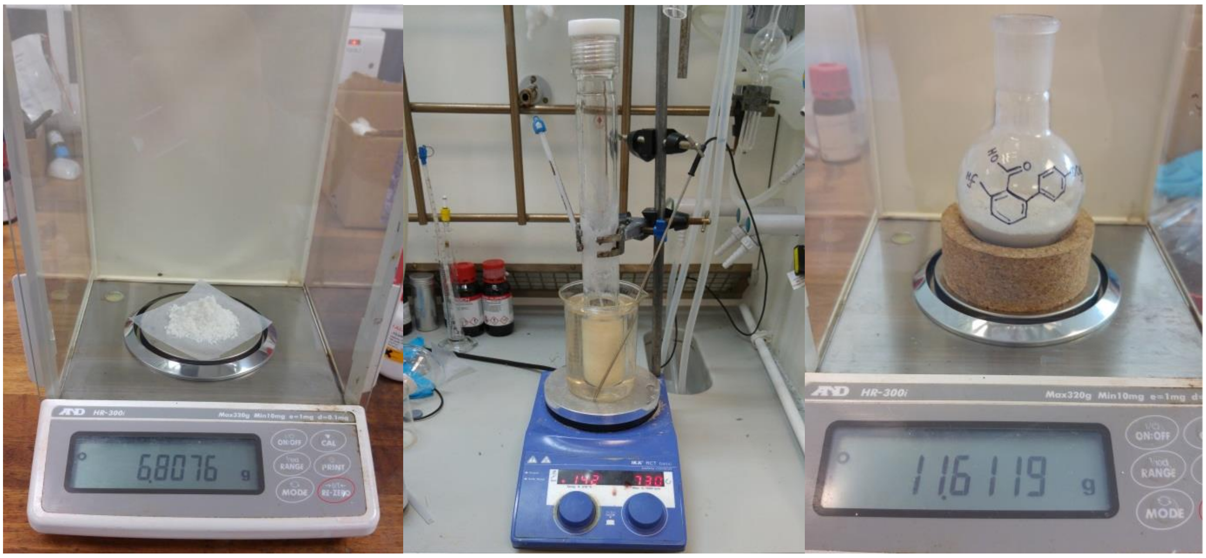

Figure 1. *Left hand-side*, *o*-toluic acid; *centre*, reaction mixture; *right hand-side*, pure **3aa**.

Procedure for the preparation of 5-indole carboxylic acid derivative 4d

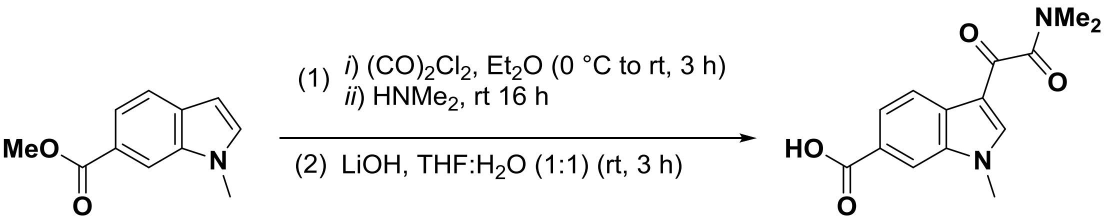

A modified procedure of Nichol's method for the synthesis of Psilocybin was employed.[4] A solution of methyl 1-methyl-1H-indole-6-carboxylate (946.1 mg, 1.0

equiv, 5.0 mmol) in anhydrous Et_2O (25.0 mL, 0.2 M) was stirred in a 100 mL 2 necked flask and cooled in an ice bath. Oxalyl chloride (846.2 µL, 2.0 equiv, 10.0 mmol) was added dropwise and the stirring was continued for 3 h at room temperature. Then, under a positive flow of N_2, $HNMe_2$ (2.0 M in THF) was added into the reaction with vigorous stirring until a pH between 9-11 was achieved (determined by moist pH paper). After 16 h, the reaction was diluted with 15 mL of cold Et_2O and the white solid was collected by Buchner filtration. The filter cake was suspended in distilled H_2O (50 mL) and stirred for 1 h to remove dimethylamine hydrochloride. After this time, the slurry was filtered, washed with H_2O (2 × 30 mL), Et_2O (3 × 30 mL), hexane (3 × 30 mL) and dried overnight in a desiccator to afford methyl 3-(2-(dimethylamino)-2-oxoacetyl)-1-methyl-1H-indole-6-carboxylate (**4d-ester**) as a white solid (1.05 g, 73%).

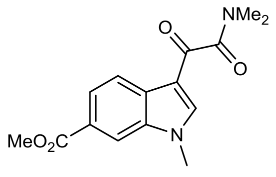

^1H NMR (400 MHz, $(CD_3)_2CO$) δ 8.31 (d, J = 8.3 Hz, 1H), 8.20 (m, 2H), 7.96 (d, J = 8.3 Hz, 1H), 4.07 (s, 3H), 3.92 (s, 3H), 3.04 (s, 3H), 3.02 (s, 3H); ^{13}C NMR (101 MHz, $(CD_3)_2CO$) δ 187.0, 168.0, 167.6, 143.1, 138.3, 130.6, 126.3, 124.3, 122.2, 113.8, 113.4, 52.3, 37.3, 34.1, 34.0; m.p. 144-145 °C; IR (ATR) 3130, 2951, 2367, 2332, 1714, 1627, 1615, 1372, 980; HRMS (ESI) m/z calcd. $C_{15}H_{17}N_2O_4$: $[M+H]^+$ 289.1183; found $[M+H]^+$: 289.1180.

A solution of 3-(2-(dimethylamino)-2-oxoacetyl)-1-methyl-1H-indole-6-carboxylate **4d-ester** (864.9 mg, 1.0 equiv, 3.0 mmol) and LiOH (143.7 mg, 2.0 equiv, 6.0 mmol) in THF/H_2O (30.0 mL, 1:1 ratio, 0.1 M) was stirred in a 100 mL flask at room temperature for 3 h. After this time, THF solvent was removed under reduced pressure. The aqueous layer was washed with EtOAc (3 × 30 mL), acidified until a pH of 1 was obtained with HCl (2.0 M), extracted with EtOAc (3 × 30 mL), dried over $MgSO_4$ and evaporated to dryness to afford 3-(2-(dimethylamino)-2-oxoacetyl)-1-methyl-1H-indole-6-carboxylic acid (**4d**) as a white solid (757.0 mg, 92%).

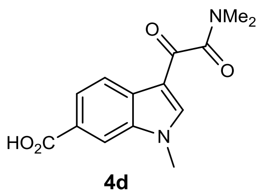

4d

^1H NMR (400 MHz, $(CD_3)_2CO$) δ 8.31 (dd, J = 8.3, 0.6 Hz, 1H), 8.23 (dd, J = 1.4, 0.7 Hz, 1H), 8.19 (s, 1H), 8.00 (dd, J = 8.3, 1.4 Hz, 1H), 4.07 (s, 3H), 3.05 (s, 3H), 3.03 (s, 3H);

^{13}C NMR (126 MHz, $(CD_3)_2CO$) δ 187.9, 169.0, 168.8, 143.9, 139.3, 131.4, 127.5, 125.5, 123.0, 114.7, 114.6, 38.2, 35.0, 34.9; m.p. 232-234 °C; IR (ATR) 3001, 2936, 1710, 1616, 1610, 1376, 779; HRMS (ESI) *m/z* calcd. $C_{14}H_{15}N_2O_4$: $[M+H]^+$ 275.1026; found $[M+H]^+$: 275.1026.

Procedure for the preparation of $KOC(CF_3)_3$

A round bottom flask equipped with a magnetic stirrer bar was loaded with 50.0 mL (50.0 mmol) of KOH solution (1.0 M in EtOH) and placed in an ice bath. Subsequently, a solution of $(CF_3)_3COH$ in EtOH (55.0 mL, 55.0 mmol, 1.0 M) was added at 0 °C. Then the ice bath was removed and the solution allowed stirring at room temperature for 30 min. The solvent was removed under reduced pressure affording the salt as a white solid, which is recrystallized from iPr_2O/hexane (12.75 g, 93%). If the purity of the salt is less than 95% (judged by quantitative ^{19}F NMR with a suitable internal standard), the yield of the arylation reaction dramatically decreases.

^{19}F NMR (471 MHz, $(CD_3)_3CO$) δ –77.39 (s, 9F); ^{13}C NMR (126 MHz, $(CD_3)_3CO$) δ 124.1 (q, *J* = 296.3 Hz, 3C), 85.3 - 84.2 (m, 1C); m.p. >250 °C; IR (ATR) 1224, 1186, 956, 720.

Optimisation Table

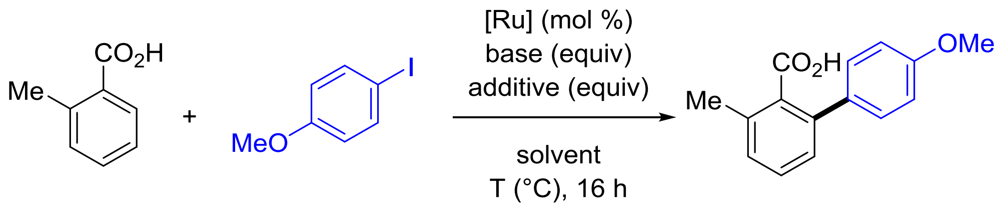

1a **2a** **3aa**

entry	[Ru] (mol %)	base (equiv)	additive (equiv)	T (°C)	Solvent	**3aa** (%)
1	$[RuCl_2(p\text{-cymene})]_2$ (2.5)	K_2CO_3 (2)	none	100	1,4-dioxane [0.5 M]	2
2	$[RuCl_2(C_6Me_6)]_2$ (2.5)	K_2CO_3 (2)	none	100	1,4-dioxane [0.5 M]	< 1
3	$[Ru(^tBuCN)_6](BF_4)_2$ (5)	K_2CO_3 (2)	none	100	1,4-dioxane [0.5 M]	7
4	Ru_3CO_{12} (1.7)	K_2CO_3 (2)	none	100	1,4-dioxane [0.5 M]	0
5	$[Ru(^tBuCN)_6](BF_4)_2$ (5)	K_2CO_3 (2)	none	100	*m*-xylene [0.5 M]	0
6	$[Ru(^tBuCN)_6](BF_4)_2$ (5)	K_2CO_3 (2)	none	100	DCE [0.5 M]	2
7	$[Ru(^tBuCN)_6](BF_4)_2$ (5)	K_2CO_3 (2)	none	100	NMP [0.5 M]	20
8	$[Ru(^tBuCN)_6](BF_4)_2$ (5)	K_2CO_3 (2)	none	100	DMSO [0.5 M]	0
9	$[Ru(^tBuCN)_6](BF_4)_2$ (5)	K_2CO_3 (2)	none	100	DMF [0.5 M]	6
10	$[Ru(^tBuCN)_6](BF_4)_2$ (5)	K_2CO_3 (2)	none	100	MeCN [0.5 M]	15
11	$[Ru(^tBuCN)_6](BF_4)_2$ (5)	K_2CO_3 (2)	none	100	^{t}BuCN [0.5 M]	41
12	$[Ru(^tBuCN)_6](BF_4)_2$ (5)	Li_2CO_3 (2)	none	100	^{t}BuCN [0.5 M]	< 1
13	$[Ru(^tBuCN)_6](BF_4)_2$ (5)	Na_2CO_3 (2)	none	100	^{t}BuCN [0.5 M]	< 1
14	$[Ru(^tBuCN)_6](BF_4)_2$ (5)	Cs_2CO_3 (2)	none	100	^{t}BuCN [0.5 M]	14
15	$[Ru(^tBuCN)_6](BF_4)_2$ (5)	K_2CO_3 (2)	$P(4\text{-OMe-}C_6H_4)_3$ (0.05)	100	^{t}BuCN [0.5 M]	7
16	$[Ru(^tBuCN)_6](BF_4)_2$ (5)	K_2CO_3 (2)	$P(4\text{-F-}C_6H_4)_3$ (0.05)	100	^{t}BuCN [0.5 M]	0
17	$[Ru(^tBuCN)_6](BF_4)_2$ (5)	K_2CO_3 (2)	PCy_3•HBF_4 (0.05)	100	^{t}BuCN [0.5 M]	40
18	$[Ru(^tBuCN)_6](BF_4)_2$ (5)	K_2CO_3 (2)	SIPr•HCl (0.05)	100	^{t}BuCN [0.5 M]	39
19	$[Ru(^tBuCN)_6](BF_4)_2$ (5)	K_2CO_3 (2)	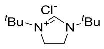 (0.05)	100	^{t}BuCN [0.5 M]	10
20	$[Ru(^tBuCN)_6](BF_4)_2$ (5)	K_2CO_3 (2)	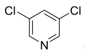 (0.05)	100	^{t}BuCN [0.5 M]	40
21	$[Ru(^tBuCN)_6](BF_4)_2$ (5)	K_2CO_3 (2)	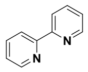 (0.05)	100	^{t}BuCN [0.5 M]	7
22	$[Ru(^tBuCN)_6](BF_4)_2$ (5)	K_2CO_3 (2)	KOAc (1)	100	^{t}BuCN [0.5 M]	26
23	$[Ru(^tBuCN)_6](BF_4)_2$ (5)	K_2CO_3 (2)	KOPiv (1)	100	^{t}BuCN [0.5 M]	51
24	$[Ru(^tBuCN)_6](BF_4)_2$ (5)	K_2CO_3 (2)	KOAd (1)	100	^{t}BuCN [0.5 M]	15
25	$[Ru(^tBuCN)_6](BF_4)_2$ (5)	K_2CO_3 (2)	$KOC(CF_3)_3$ (1)	100	^{t}BuCN [0.5 M]	61
26	$[Ru(^tBuCN)_6](BF_4)_2$ (5)	K_2CO_3 (2)	$KOC(CF_3)_3$ (1)	120	^{t}BuCN [0.5 M]	91

27	$[Ru(^tBuCN)_6](BF_4)_2$ (5)	K_2CO_3 (2)	$KOC(CF_3)_3$ (1)	130	^tBuCN [0.5 M]	92
28	$[Ru(^tBuCN)_6](BF_4)_2$ (5)	K_2CO_3 (2)	$KOC(CF_3)_3$ (1)	140	^tBuCN [0.5 M]	99
29	$[Ru(^tBuCN)_6](BF_4)_2$ (5)	K_2CO_3 (2)	$KOC(CF_3)_3$ (1)	150	^tBuCN (5 equiv)	66
30	$[Ru(^tBuCN)_6](BF_4)_2$ (5)	K_2CO_3 (2)	$KOC(CF_3)_3$ (1)	140	^tBuCN (3 equiv)	94
31	$[Ru(^tBuCN)_6](BF_4)_2$ (5)	K_2CO_3 (2)	$KOC(CF_3)_3$ (1)	140	^tBuCN (5 equiv)	95
32	$[Ru(^tBuCN)_6](BF_4)_2$ (5)	K_2CO_3 (2)	$KOC(CF_3)_3$ (1)	140	^tBuCN (8 equiv)	99
33	$[Ru(^tBuCN)_6](BF_4)_2$ (5)	K_2CO_3 (2)	$KOC(CF_3)_3$ (1)	140	^tBuCN (15 equiv)	96
34	$[Ru(^tBuCN)_6](BF_4)_2$ (3)	K_2CO_3 (2)	$KOC(CF_3)_3$ (1)	140	^tBuCN (8 equiv)	99
35[b]	$[Ru(^tBuCN)_6](BF_4)_2$ (3)	K_2CO_3 (2)	$KOC(CF_3)_3$ (1)	140	^tBuCN (8 equiv)	0

[a] Reaction conditions: **1a** (0.30 mmol, 1.0 equiv), **2a** (2.0 equiv), [Ru] (*see above*), base and additive (*see above*) were stirred under N_2 in a closed vessel in the specified solvent (*see above*) at stated temperature (*see above*) for 16 h; yield is estimated by ^1H NMR using 1,3-dinitrobenzene as internal standard. [b] under air.

Experimental characterization data for products

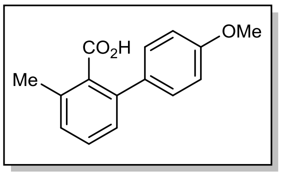

2-(4-methoxyphenyl)-6-methylbenzoic acid (**3aa**)

The General Procedure **A** was applied with 2-methylbenzoic acid (40.8 mg, 0.30 mmol) and 4-iodoanisole (140.4 mg, 2.0 equiv, 0.60 mmol). Column chromatography (hexane/EtOAc/AcOH 80:20:1) afforded the title product as a white solid (71.2 mg, 98%).

Spectroscopic data matched those previously reported.[5]

^{1}H NMR (400 MHz, $(CD_3)_3CO$) δ 7.38 (app t, J = 7.6 Hz, 1H), 7.32 - 7.27 (m, 2H), 7.23 (d, J = 7.6 Hz, 1H), 7.01 - 7.00 (m, 2H,), 6.91 (dd, J = 8.3, 1.7 Hz, 1H), 3.81 (s, 3H), 2.41 (s, 3H);^{13}C NMR (101 MHz, $(CD_3)_3CO$) δ 171.0, 160.5, 143.3, 140.0, 135.4, 130.1, 129.9, 129.7, 127.9, 121.6, 114.9, 113.8, 55.5, 19.8.

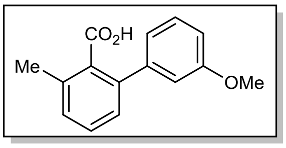

2-(3-methoxyphenyl)-6-methylbenzoic acid (**3ab**)

The General Procedure **A** was applied with 2-methylbenzoic acid (40.8 mg, 0.30 mmol) and 3-iodoanisole (71.5 µL, 2.0 equiv, 0.60 mmol). Column chromatography (hexane/EtOAc/AcOH 80:20:1) afforded the title product as a white solid (45.8 mg, 63%).

^{1}H NMR (400 MHz, $(CD_3)_2CO$) δ 7.38 (app t, J = 7.6 Hz, 1H), 7.32 - 7.27 (m, 2H) 7.23 (d, J = 7.6 Hz, 1H), 7.01 - 7.00 (m, 2H), 6.91 (dd, J = 8.3, 1.7 Hz, 1H), 3.81 (s, 3H), 2.41 (s, 3H); ^{13}C NMR (101 MHz, $(CD_3)_2CO$) δ 171.0, 160.5, 143.3, 140.0, 135.4, 130.1, 129.9, 129.7, 127.9, 121.6, 114.9, 113.8, 55.5, 19.8; m.p. 140-142 °C; IR (ATR) 3030, 2917, 1721, 1284, 1237, 1169; HRMS (ESI) m/z calcd. $C_{15}H_{13}NaO_3$: $[M–H]^-$ 241.0870; found: $[M–H]^-$ 241.0859.

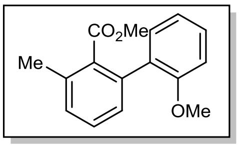

methyl 2'-methoxy-3-methyl-[1,1'-biphenyl]-2-carboxylate (**3ac**)

The General Procedure **A** was applied with 2-methylbenzoic acid (40.8 mg, 0.30 mmol) and 2-iodoanisole (78.1 µL, 2.0 equiv, 0.6

mmol) with [Ru(^{t}BuCN)$_6$](BF_4)$_2$ (13.9 mg, 6 mol %) and H_2O (16.2 µL, 3.0 equiv, 0.9 mmol). Column chromatography (0-20% EtOAc-hexane), after derivatization with MeI, afforded the title product as a colourless oil (52.3 mg, 68%).

Spectroscopic data matched those previously reported.[6]

^{1}H NMR (400 MHz, $CDCl_3$) δ 7.37 - 7.29 (m, 2H), 7.22 - 7.18 (m, 3H), 6.99 (td, *J* = 7.4, 1.0 Hz, 1H), 6.93 (dd, *J* = 8.2, 0.6 Hz, 1H), 3.75 (s, 3H), 3.54 (s, 3H), 2.44 (s, 3H); ^{13}C NMR (101 MHz, $CDCl_3$) δ 169.8, 156.3, 137.5, 135.9, 133.4, 130.7, 130.1, 129.5, 129.5, 129.0, 128.5, 120.6, 110.6, 55.5, 51.5, 20.4.

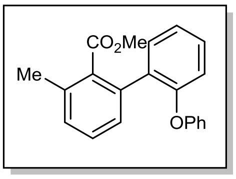

methyl 3-methyl-2'-phenoxy-[1,1'-biphenyl]-2-carboxylate (**3ad**)

The General Procedure **A** was applied with 2-methylbenzoic acid (40.8 mg, 0.30 mmol) and 1-iodo-2-phenoxybenzene (177.7 mg, 2.0 equiv, 0.60 mmol) with [Ru(^{t}BuCN)$_6$](BF_4)$_2$ (13.9 mg, 6 mol %). Column chromatography (0-20% EtOAc-hexane), after derivatization with MeI, afforded the title product as a colourless oil (76.4 mg, 80%).

^{1}H NMR (400 MHz, $CDCl_3$) δ 7.28 - 7.18 (m, 6H), 7.14 - 7.09 (m, 2H), 6.97 (app tt, *J* = 8.0, 1.0 Hz, 1H), 6.93 - 6.87 (m, 3H), 3.57 (s, 3H), 2.37 (s, 3H); ^{13}C NMR (101 MHz, $CDCl_3$) δ 169.8, 157.6, 153.9, 136.6, 136.0, 133.4, 132.9, 131.1, 129.7, 129.6, 129.3, 129.0, 128.4, 123.4, 122.9, 119.2, 118.7, 51.7, 20.4; m.p. 120-125 °C; IR (ATR) 3069, 2945, 1726, 1488, 1261, 1067, 750; HRMS (ESI) *m/z* calcd. $C_{21}H_{19}O_3$: $[M+H]^+$ 319.1329; found: $[M+H]^+$ 319.1325.

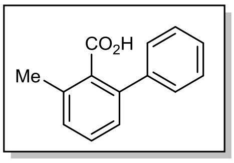

3-methyl-[1,1'-biphenyl]-2-carboxylic acid (**3ae**)

The General Procedure **A** was applied with 2-methylbenzoic acid (40.8 mg, 0.30 mmol) and iodobenzene (66.9 µL, 2.0 equiv, 0.60 mmol). Column chromatography (hexane/EtOAc/AcOH 80:20:1) afforded the title product as a white solid (61.1 mg, 96%).

Spectroscopic data matched those previously reported.[7]

^{1}H NMR (400 MHz, $(CD_3)_2CO$) δ 7.45 - 7.32 (m, 6H), 7.29 (ddd, *J* = 7.7, 1.2, 0.7 Hz, 1H), 7.22 (ddd, *J* = 7.6, 1.2, 0.6 Hz, 1H), 2.42 (s, 3H); ^{13}C NMR (101 MHz, $(CD_3)_2CO$) δ 170.8, 141.9, 140.2, 135.5, 135.2, 129.9, 129.8, 129.3, 129.1, 128.2, 128.1, 19.8.

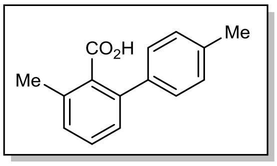

2-methyl-6-(4-methylphenyl)benzoic acid (**3af**)

The General Procedure **A** was applied with 2-methylbenzoic acid (40.8 mg, 0.30 mmol) and 1-iodo-4-methylbenzene (130.8 mg, 2.0 equiv, 0.60 mmol). Column chromatography (hexane/EtOAc/AcOH 80:20:1) afforded the title product as a white solid (66.5 mg, 98%).

Spectroscopic data matched those previously reported.[8]

^{1}H NMR (500 MHz, $CDCl_3$) δ 7.37 (app t, *J* = 7.6 Hz, 1H), 7.32 (app d, *J* = 7.3 Hz, 2H) 7.23 - 7.19 (m, 4H), 2.46 (s, 3H), 2.40 (s, 3H); ^{13}C NMR (125.8 MHz, $CDCl_3$) δ 174.9, 140.2, 137.9, 137.4, 135.5, 132.2, 129.9, 129.3, 129.1, 128.4, 127.6, 21.4, 20.0.

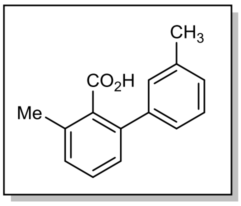

3,3'-dimethyl-[1,1'-biphenyl]-2-carboxylic acid (**3ag**)

The General Procedure **A** was applied with 2-methylbenzoic acid (40.8 mg, 0.30 mmol) and 1-iodo-3-methylbenzene (77.0 µL, 2.0 equiv, 0.60 mmol). Column chromatography (hexane/EtOAc/AcOH 80:20:1) afforded the title product as a white solid (63.8 mg, 94%).

Spectroscopic data matched those previously reported.[9]

^{1}H NMR (400 MHz, $(CD_3)_2CO$) δ 7.38 (app t, *J* = 7.6 Hz, 1H), 7.29 - 7.15 (m, 6H), 2.44 (s, 3H), 2.35 (s, 3H); ^{13}C NMR (101 MHz, $(CD_3)_2CO$) δ 170.9, 141.9, 140.3, 138.5, 135.4, 135.2, 130.0, 129.8, 129.7, 129.0, 128.8, 128.0, 126.4, 21.4, 19.8.

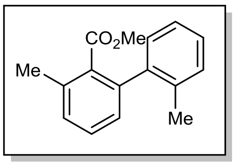

methyl 2',3-dimethyl-[1,1'-biphenyl]-2-carboxylate (**3ah**)

The General Procedure **A** was applied with 2-methylbenzoic acid (40.8 mg, 0.30 mmol) and 1-iodo-2-methylbenzene (76.4 µL, 2.0 equiv, 0.60 mmol) with [Ru(*t*BuCN)$_6$](BF$_4$)$_2$ (13.9 mg, 6 mol %) and H_2O (16.2 µL, 3.0 equiv, 0.90 mmol). Column chromatography (0-20% EtOAc-hexane), after derivatization with MeI, afforded the title product as a colourless oil (30.3 mg, 42%).

^{1}H NMR (400 MHz, $CDCl_3$) δ 7.33 (t, *J* = 7.6 Hz, 1H), 7.23 - 7.20 (m, 3H), 7.18 - 7.14 (m, 1H), 7.10 (dm, *J* = 7.3 Hz, 1H), 7.06 (ddd, *J* = 7.6, 1.2, 0.6 Hz, 1H), 3.46 (s, 3H), 2.40 (s, 3H), 2.13 (s, 3H); ^{13}C NMR (101 MHz, $CDCl_3$) δ 169.9, 140.3, 140.1, 136.2, 135.4, 133.9, 129.9, 129.3, 129.1, 129.1, 127.7, 127.3, 125.2, 51.7, 20.2, 19.9; m.p. 90-93 °C; IR (ATR) 3040, 2957, 2845, 1728, 1269, 1112, 759; HRMS (ESI) *m/z* calcd. $C_{16}H_{16}O_2Na$: $[M+Na]^+$ 263.1043; found: $[M+Na]^+$ 263.1037.

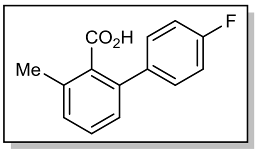

4'-fluoro-3-methyl-[1,1'-biphenyl]-2-carboxylic acid (**3ai**)

The General Procedure **A** was applied with 2-methylbenzoic acid (40.8 mg, 0.30 mmol) and 1-fluoro-4-iodobenzene (69.2 µL, 2.0 equiv, 0.60 mmol). Column chromatography (hexane/EtOAc/AcOH 80:20:1) afforded the title product as a white solid (60.1 mg, 87%).

^{1}H NMR (400 MHz, $(CD_3)_2CO$) δ 7.48 - 7.43 (m, 2H), 7.39 (app t, *J* = 7.6 Hz, 1H), 7.29 (ddd, *J* = 7.7, 1.2, 0.6 Hz, 1H), 7.21 (ddd, *J* = 7.7, 1.2, 0.6 Hz, 1H), 7.19 - 7.15 (m, 2H), 2.42 (s, 3H); ^{13}C NMR (101 MHz, $(CD_3)_2CO$) δ 170.7, 163.2 (d, *J* = 244.5 Hz), 139.1, 138.2 (d, *J* = 3.3 Hz), 135.6, 135.3, 131.3 (d, *J* = 8.2 Hz), 130.0 (d, *J* = 8.3 Hz), 128.1, 115.9, 115.7, 19.8; ^{19}F NMR (376 MHz, $(CD_3)_2CO$) δ –117.05 (tt, *J* = 9.0, 5.4 Hz, 1F); m.p. 126-129 °C; IR (ATR) 3040, 2940, 2731, 1690, 1700, 750; HRMS (ESI) *m/z* calcd. $C_{14}H_{11}FNaO_2$: $[M+Na]^+$ 253.0635; found: $[M+Na]^+$ 253.0634.

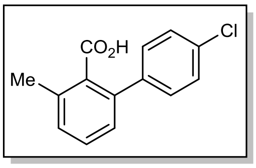

2-(4-chlorophenyl)-6-methylbenzoic acid (**3aj**)

The General Procedure **A** was applied with 2-methylbenzoic acid (40.8 mg, 0.30 mmol) and 1-chloro-4-iodobenzene (143.1

mg, 2.0 equiv, 0.60 mmol). Column chromatography (hexane/EtOAc/AcOH 80:20:1) afforded the title product as a white solid (68.8 mg, 93%).

^{1}H NMR (500 MHz, $CDCl_3$) δ 7.39 - 7.33 (m, 5H), 7.25 (app d, J = 7.3 Hz, 1H), 7.18 (d, J = 7.6 Hz, 1H), 2.46 (s, 3H); ^{13}C NMR (125.8 MHz, $CDCl_3$) δ 174.3, 139.2, 139.1, 135.8, 133.9, 132.1, 130.0, 129.9, 129.7, 128.7, 127.5, 20.0; m.p. 128-130 °C; IR (ATR) 3025, 2964, 1682, 1288, 830; HRMS (ESI) *m/z* calcd. $C_{14}H_{10}ClO_2$: $[M–H]^−$ 245.0635; found: $[M–H]^−$ 245.0375.

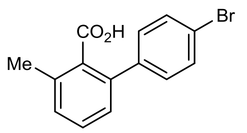

4'-bromo-3-methyl-[1,1'-biphenyl]-2-carboxylic acid (**3ak**)

The General Procedure **A** was applied with 2-methylbenzoic acid (40.8 mg, 0.30 mmol) and 1-bromo-4-iodobenzene (169.7 mg, 2.0 equiv, 0.60 mmol) for 3 h. Column chromatography (hexane/EtOAc/AcOH 80:20:1) afforded the title product as a white solid (66.4 mg, 76%).

^{1}H NMR (400 MHz, $CDCl_3$) δ 7.53 - 7.50 (m, 2H), 7.37 (app t, J = 7.7 Hz, 1H), 7.29 - 7.24 (m, 3H), 7.17 (d, J = 7.5 Hz, 1H), 2.45 (s, 3H); ^{13}C NMR (101 MHz, $CDCl_3$) δ 174.5, 139.7, 139.1, 135.8, 132.2, 131.6, 130.2, 130.0, 129.8, 127.4, 122.1, 20.0; m.p. 112-115 °C; IR (ATR) 3057, 2927, 2848, 2674, 2547, 1687, 1289, 783; HRMS (ESI) *m/z* calcd. $C_{14}H_{12}BrO_2$: $[M+H]^+$ 291.0015; found: $[M+H]^+$ 291.0001.

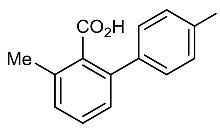

3al

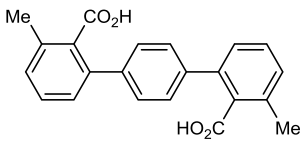

3al'

4'-iodo-3-methyl-[1,1'-biphenyl]-2-carboxylic acid (**3al**) and 3,3''-dimethyl-[1,1':4',1''-terphenyl]-2,2''-dicarboxylic acid (**3al'**)

The General Procedure **A** was applied with 2-methylbenzoic acid (40.8 mg, 0.30 mmol) and 1,4-diiodobenzene (197.9 mg, 2.0 equiv, 0.60 mmol) for 1 h. Column chromatography (hexane/EtOAc/AcOH 80:20:1) afforded 4'-iodo-3-methyl-[1,1'-biphenyl]-2-carboxylic acid (64.9 mg, 64%) and 3,3''-dimethyl-[1,1':4',1''-terphenyl]-2,2''-dicarboxylic acid (15.6 mg, 15%) as white solids.

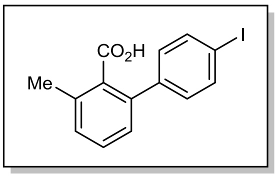

4'-iodo-3-methyl-[1,1'-biphenyl]-2-carboxylic acid (**3al**)

^{1}H NMR (500 MHz, $(CD_3)_2CO$) δ 11.37 (br s, 1H), 7.79 (d, *J* = 8.3 Hz, 2H), 7.40 (app t, *J* = 7.7 Hz, 1H), 7.3 (app d, *J* = 7.6 Hz, 1H), 7.25 - 7.21 (m, 3H), 2.42 (s, 3H); ^{13}C NMR δ 170.6, 141.6, 139.1, 138.2, 135.8, 135.0, 131.5, 130.3, 130.1, 127.9, 93.6, 19.8, m.p. 144-146 °C; IR (ATR) 3020, 2950, 1687, 1320, 762; HRMS (ESI) *m/z* calcd. $C_{14}H_{11}INaO_2$: $[M+Na]^+$ 360.9696; found: $[M+Na]^+$ 360.9691.

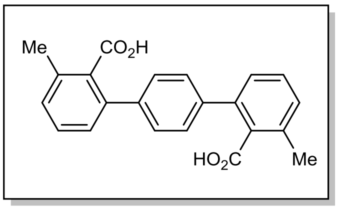

3,3''-dimethyl-[1,1':4',1''-terphenyl]-2,2''-dicarboxylic acid (**3al'**)

^{1}H NMR (400 MHz, $(CD_3)_2SO$) δ 7.46 (s, 4H), 7.40 (app t, *J* = 7.6 Hz, 2H), 7.29 (d, *J* = 7.5 Hz, 2H), 7.23 (d, *J* = 7.6 Hz, 2H), 2.35 (s, 6H); ^{13}C NMR (101 MHz, $(CD_3)_2SO$) δ 170.6, 139.5, 137.7, 134.9, 133.9, 129.0, 128.8, 128.2, 127.1, 19.3; m.p. >250 °C; IR (ATR) 3080, 2974, 2886, 2647, 2583, 1682, 1302, 1138, 783; HRMS (ESI) *m/z* calcd. $C_{22}H_{17}O_4$: $[M–H]^–$ 345.1127; found: $[M–Na]^–$ 345.1114.

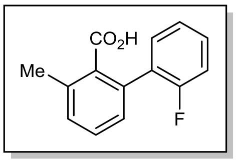

2'-fluoro-3-methyl-[1,1'-biphenyl]-2-carboxylic acid (**3am**)

The General Procedure **A** was applied with 2-methylbenzoic acid (40.8 mg, 0.30 mmol) and 2-fluoroiodobenzene (70.0 µL, 2.0 equiv, 0.60 mmol) with $[Ru(^tBuCN)_6](BF_4)_2$ (13.9 mg, 6 mol %) and H_2O (16.2 µL, 3.0 equiv, 0.90 mmol). Column chromatography (hexane/EtOAc/AcOH 80:20:1) afforded the title product as a white solid (44.2 mg, 64%).

^{1}H NMR (400 MHz, $CDCl_3$) δ 7.37 (t, *J* = 7.7 Hz, 1H), 7.33 - 7.23 (m, 3H), 7.18 (app d, *J* = 7.6 Hz, 1H), 7.13 (td, *J* = 7.6, 1.1 Hz, 1H), 7.07 (app t, *J* = 9.1 Hz, 1H), 2.46 (s, 3H); ^{13}C NMR (101 MHz, $CDCl_3$) δ 174.3, 159.7 (d, *J* = 246.3 Hz), 136.6, 134.8, 132.3 (d, *J* = 4.6 Hz), 131.1 (d, *J* = 3.2 Hz), 130.4, 130.1, 129.6 (d, *J* = 8.1 Hz), 128.5, 128.4 (d, *J* = 15.9 Hz), 124.1 (d, *J* = 3.7 Hz), 115.6 (d, *J* = 22.3 Hz), 20.6; ^{19}F NMR (376 MHz, $CDCl_3$) δ –115.87 (ddd, *J* = 10.2, 7.4, 5.5 Hz, 1F); m.p. 118-120 °C; IR (ATR) 3057, 2924, 2650, 2550, 2350, 1700, 1291, 756; HRMS (ESI) *m/z* calcd. $C_{14}H_{11}FNaO_2$: $[M+Na]^+$ 253.0635; found: $[M+Na]^+$ 253.0631.

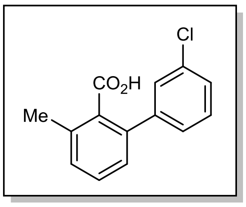

3'-chloro-3-methyl-[1,1'-biphenyl]-2-carboxylic acid (**3an**)

The General Procedure **A** was applied with 2-methylbenzoic acid (40.8 mg, 0.30 mmol) and 3-chloroiodobenzene (74.3 µL, 2.0 equiv, 0.60 mmol). Column chromatography (hexane/EtOAc/AcOH 80:20:1) afforded the title product as a white solid (60.7 mg, 82%).

^{1}H NMR (400 MHz, CD_3CN) δ 7.43 - 7.39 (m, 4H), 7.36 - 7.30 (m, 2H), 7.24 - 7.22 (app d, *J* = 7.6 Hz, 1H), 2.39 (s, 3H); ^{13}C NMR (101 MHz, $(CD_3)_2CO$) δ 170.5, 144.0, 138.7, 135.8, 135.1, 134.4, 130.8, 130.5, 130.1, 129.2, 128.2, 128.0, 127.9, 19.8; m.p. 119-123 °C; IR (ATR) 3057, 2924, 2641, 2529, 1690, 1458, 1287, 778; HRMS (ESI) *m/z* calcd. $C_{14}H_{10}ClO_2$: $[M–H]^-$ 245.0375; found: $[M–H]^-$ 245.0365.

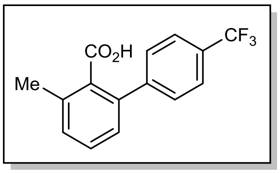

2-methyl-6-[4-(trifluoromethyl)phenyl]benzoic acid (**3ao**)

The General Procedure **A** was applied with 2-methylbenzoic acid (40.8 mg, 0.30 mmol) and 4-iodobenzotrifluoride (88.2 µL, 2.0 equiv, 0.60 mmol). Column chromatography (hexane/EtOAc/AcOH 80:20:1) afforded the title product as a white solid (67.2 mg, 80%).

Spectroscopic data matched those previously reported.[9]

^{1}H NMR (500 MHz, $CDCl_3$) δ 7.64 (app d, *J* = 8.0 Hz, 2H), 7.51 (app d, *J* = 8.0 Hz, 2H), 7.41 (app t, *J* = 7.7 Hz, 1H), 7.29 (d, *J* = 7.7 Hz, 1H), 7.20 (d, *J* = 7.6 Hz, 1H), 2.46 (s, 3H); ^{13}C NMR (126 MHz, $CDCl_3$); δ 174.5, 144.5, 139.0, 136.1, 132.0, 130.2, 130.2, 129.9 (q, *J* = 32.6 Hz), 128.9, 127.5, 125.4 (q, *J* = 3.8 Hz), 124.3 (q, *J* = 272.1 Hz), 20.0; ^{19}F NMR (376 MHz, CD_3Cl_3) δ –62.49 (s, 3F).

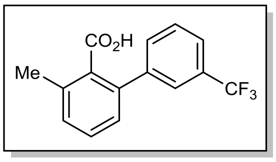

3-methyl-3'-(trifluoromethyl)-[1,1'-biphenyl]-2-carboxylic acid (**3ap**)

The General Procedure **A** was applied with 2-methylbenzoic acid (40.8 mg, 0.30 mmol) and 3-iodobenzotrifluoride (86.5 µL, 2.0 equiv, 0.60 mmol). Column chromatography (hexane/EtOAc/AcOH 80:20:1) afforded the title product as a white solid (63.9 mg, 76%).

^{1}H NMR (400 MHz, $(CD_3)_2CO$) δ 7.74 - 7.64 (m, 4H), 7.45 (t, J = 7.6 Hz, 1H), 7.37 (ddd, J = 7.7, 1.2, 0.6 Hz, 1H), 7.29 (ddd, J = 7.6, 1.2, 0.6 Hz, 1H), 2.44 (s, 3H); ^{13}C NMR (101 MHz, $(CD_3)_2CO$) δ 170.5, 142.9, 138.7, 135.9, 135.2, 133.2, 130.9 (q, J = 31.9 Hz), 130.7, 130.2, 130.2, 128.1, 125.9 (q, J = 3.9 Hz), 125.3 (q, J = 271.7 Hz), 125.0 (q, J = 3.8 Hz), 19.8; ^{19}F NMR (376 MHz, $(CD_3)_2CO$) δ –63.07 (s, 3F); m.p. 140-142 °C, IR (ATR) 3071, 2962, 2880, 1698, 1333, 1125, 807, 703; HRMS (ESI) *m/z* calcd. $C_{15}H_{10}F_3O_2$: $[M–H]^-$ 279.0630; found: $[M–H]^-$ 279.0630.

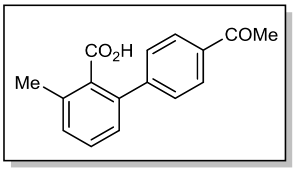

4'-acetyl-3-methyl-[1,1'-biphenyl]-2-carboxylic acid (**3aq**)

The General Procedure **A** was applied with 2-methylbenzoic acid (40.8 mg, 0.30 mmol) and 4-iodoacetophenone (147.6 mg, 2.0 equiv, 0.60 mmol) for 3 h. Column chromatography (hexane/EtOAc/AcOH 80:20:1) afforded the title product as a white solid (54.2 mg, 71%).

^{1}H NMR (400 MHz, $CDCl_3$) δ 7.98 (dm, J = 8.5 Hz, 1H), 7.51 (dm, J = 8.5 Hz, 1H), 7.40 (app t, J = 7.7 Hz, 1H), 7.28 (app d, J = 7.9 Hz, 2H), 7.21 (d, J = 7.6 Hz, 2H), 2.62 (s, 3H), 2.47 (s, 3H); ^{13}C NMR (101 MHz, $CDCl_3$) δ 198.4, 174.3, 145.9, 139.2, 136.1, 136.0, 132.2, 130.1, 130.0, 128.8, 128.6, 127.4, 26.7, 20.1; m.p. 154-156 °C; IR (ATR) 3074, 2951, 1717, 1677, 1320, 792, 728; HRMS (ESI) *m/z* calcd. $C_{16}H_{15}O_3$: $[M+H]^+$ 255.1016; found: $[M+H]^+$ 255.1012.

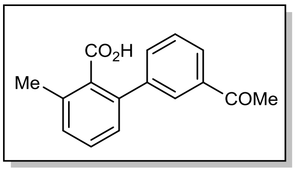

3'-acetyl-3-methyl-[1,1'-biphenyl]-2-carboxylic acid (**3ar**)

The General Procedure **A** was applied with 2-methylbenzoic acid (40.8 mg, 0.30 mmol) and 3-iodoacetophenone (83.5 µL, 2.0 equiv, 0.60 mmol) for 3 h. Column chromatography (hexane/EtOAc/AcOH 80:20:1) afforded the title product as a white solid (45.8 mg, 60%).

^{1}H NMR (400 MHz, $CDCl_3$) δ 8.00 (app s, 1H), 7.94 (app d, *J* = 7.8 Hz, 1H), 7.60 (app d, *J* = 7.8 Hz, 1H), 7.47 (app t, *J* = 7.7 Hz, 1H), 7.39 (app t, *J* = 7.7 Hz, 1H), 7.26 (d, *J* = 7.6 Hz, 1H), 7.22 (d, *J* = 7.6 Hz, 1H), 2.58 (s, 3H), 2.45 (s, 3H); ^{13}C NMR (101 MHz, $CDCl_3$) δ 198.4, 174.3, 141.3, 139.1, 137.2, 135.8, 133.2, 132.6, 129.9, 129.9, 128.8, 128.7, 127.5, 127.5, 26.8, 20.0; m.p.= 148-150 °C; IR (ATR) 3057, 2930, 2609, 1724, 1682, 1247; HRMS (ESI) *m/z* calcd. $C_{16}H_{15}O_3$: $[M+H]^+$ 255.1016; found: $[M+H]^+$ 255.1003.

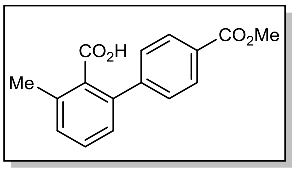

2-[4-(methoxycarbonyl)phenyl]-6-methylbenzoic acid (**3as**)

The General Procedure **A** was applied with 2-methylbenzoic acid (40.8 mg, 0.30 mmol) and methyl 4-iodobenzoate (157.2 mg, 2.0 equiv, 0.60 mmol) for 3 h. Column chromatography (hexane/EtOAc/AcOH 80:20:1) afforded the title product as a white solid (62.4 mg, 77%).

^{1}H NMR (500 MHz, $CDCl_3$) δ 8.05 (d, *J* = 7.4 Hz, 2H), 7.47 (d, *J* = 7.4 Hz, 2H), 7.39 (t, *J* = 7.3 Hz, 1H), 7.26 (d, *J* = 5.9 Hz, 1H), 7.21 (d, *J* = 7.4 Hz, 1H), 3.92 (s, 3H), 2.45 (s, 3H);^{13}C NMR (125.8 MHz, $CDCl_3$) δ 174.1, 167.2, 145.6, 139.3, 136.0, 132.3, 130.1, 130.0, 129.8, 129.3, 128.6, 127.4, 52.3, 20.1; m.p. 128–130 °C; IR (ATR) 3022, 2917, 1732, 1706, 1281, 768; HRMS (ESI) *m/z* calcd. $C_{16}H_{15}O_3$: $[M–H]^–$ 269.0814; found: $[M–H]^–$ 269.0811.

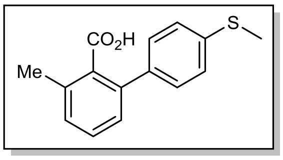

3-methyl-4'-(methylthio)-[1,1'-biphenyl]-2-carboxylic acid (**3at**)

The General Procedure **A** was applied with 2-methylbenzoic acid (40.8 mg, 0.30 mmol) and 4-iodothioanisole (150.1 mg, 2.0 equiv, 0.60 mmol). Column chromatography (hexane/EtOAc/AcOH 80:20:1) afforded the title product as a white solid (71.3 mg, 92%).

^{1}H NMR (400 MHz, $(CD_3)_2CO$) δ 7.40 - 7.36 (m, 3H), 7.31 - 7.26 (m, 3H), 7.21 (app d, J = 7.6 Hz, 1H), 2.52 (s, 3H), 2.42 (s, 3H); ^{13}C NMR (101 MHz, $(CD_3)_2CO$) δ 170.9, 139.6, 138.9, 138.4, 135.5, 135.2, 129.9, 129.8, 129.8, 127.9, 126.7, 19.8, 15.3; m.p. 82-88 °C; IR (ATR) 3060, 2921, 2629, 2523, 1698, 1459, 1244, 820; HRMS (ESI) *m/z* calcd. $C_{15}H_{14}NaO_2S$: $[M+Na]^+$ 281.0607; found: $[M+Na]^+$ 281.0607.

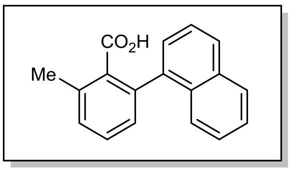

2-methyl-6-(naphthalen-1-yl)benzoic acid (**3au**)

The General Procedure **A** was applied with 2-methylbenzoic acid (40.8 mg, 0.30 mmol) and 1-iodonaphtalene (87.6 µL, 2.0 equiv, 0.60 mmol) with $[Ru(^tBuCN)_6](BF_4)_2$ (13.9 mg, 6 mol %). Column chromatography (hexane/EtOAc/AcOH 80:20:1) afforded the title product as a white solid (64.5 mg, 82%).

^{1}H NMR (500 MHz, $(CD_3)_2CO$) δ 10.99 (br s, 1H), 7.93 (d, J = 8.2 Hz, 1H), 7.91 (d, J = 8.3 Hz, 1H), 7.60 (d, J = 8.4 Hz, 1H), 7.52 - 7.38 (m, 6H), 7.20 (d, J = 7.5 Hz, 1H), 2.48 (s, 3H); ^{13}C NMR (126 MHz, $(CD_3)_2CO$) δ 170.1, 139.4, 138.8, 136.4, 135.7, 134.6, 133.1, 130.2, 129.5, 129.2, 128.9, 128.6, 127.8, 127.1, 126.7, 126.6, 125.9, 20.0; m.p 148-150 °C; IR (ATR) 3042, 2930, 1699, 1260, 820; HRMS (ESI) *m/z* calcd. $C_{18}H_{14}NaO_2$: $[M+Na]^+$ 285.0886; found: $[M+Na]^+$ 285.0881.

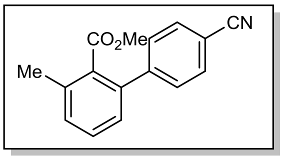

4'-cyano-3-methyl-[1,1'-biphenyl]-2-carboxylic acid (**3av**)

The General Procedure **A** was applied with 2-methylbenzoic acid (40.8 mg, 0.30 mmol) and 4-iodobenzonitrile (137.4 mg, 2.0 equiv, 0.60 mmol) for 1 h. Column chromatography (0-20% EtOAc-hexane), after derivatization with MeI, afforded the title product as a colourless oil (22.6 mg, 30%).

^{1}H NMR (500 MHz, $(CD_3)_2CO$) δ 7.85 (dm, J = 8.5 Hz, 2H), 7.55 (dm, J = 8.5 Hz, 2H), 7.48 (app t, J = 7.7 Hz, 1H), 7.38 (app d, J = 7.6 Hz, 1H), 7.30 (app d, J = 7.6 Hz, 1H), 3.60 (s, 3H), 2.38 (s, 3H); ^{13}C NMR (126 MHz, $(CD_3)_2CO$) δ 170.0, 146.5, 139.2, 136.7, 134.2, 133.1, 131.1, 130.7, 130.0, 127.9, 119.3, 112.1, 52.2, 19.7; IR (ATR) 3051, 2951, 2226, 1725, 1269, 1089, 790; HRMS (ESI) *m/z* calcd. $C_{16}H_{14}NO_2$: $[M+H]^+$ 252.1025; found: $[M+H]^+$ 252.1021.

2-methyl-6-(1-methyl-1H-indol-5-yl)benzoic acid (**3aw**)

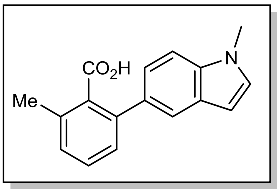

The General Procedure **A** was applied with 2-methylbenzoic acid (40.8 mg, 0.30 mmol) and 5-iodo-1-methyl-1*H*-indole (154.2 mg, 2.0 equiv, 0.60 mmol). Column chromatography (hexane/EtOAc/AcOH 80:20:1) afforded the title product as a white solid (75.6 mg, 95%).

^{1}H NMR (500 MHz, $(CD_3)_2CO$) δ 11.16 (br s, 1H), 7.63 (s, 1H), 7.40 (d, *J* = 8.4 Hz, 1H), 7.36 (t, *J* = 7.6 Hz, 1H), 7.28 - 7.23 (m, 4H), 6.45 (d, *J* = 3.0 Hz, 1H), 3.84 (s, 3H), 2.42 (s, 3H); ^{13}C NMR (126 MHz, $(CD_3)_2CO$) δ 171.2, 141.6, 137.2, 135.6, 135.2, 132.9, 130.6, 129.6, 129.5, 128.9, 128.5, 123.1, 121.3, 110.0, 101.7, 32.9, 19.8, m.p. 170-173 °C; IR (ATR) 3100, 2950, 2300, 1710, 1315, 780, 660; HRMS (ESI) *m/z* calcd. $C_{17}H_{16}NO_2$: $[M+H]^+$ 266.1174; found: $[M+H]^+$ 266.1174.

2-methyl-6-(thiophen-2-yl)benzoic acid (**3ax**)

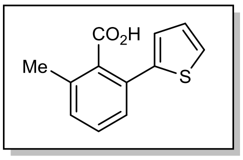

The General Procedure **A** was applied with 2-methylbenzoic acid (40.8 mg, 0.30 mmol) and 2-iodothiophene (66.3 µL, 2.0 equiv, 0.60 mmol). Column chromatography (hexane/EtOAc/AcOH 80:20:1) afforded the title product as a white solid (34.7 mg, 53%).

^{1}H NMR (500 MHz, $(CD_3)_2CO$) δ 7.49 (app d, *J* = 5.1 Hz, 1H), 7.38 - 7.33 (m, 2H), 7.28 (app d, *J* = 7.1 Hz, 1H), 7.23 (app d, *J* = 3.4 Hz, 1H), 7.10 - 7.07 (m, 1H), 2.40 (s, 3H); ^{13}C NMR (126 MHz, $(CD_3)_2CO$) δ 170.9, 142.6, 135.6, 135.5, 132.1, 130.3, 129.8, 128.4, 128.1, 127.1, 126.9, 19.6; m.p. 78-80 °C; IR (ATR) 3066, 2960, 1720, 1160, 850, 659; HRMS (ESI) *m/z* calcd. $C_{12}H_{10}NaO_2S$: $[M+Na]^+$ 241.0294; found: $[M+Na]^+$ 241.0291.

2-methoxy-6-(4-methoxyphenyl)benzoic acid (**3ba**)

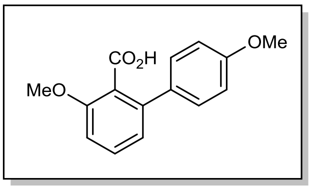

The General Procedure **A** was applied with 2-methoxybenzoic acid (45.6 mg, 0.30 mmol) and 4-iodoanisole (140.4 mg, 2.0 equiv, 0.60 mmol). Column

chromatography (hexane/EtOAc/AcOH 80:20:1) afforded the title product as a white solid (72.1 mg, 93%).

^{1}H NMR (500 MHz, $CDCl_3$) δ 7.25 - 7.23 (m, 3H), 6.83 (d, *J* = 7.5 Hz, 1H), 6.79 - 6.76 (m, 3H), 3.73 (s, 3H), 3.68 (s, 3H); ^{13}C NMR (126 MHz, $CDCl_3$) δ 173.6, 159.4, 156.6, 140.9, 132.3, 130.8, 129.6, 122.3, 122.3, 114.0, 109.7, 56.2, 55.3; m.p. 120-122 ℃; IR (ATR) 2945, 1732, 1464, 1240; HRMS (ESI) *m/z* calcd. $C_{15}H_{15}O_4$: $[M+H]^+$ 259.0965; found: $[M+H]^+$ 259.0970.

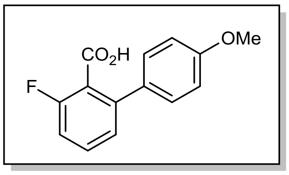

2-fluoro-6-(4-methoxyphenyl)benzoic acid (**3ca**)

The General Procedure **A** was applied with 2-fluorobenzoic acid (42.0 mg, 0.30 mmol) and 4-iodoanisole (140.4 mg, 2.0 equiv, 0.60 mmol). Column chromatography (hexane/EtOAc/AcOH 80:20:1) afforded the title product as a light brown solid (73.1 mg, 99%).

^{1}H NMR (500 MHz, $CDCl_3$) δ 11.33 (br s, 1H), 7.44 (td, *J* = 8.0, 5.8 Hz, 1H), 7.35 (d, *J* = 8.7 Hz, 2H), 7.18 (d, *J* = 7.6 Hz, 1H), 7.10 (app t, *J* = 8.8 Hz, 1H), 6.94 (d, *J* = 8.7 Hz, 2H), 3.84 (s, 3H); ^{13}C NMR (125.8 MHz, $CDCl_3$) δ 171.4, 159.9 (d, *J* = 251.8 Hz), 159.7, 142.4 (d, *J* = 2.2 Hz), 131.7 (d, *J* = 9.1 Hz), 131.5 (d, *J* = 2.1 Hz), 129.6, 125.8 (d, *J* = 2.9 Hz), 120.6 (d, *J* = 15.7 Hz), 114.3 (d, *J* = 21.6 Hz), 114.2, 55.4; ^{19}F (471 MHz, $CDCl_3$) δ –114.44 (dd, *J* = 9.2, 5.8 Hz, 1F); m.p. 98-100 °C; IR (ATR) 2929, 1700, 1236, 836; HRMS (ESI) *m/z* calcd. $C_{14}H_{10}FO_3$: $[M–H]^-$ 245.0608; found: $[M–H]^-$ 245.0605.

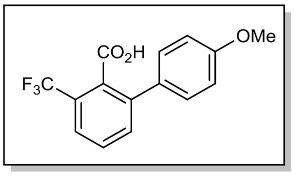

2-(4-methoxyphenyl)-6-(trifluoromethyl)benzoic acid (**3da**)

The General Procedure **A** was applied with 2-(trifluoromethyl)benzoic acid (57.0 mg, 0.30 mmol) and 4-iodoanisole (140.4 mg, 2.0 equiv, 0.60 mmol). Column chromatography (hexane/EtOAc/AcOH 80:20:1) afforded the title product as a white solid (81.8 mg, 92%).

^{1}H NMR (500 MHz, $CDCl_3$) δ 7.68 (d, *J* = 7.2 Hz, 1H), 7.59 - 7.55 (m, 2H), 7.33 (d, *J* = 8.2 Hz, 2H), 6.94 (d, *J* = 8.2 Hz, 2H), 3.85 (s, 3H); ^{13}C NMR (125.8 MHz, $CDCl_3$) δ 172.9, 159.8, 141.0, 133.9, 131.3, 130.4, 129.9, 129.8, 127.9 (q, *J* = 32.0 Hz), 124.8

(q, J = 4.6 Hz), 123.6 (q, J = 273.7 Hz), 114.2, 55.4; ^{19}F NMR (471 MHz, $CDCl_3$) δ –59.28 (s, 3F); m.p 154-156 °C; IR (ATR) 2982, 1706, 1290, 1117; HRMS (ESI) *m/z* calcd. $C_{15}H_{11}F_3NaO_3$: $[M+Na]^+$ 319.0553; found: $[M+Na]^+$ 319.0555.

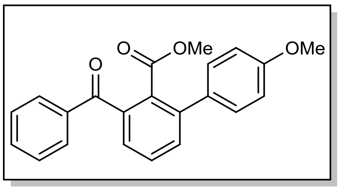

methyl 3-benzoyl-4'-methoxy-[1,1'-biphenyl]-2-carboxylate (**3ea**)

The General Procedure **A** was applied with 2-benzoylbenzoic acid (67.9 mg, 0.30 mmol) and 4-iodoanisole (140.4 mg, 2.0 equiv, 0.60 mmol) Column chromatography (0-20% EtOAc-hexane), after derivatization with MeI, afforded the title product as a white solid (87.3 mg, 84%).

Spectroscopic data matched those previously reported.[10]

^1H NMR (500 MHz, $(CD_3)_2CO$) δ 7.81 (d, J = 8.1 Hz, 2H), 7.70 - 7.64 (m, 3H), 7.56 (app t, J = 7.7 Hz, 2H), 7.52 (dd, J = 6.7, 2.0 Hz, 1H), 7.32 (d, J = 8.6 Hz, 2H), 7.01 (d, J = 8.6 Hz, 2H), 3.85 (s, 3H), 3.38 (s, 3H); ^{13}C NMR (126 MHz, $(CD_3)_2CO$) δ 196.6, 169.2, 160.5, 141.8, 139.7, 137.9, 134.1, 133.5, 133.4, 133.0, 130.7, 130.3, 130.3, 129.4, 128.4, 114.8, 55.6, 52.2.

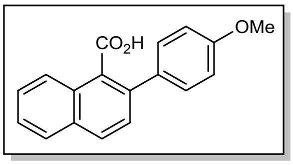

2-(4-methoxyphenyl)naphthalene-1-carboxylic acid (**3fa**)

The General Procedure **A** was applied with 2-benzoylbenzoic acid (51.7 mg, 0.30 mmol) and 4-iodoanisole (140.4 mg, 2.0 equiv, 0.60 mmol). Column chromatography (hexane/EtOAc/AcOH 80:20:1) afforded the title product as a white solid (78.5 mg, 94%).

^1H NMR (500 MHz, $CDCl_3$) δ 8.05 (d, J = 8.4 Hz, 1H), 7.96 (d, J = 8.5 Hz, 1H), 7.90 (d, J = 8.1 Hz, 1H), 7.59 (t, J = 7.5 Hz, 1H), 7.55 - 7.48 (m, 4H), 6.98 (d, J = 8.4 Hz, 2H), 3.84 (s, 3H); ^{13}C NMR (126 MHz, $CDCl_3$) δ 159.5, 154.8, 137.8, 133.2, 132.2, 130.3, 130.0, 129.9, 128.3, 127.9, 127.7, 126.6, 126.4, 125.1, 114.2, 55.4; m.p. 174-176 °C; IR (ATR) 3235, 1691, 1241, 1464, 1017; HRMS (ESI) *m/z* calcd. $C_{18}H_{13}O_3$: $[M–H]^–$ 277.0865; found: $[M–H]^–$ 277.0854.

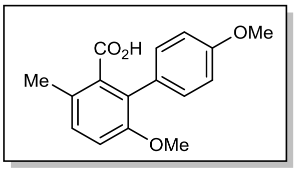

3-methoxy-2-(4-methoxyphenyl)-6-methylbenzoic acid (**3ga**)

The General Procedure **A** was applied with 2-methoxy-5-methylbenzoic acid (49.9 mg, 0.30 mmol) and 4-iodoanisole (140.4 mg, 2.0 equiv, 0.60 mmol). Column chromatography (hexane/EtOAc/AcOH 80:20:1) afforded the title product as a light brown solid (71.9 mg, 88%).

^{1}H NMR (500 MHz, $CDCl_3$) δ 7.25 (app d, J = 8.2 Hz, 2H), 7.14 (d, J = 8.4 Hz, 1H), 6.90 (app t, J = 7.5 Hz, 3H), 3.81 (s, 3H), 3.71 (s, 3H), 2.31 (s, 3H); ^{13}C NMR (125.8 MHz, $CDCl_3$) δ 174.4, 159.0, 154.9, 134.8, 131.1, 130.1, 128.3, 128.2, 126.6, 113.5, 112.5, 56.1, 55.2, 19.0; m.p. >150 °C decomposition; IR (ATR) 2917, 1689, 1327,1293; HRMS (ESI) m/z calcd. $C_{16}H_{16}O_4$: $[M–H]^–$ 271.0965; found: $[M–H]^–$ 271.0962.

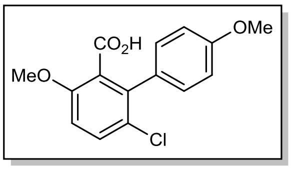

6-chloro-3,4'-dimethoxy-[1,1'-biphenyl]-2-carboxylic acid (**3ha**)

The General Procedure **A** was applied with 2-methoxy-5-chlorobenzoic acid (56.0 mg, 0.30 mmol) and 4-iodoanisole (140.4 mg, 2.0 equiv, 0.60 mmol) with $[Ru(^tBuCN)_6](BF_4)_2$ (13.9 mg, 6 mol %). Column chromatography (hexane/EtOAc/AcOH 80:20:1) afforded the title product as a light brown solid (70.2 mg, 80%).

^{1}H NMR (400 MHz, $CDCl_3$) δ 7.45 (d, J = 8.9 Hz, 1H), 7.23 (d, J = 8.7 Hz, 2H), 6.92 (d, J = 8.7 Hz, 2H), 6.88 (d, J = 9.0 Hz, 1H), 3.86 (s, 3H), 3.84 (s, 3H); ^{13}C NMR (101 MHz, $CDCl_3$) δ 171.1, 159.4, 154.7, 138.7, 131.2, 130.7, 128.6, 125.6, 125.2, 113.5, 111.3, 56.3, 55.2; m.p. 140-144 °C; IR (ATR) 3069, 3004, 2945, 2839, 1704, 1515, 1460, 1286, 826; HRMS (ESI) m/z calcd. $C_{15}H_{14}ClO_4$: $[M+H]^+$ 293.0575; found: $[M+H]^+$ 293.0560.

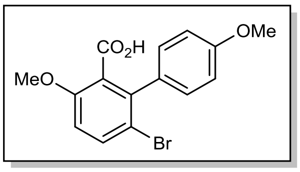

6-bromo-3,4'-dimethoxy-[1,1'-biphenyl]-2-carboxylic acid (**3ia**)

The General Procedure **A** was applied with 2-methoxy-5-bromobenzoic acid (69.3 mg, 0.30 mmol) and 4-iodoanisole (140.4 mg, 2.0 equiv, 0.60 mmol) with $[Ru(^tBuCN)_6](BF_4)_2$ (13.9 mg, 6 mol %).

Column chromatography (hexane/EtOAc/AcOH 80:20:1) afforded the title product as a light brown solid (42.5 mg, 42%).

^{1}H NMR (400 MHz, $CDCl_3$) δ 7.64 (d, *J* = 8.9 Hz, 1H), 7.21 (d, *J* = 8.7 Hz, 2H), 6.92 (d, *J* = 8.7 Hz, 2H), 6.82 (d, *J* = 9.0 Hz, 1H), 3.86 (s, 3H), 3.84 (s, 3H); ^{13}C NMR (101 MHz, $CDCl_3$) δ 171.1, 159.4, 155.3, 140.5, 134.4, 130.6, 130.5, 125.3, 115.2, 113.4, 111.6, 56.3, 55.2; m.p. 150-152 °C; IR (ATR) 3013, 2933, 2897, 1704, 1514, 1285, 809; HRMS (ESI) *m/z* calcd. $C_{15}H_{14}BrO_4$: $[M+H]^+$ 337.0070; found: $[M+H]^+$ 337.0054.

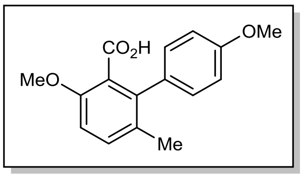

3,4'-dimethoxy-6-methyl-[1,1'-biphenyl]-2-carboxylic acid (**3ja**)

The General Procedure **A** was applied with 2-methoxy-5-methylbenzoic acid (49.9 mg, 0.30 mmol) and 4-iodoanisole (140.4 mg, 2.0 equiv, 0.60 mmol) with $[Ru(^{t}BuCN)_6](BF_4)_2$ (13.9 mg, 6 mol %). Column chromatography (hexane/EtOAc/AcOH 80:20:1) afforded the title product as a light brown solid (17.2 mg, 21%).

^{1}H NMR (400 MHz, $CDCl_3$) δ 7.24 (d, *J* = 8.5 Hz, 1H), 7.15 (d, *J* = 8.7 Hz, 2H), 6.90 (d, *J* = 8.7 Hz, 2H), 6.84 (d, *J* = 8.5 Hz, 1H), 3.85 (s, 3H), 3.83 (s, 3H), 2.04 (s, 3H); ^{13}C NMR (101 MHz, $CDCl_3$) δ 170.9, 158.9, 154.2, 140.2, 131.9, 131.0, 130.4, 129.1, 123.8, 113.6, 110.1, 56.2, 55.3, 19.8; m.p. 138-140 °C; IR (ATR) 3010, 2933, 2836, 1710, 1515, 1290, 1176, 814; HRMS (ESI) *m/z* calcd.$C_{16}H_{17}O_4$: $[M+H]^+$ 273.1121; found: $[M+H]^+$ 273.1117.

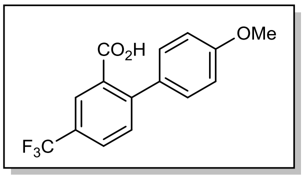

2-(4-methoxyphenyl)-5-(trifluoromethyl)benzoic acid (**3ka**)

The General Procedure **A** was applied with 3-(trifluoromethyl)benzoic acid (57.0 mg, 0.3 mmol) and 4-iodoanisole (140.4 mg, 0.60 mmol). Column chromatography (hexane/EtOAc/AcOH 80:20:1) afforded the title product as a white solid (85.3 mg, 96%).

^{1}H NMR (500 MHz, $CDCl_3$) δ 11.04 (br s, 1H,), 8.10 (s, 1H), 7.69 (d, *J* = 8.0 Hz, 1H), 7.40 (d, *J* = 8.0 Hz, 1H), 7.17 (d, *J* = 8.1 Hz, 2H), 6.85 (d, *J* = 8.2 Hz, 2H), 3.75 (s, 3H); ^{13}C NMR (125.8 MHz, $CDCl_3$) δ 173.0, 159.8, 146.6, 132.0, 131.9, 130.0, 129.7,

129.4 (q, J = 33.6 Hz), 128.7 (q, J = 3.2 Hz), 127.9 (q, J = 3.7 Hz), 123.9 (q, J = 272.2 Hz), 114.0, 55.4; ^{19}F (471 MHz, $CDCl_3$) δ –62.59 (s, 3F); m.p. 118-120 °C; IR (ATR) 1693, 1331, 1248, 1122; HRMS (ESI) *m/z* calcd. $C_{15}H_{10}F_3O_3$: $[M–H]^-$ 295.0577; found: $[M–H]^-$ 295.0570.

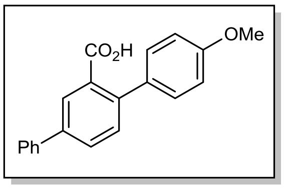

4-methoxy-[1,1':4',1''-terphenyl]-2'-carboxylic acid (**3la**)

The General Procedure **A** was applied with [1,1'-biphenyl]-3-carboxylic acid (59.5 mg, 0.30 mmol) and 4-iodoanisole (140.4 mg, 0.60 mmol). Column chromatography (hexane/EtOAc/AcOH 80:20:1) afforded the title product as a white solid (83.1 mg, 91%).

^{1}H NMR (400 MHz, $CDCl_3$) δ 8.18 (d, J = 2.0 Hz, 1H), 7.78 (dd, J = 8.0, 2.0 Hz, 1H), 7.67 - 7.64 (m, 2H), 7.50 - 7.37 (m, 4H), 7.35 - 7.31 (m, 2H), 6.97 - 6.94 (m, 2H), 3.85 (s, 3H); ^{13}C NMR (101 MHz, $CDCl_3$) δ 173.7, 159.3, 141.8, 139.9, 139.7, 133.1, 131.9, 130.6, 129.9, 129.8, 129.4, 129.1, 127.9, 127.2, 113.8, 55.4; m.p. 184-186 °C; IR (ATR) 3066, 2939, 2553, 1692, 1241, 965; HRMS (ESI) *m/z* calcd. $C_{20}H_{15}O_3$: $[M–H]^-$ 303.1021; found: $[M–H]^-$ 303.1015.

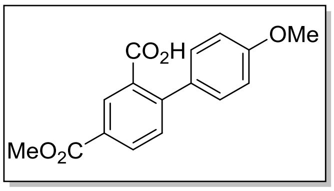

4'-methoxy-4-(methoxycarbonyl)-[1,1'-biphenyl]-2-carboxylic acid (**3ma**)

The General Procedure **A** was applied with 3-(methoxycarbonyl)benzoic acid (54.1 mg, 0.30 mmol) and 4-iodoanisole (140.4 mg, 0.60 mmol) for 3 h. Column chromatography (hexane/EtOAc/AcOH 80:20:1) afforded the title product as a white solid (73.0 mg, 85%).

^{1}H NMR (400 MHz, $CDCl_3$) δ 8.58 (d, J = 1.7 Hz, 1H), 8.18 (dd, J = 8.0, 1.7 Hz, 1H), 7.45 (d, J = 8.1 Hz, 1H), 7.29 (d, J = 8.6 Hz, 2H), 6.94 (d, J = 8.7 Hz, 2H), 3.96 (s, 3H), 3.85 (s, 3H); ^{13}C NMR (101 MHz, $CDCl_3$) δ 172.9, 166.3, 159.7, 147.3, 132.9, 132.3, 132.1, 131.5, 129.8, 129.8, 128.8, 113.9, 55.4, 52.5; m.p. 150-156 °C, IR (ATR) 3219, 3010, 2836, 1919, 1696, 1253, 1178, 830, 732; HRMS (ESI) *m/z* calcd. $C_{16}H_{14}O_5$: $[M–H]^-$ 287.0914; found: $[M–H]^-$: 287.0910.

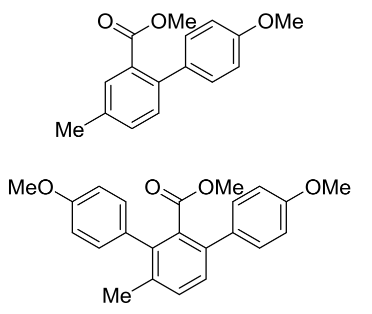

methyl 4'-methoxy-4-methyl-[1,1'-biphenyl]-2-carboxylate (**3na**) and methyl 4,4''-dimethoxy-4'-methyl-[1,1':3',1''-terphenyl]-2'-carboxylate (**3na'**)

The General Procedure **A** was applied with 3-methylbenzoic acid (40.9 mg, 0.30 mmol) and 4-iodoanisole (140.4 mg, 0.60 mmol). Column chromatography (EtOAc-hexane 0-20%), after derivatization with MeI, afforded **3na** (63.0 mg, 82%) and **3na'** (10.9 mg, 10) as white solids.

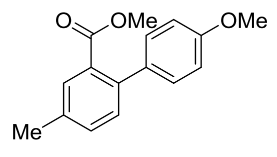

methyl 4'-methoxy-4-methyl-[1,1'-biphenyl]-2-carboxylate (**3na**)

Spectroscopic data matched those previously reported.[10]

^{1}H NMR (400 MHz, $CDCl_3$) δ 7.59 (s, 1H), 7.32 - 7.29 (m, 1H), 7.25 - 7.21 (m, 3H), 6.92 - 6.90 (m, 2H), 3.83 (s, 3H), 3.65 (s, 3H), 2.40 (s, 3H); ^{13}C NMR (101 MHz, $CDCl_3$) δ 169.7, 158.9, 139.3, 136.8, 133.7, 132.1, 130.8, 130.7, 130.3, 129.6, 113.6, 55.4, 52.1, 21.0.

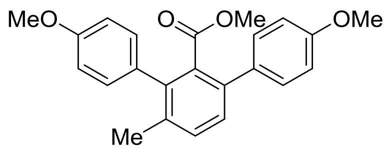

methyl 4,4''-dimethoxy-4'-methyl-[1,1':3',1''-terphenyl]-2'-carboxylate (**3na'**)

^{1}H NMR (400 MHz, $CDCl_3$) δ 7.35 (d, J = 8.2 Hz, 3H), 7.28 (s, 1H), 7.19 (d, J = 8.7 Hz, 2H), 6.95 - 6.92 (m, 4H), 3.86 (s, 3H), 3.85 (s, 3H), 3.32 (s, 3H), 2.16 (s, 3H); ^{13}C NMR (101 MHz, $CDCl_3$) δ 170.2, 159.1, 158.8, 139.2, 136.8, 135.6, 134.3, 133.1, 131.5, 131.0, 130.4, 129.6, 128.6, 113.9, 113.6, 55.4, 55.3, 51.7, 20.5; m.p. 150-154 °C, IR (ATR) 3021, 2915, 1696, 1511, 1223, 839, 787; HRMS (ESI) m/z calcd. $C_{23}H_{22}O_4Na$: $[M+Na]^+$ 385.1410; found: $[M+Na]^+$ 385.1405.

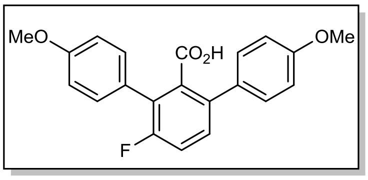

4'-fluoro-4,4''-dimethoxy-[1,1':3',1''-terphenyl]-2'-carboxylic acid (**3oa**)

The General Procedure **B** was applied with 3-fluorobenzoic acid (42.0 mg, 0.30 mmol) and 4-iodoanisole (280.8 mg, 4.0 equiv, 1.20 mmol). Column chromatography (hexane/EtOAc/AcOH 80:20:1) afforded the title product as a white solid (104.7 mg, 99%).

^{1}H NMR (500 MHz, $(CD_3)_2CO$) δ 11.27 (br s, 1H), 7.41 - 7.30 (m, 6H), 6.98 (dd, *J* = 11.4, 8.6 Hz, 4H), 3.83 (d, *J* = 5.2 Hz, 6H); ^{13}C NMR (126 MHz, $(CD_3)_2CO$) δ 169.2, 160.6, 160.3, 159.5 (d, *J* = 244.3 Hz), 137.4, 137.4, 136.1 (d, *J* = 3.9 Hz), 133.0, 131.9, 131.4 (d, *J* = 8.3 Hz), 130.7, 127.6 (d, *J* = 18.1 Hz), 126.4, 116.8 (d, *J* = 23.4 Hz), 114.5 (d, *J* = 25.5 Hz), 55.6, 55.6; m.p. 144-148 °C, IR (ATR) 3068, 2903, 2838, 2367, 2364, 1731, 1700, 1610, 1246, 825; HRMS (ESI) *m/z* calcd. $C_{21}H_{17}FNaO_4$: $[M+Na]^+$ 375.1003; found: $[M+Na]^+$ 375.0999.

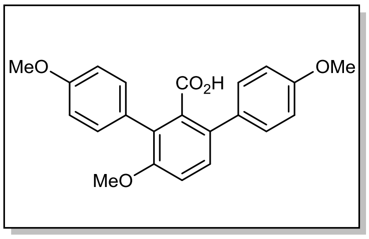

3-methoxy-2,6-bis(4-methoxyphenyl)benzoic acid (**3pa**)

The General Procedure **B** was applied with 3-methoxybenzoic acid (45.6 mg, 0.3 mmol) and 4-iodoanisole (280.8 mg, 4.0 equiv, 1.20 mmol). Column chromatography (hexane/EtOAc/AcOH 80:20:1) afforded the title product as a white solid (98.4 mg, 90%).

^{1}H NMR (500 MHz, $CDCl_3$) 7.28 - 7.22 (m, 5H), 7.02 - 7.01 (m, 1H), 6.87 - 6.85 (m, 4H), 3.79 (s, 6H), 3.74 (s, 3H); ^{13}C NMR (126 MHz, $CDCl_3$) δ 173.6, 159.0 (2C), 155.8, 134.2, 132.7, 131.8, 131.1, 130.1, 129.7, 128.5, 127.9, 113.8, 113.5, 112.2, 56.1, 55.3, 55.2; m.p. 158-160 °C; IR (ATR) 1693, 1514, 1249, 1177, 1121, 827; HRMS (ESI) *m/z* calcd. $C_{22}H_{21}O_5$: $[M+H]^+$ 365.1384; found: $[M+H]^+$ 365.1373.

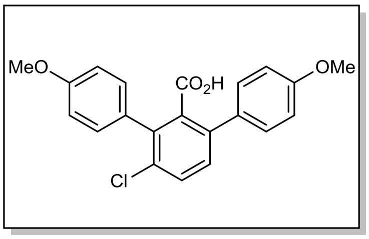

4'-chloro-4,4''-dimethoxy-[1,1':3',1''-terphenyl]-2'-carboxylic acid (**3qa**)

The General Procedure **B** was applied with 3-chlorobenzoic acid (47.0 mg, 0.30 mmol) and 4-iodoanisole (280.8 mg, 4.0 equiv, 1.20 mmol). Column chromatography (hexane/EtOAc/AcOH 80:20:1) afforded the title product as a white solid (104.0 mg, 94%).

^{1}H NMR (400 MHz, $CDCl_3$) δ 7.55 (d, J = 8.3 Hz, 1H), 7.34 (d, J = 8.7 Hz, 2H), 7.29 - 7.25 (m, 3H), 6.95 - 6.91 (m, 4H), 3.85 (s, 3H), 3.84 (s, 3H); ^{13}C NMR (101 MHz, $CDCl_3$) δ 170.1, 159.6, 159.5, 138.2, 138.1, 134.6, 133.2, 131.8, 130.9, 130.7, 130.2, 129.7, 129.2, 114.1, 113.6, 55.4, 55.3; m.p. 136-140 °C, IR (ATR) 3052, 2930, 1750, 1703, 1246, 825, 710; HRMS (ESI) m/z calcd. $C_{21}H_{16}ClO_4$: $[M–H]^-$ 367.0737; found: $[M–H]^-$ 367.0735.

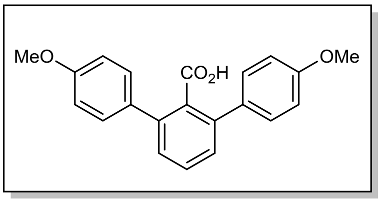

2,6-bis(4-methoxyphenyl)benzoic acid (**3ra**)

The General Procedure **B** was applied with benzoic acid (36.6 mg, 0.30 mmol) and 4-iodoanisole (280.8 mg, 4.0 equiv, 1.20 mmol). Column chromatography (hexane/EtOAc/AcOH 80:20:1) afforded the title product as a white solid (97.3 mg, 97%).

^{1}H NMR (400 MHz, $CDCl_3$) δ 7.48 (dd, J = 8.1, 7.3 Hz, 1H), 7.37 - 7.32 (m, 6H), 6.93 - 6.90 (m, 4H), 3.84 (s, 6H); ^{13}C NMR (101 MHz, $CDCl_3$) δ 174.1, 159.3, 140.0, 132.9, 131.7, 129.7, 129.7, 128.8, 113.9, 55.4; m.p. 142-144 °C; IR (ATR) 1695, 1514, 1248, 1179; HRMS (ESI) m/z calcd. $C_{21}H_{17}O_4$: $[M–H]^-$ 333.1132; found: $[M–H]^-$ 333.1125.

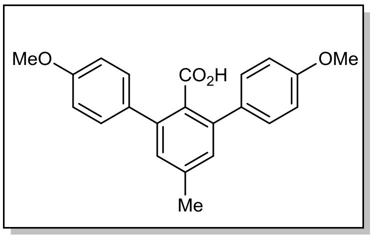

2,6-bis(4-methoxyphenyl)-4-methylbenzoic acid (**3sa**)

The General Procedure **B** was applied with 4-methylbenzoic acid (40.9 mg, 0.30 mmol) and 4-iodoanisole (280.8 mg, 4.0 equiv, 1.20 mmol). Column

chromatography (hexane/EtOAc/AcOH 80:20:1) afforded the title product as a white solid (88.8 mg, 85%).

^{1}H NMR (500 MHz, $CDCl_3$) δ 7.33 (d, J = 8.5 Hz, 4H), 7.13 (s, 2H), 6.90 (d, J = 8.5 Hz, 4H), 3.83 (s, 6H), 2.42 (s, 3H); ^{13}C NMR (126 MHz, $CDCl_3$) δ 174.2, 159.3, 140.1, 139.6, 133.1, 129.7, 129.6, 129.2, 113.9, 55.4, 21.4; m.p. 198-200 °C; IR (ATR) 3215, 2960, 1690, 1512, 1245; HRMS (ESI) *m/z* calcd. $C_{22}H_{19}O_4$: $[M–H]^–$ 347.1283; found: $[M–H]^–$ 347.1268.

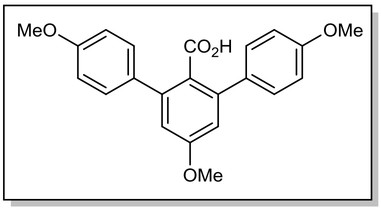

4,4'',5'-trimethoxy-[1,1':3',1''-terphenyl]-2'-carboxylic acid (**3ta**)

The General Procedure **B** was applied with 4-methoxybenzoic acid (45.6 mg, 0.30 mmol) and 4-iodoanisole (280.8 mg, 4.0 equiv, 1.20 mmol) with $[Ru(^{t}BuCN)_6](BF_4)_2$ (23.2 mg, 10 mol %). Column chromatography (hexane/EtOAc/AcOH 80:20:1) afforded the title product as a white solid (91.8 mg, 84%).

^{1}H NMR (500 MHz, $(CD_3)CO$) δ 7.41 (app d, J = 8.6 Hz, 4H), 6.96 (app d, J = 8.6 Hz, 4H), 6.86 (s, 2H), 3.90 (s, 3H), 3.83 (s, 6H); ^{13}C NMR (126 MHz, $(CD_3)CO$) δ 170.8, 160.3, 160.3, 142.1, 134.1, 130.5, 127.6, 114.7, 114.4, 55.8, 55.6; m.p. 190-194 °C; IR (ATR) 3013, 2895, 2830, 2155, 1692, 1616, 1586, 1253, 1030, 823; HRMS (ESI) *m/z* calcd. $C_{22}H_{21}O_5$: $[M+H]^+$ 365.1384; found: $[M+H]^+$ 365.1382.

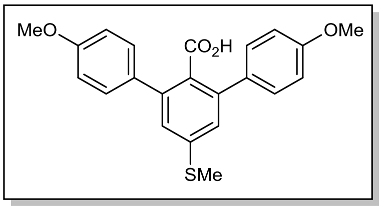

4,4''-dimethoxy-5'-(methylthio)-[1,1':3',1''-terphenyl]-2'-carboxylic acid (**3ua**).

The General Procedure **B** was applied with 4-(methylthio)benzoic acid (50.5 mg, 0.30 mmol) and 4-iodoanisole (280.8 mg, 4.0 equiv, 1.20 mmol). Column chromatography (hexane/EtOAc/AcOH 80:20:1) afforded the title product as a white solid (89.0 mg, 78%).

^{1}H NMR (400 MHz, $(CD_3)CO$) δ 7.42 - 7.40 (m, 4H), 7.17 (s, 2H), 6.98 - 6.96 (m, 4H), 3.83 (s, 6H), 2.57 (s, 3H); ^{13}C NMR (101 MHz, $(CD_3)CO$) δ 170.5, 160.4, 140.9, 140.9, 133.6, 131.4, 130.6, 126.2, 114.5, 55.6, 15.0; m.p. 118-122 °C; IR (ATR)

3039, 3001, 2924, 2842, 2353, 1694, 1509, 1242, 831; HRMS (ESI) *m/z* calcd. $C_{22}H_{21}O_4S$: $[M+H]^+$ 381.1155; found: $[M+H]^+$ 381.1152.

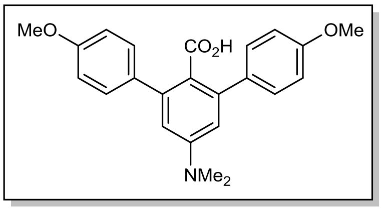

5'-(dimethylamino)-4,4''-dimethoxy-[1,1':3',1''-terphenyl]-2'-carboxylic acid (**3va**)

The General Procedure **B** was applied with 4-(dimethylamino)benzoic acid (49.6 mg, 0.30 mmol) and 4-iodoanisole (280.8 mg, 4.0 equiv, 1.20 mmol) with $[Ru(^tBuCN)_6](BF_4)_2$ (23.2 mg, 10 mol %) for 1 h. Column chromatography (hexane/EtOAc/AcOH 80:20:1) afforded the title product as a brown solid (95.1 mg, 84%).

^{1}H NMR (500 MHz, $(CD_3)CO$) δ 10.59 (br s, 1H), 7.40 - 7.39 (m, 4H), 6.95 - 6.93 (m, 4H), 6.61 (s, 2H), 3.82 (s, 6H), 3.03 (s, 6H); ^{13}C NMR (126 MHz, $(CD_3)CO$) δ 171.4, 160.0, 151.2, 141.8, 135.3, 130.5, 122.9, 114.3, 112.9, 55.5, 40.4; m.p. >170 °C; IR (ATR) 3007, 2965, 2815, 2348, 1684, 1598, 1502, 1248, 1174, 826; HRMS (ESI) *m/z* calcd. $C_{23}H_{24}O_4N$: $[M+H]^+$ 378.1700; found: $[M+H]^+$ 378.1697.

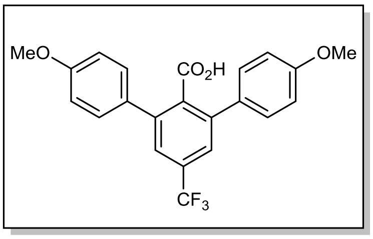

2,6-bis(4-methoxyphenyl)-4-(trifluoromethyl)benzoic acid (**3wa**).

The General Procedure **B** was applied with 4-(trifluoromethyl)benzoic acid (57.0 mg, 0.30 mmol) and 4-iodoanisole (280.8 mg, 4.0 equiv, 1.20 mmol). Column chromatography (hexane/EtOAc/AcOH 80:20:1) afforded the title product as a white solid (119.5 mg, 99%).

^{1}H NMR (500 MHz, $CDCl_3$) δ 9.66 (br s, 1H), 7.58 (s, 2H), 7.34 (app d, J = 8.0 Hz, 4H), 6.92 (app d, J = 8.1 Hz, 4H), 3.84 (s, 6H); ^{13}C NMR (126 MHz, $CDCl_3$) δ 173.5, 159.8, 140.8, 135.1, 131.9 (q, J = 32.6 Hz), 131.5, 129.8, 125.5 (q, J = 3.6 Hz), 123.8 (q, J = 272.9 Hz), 114.1, 55.4; ^{19}F (376 MHz, $CDCl_3$) δ –62.78 (s, 3F); m.p. 172-174 °C; IR (ATR) 3021, 2983, 1691, 1515, 1253, 1079, 748; HRMS (ESI) m/z calcd. $C_{22}H_{18}F_3O_4$: $[M+H]^+$ 403.1152; found: $[M+H]^+$ 403.1135.

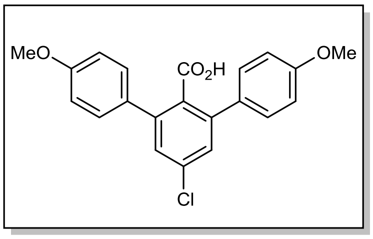

5'-chloro-4,4''-dimethoxy-[1,1':3',1''-terphenyl]-2'-carboxylic acid (**3xa**)

The General Procedure **B** was applied with 4-chlorobenzoic acid (47.0 mg, 0.30 mmol) and 4-iodoanisole (280.8 mg, 4.0 equiv, 1.20 mmol). Column chromatography (hexane/EtOAc/AcOH 80:20:1) afforded the title product as a white solid (97.4 mg, 88%).

^{1}H NMR (400 MHz, $(CD_3)CO$) δ 7.44 - 7.41 (m, 4H), 7.36 (s, 2H), 7.00 – 6.98 (m, 4H), 3.84 (s, 6H); ^{13}C NMR (101 MHz, $(CD_3)CO$) δ 169.9, 160.6, 142.2, 134.7, 133.6, 132.5, 130.6, 128.9, 114.6, 55.6; m.p. 96-98 °C; IR (ATR) 3060, 3027, 2968, 2904, 2364, 2329, 1698, 1608, 1511, 1247, 831; HRMS (ESI) *m/z* calcd. $C_{21}H_{17}ClNaO_4$: $[M+Na]^+$ 391.0708; found: $[M+Na]^+$ 391.0704.

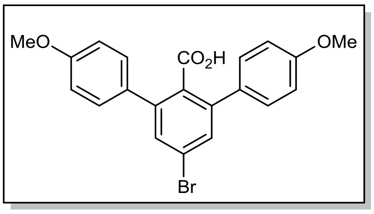

5'-bromo-4,4''-dimethoxy-[1,1':3',1''-terphenyl]-2'-carboxylic acid (**3ya**)

The General Procedure **B** was applied with 4-bromobenzoic acid (60.3 mg, 0.30 mmol) and 4-iodoanisole (280.8 mg, 4.0 equiv, 1.20 mmol) in the absence of H_2O for 12 h. Column chromatography (hexane/EtOAc/AcOH 80:20:1) afforded the title product as a white solid (80.6 mg, 65%).

^{1}H NMR (500 MHz, $(CD_3)CO$) δ 7.50 (s, 2H), 7.42 (d, *J* = 8.6 Hz, 4H), 6.98 (d, *J* = 8.6 Hz, 4H), 3.84 (s, 6H); ^{13}C NMR (126 MHz, $(CD_3)CO$) δ 170.0, 160.6, 142.3, 134.1, 132.4, 131.8, 130.7, 122.9, 114.6, 55.6; m.p. 220-222 °C; IR (ATR) 3036, 295, 2839, 2361, 1698, 1613, 1512, 1248, 1174, 832; HRMS (ESI) *m/z* calcd. $C_{21}H_{17}BrNaO_4$: $[M+Na]^+$ 435.0202; found: $[M+Na]^+$ 435.0200.

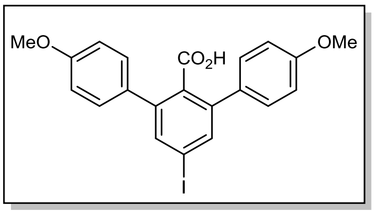

5'-iodo-4,4''-dimethoxy-[1,1':3',1''-terphenyl]-2'-carboxylic acid (**3za**)

The General Procedure **B** was applied with 4-iodobenzoic acid (74.4 mg, 0.30 mmol) and 4-

iodoanisole (280.8 mg, 4.0 equiv, 1.20 mmol) for 4 h. Column chromatography (hexane/EtOAc/AcOH 80:20:1) afforded the title product as a white solid (48.3 mg, 35%).

^{1}H NMR (400 MHz, (CD$_3$)CO) δ 7.71 (s, 2H), 7.41 (d, *J* = 8.7 Hz, 4H), 6.98 (d, *J* = 8.7 Hz, 4H), 3.83 (s, 6H); ^{13}C NMR (101 MHz, (CD$_3$)CO) δ 170.1, 160.6, 142.1, 137.8, 134.6, 132.3, 130.6, 114.6, 95.0, 55.6; m.p. 234-238 °C; IR (ATR) 3057, 2959, 2833, 1510, 1251, 1178, 832; HRMS (ESI) *m/z* calcd. $C_{21}H_{17}INaO_4$: [M+Na]$^+$ 483.0064; found: [M+Na]$^+$ 483.0061.

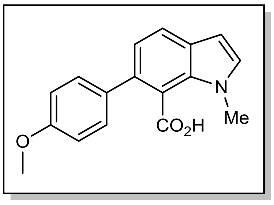

6-(4-methoxyphenyl)-1-methyl-1H-indole-7-carboxylic acid (**5aa**)

The General Procedure **A** was applied with 1-methyl-1H-indole-7-carboxylic acid (52.6 mg, 0.30 mmol) and 4-iodoanisole (140.4 mg, 2.0 equiv, 0.60 mmol). Column chromatography (hexane/EtOAc/AcOH 75:25:1) afforded the title product as a white solid (77.6 mg, 92%).

^{1}H NMR (500 MHz, (CD$_3$)$_2$CO) δ 7.65 (d, *J* = 8.1 Hz, 1H), 7.41 (d, *J* = 8.3 Hz, 2H), 7.27 (s, 1H), 7.03 (d, *J* = 8.1 Hz, 1H), 6.96 (d, *J* = 7.7 Hz, 2H), 6.52 (s, 1H), 3.88 (s, 3H), 3.83 (s, 3H); ^{13}C NMR (126 MHz, (CD$_3$)$_2$CO) δ 170.8, 159.8, 134.9, 133.7, 132.9, 132.7, 131.0, 130.0, 122.3, 122.2, 118.8, 114.3, 101.5, 55.5, 35.0; m.p. 176-178 °C; IR (ATR) 3044, 2966, 2047, 1684, 1329, 1112, 854; HRMS (ESI) *m/z* calcd. $C_{17}H_{16}NO_3$: [M+H]$^+$ 282.1125; found: [M+H]$^+$ 282.1117.

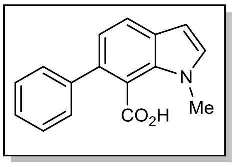

1-methyl-6-phenyl-1H-indole-7-carboxylic acid (**5ae**)

The General Procedure **A** was applied with 1-methyl-1H-indole-7-carboxylic acid (52.6 mg, 0.30 mmol) and iodobenzene (66.9 µL, 2.0 equiv, 0.60 mmol). Column chromatography (hexane/EtOAc/AcOH 75:25:1) afforded the title product as a white solid (67.9 mg, 90%).

^{1}H NMR (500 MHz, (CD$_3$)$_2$CO) δ 11.57 (br s, 1H), 7.68 (d, *J* = 8.1 Hz, 1H), 7.49 (app d, *J* = 7.5 Hz, 2H), 7.40 (app t, *J* = 7.5 Hz, 2H), 7.34 (app t, *J* = 7.3 Hz, 1H), 7.30 (d,

J = 3.1 Hz, 1H), 7.05 (d, J = 8.1 Hz, 1H), 6.54 (d, J = 3.1 Hz, 1H), 3.89 (s, 3H); ^{13}C NMR (126 MHz, $(CD_3)_2CO$) δ 170.6, 142.7, 134.0, 132.9, 132.9, 130.3, 130.0, 128.9, 127.7, 122.4, 122.1, 118.7, 101.5, 35.1; m.p. 120-122 °C; IR (ATR) 3054, 2965, 1698, 1301, 1012, 750; HRMS (ESI) *m/z* calcd. $C_{16}H_{14}NO_2$: $[M+H]^+$ 252.1019; found: $[M+H]^+$ 252.1015.

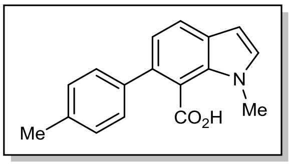

1-methyl-6-(p-tolyl)-1H-indole-7-carboxylic acid (**5af**)

The General Procedure **A** was applied with 1-methyl-1H-indole-7-carboxylic acid (52.6 mg, 0.30 mmol) and 1-iodo-4-methylbenzene (130.8 mg, 2.0 equiv, 0.60 mmol). Column chromatography (hexane/EtOAc/AcOH 75:25:1) afforded the title product as a white solid (71.6 mg, 90%).

^1H NMR (500 MHz, $(CD_3)_2CO$) δ 11.54 (br s, 1H), 7.66 (d, J = 8.1 Hz, 1H), 7.39 (d, J = 7.6 Hz, 2H), 7.28 (s, 1H), 7.21 (d, J = 7.6 Hz, 2H), 7.04 (d, J = 8.1 Hz, 1H), 6.52 (d, J = 1.7 Hz, 1H), 3.88 (s, 3H), 2.36 (s, 3H); ^{13}C NMR (126 MHz, $(CD_3)_2CO$) δ 170.7, 139.8, 137.2, 134.0, 132.9, 132.8, 130.2, 129.9, 129.6, 122.3, 122.1, 118.7, 101.5, 35.1, 21.1; m.p. 236-240 °C, IR (ATR) 3060, 2992, 1692, 1507, 1442, 814, 685; HRMS (ESI) *m/z* calcd. $C_{17}H_{16}NO_2$: $[M+H]^+$ 266.1176; found: $[M+H]^+$ 266.1185.

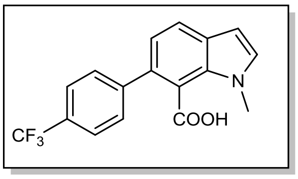

1-methyl-6-(4-(trifluoromethyl)phenyl)-1H-indole-7-carboxylic acid (**5ao**)

The General Procedure **A** was applied with 1-methyl-1H-indole-7-carboxylic acid (52.6 mg, 0.30 mmol) and 1-iodo-4-(trifluoromethyl)benzene (88.2 µL, 2.0 equiv, 0.60 mmol) for 8 h. Column chromatography (hexane/EtOAc/AcOH 75:25:1) afforded the title product as a orange solid (78.5 mg, 82%).

^1H NMR (500 MHz, $(CD_3)_2CO$) δ 7.77 - 7.69 (m, 5H), 7.35 - 7.33 (m, 1H), 7.09 (d, J = 8.1 Hz, 1H), 6.57 (d, J = 3.1 Hz, 1H), 3.90 (s, 3H); ^{13}C NMR (126 MHz, $(CD_3)_2CO$) δ 170.3, 146.9, 133.5, 132.8, 132.5, 131.0, 130.7, 129.3 (q, J = 32.2 Hz), 125.8 (q, J = 3.8 Hz), 125.6 (q, J = 271.2 Hz), 122.7, 121.7, 118.7, 101.7, 35.2; ^{19}F NMR (376 MHz, $(CD_3)_2CO$) δ –62.77 (s, 3F); m.p. 204-210 °C; IR (ATR) 3054, 2945, 2140,

1684, 1324, 1158, 1118, 820; HRMS (ESI) *m/z* calcd. $C_{17}H_{11}F_3NO_2$: $[M–H]^-$ 318.0747; found: $[M–H]^-$ 318.0738.

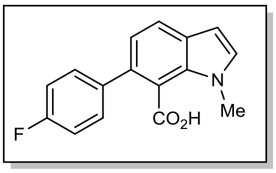

6-(4-fluorophenyl)-1-methyl-1H-indole-7-carboxylic acid (**5ai**)

The General Procedure **A** was applied with 1-methyl-1H-indole-7-carboxylic acid (52.6 mg, 0.30 mmol) and 1-fluoro-4-iodobenzene (69.2 µL, 2.0 equiv, 0.60 mmol). Column chromatography (hexane/EtOAc/AcOH 75:25:1) afforded the title product as a white solid (67.1 mg, 83%).

^{1}H NMR (500 MHz, $(CD_3)_2CO$) δ 11.59 (br s, 1H), 7.68 (d, *J* = 8.1 Hz, 1H), 7.52 - 7.49 (m, 2H), 7.30 (s, 1H), 7.17 (app t, *J* = 8.5 Hz, 2H), 7.04 (d, *J* = 8.1 Hz, 1H), 6.54 (d, *J* = 2.5 Hz, 1H), 3.89 (s, 3H); ^{13}C NMR (126 MHz, $(CD_3)_2CO$) δ 170.6, 163.0 (d, *J* = 243.7 Hz), 138.9 (d, *J* = 3.1 Hz), 133.0, 132.9, 132.8, 131.8 (d, *J* = 8.0 Hz), 130.4, 122.5, 122.0, 118.8, 115.6 (d, *J* = 21.5 Hz), 101.6, 35.1; ^{19}F NMR (376 MHz, $(CD_3)_2CO$) δ –117.71 (tt, *J* = 9.0, 5.5 Hz, 1F); m.p. 174-176 °C; IR (ATR) 3042, 2967, 2113, 1698, 1452, 1136, 846; HRMS (ESI) m/z calcd. $C_{16}H_{13}FNO_2$: $[M+H]^+$ 270.0925; found: $[M+H]^+$ 270.0918.

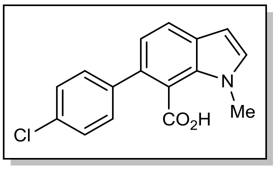

6-(4-chlorophenyl)-1-methyl-1H-indole-7-carboxylic acid (**5aj**)

The General Procedure **A** was applied with 1-methyl-1H-indole-7-carboxylic acid (52.6 mg, 0.30 mmol) and 1-chloro-4-iodobenzene (143.1 mg, 2.0 equiv, 0.60 mmol). Column chromatography (hexane/EtOAc/AcOH 75:25:1) afforded the title product as a white solid (75.4 mg, 88%).

^{1}H NMR (500 MHz, $(CD_3)_2CO$) δ 11.64 (br s, 1H), 7.69 (d, *J* = 8.1 Hz, 1H), 7.49 (d, *J* = 8.1 Hz, 2H), 7.44 (d, *J* = 8.0 Hz, 2H), 7.31 (s, 1H), 7.04 (d, *J* = 8.1 Hz, 1H), 6.54 (d, *J* = 2.0 Hz, 1H), 3.89 (s, 3H); ^{13}C NMR (126 MHz, $(CD_3)_2CO$) δ 170.5, 141.5, 133.4, 133.2, 132.8, 132.6, 131.6, 130.6, 129.0, 122.6, 121.8, 118.7, 101.6, 35.1; m.p. 166-168 °C; IR (ATR) 3021, 2985, 1654, 1325, 1112, 784, 679; HRMS (ESI) m/z calcd. $C_{16}H_{13}ClNO_2$: $[M+H]^+$ 286.0629; found: $[M+H]^+$ 286.0620.

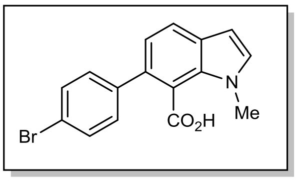

6-(4-bromophenyl)-1-methyl-1H-indole-7-carboxylic acid (**5ak**)

The General Procedure **A** was applied with 1-methyl-1H-indole-7-carboxylic acid (52.6 mg, 0.30 mmol) and 1-bromo-4-iodobenzene (169.7 mg, 2.0 equiv, 0.60 mmol) for 3 h. Column chromatography (hexane/EtOAc/AcOH 75:25:1) afforded the title product as a white solid (76.3 mg, 77%).

^{1}H NMR (500 MHz, $(CD_3)_2CO$) δ 11.57 (br s, 1H), 7.69 (d, *J* = 8.1 Hz, 1H), 7.59 (d, *J* = 7.8 Hz, 2H), 7.42 (d, *J* = 7.2 Hz, 2H), 7.31 (s, 1H), 7.04 (d, *J* = 8.1 Hz, 1H), 6.54 (s, 1H), 3.89 (s, 3H); ^{13}C NMR (126 MHz, $(CD_3)_2CO$) δ 170.5, 142.0, 133.2, 132.8, 132.6, 132.0, 130.6, 122.6 (2C), 121.8, 121.6, 118.7, 101.6, 35.1; m.p. 210-212 °C; IR (ATR) 3084, 2987, 1687, 1324, 1120, 842; HRMS (ESI) m/z calcd. $C_{16}H_{13}BrNO_2$: $[M+H]^+$ 330.0124; found: $[M+H]^+$ 330.0116.

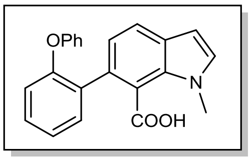

1-methyl-6-(2-phenoxyphenyl)-1H-indole-7-carboxylic acid (**5ad**)

The General Procedure **A** was applied with 1-methyl-1H-indole-7-carboxylic acid (52.6 mg, 0.30 mmol) and 1-iodo-2-phenoxybenzene (177.6 mg, 2.0 equiv, 0.60 mmol) with $[Ru(^tBuCN)_6](BF_4)_2$ (13.9 mg, 6 mol %) for 8 h. Column chromatography (hexane/EtOAc/AcOH 75:25:1) afforded the title product as a white solid (44.3 mg, 43%).

^{1}H NMR (400 MHz, $CDCl_3$) δ 7.66 (d, *J* = 8.1 Hz, 1H), 7.41 (dd, *J* = 7.6, 1.7 Hz, 1H), 7.29 (ddd, *J* = 8.1, 7.6, 1.7 Hz, 1H), 7.23 - 7.18 (m, 2H), 7.14 (td, *J* = 7.5, 1.1 Hz, 1H), 7.08 (d, *J* = 8.1 Hz, 1H), 7.04 (d, *J* = 3.2 Hz, 1H), 7.00 - 6.89 (m, 4H), 6.51 (d, *J* = 3.1 Hz, 1H), 3.78 (s, 3H); ^{13}C NMR (101 MHz, $CDCl_3$) δ 173.1, 157.5, 154.4, 133.3, 132.6, 132.1, 132.0, 130.8, 129.9, 129.6, 128.9, 123.4, 123.0, 122.9, 122.3, 119.0, 118.7, 116.4, 101.4, 35.9; m.p. 174-176 °C; IR (ATR) 3089, 2978, 1700, 1354, 1145, 853; HRMS (ESI) m/z calcd. $C_{22}H_{18}NO_3$: $[M+H]^+$ 344.1287; found $[M+H]^+$: 344.1278.

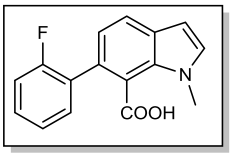

6-(2-fluorophenyl)-1-methyl-1H-indole-7-carboxylic acid (**5am**)

The General Procedure **A** was applied with 1-methyl-1H-indole-7-carboxylic acid (52.6 mg, 0.30 mmol) and 1-fluoro-2-iodobenzene

(70.0 µL, 2.0 equiv, 0.60 mmol) with [Ru(^{t}BuCN)$_6$](BF$_4$)$_2$ (13.9 mg, 6.0 mol %) and H_2O (16.2 µL, 3.0 equiv. 0.90 mmol) for 3 h. Column chromatography (hexane/EtOAc/AcOH 75:25:1) afforded the title product as a light yellow solid (63.0 mg, 78%).

^{1}H NMR (500 MHz, (CD$_3$)$_2$CO) δ 7.69 (d, *J* = 8.1 Hz, 1H), 7.42 - 7.38 (m, 2H), 7.32 (d, *J* = 3.1 Hz, 1H), 7.22 - 7.16 (m, 2H), 7.02 (d, *J* = 8.1 Hz, 1H), 6.55 (d, *J* = 3.1 Hz, 1H), 3.88 (s, 3H); ^{13}C NMR (126 MHz, (CD$_3$)$_2$CO) δ 169.8, 160.9 (d, *J* = 244.7 Hz), 133.2, 133.0, 132.9 (d, *J* = 3.1 Hz), 130.8, 130.1 (d, *J* = 8.0 Hz), 130.0 (d, *J* = 16.4 Hz), 128.0, 124.6 (d, *J* = 3.6 Hz), 122.4 (d, *J* = 0.9 Hz), 122.3, 119.5, 116.1 (d, *J* = 22.6 Hz), 101.7, 35.3; ^{19}F NMR (376 MHz, (CD$_3$)$_2$CO) δ –115.96 (dd, *J* = 5.3, 1.3 Hz); m.p. 198-200 °C; IR (ATR) 3071, 2906, 2559, 2353, 1690, 1522, 1433, 1259, 823, 750; HRMS (ESI) m/z calcd. $C_{16}H_{13}FNO_2$: [M+H]$^+$ 270.0925; found: [M+H]$^+$ 270.0914.

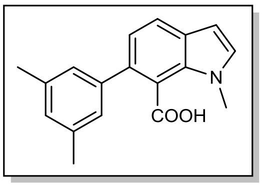

6-(3,5-dimethylphenyl)-1-methyl-1H-indole-7-carboxylic acid (**5aaa**)

The General Procedure **A** was applied with 1-methyl-1H-indole-7-carboxylic acid (52.6 mg, 0.30 mmol) and 1-iodo-3,5-dimethylbenzene (86.6 µL, 2.0 equiv, 0.60 mmol). Column chromatography (hexane/EtOAc/AcOH 75:25:1) afforded the title product as a white solid (77.1 mg, 92%).

^{1}H NMR (500 MHz, (CD$_3$)$_2$CO) δ 11.52 (br s, 1H), 7.65 (d, *J* = 8.1 Hz, 1H), 7.28 (s, 1H), 7.10 (s, 2H), 7.02 (d, *J* = 8.1 Hz, 1H), 6.97 (s, 1H), 6.52 (s, 1H), 3.89 (s, 3H), 2.31 (s, 6H); ^{13}C NMR (126 MHz, (CD$_3$)$_2$CO) δ 170.6, 142.6, 138.1, 134.2, 132.9, 132.8, 130.2, 129.2, 127.8, 122.2, 122.1, 118.6, 101.5, 35.1, 21.4; m.p. 190-192 °C; IR (ATR) 3087, 2954, 1694, 1354, 1289, 820; HRMS (ESI) *m/z* calcd. $C_{18}H_{18}NO_2$: [M+H]$^+$ 280.1332; found: [M+H]$^+$ 280.1324.

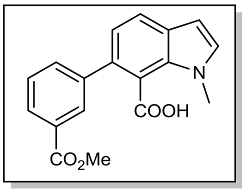

6-(3-(methoxycarbonyl)phenyl)-1-methyl-1H-indole-7-carboxylic acid (**5aba**)

The General Procedure **A** was applied with 1-methyl-1H-indole-

7-carboxylic acid (52.6 mg, 0.30 mmol) and methyl 3-iodobenzoate (157.2 mg, 2.0 equiv, 0.60 mmol) for 3 h. Column chromatography (hexane/EtOAc/AcOH 75:25:1) afforded the title product as a white solid (59.4 mg, 64%).

^{1}H NMR (500 MHz, $(CD_3)_2CO$) δ 8.12 (s, 1H), 7.97 (d, *J* = 7.8 Hz, 1H), 7.74 - 7.71 (m, 2H), 7.55 (app t, *J* = 7.7 Hz, 1H), 7.32 (d, *J* = 3.0 Hz, 1H), 7.09 (d, *J* = 8.1 Hz, 1H), 6.56 (d, *J* = 3.1 Hz, 1H), 3.90 (s, 3H), 2.61 (s, 3H); ^{13}C NMR (126 MHz, $(CD_3)_2CO$) δ 197.9, 170.6, 143.1, 138.0, 134.4, 133.2, 133.0, 132.8, 130.7, 129.9, 129.3, 127.5, 122.6, 121.9, 118.9, 101.6, 35.1, 26.8; m.p. 180-182 °C; IR (ATR) 3057, 2930, 1716, 1675, 1242, 800; HRMS (ESI) *m/z* calcd. $C_{18}H_{15}NO_4Na$: $[M+Na]^+$ 332.0893; found: $[M+Na]^+$ 332.0892.

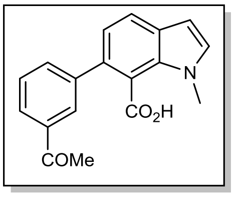

6-(3-acetylphenyl)-1-methyl-1H-indole-7-carboxylic acid (**5ar**)

The General Procedure **A** was applied with 1-methyl-1H-indole-7-carboxylic acid (52.6 mg, 0.30 mmol) and 1-(3-iodophenyl)ethan-1-one (147.6 mg, 2.0 equiv, 0.60 mmol) for 3 h. Column chromatography (hexane/EtOAc/AcOH 75:25:1) afforded the title product as a white solid (44.9 mg, 51%).

^{1}H NMR (500 MHz, $(CD_3)_2CO$) δ 8.15 (s, 1H), 8.00 (d, *J* = 7.8 Hz, 1H), 7.75 - 7.73 (m, 2H), 7.55 (app t, *J* = 7.7 Hz, 1H), 7.33 (s, 1H), 7.07 (d, *J* = 8.1 Hz, 1H), 6.56 (d, *J* = 3.1 Hz, 1H), 3.90 (s, 6H); ^{13}C NMR (126 MHz, $(CD_3)_2CO$) δ 170.4, 167.2, 143.1, 134.5, 133.35, 132.9, 132.8, 131.1, 130.8, 130.7, 129.2, 128.7, 122.6, 121.8, 118.8, 101.6, 52.4, 35.1; m.p. 192-194 °C; IR (ATR) 3060, 2948, 1716, 1298, 1227, 679; HRMS (ESI) *m/z* calcd. $C_{18}H_{16}NO_3$: $[M+H]^+$ 294.1125; found: $[M+H]^+$ 294.1121.

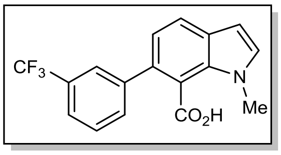

1-methyl-6-(3-(trifluoromethyl)phenyl)-1H-indole-7-carboxylic acid (**5ap**)

The General Procedure **A** was applied with 1-methyl-1H-indole-7-carboxylic acid (52.6 mg, 0.30 mmol) and 1-iodo-3-(trifluoromethyl)benzene (86.5 µL, 2.0 equiv, 0.60 mmol). Column chromatography (hexane/EtOAc/AcOH 75:25:1) afforded the title product as a light pink solid (72.8 mg, 76%).

^{1}H NMR (500 MHz, $(CD_3)_2CO$) δ 7.81 - 7.64 (m, 5H), 7.34 (s, 1H), 7.09 (d, *J* = 8.1 Hz, 1H), 6.57 (d, *J* = 2.2 Hz, 1H), 3.91 (s, 3H); ^{13}C NMR (126 MHz, $(CD_3)_2CO$) δ 169.5, 142.8, 133.0 (d, *J* = 0.9 Hz), 132.6, 131.9, 131.4, 130.0, 129.8 (q, *J* = 31.8 Hz), 129.0, 125.6 (q, *J* = 3.9 Hz), 124.5 (q, *J* = 271.6 Hz), 123.6 (q, *J* = 3.8 Hz), 121.9, 120.9, 117.9, 100.8, 34.3; ^{19}F NMR (376 MHz, $(CD_3)_2CO$) δ –75.94 (s, 3F); m.p. 148-150 °C; IR (ATR) 3001, 2886, 1695, 1516, 1359, 1250, 1129, 809; HRMS (ESI) *m/z* calcd. $C_{17}H_{11}F_3NO_2$: $[M–H]^-$ 318.0747; found: $[M–H]^-$ 318.0734.

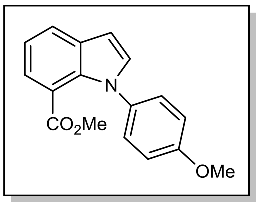

1-(4-methoxyphenyl)-1H-indole-7-carboxylic acid (**5ba**)

The General Procedure **A** was applied with 1H-indole-7-carboxylic acid (48.3 mg, 0.30 mmol) and 4-iodoanisole (140.4 mg, 2.0 equiv, 0.60 mmol) with H_2O (16.2 µL, 3.0 equiv, 0.90 mmol). Column chromatography (0-20% EtOAc/hexane), after derivatization with MeI, afforded the title product as an off-white solid (21.9 mg, 26%).

^{1}H NMR (500 MHz, $(CD_3)_2CO$) δ 7.83 (dd, *J* = 7.9, 1.2 Hz, 1H), 7.47 (dd, *J* = 7.4, 1.1 Hz, 1H), 7.38 (d, *J* = 3.3 Hz, 1H), 7.26 - 7.23 (m, 2H), 7.18 (dd, *J* = 7.8, 7.4 Hz, 1H), 7.08 - 7.05 (m, 2H), 6.75 (d, *J* = 3.3 Hz, 1H), 3.87 (s, 3H), 3.20 (s, 3H); ^{13}C NMR (126 MHz, $(CD_3)_2CO$) δ 168.3, 159.6, 135.7, 134.0, 132.8, 131.9, 126.5, 125.3, 124.8, 120.2, 118.3, 115.3, 104.2, 56.0, 51.7; m.p. 126-130 °C; IR (ATR) 3016, 2948, 1716, 1500, 1436, 1245, 1196, 1137, 746; HRMS (ESI) *m/z* calcd. $C_{17}H_{16}NO_3$: $[M+H]^+$ 282.1125; found: $[M+H]^+$ 282.1113.

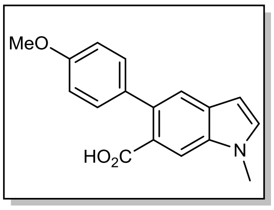

5-(4-methoxyphenyl)-1-methyl-1H-indole-6-carboxylic acid (**5ca**)

The General Procedure **A** was applied with 1-methyl-1*H*-indole-6-carboxylic acid (52.6 mg, 0.30 mmol) and 4-iodoanisole (140.4 mg, 2.0 equiv, 0.60 mmol) with $[Ru(^{t}BuCN)_6](BF_4)_2$ (11.6 mg, 5 mol %). Column chromatography (hexane/EtOAc/AcOH 75:25:1) afforded the title product as a white solid (69.2 mg, 82%).

^{1}H NMR (500 MHz, $(CD_3)_2CO$) δ 7.97 (s, 1H), 7.50 (s, 1H), 7.42 (s, 1H), 7.29 (d, *J* = 8.2 Hz, 2H), 6.92 (d, *J* = 7.1 Hz, 2H), 6.51 (s, 1H), 3.93 (s, 3H), 3.82 (s, 3H); ^{13}C NMR (126 MHz, $(CD_3)_2CO$) δ 170.7, 159.4, 136.5, 136.0, 134.5, 133.4, 131.7, 130.8,

125.4, 123.4, 113.9, 112.9, 101.7, 55.5, 33.1; m.p. 188-192 °C; IR (ATR) 3014, 2954, 2715, 1698, 1423, 1211, 782; HRMS (ESI) *m/z* calcd. $C_{17}H_{16}NO_3$: $[M+H]^+$ 282.1125; found: $[M+H]^+$ 282.1121.

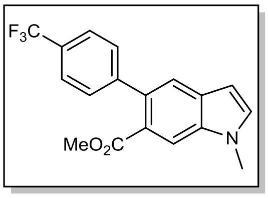

1-methyl-5-(4-(trifluoromethyl)phenyl)-1H-indole-6-carboxylic acid (**5co**)

The General Procedure **A** was applied with 1-methyl-1*H*-indole-6-carboxylic acid (52.6 mg, 0.30 mmol) and 1-iodo-4-(trifluoromethyl)benzene (88.2 µL, 2.0 equiv, 0.60 mmol) with $[Ru(^tBuCN)_6](BF_4)_2$ (11.6 mg, 5.0 mol %) and H_2O (16.2 µL, 3.0 equiv, 0.90 mmol) for 3 h. Column chromatography (0-20% EtOAc/hexane), after derivatization with MeI, afforded the title product as a white solid (64.0 mg, 64%).

^1H NMR (400 MHz, $(CD_3)_2CO$) δ 8.02 (s, 1H), 7.72 (d, *J* = 8.1 Hz, 2H), 7.58 (s, 1H), 7.53 (d, *J* = 8.2 Hz, 2H), 7.51 (d, *J* = 3.1 Hz, 1H), 6.57 (d, *J* = 3.0 Hz, 1H), 3.97 (s, 3H), 3.63 (s, *J* = 4.3 Hz, 3H); ^{13}C NMR (126 MHz, $(CD_3)_2CO$) δ 168.5, 147.5, 135.5, 133.2, 132.4, 130.9, 129.5, 127.6 (q, *J* = 31.9 Hz), 125.0 (d, *J* = 270.7 Hz), 124.5 (q, *J* = 3.9 Hz), 123.6, 122.7, 112.4, 101.2, 51.0, 32.3; ^{19}F NMR (471 MHz, $(CD_3)_2CO$) δ –62.64 (s, 3F); m.p. 152-154 °C; IR (ATR) 3012, 2991, 1713, 1489, 1354, 1261, 809; 715; HRMS (ESI) *m/z* calcd. $C_{18}H_{15}F_3NO_2$: $[M+H]^+$ 334.1049; found: $[M+H]^+$ 334.1045.

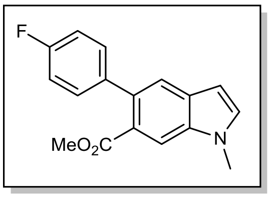

5-(4-fluorophenyl)-1-methyl-1H-indole-6-carboxylic acid (**5ci**)

The General Procedure **A** was applied with 1-methyl-1*H*-indole-6-carboxylic acid (52.6 mg, 0.30 mmol) and 1-iodo-4-fluorobenzene (69.2 µL, 2.0 equiv, 0.60 mmol) with $[Ru(^tBuCN)_6](BF_4)_2$ (11.6 mg, 5.0 mol %) for 8 h. Column chromatography (0-20% EtOAc/hexane), after derivatization with MeI, afforded the title product as a white solid (47.6 mg, 56%).

^1H NMR (500 MHz, $(CD_3)_2CO$) δ 7.94 (s, 1H), 7.52 (s, 1H), 7.46 (d, *J* = 2.9 Hz, 1H), 7.34 - 7.31 (m, 2H), 7.16 - 7.11 (m, 2H), 6.53 (d, *J* = 3.0 Hz, 1H), 3.94 (s, 3H), 3.61 (s, 3H); ^{13}C NMR (126 MHz, $(CD_3)_2CO$) δ 169.8, 162.6 (d, *J* = 242.7 Hz), 140.4 (d, *J*

= 3.3 Hz), 136.2, 133.8, 133.5, 131.7, 131.4 (d, J = 8.1 Hz), 125.0, 123.4, 115.2 (d, J = 21.4 Hz), 112.9, 101.9, 51.8; 33.2; ^{19}F NMR (376 MHz, $(CD_3)_2CO$) δ –118.94 (tt, J = 9.0, 5.5 Hz, 1F); m.p. 138-140 °C; IR (ATR) 3095, 2986, 1713, 1510, 1474, 1233; HRMS (ESI) *m/z* calcd. $C_{17}H_{15}FNO_2$: $[M+H]^+$ 284.1081; found: $[M+H]^+$ 284.1076.

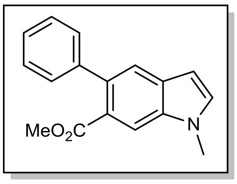

1-methyl-5-phenyl-1H-indole-6-carboxylic acid (**5ce**)

The General Procedure **A** was applied with 1-methyl-1*H*-indole-6-carboxylic acid (52.6 mg, 0.30 mmol) and 1-iodobenzene (66.9 µL, 2.0 equiv, 0.60 mmol) with $[Ru(^tBuCN)_6](BF_4)_2$ (11.6 mg, 5.0 mol %) and H_2O (16.2 µL, 3.0 equiv, 0.90 mmol) for 3 h. Column chromatography (0-20% EtOAc/hexane), after derivatization with MeI, afforded the title product as a white solid (57.3 mg, 72%).

^{1}H NMR (400 MHz, $CDCl_3$) δ 7.94 (s, 1H), 7.58 (s, 1H), 7.41 - 7.30 (m, 5H), 7.22 (d, J = 3.0 Hz, 1H), 6.52 (d, J = 2.5 Hz, 1H), 3.87 (s, 3H), 3.65 (s, 3H); ^{13}C NMR (101 MHz, $CDCl_3$) δ 170.0, 143.2, 135.3, 134.4, 132.3, 130.9, 128.9, 127.9, 126.4, 124.1, 123.0, 112.1, 101.5, 51.9, 33.2; m.p. 130-134 °C; IR (ATR) 3104, 2939, 1716, 1476, 1233, 1033, 703; HRMS (ESI) m/z calcd. $C_{17}H_{16}NO_2$: $[M+H]^+$ 266.1176; found: $[M+H]^+$ 266.1163.

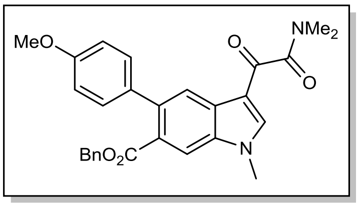

Benzyl 3-(2-(dimethylamino)-2-oxoacetyl)-5-(4-methoxyphenyl)-1-methyl-1H-indole-6-carboxylate (**5da**)

The General Procedure **A** was applied with 3-(2-(dimethylamino)-2-oxoacetyl)-1-methyl-1H-indole-6-carboxylic acid (82.3 mg, 0.30 mmol) and 4-iodoanisole (140.4 mg, 2.0 equiv, 0.60 mmol) with $[Ru(^tBuCN)_6](BF_4)_2$ (11.6 mg, 5 mol %) for 5 h. Column chromatography ($(CH_3)_2CO/Et_2O$ 20:80), after derivatization with BnCl, afforded the title product as a off-white solid (105.9 mg, 75%).

^{1}H NMR (400 MHz, $CDCl_3$) δ 8.33 (s, 1H), 7.99 (s, 1H), 7.89 (s, 1H), 7.36 - 7.19 (m, 5H), 7.09 - 6.99 (m, 2H), 6.92 - 6.82 (m, 2H), 5.11 (s, 2H), 3.88 (s, 3H), 3.82 (s, 3H), 3.08 (s, 3H), 3.06 (s, 3H); ^{13}C NMR (126 MHz, $CDCl_3$) δ 185.2, 169.3, 167.3, 158.9, 141.7, 137.8, 136.1, 135.5, 134.6, 130.0, 128.7, 128.4, 128.4, 128.2, 127.1, 125.6,

113.6, 113.5, 112.4, 67.2, 55.4, 37.6, 34.7, 34.1; m.p. 162-164 °C; IR (ATR) 3125, 2924, 2842, 1714, 1628, 1626, 1175, 749; HRMS (ESI) *m/z* calcd. $C_{28}H_{26}N_2NaO_5$: $[M+Na]^+$ 493.1739; found $[M+Na]^+$: 493.1718.

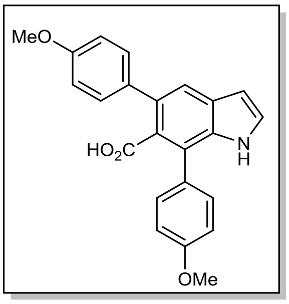

5,7-bis(4-methoxyphenyl)-1H-indole-6-carboxylic acid (**5ea**)

The General Procedure **B** was applied with 1*H*-indole-6-carboxylic acid (48.3 mg, 0.30 mmol) and 4-iodoanisole (280.8 mg, 4.0 equiv, 1.20 mmol) in the absence of H_2O for 12 h. Column chromatography (hexane/EtOAc/AcOH 80:20:1) afforded the title product as white solid (77.2 mg, 69%).

^{1}H NMR (500 MHz, $(CD_3)_2CO$) δ 10.71 (s, 1H), 10.00 (s, 1H), 7.52 (s, 1H), 7.45 - 7.41 (m, 4H), 7.40 (t, *J* = 2.5 Hz, 1H), 7.02 - 7.00 (m, 2H), 6.96 - 6.93 (m, 2H), 6.59 - 6.58 (m, 1H), 3.86 (s, 3H), 3.82 (s, 3H); ^{13}C NMR (126 MHz, $(CD_3)_2CO$) δ 171.2, 160.2, 159.6, 135.7, 134.7, 132.1, 131.6, 130.9, 130.0, 129.4, 128.9, 128.1, 124.1, 121.3, 114.7, 114.2, 102.9, 55.6, 55.5; m.p. 138-140 °C; IR (ATR) 3384, 2965, 2860, 1701, 1516, 1248, 1027, 832; HRMS (ESI) *m/z* calcd. $C_{23}H_{20}NO_4$: $[M+H]^+$ 374.1387; found $[M+H]^+$: 374.1385.

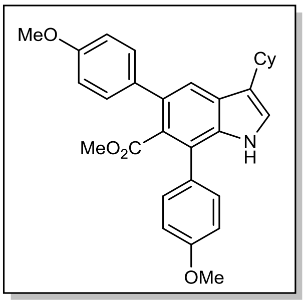

Methyl 3-cyclohexyl-5,7-bis(4-methoxyphenyl)-1H-indole-6-carboxylate (**5fa**)

The General Procedure **B** was applied with 3-cyclohexyl-1-methyl-1H-indole-6-carboxylic acid (77 mg, 0.3 mmol) and 4-iodoanisole (280.8 mg, 4.0 equiv 1.2 mmol) with $[Ru(^tBuCN)_6](BF_4)_2$ (5 mol %) in the absence of H_2O for 12 h Column chromatography (0-20% EtOAc/hexane) afforded the title product as white solid (77.2 mg, 59%).

^{1}H NMR (500 MHz, $(CD_3)_2CO$) δ 9.73 (br s, 1H), 7.56 (d, *J* = 0.7 Hz, 1H), 7.38 - 7.34 (m, 4H), 7.17 (dd, *J* = 2.5, 0.8 Hz, 1H), 7.01 (dm, *J* = 8.8 Hz, 2H), 6.96 (dm, *J* = 8.8 Hz, 2H), 3.85 (s, 3H), 3.83 (s, 3H), 3.31 (s, 3H), 2.93 - 2.88 (m, 1H), 2.14 - 2.10 (m, 2H), 1.85 - 1.82 (m, 2H), 1.78-1.74 (m, 1H), 1.56 - 1.45 (m, 4H), 1.38 - 1.30 (m, 1H); ^{13}C NMR (101 MHz, $(CD_3)_2CO$) δ 171.0, 160.2, 159.6, 135.8, 134.9, 131.7, 131.3,

130.7, 129.9, 128.4, 127.9, 124.6, 123.7, 123.5, 119.8, 114.8, 114.3, 55.6, 55.5, 51.6, 36.2, 34.9, 27.6, 27.2; m.p. 164-166 °C; IR (ATR) 2924, 2851, 1709, 1615, 1470, 1277, 1240, 1090; HRMS (ESI) *m/z* calcd. $C_{30}H_{31}NO_4Na$: $[M+Na]^+$ 492.2145; found $[M+Na]^+$: 492.2139.

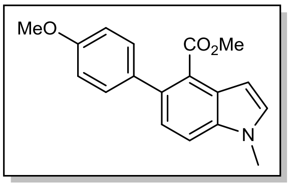

5-(4-methoxyphenyl)-1-methyl-1H-indole-4-carboxylic acid (**5ga**)

The General Procedure **A** was applied with 1-methyl-1*H*-indole-4-carboxylic acid (52.6 mg, 0.30 mmol) and 4-iodoanisole (140.4 mg, 2.0 equiv, 0.60 mmol). Column chromatography (0-15% EtOAc/hexane), after derivatization with MeI, afforded the title product as a light yellow solid (62.0 mg, 70%).

^{1}H NMR (500 MHz, $CDCl_3$) δ 7.44 (d, *J* = 8.4 Hz, 1H), 7.32 (d, *J* = 8.5 Hz, 2H), 7.28 - 7.22 (m, 1H), 7.16 (d, *J* = 3.0 Hz, 1H), 6.95 (d, *J* = 8.5 Hz, 2H), 6.73 (d, *J* = 2.9 Hz, 1H), 3.86 (s, 3H), 3.84 (s, 3H), 3.72 (s, 3H); ^{13}C NMR (126 MHz, $CDCl_3$) δ 169.8, 158.6, 136.2, 140.0, 133.8, 130.8, 129.9, 127.8, 124.3, 122.7, 113.6, 111.7, 101.3, 55.4, 51.8, 33.2; m.p. 160-162 °C; IR (ATR) 3042, 2968, 2152, 1710, 1516, 1245, 686; HRMS (ESI) *m/z* calcd. $C_{18}H_{17}NaNO_3$: $[M+Na]^+$ 318.1106; found $[M+Na]^+$: 318.1094.

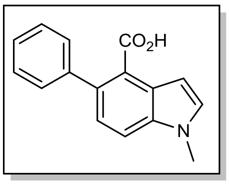

1-methyl-5-phenyl-1H-indole-4-carboxylic acid (**5ge**)

The General Procedure **A** was applied with 1-methyl-1*H*-indole-4-carboxylic acid (52.6 mg, 0.30 mmol) and 4-iodobenzene (66.9 µL, 2.0 equiv, 0.60 mmol). Column chromatography (hexane/EtOAc/AcOH 75:25:1) afforded the title product as orange solid (61.1 mg, 81%).

^{1}H NMR (500 MHz, $(CD_3)_2CO$) 7.58 (d, *J* = 8.4 Hz, 1H), 7.43 (app d, *J* = 7.7 Hz, 2H), 7.39 - 7.36 (m, 3H), 7.30 (app t, *J* = 7.3 Hz, 1H), 7.21 (d, *J* = 8.4 Hz, 1H), 6.72 (s, 1H), 3.90 (s, 3H).; ^{13}C NMR (126 MHz, $(CD_3)_2CO$) δ 170.4, 143.6, 137.3, 134.0, 131.7, 129.8, 128.8, 128.5, 127.2, 124.7, 124.6, 112.3, 102.0, 33.1; m.p. 184-186 °C; IR (ATR) 3048, 2902, 1684, 1507, 1480, 1421, 1242, 761, 694; HRMS (ESI) *m/z* calcd. $C_{16}H_{14}NO_2$: $[M+H]^+$ 252.1019; found $[M+H]^+$: 252.1016.

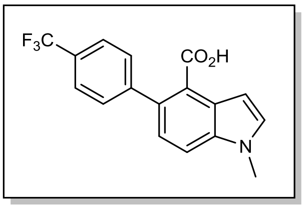

1-methyl-5-(4-(trifluoromethyl)phenyl)-1H-indole-4-carboxylic acid (**5go**)

The General Procedure **A** was applied with 1-methyl-1*H*-indole-4-carboxylic acid (52.6 mg, 0.30 mmol) and 4-iodobenzotrifluoride (88.2 µL, 2.0 equiv, 0.60 mmol). Column chromatography (hexane/EtOAc/AcOH 75:25:1) afforded the title product as light pink solid (66.0 mg, 69%).

^{1}H NMR (500 MHz, $(CD_3)_2CO$) δ 7.73 (d, *J* = 8.2 Hz, 2H), 7.65 (d, *J* = 8.4 Hz, 1H), 7.62 (d, *J* = 8.1 Hz, 2H), 7.42 (d, *J* = 3.1 Hz, 1H), 7.23 (d, *J* = 8.4 Hz, 1H), 6.80 (d, *J* = 3.1 Hz, 1H), 3.93 (s, 3H); ^{13}C NMR (126 MHz, $(CD_3)_2CO$) δ 169.6, 148.1, 137.7, 133.1, 132.3, 130.5, 128.8 (q, *J* = 32.0 Hz), 128.8, 125.7 (q, *J* = 270.9 Hz), 125.6 (q, *J* = 3.8 Hz), 124.4, 124.2, 112.9, 102.3, 33.1; ^{19}F NMR (376 MHz, $(CD_3)_2CO$) δ –62.65 (s, 3F); m.p. 194-200 °C; IR (ATR) 3204, 2924, 1681, 1610, 1321, 1112, 812; HRMS (ESI) *m/z* calcd. $C_{17}H_{13}F_3NO_2$: $[M+H]^+$ 320.0893; found $[M+H]^+$: 320.0888.

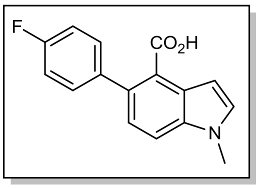

5-(4-fluorophenyl)-1-methyl-1H-indole-4-carboxylic acid (**5gi**)

The General Procedure **A** was applied with 1-methyl-1*H*-indole-4-carboxylic acid (52.6 mg, 0.30 mmol) and 4-fluoroiodobenzene (69.2 µL, 2.0 equiv, 0.60 mmol). Column chromatography (hexane/EtOAc/AcOH 75:25:1) afforded the title product as white solid (55.0 mg, 68%).

^{1}H NMR (500 MHz, $(CD_3)_2CO$) δ 7.60 - 7.58 (m, 1H), 7.42 (dd, *J* = 8.3, 5.6 Hz, 2H), 7.37 (s, 1H), 7.19 - 7.13 (m, 3H), 6.74 (s, 1H), 3.90 (s, 3H).; ^{13}C NMR (126 MHz, $(CD_3)_2CO$) δ 169.2, 161.9 (d, *J* = 243.0 Hz), 139.1 (d, *J* = 3.2 Hz), 136.5, 132.3, 131.0, 130.7 (d, *J* = 8.1 Hz), 127.7, 123.6, 123.5, 114.5 (d, *J* = 21.5 Hz), 111.6, 101.2, 32.2; m.p. 216-220 °C; ^{19}F NMR (376 MHz, $(CD_3)_2CO$) δ –118.62 (tt, *J* = 9.0, 5.5 Hz, 1F); IR (ATR) 3094, 2942, 1684, 1510, 1253, 1215, 850, 809; HRMS (ESI) *m/z* calcd. $C_{16}H_{13}FNO_2$: $[M+H]^+$ 270.0925; found $[M+H]^+$: 270.0922.

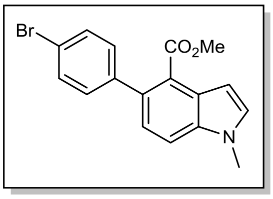

5-(4-bromophenyl)-1-methyl-1H-indole-4-carboxylic acid (**5gk**)

The General Procedure **A** was applied with 1-methyl-1*H*-indole-4-carboxylic acid (52.6 mg, 0.30 mmol) and 1-bromo-4-iodobenzene (169.7 mg, 2.0 equiv 0.60 mmol). Column chromatography (0-15% EtOAc/hexane), after derivatization with MeI, afforded the title product as a white solid (62.0 mg, 60%).

^{1}H NMR (400 MHz, $(CD_3)_2CO$) δ 7.62 (d, *J* = 8.4 Hz, 1H), 7.60 - 7.56 (m, 2H), 7.40 (d, *J* = 3.1 Hz, 1H), 7.32 - 7.25 (m, 2H), 7.21 (d, *J* = 8.4 Hz, 1H), 6.67 (d, *J* = 3.1 Hz, 1H), 3.91 (s, 3H), 3.67 (s, 3H). ^{13}C NMR (101 MHz, $(CD_3)_2CO$) δ 169.5, 142.8, 137.5, 133.0, 132.3, 131.9, 131.6, 128.6, 124.2, 123.7, 121.0, 112.9, 101.8, 51.8, 33.1; m.p. 164-166 °C; IR (ATR) 3068, 2892, 1664, 1497, 1466, 1390, 1142, 721, 692; HRMS (ESI) *m/z* calcd. $C_{17}H_{14}NBrO_3Na$: $[M+Na]^+$ 366.0106; found $[M+Na]^+$: 366.0103.

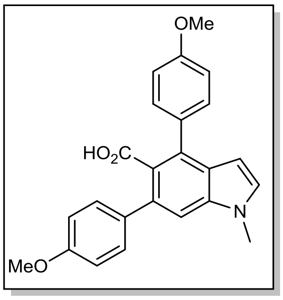

4,6-bis(4-methoxyphenyl)-1-methyl-1H-indole-5-carboxylic acid (**5ia**)

The General Procedure **B** was applied with 1-methyl-1*H*-indole-5-carboxylic acid (52.6 mg, 0.30 mmol) and 4-iodoanisole (280.8 mg, 4.0 equiv, 1.20 mmol) for 16 h. Column chromatography (hexane/EtOAc/AcOH 80:20:1) afforded the title product as a yellow solid (102.3 mg, 88%).

^{1}H NMR (500 MHz, $(CD_3)_2CO$) δ 7.47 - 7.44 (m, 4H), 7.32 (s, 1H), 7.29 (d, *J* = 2.2 Hz, 1H), 7.01 - 6.93 (m, 4H), 6.26 (d, *J* = 3.1 Hz, 1H), 3.89 (s, 3H), 3.85 (s, 3H), 3.83 (s, 3H); ^{13}C NMR (126 MHz, $(CD_3)_2CO$) δ 171.8, 159.9, 159.8, 137.4, 135.7, 134.4, 132.5, 131.4, 131.3, 131.0, 127.8, 127.0, 114.2, 110.5, 101.5, 55.5, 55.5, 33.1, 32.3, 23.3; m.p. 192-194 °C; IR (ATR) 2984, 2722, 1694, 1600, 1392, 1333, 1212, 990; HRMS (ESI) *m/z* calcd. $C_{24}H_{22}NO_4$: $[M+H]^+$ 388.1543; found $[M+H]^+$: 388.1533.

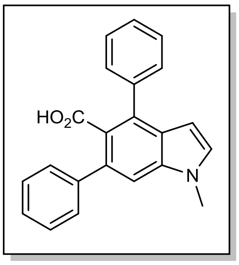

1-methyl-4,6-diphenyl-1H-indole-5-carboxylic acid (**5ie**)

The General Procedure **B** was applied with 1-methyl-1*H*-indole-5-carboxylic acid (52.6 mg, 0.30 mmol) and iodobenzene (133.8 µL, 4.0 equiv, 1.20 mmol for 16 h. Column chromatography (hexane/EtOAc/AcOH 80:20:1) afforded the title product as white solid (90.4 mg, 92%).

^{1}H NMR (400 MHz, $(CD_3)_2CO$) δ 7.57 - 7.53 (m, 4H), 7.47 - 7.32 (m, 8H), 6.26 (d, *J* = 2.9 Hz, 1H), 3.91 (s, 3H); ^{13}C NMR (101 MHz, $(CD_3)_2CO$) δ 171.4, 143.4, 140.3, 137.4, 134.8, 133.0 (2C), 131.7, 130.3, 129.9, 128.8, 128.0, 127.9, 127.6, 126.6, 111.0, 101.5, 33.1; m.p. 210-214 °C; IR (ATR) 3021, 2699, 1684, 1588, 1387, 1354, 1199, 1010; HRMS (ESI) *m/z* calcd. $C_{22}H_{18}NO_2$: $[M+H]^+$ 328.1332; found $[M+H]^+$: 328.1322.

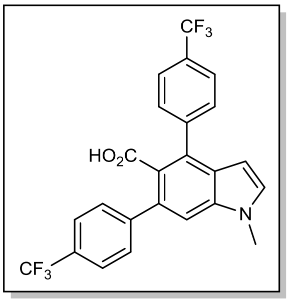

1-methyl-4,6-bis(4-(trifluoromethyl)phenyl)-1H-indole-5-carboxylic acid (**5io**)

The General Procedure **B** was applied with 1-methyl-1*H*-indole-5-carboxylic acid (52.6 mg, 0.30 mmol) and 1-iodo-4-(trifluoromethyl)benzene (176.4 µL, 4.0 equiv, 1.20 mmol) for 16 h. Column chromatography (hexane/EtOAc/AcOH 80:20:1) afforded the title product as white solid (116.8 mg, 84%).

^{1}H NMR (400 MHz, $(CD_3)_2CO$) δ 11.03 (br s, 1H), 7.83 (d, *J* = 8.2 Hz, 2H), 7.80 -7.70 (m, 6H), 7.57 (s, 1H), 7.46 - 7.40 (m, 1H), 6.28 (dd, *J* = 3.2, 0.9 Hz, 1H), 3.96 (s, 3H); ^{13}C NMR (101 MHz, $(CD_3)_2CO$) δ 170.9, 147.3 (d, *J* = 1.1 Hz), 144.4 (d, *J* = 1.4 Hz), 137.5, 133.4, 132.8, 132.0, 131.0, 130.5, 129.7 (q, *J* = 32.2 Hz), 129.3 (q, *J* = 32.1 Hz) 128.1, 126.1, 126.0 - 125.7 (m, 2 C), 125.6 (q, *J* = 271.1 Hz), 125.5 (q, *J* = 271.4 Hz), 111.9, 101.3, 33.3; ^{19}F NMR (376 MHz, $(CD_3)_2CO$) δ –62.75 (s, 3F), –62.81 (s, 3F); m.p. 196-198 °C; IR (ATR) 2977, 2701, 1644, 1590, 1382, 1211, 1112, 850; HRMS (ESI) *m/z* calcd. $C_{24}H_{16}F_6NO_2$: $[M+H]^+$ 464.1080; found $[M+H]^+$: 464.1071.

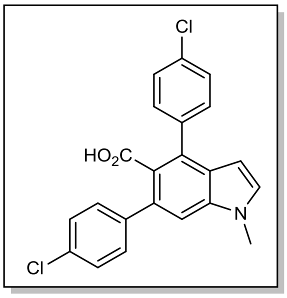

4,6-bis(4-chlorophenyl)-1-methyl-1H-indole-5-carboxylic acid (**5ij**)

The General Procedure **B** was applied with 1-methyl-1*H*-indole-5-carboxylic acid (52.6 mg, 0.30 mmol) and 1-chloro-4-iodobenzene (286.2 mg, 4.0 equiv, 1.20 mmol) for 16 h. Column chromatography (hexane/EtOAc/AcOH 80:20:1) afforded the title product as white solid (90.3 mg, 76%).

^{1}H NMR (500 MHz, $(CD_3)_2CO$) δ 10.90 (br s, 1H), 7.54 - 7.48 (m, 6H), 7.46 - 7.42 (m, 3H), 7.37 (s, 1H), 6.27 (d, *J* = 3.1 Hz, 1H), 3.92 (s, 3H); ^{13}C NMR (126 MHz, $(CD_3)_2CO$) δ 171.1, 142.0, 139.0, 137.4, 133.7, 133.4, 133.3, 132.3, 131.9, 131.9, 131.5, 129.0, 128.9, 127.9, 126.4, 111.3, 101.3, 33.2; m.p. 202-206 °C; IR (ATR) 3122, 2611, 1701, 1544, 1412, 1350, 888; HRMS (ESI) *m/z* calcd. $C_{22}H_{16}Cl_2NO_2$: $[M+H]^+$ 396.0553; found $[M+H]^+$: 396.0544.

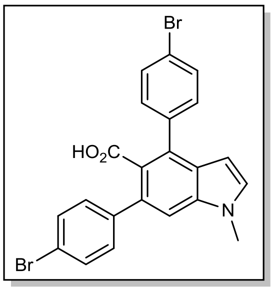

4,6-bis(4-bromophenyl)-1-methyl-1H-indole-5-carboxylic acid (**5ik**)

The General Procedure **B** was applied with 1-methyl-1*H*-indole-5-carboxylic acid (52.6 mg, 0.30 mmol) and 1-chloro-4-iodobenzene (339.4 mg, 4.0 equiv, 1.20 mmol) for 16 h. Column chromatography (hexane/EtOAc/AcOH 80:20:1) afforded the title product as white solid (119.4mg, 82%).

^{1}H NMR (500 MHz, $(CD_3)_2CO$) δ 10.92 (br s, 1H), 7.65 (d, *J* = 8.4 Hz, 2H), 7.59 (d, *J* = 8.3 Hz, 2H), 7.48 - 7.45 (m, 5H), 7.38 (d, *J* = 2.4 Hz, 1H), 6.27 (d, *J* = 3.1 Hz, 1H), 3.93 (s, 3H). ^{13}C NMR (126 MHz, $(CD_3)_2CO$) δ 171.0, 142.4, 139.4, 137.4, 133.5, 132.4, 132.3, 132.0, 131.9, 131.9, 131.8, 127.9, 126.2, 121.9, 121.5, 111.3, 101.3, 33.2; m.p. 186-190 °C; IR (ATR) 3002, 2692, 1681, 1549, 1379, 1239, 1112, 780; HRMS (ESI) *m/z* calcd. $C_{22}H_{16}Br_2NO_2$: $[M+H]^+$ 483.9542; found $[M+H]^+$: 483.9532.

^{1}H NMR, ^{13}C NMR and ^{19}F NMR spectra

^{1}H NMR ($(CD_3)_3CO$)

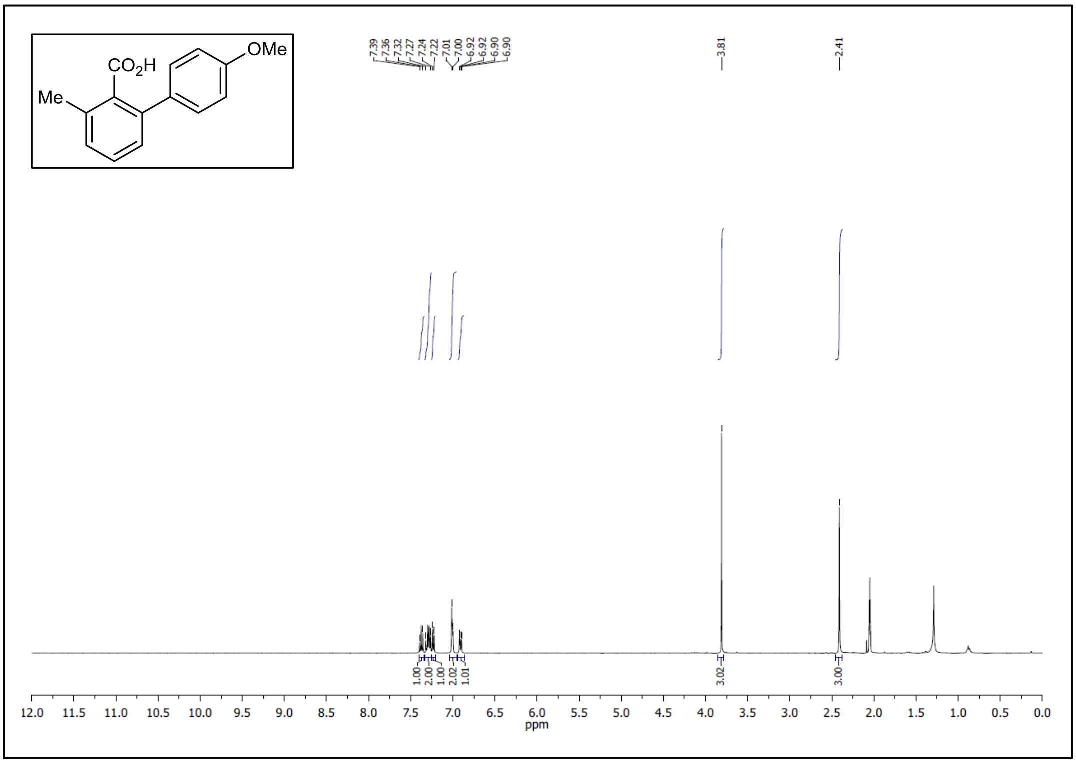

^{13}C NMR ($(CD_3)_3CO$)

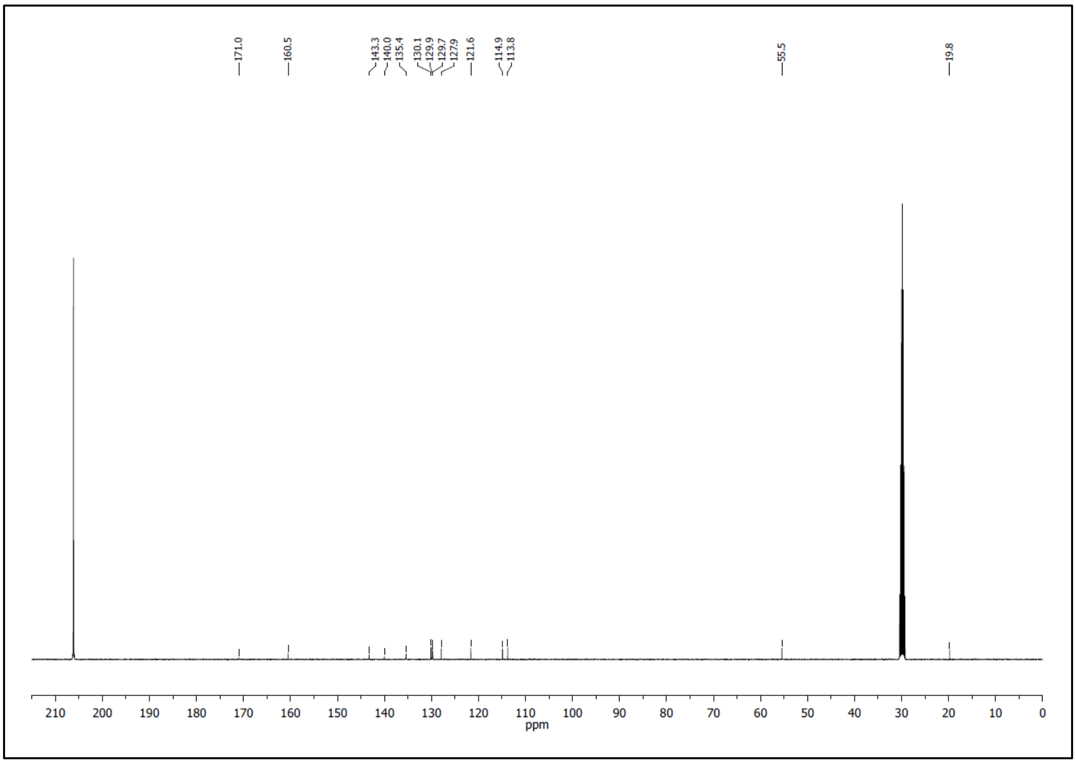

2-(3-methoxyphenyl)-6-methylbenzoic acid (**3ab**)

^{1}H NMR ($(CD_3)_3CO$)

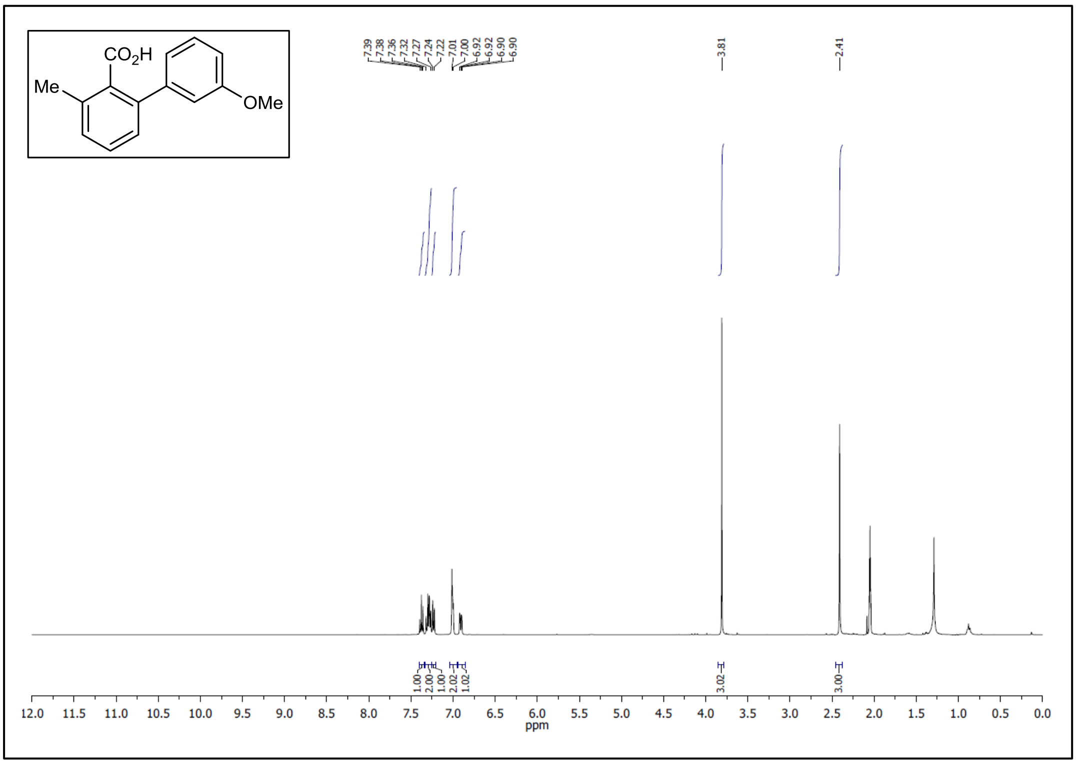

^{13}C NMR ($(CD_3)_3CO$

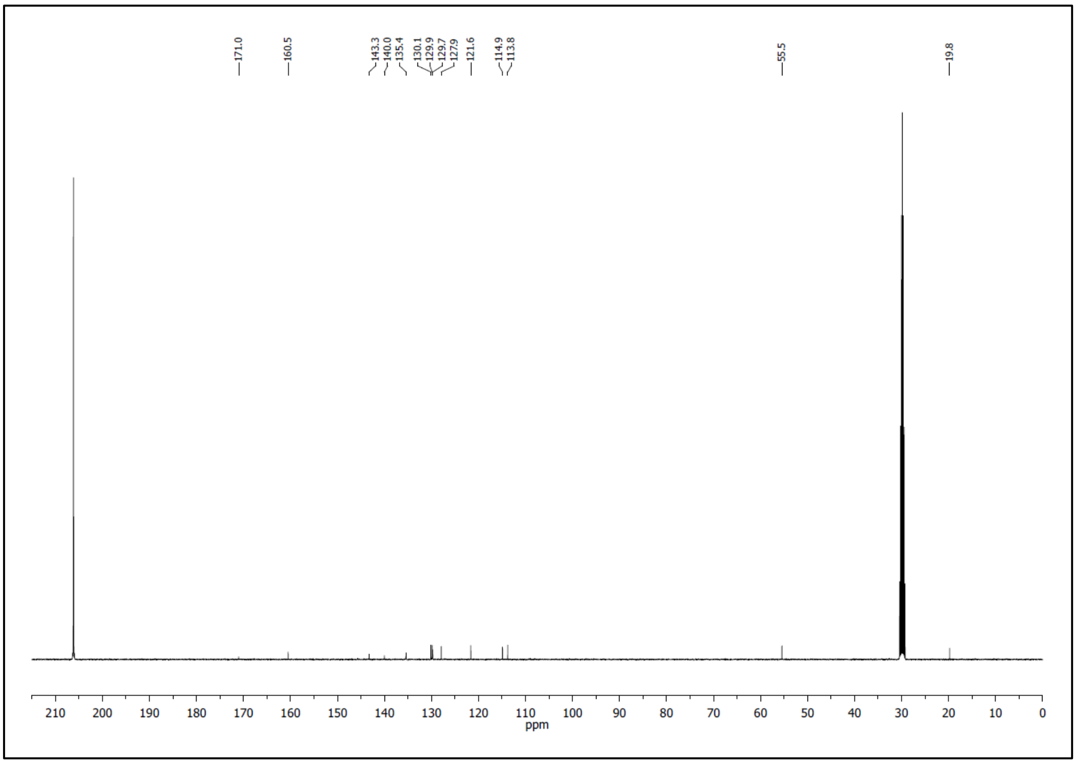

methyl 2'-methoxy-3-methyl-[1,1'-biphenyl]-2-carboxylate (**3ac**)

^{1}H NMR ($CDCl_3$)

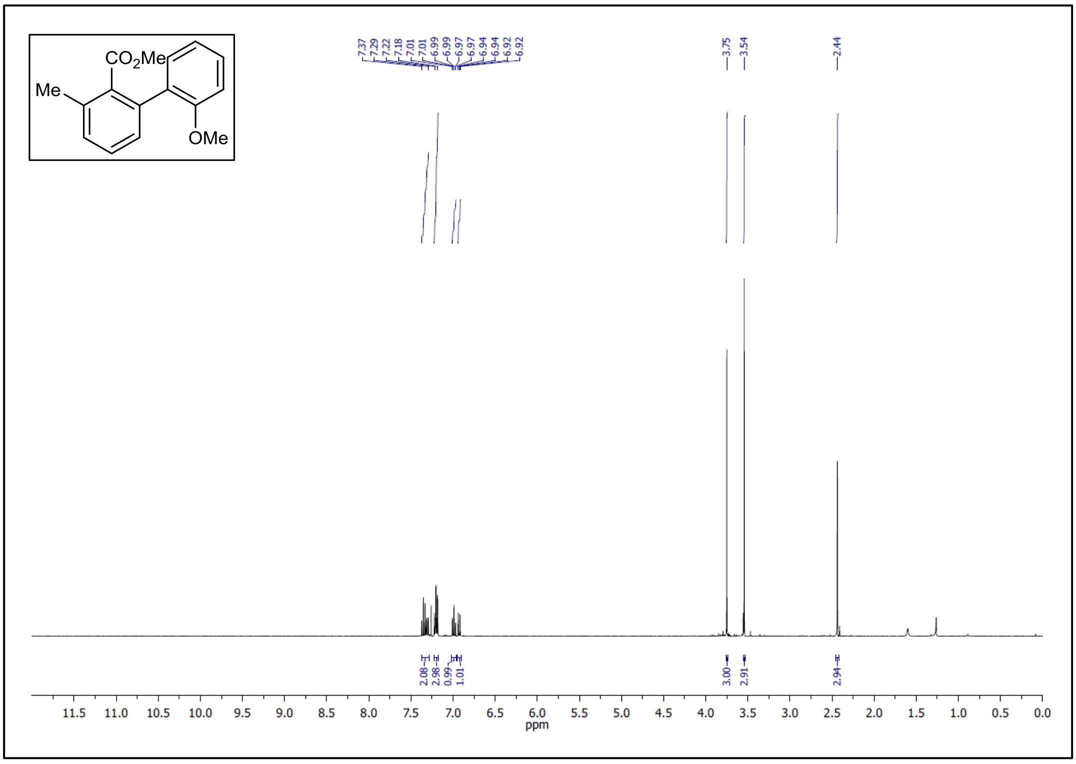

^{13}C NMR ($CDCl_3$)

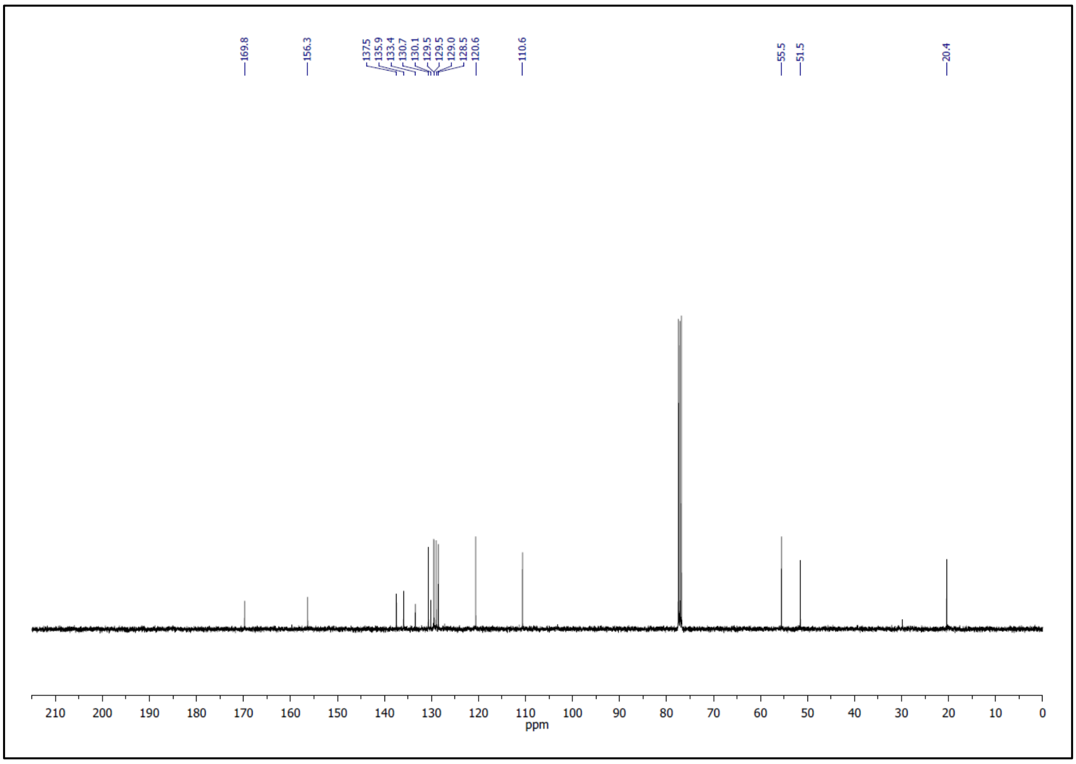

methyl 3-methyl-2'-phenoxy-[1,1'-biphenyl]-2-carboxylate (**3ad**)

^{1}H NMR ($CDCl_3$)

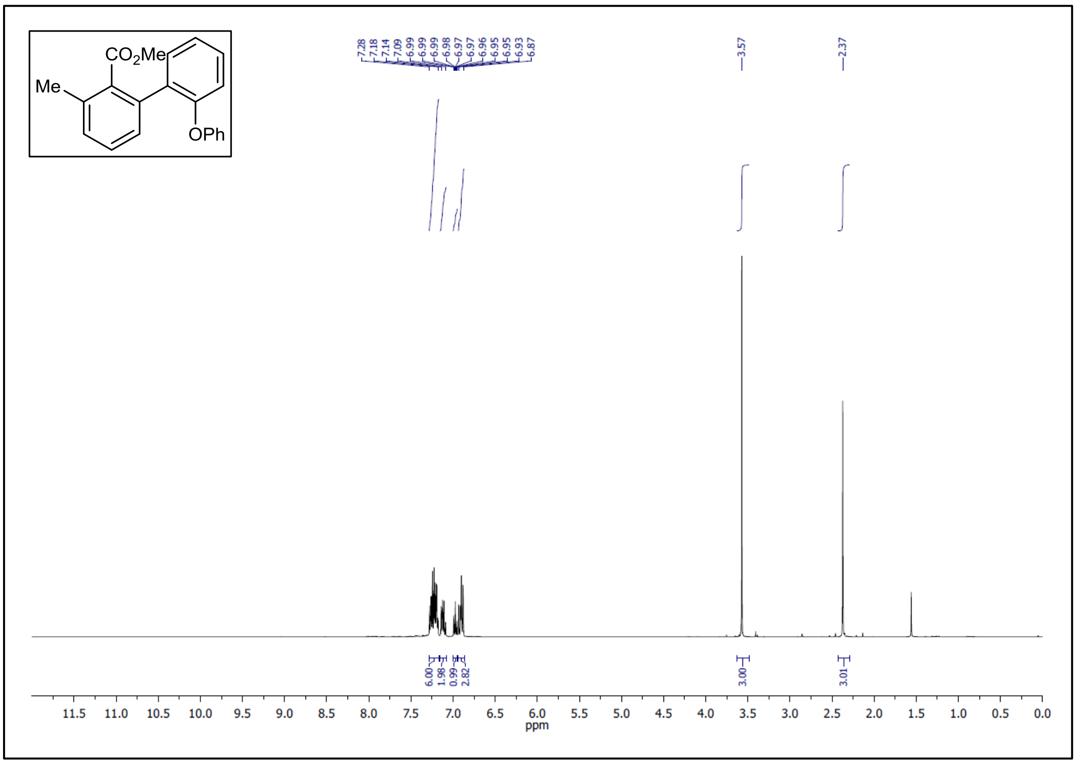

^{13}C NMR ($CDCl_3$)

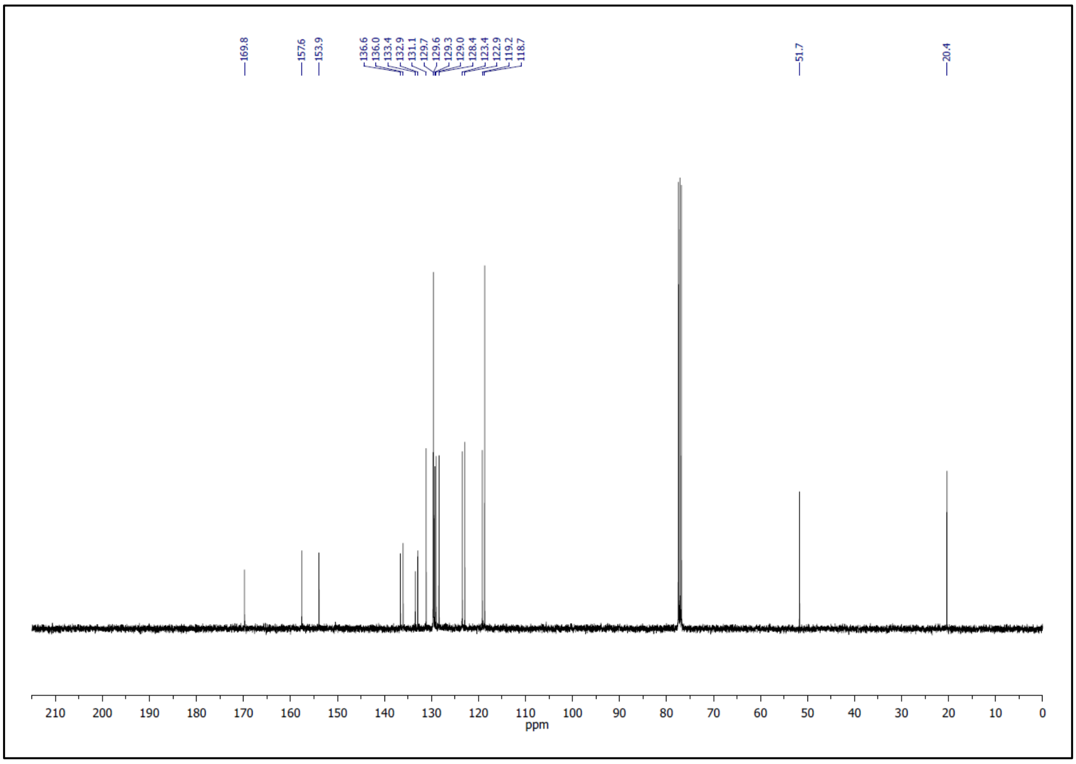

3-methyl-[1,1'-biphenyl]-2-carboxylic acid (**3ae**)

^{1}H NMR ($(CD_3)_3CO$)

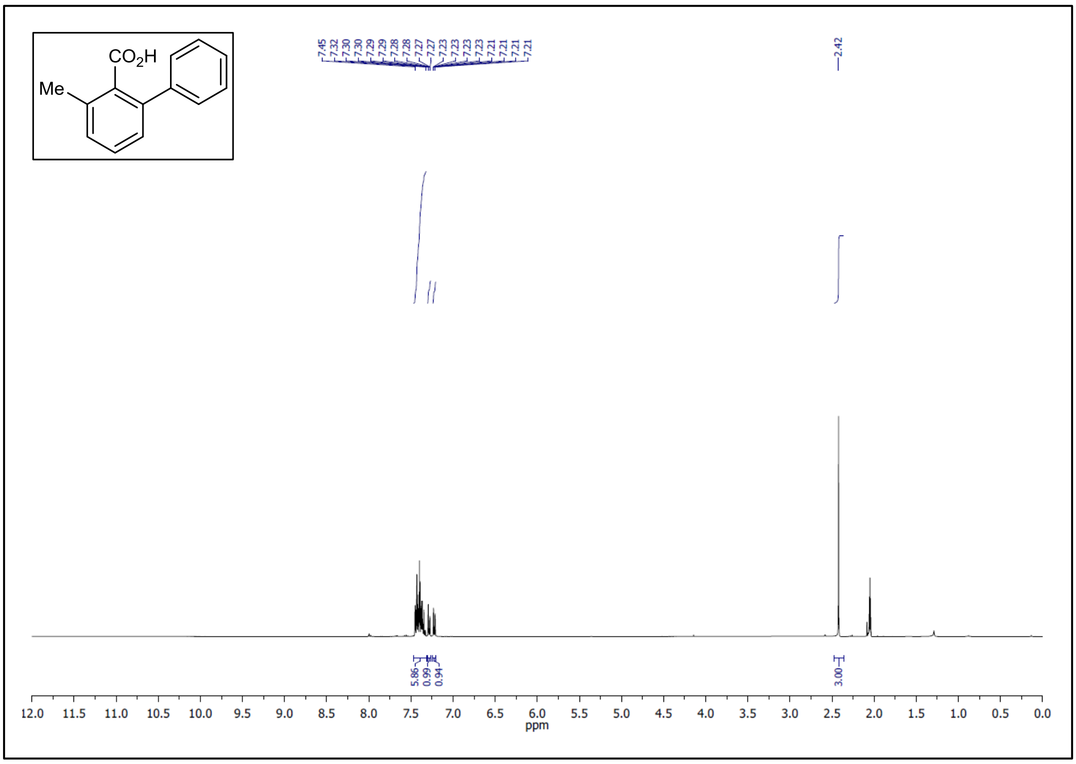

^{13}C NMR ($(CD_3)_3CO$)

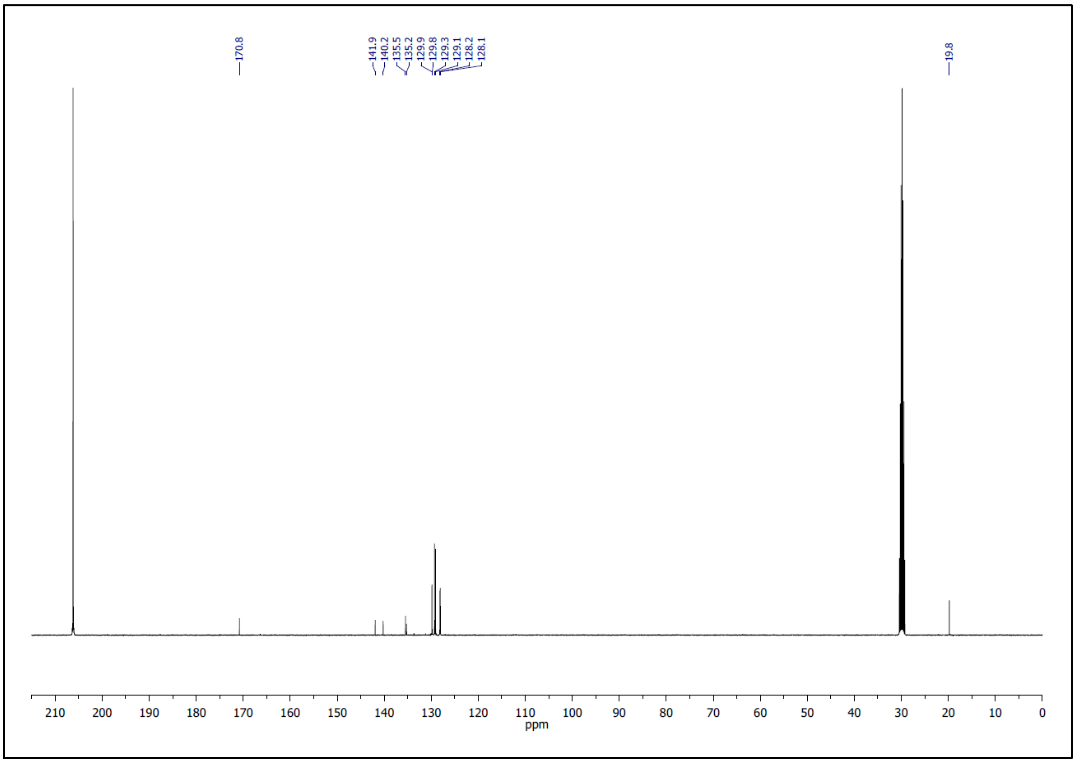

2-methyl-6-(4-methylphenyl)benzoic acid (**3af**)

^{1}H NMR ($CDCl_3$)

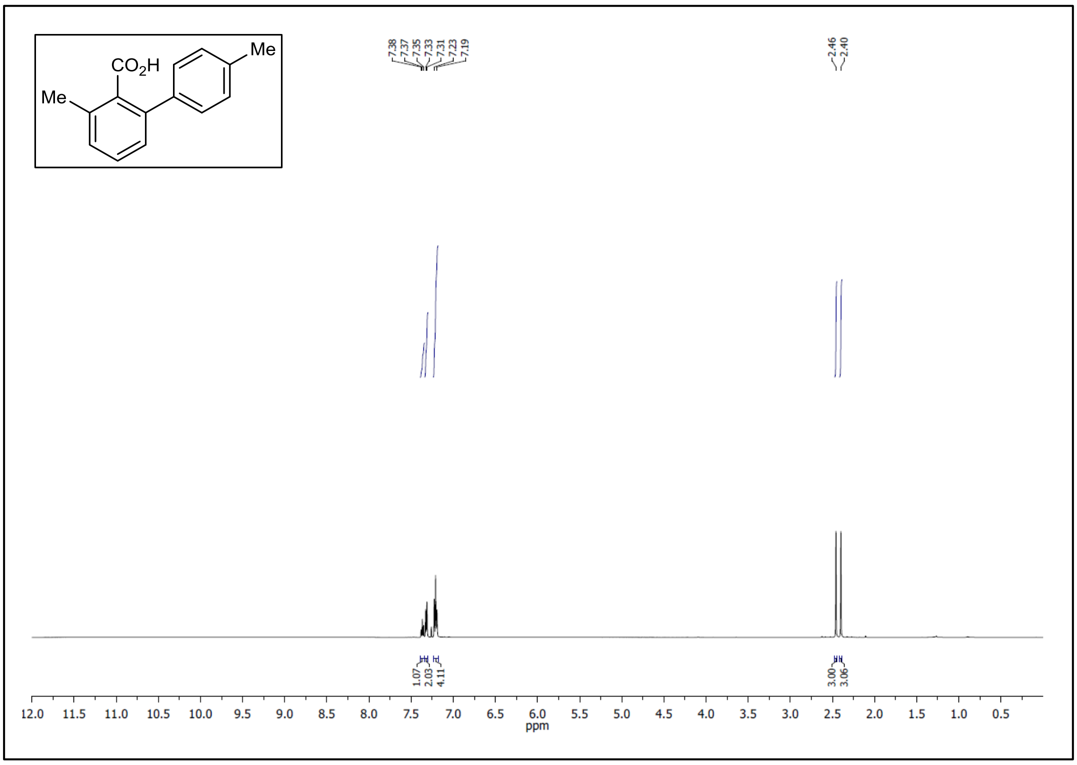

^{13}C NMR ($CDCl_3$)

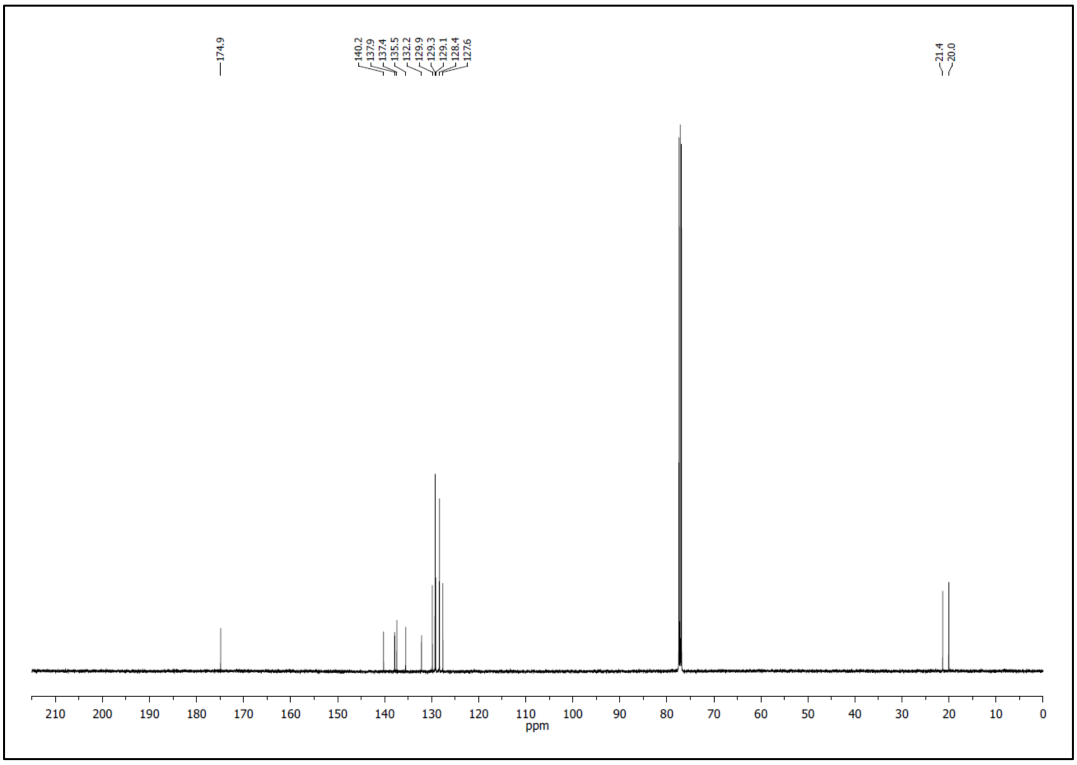

3,3'-dimethyl-[1,1'-biphenyl]-2-carboxylic acid (**3ag**)

^{1}H NMR ($(CD_3)_3CO$)

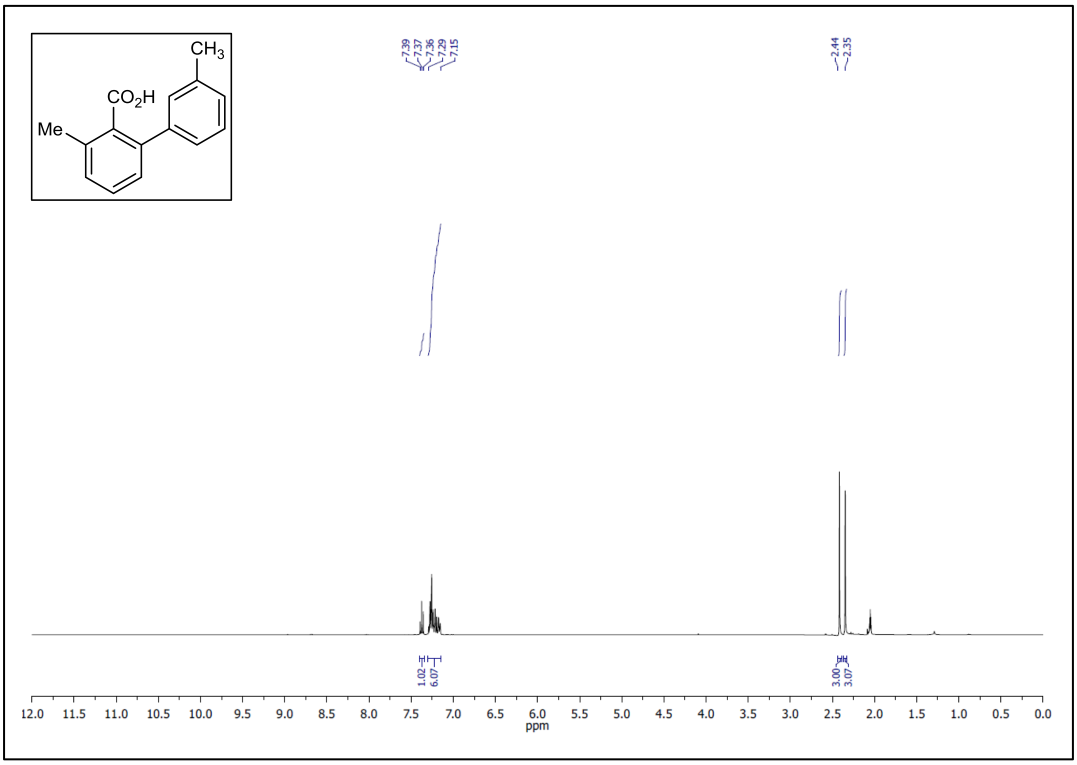

^{13}C NMR ($(CD_3)_3CO$)

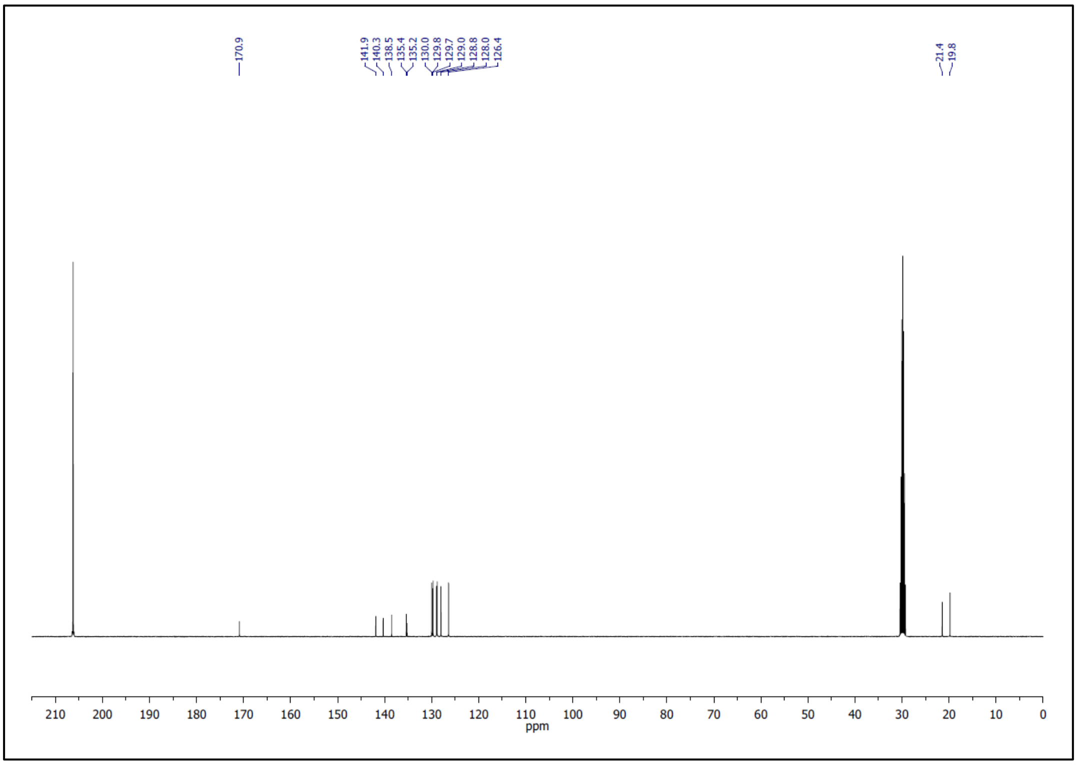

methyl 2',3-dimethyl-[1,1'-biphenyl]-2-carboxylate (**3ah**)

^{1}H NMR ($CDCl_3$)

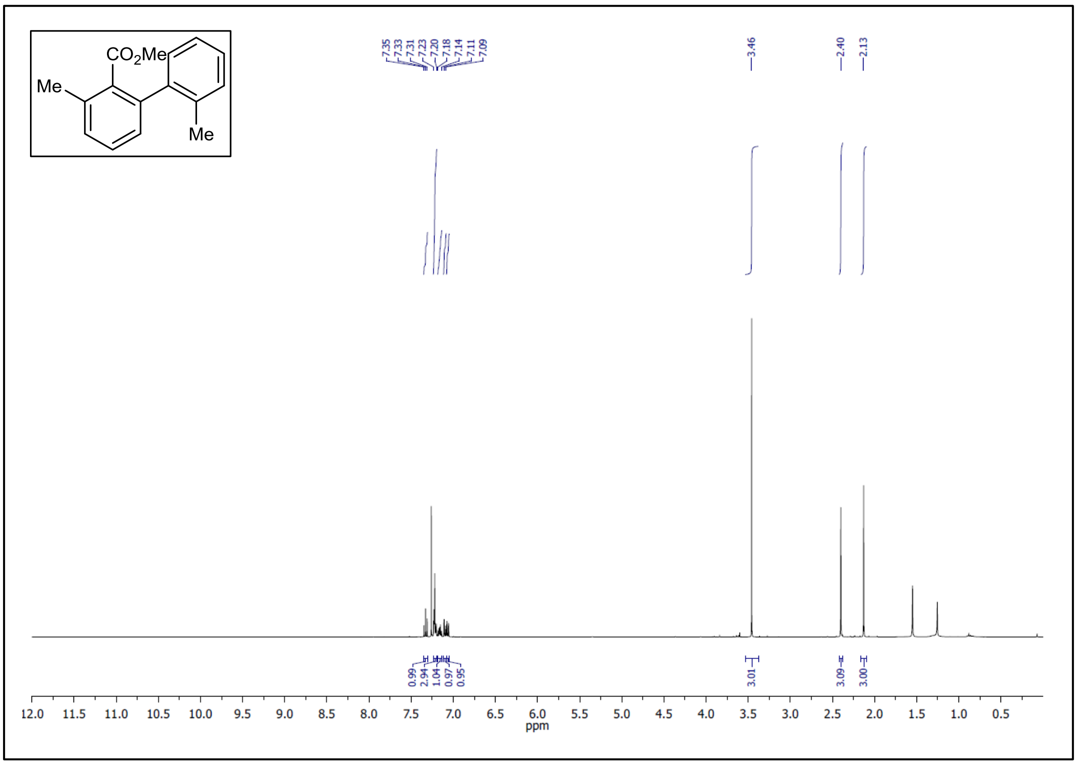

^{13}C NMR ($CDCl_3$)

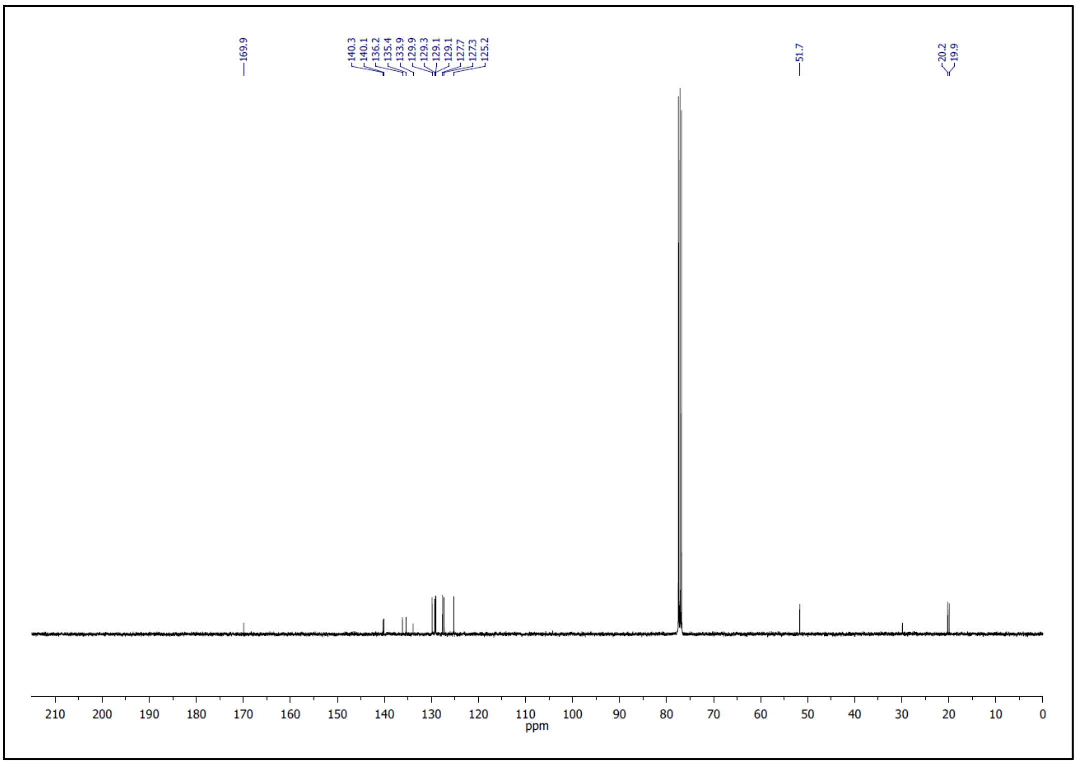

4'-fluoro-3-methyl-[1,1'-biphenyl]-2-carboxylic acid (**3ai**)

^{1}H NMR ($(CD_3)_3CO$)

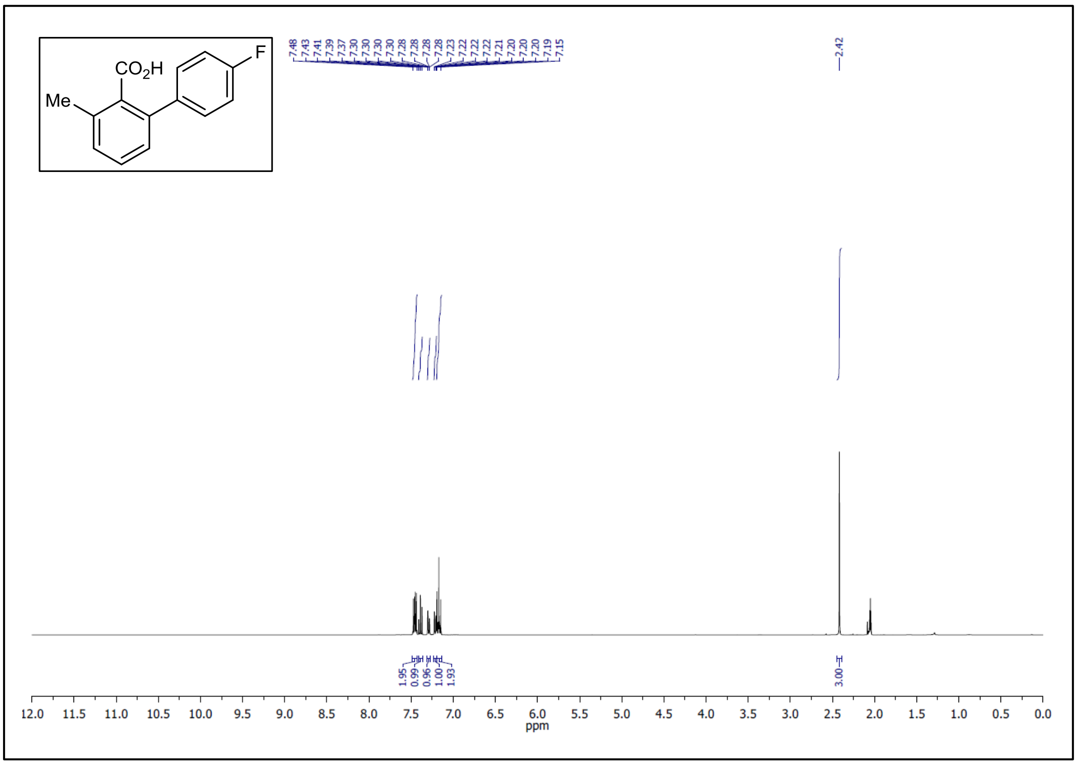

^{13}C NMR ($(CD_3)_3CO$)

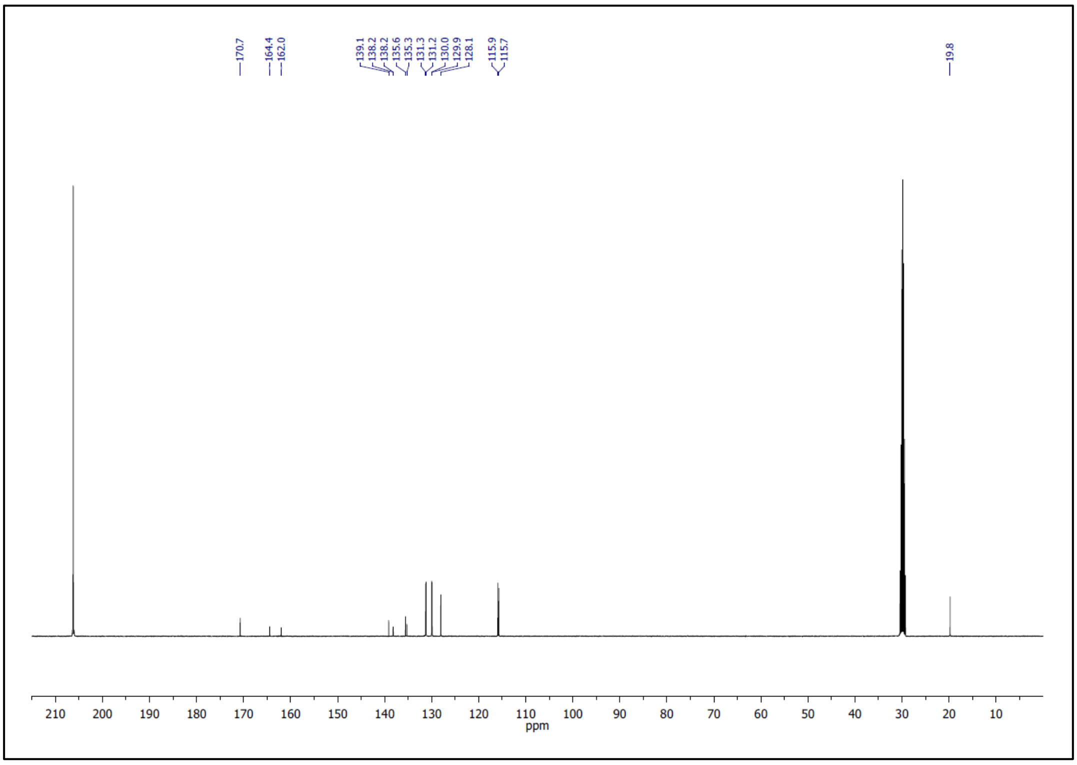

^{19}F NMR ($(CD_3)_3CO$)

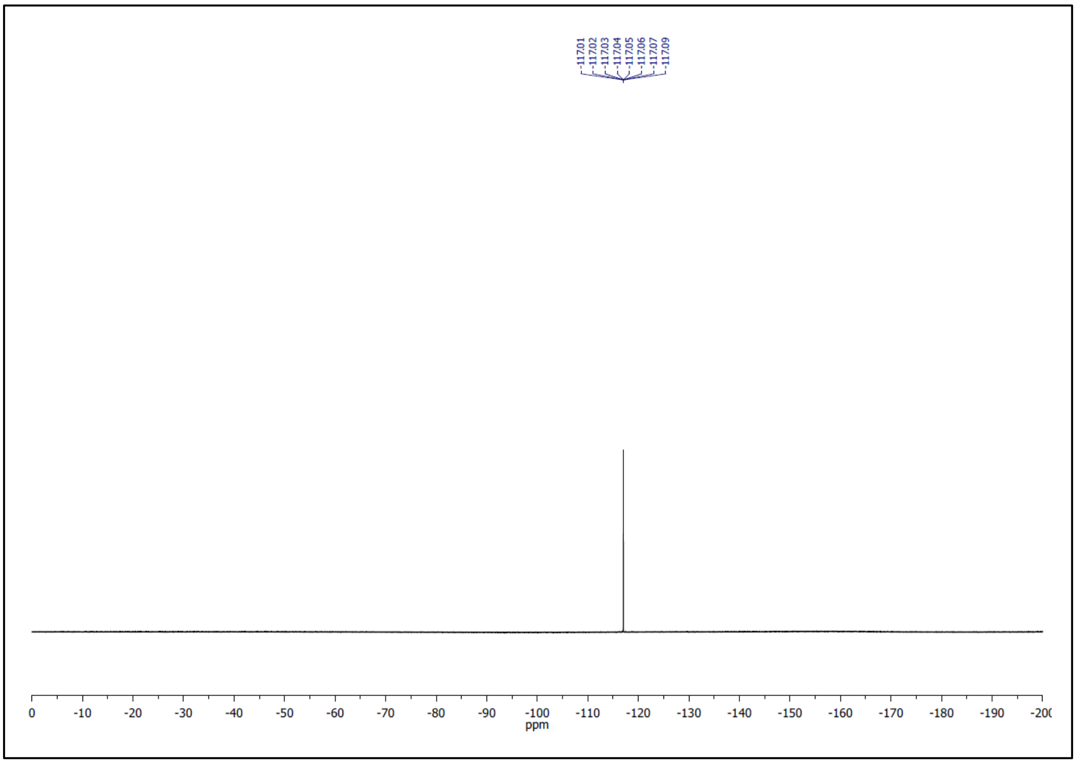

2-(4-chlorophenyl)-6-methylbenzoic acid (**3aj**)

^{1}H NMR ($CDCl_3$)

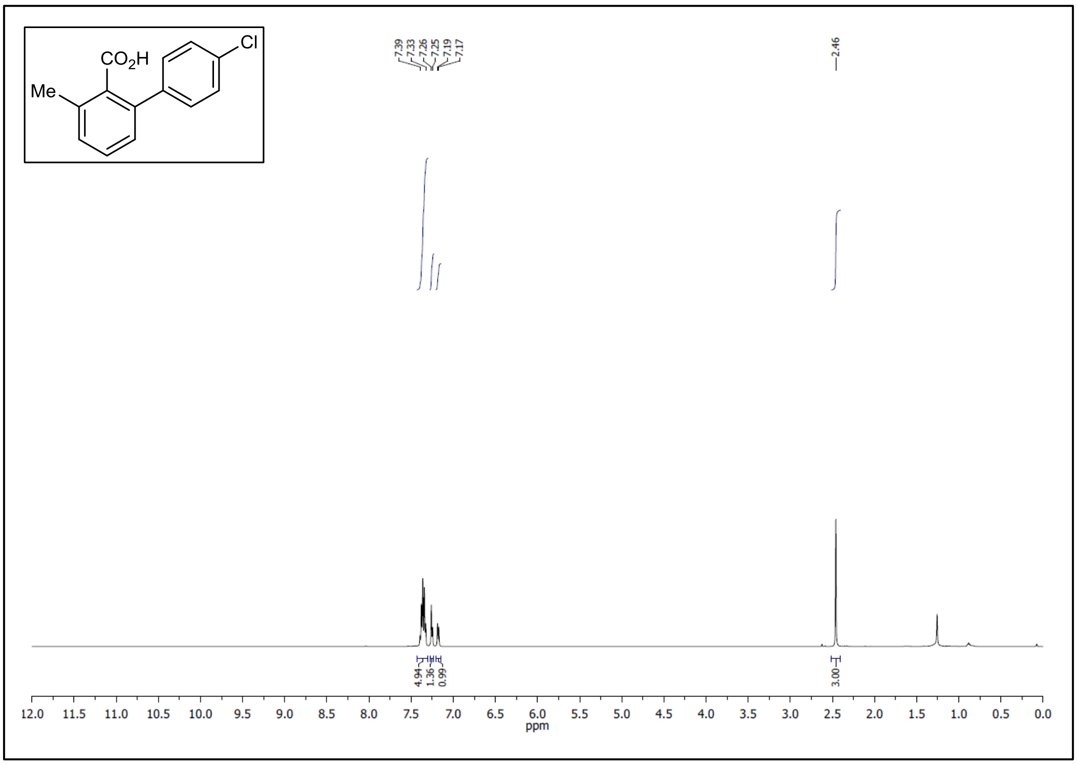

^{13}C NMR ($CDCl_3$)

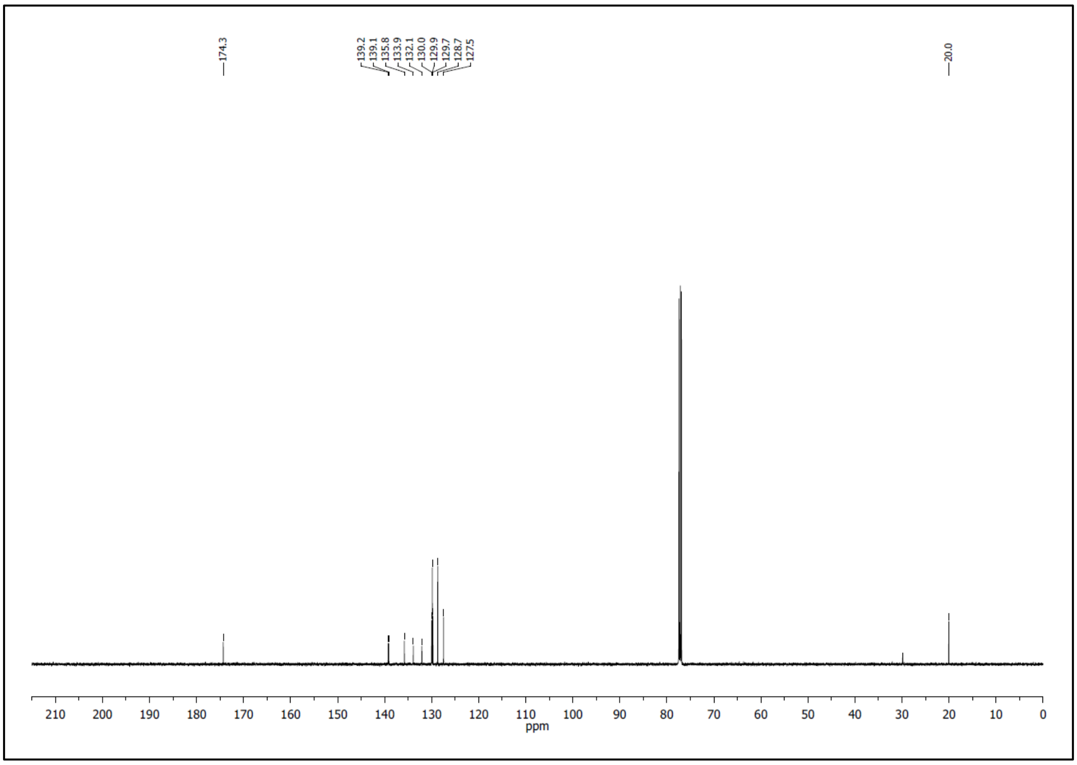

4'-bromo-3-methyl-[1,1'-biphenyl]-2-carboxylic acid (**3ak**)

^{1}H NMR ($CDCl_3$)

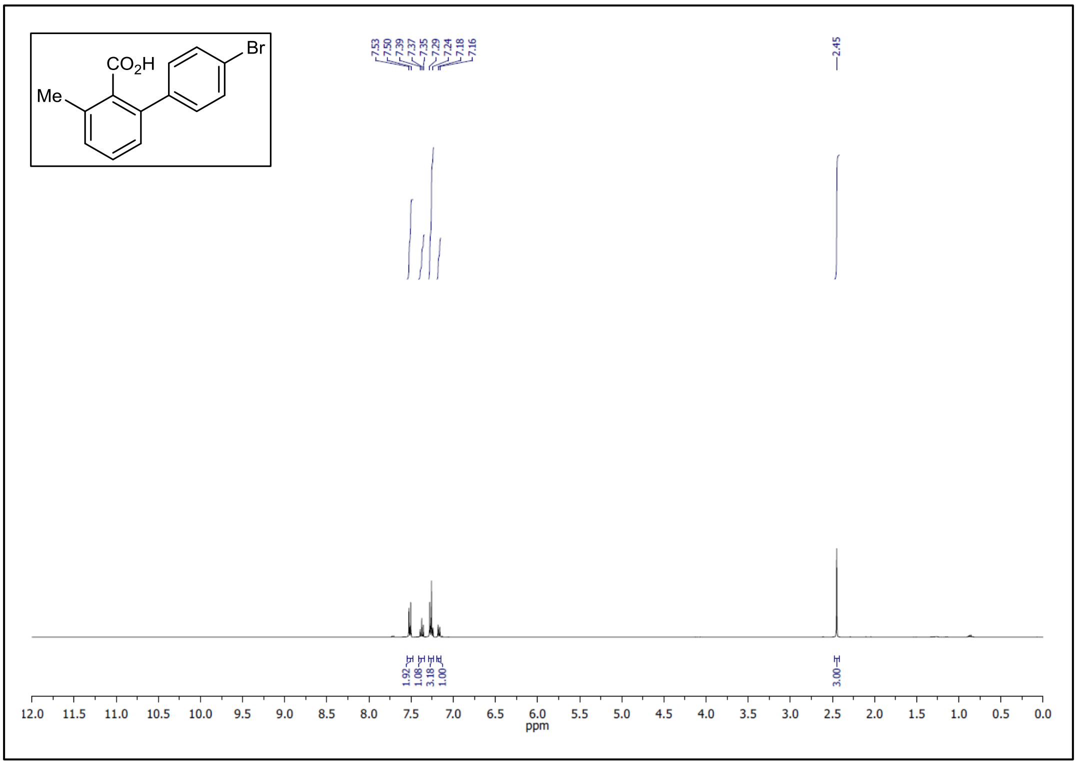

^{13}C NMR ($CDCl_3$)

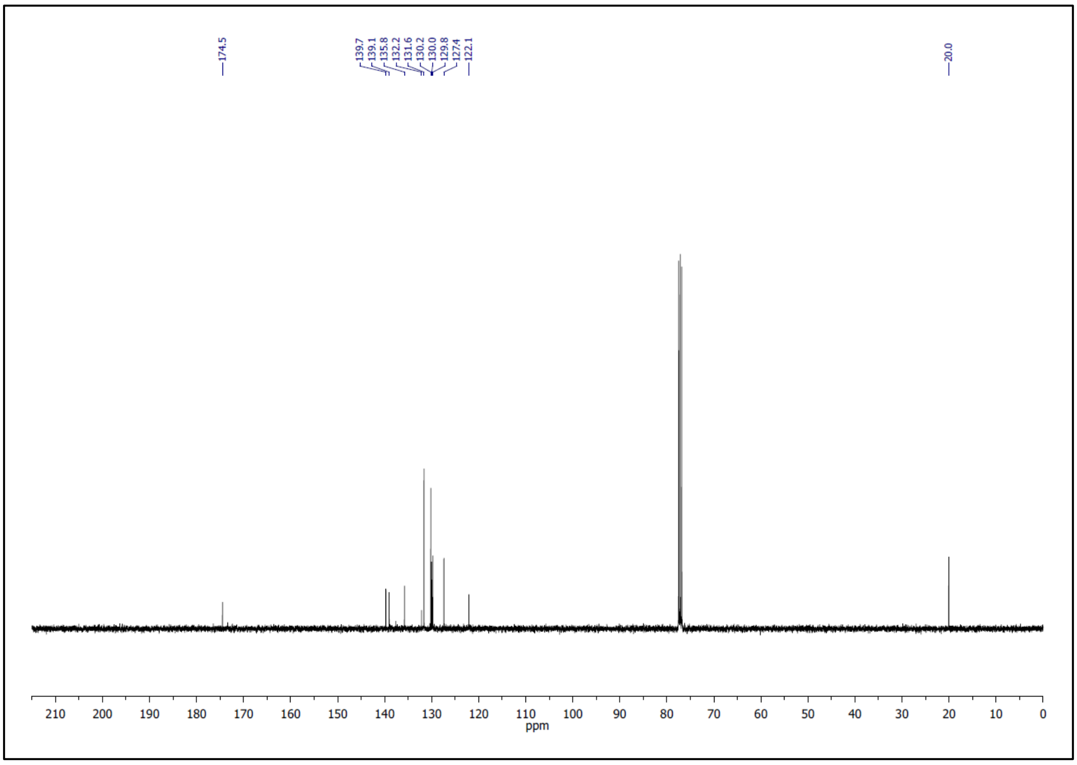

4'-iodo-3-methyl-[1,1'-biphenyl]-2-carboxylic acid (**3al**)

^{1}H NMR ((CD$_3$)$_3$CO)

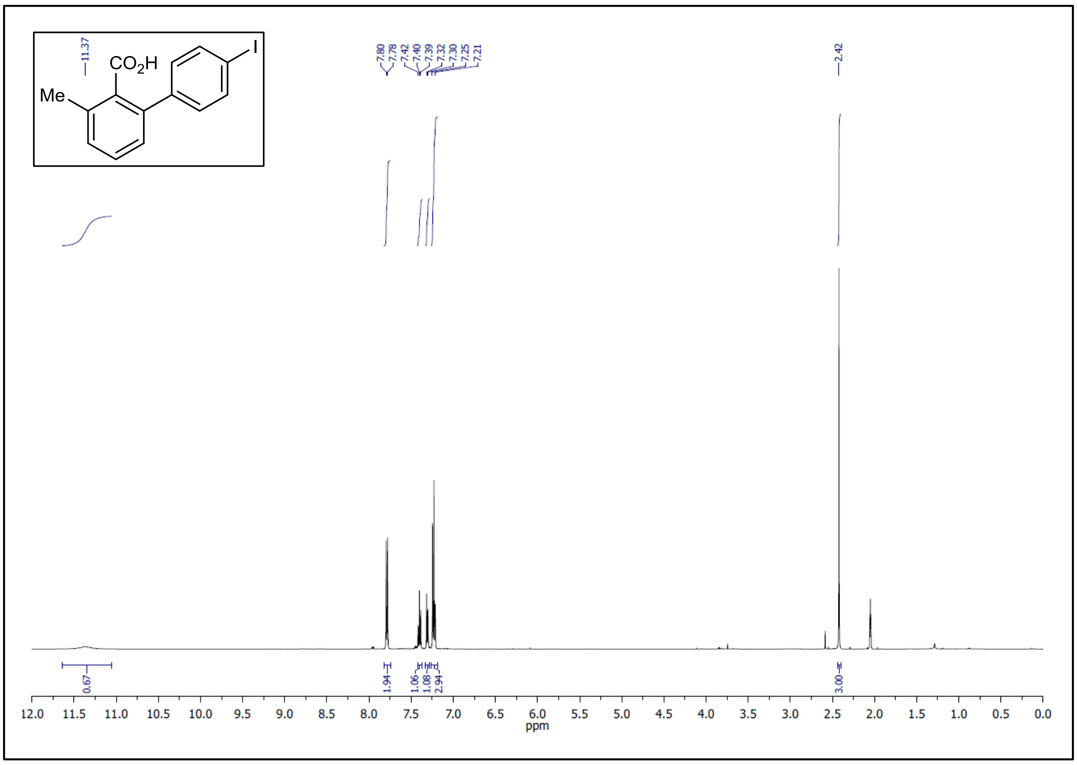

^{13}C NMR ((CD$_3$)$_3$CO)

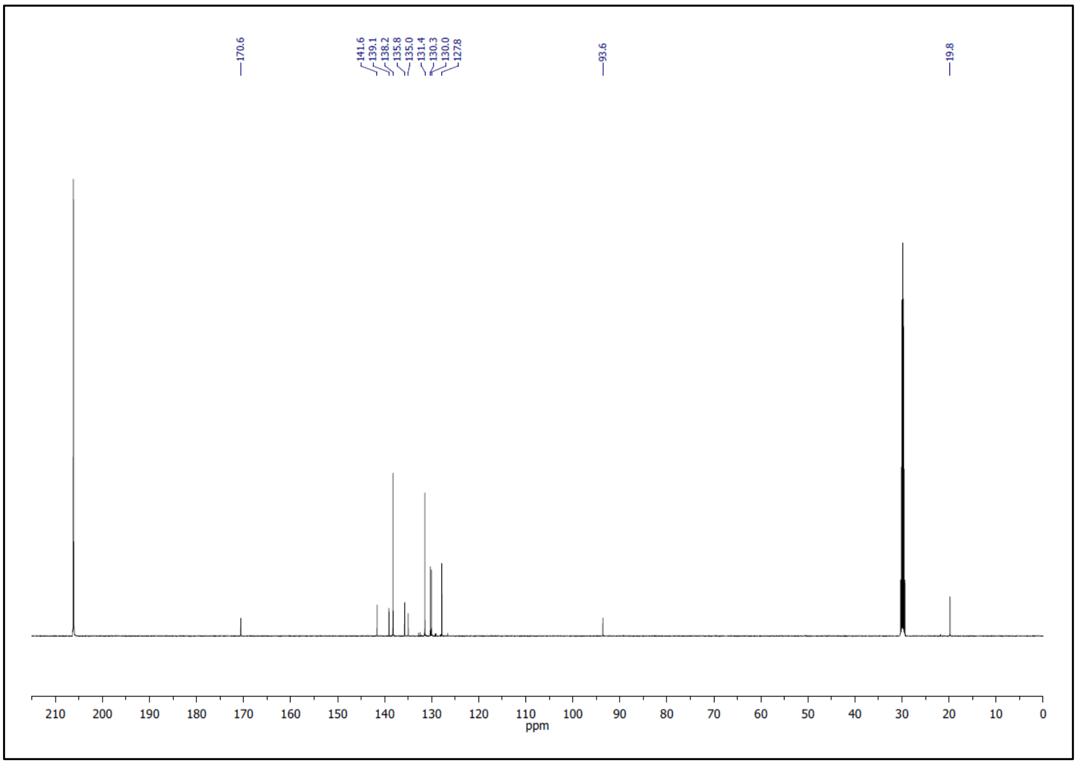

3,3''-dimethyl-[1,1':4',1''-terphenyl]-2,2''-dicarboxylic acid (**3al'**)

^{1}H NMR ($(CD_3)_2SO$)

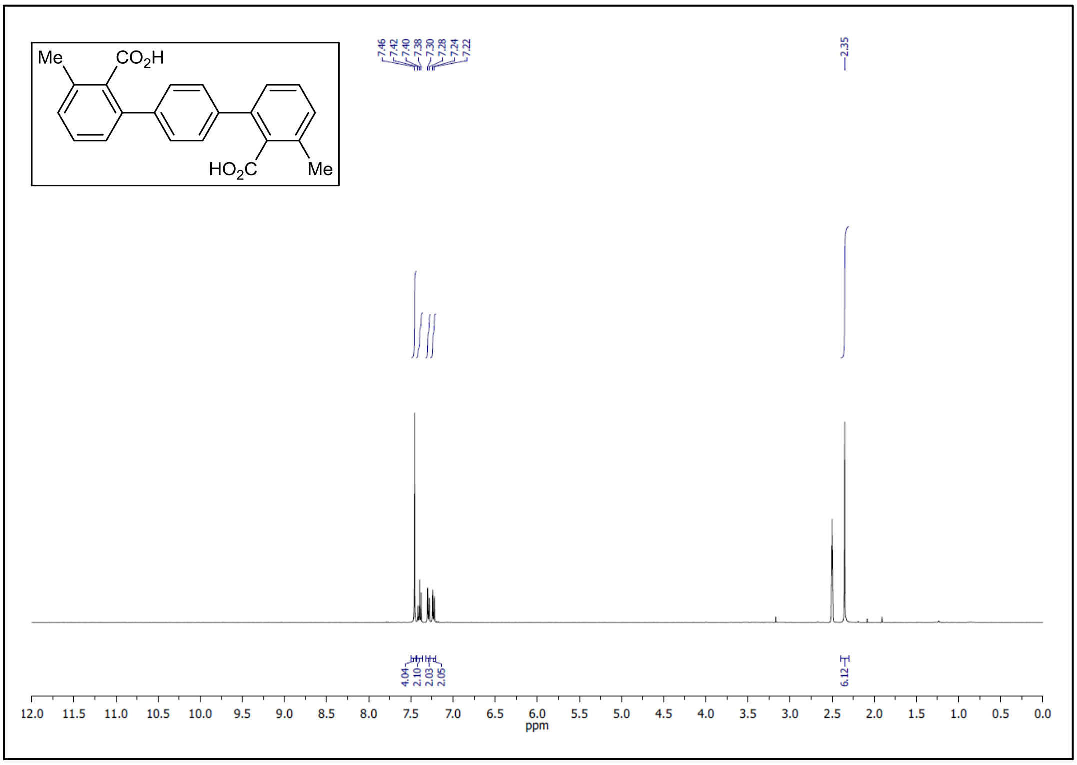

^{13}C NMR ($(CD_3)_2SO$)

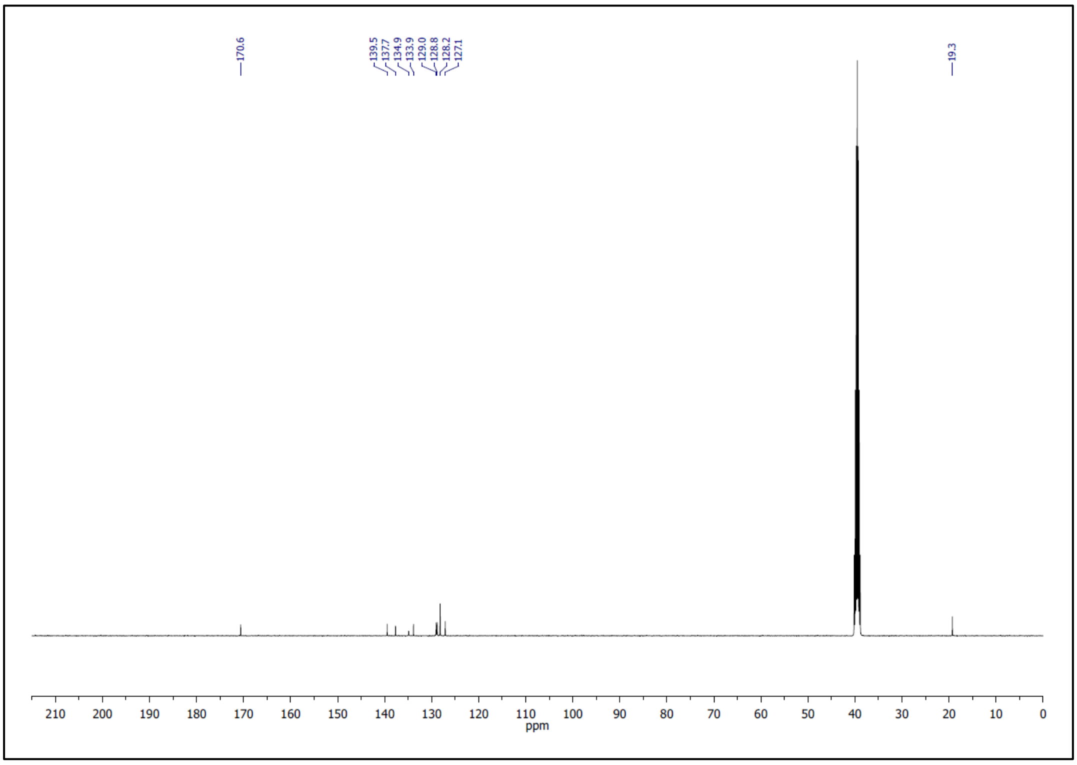

2'-fluoro-3-methyl-[1,1'-biphenyl]-2-carboxylic acid (**3am**)

^{1}H NMR ($CDCl_3$)

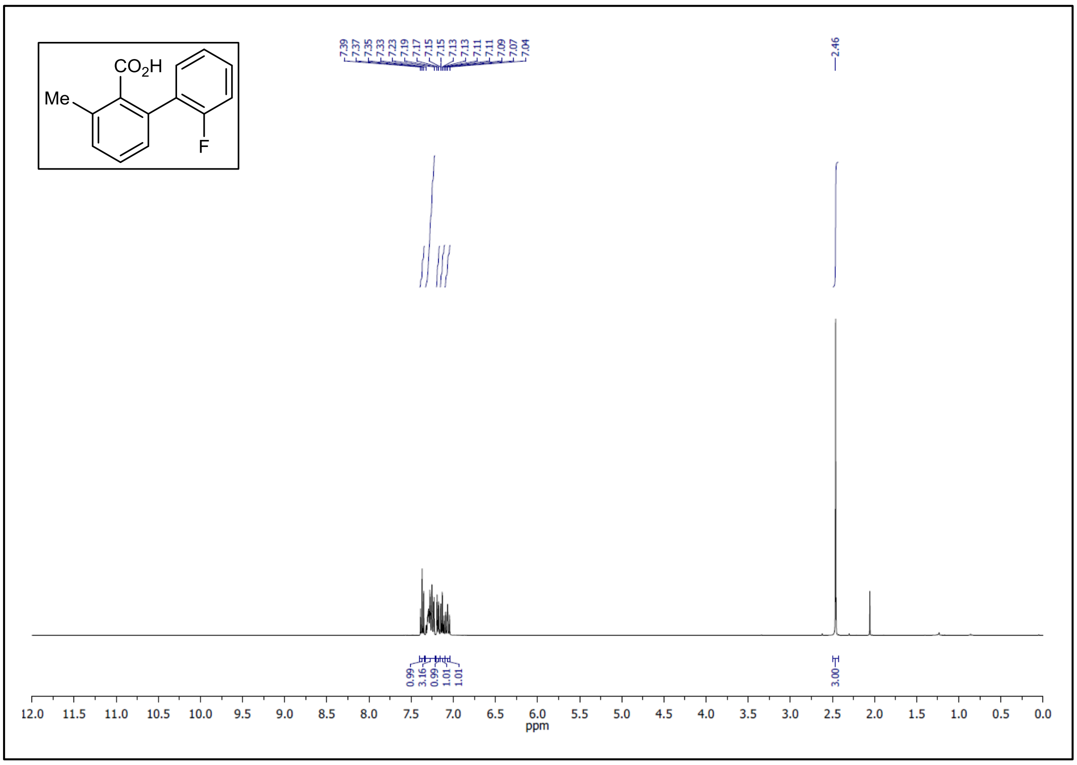

13C NMR (CDCl3)

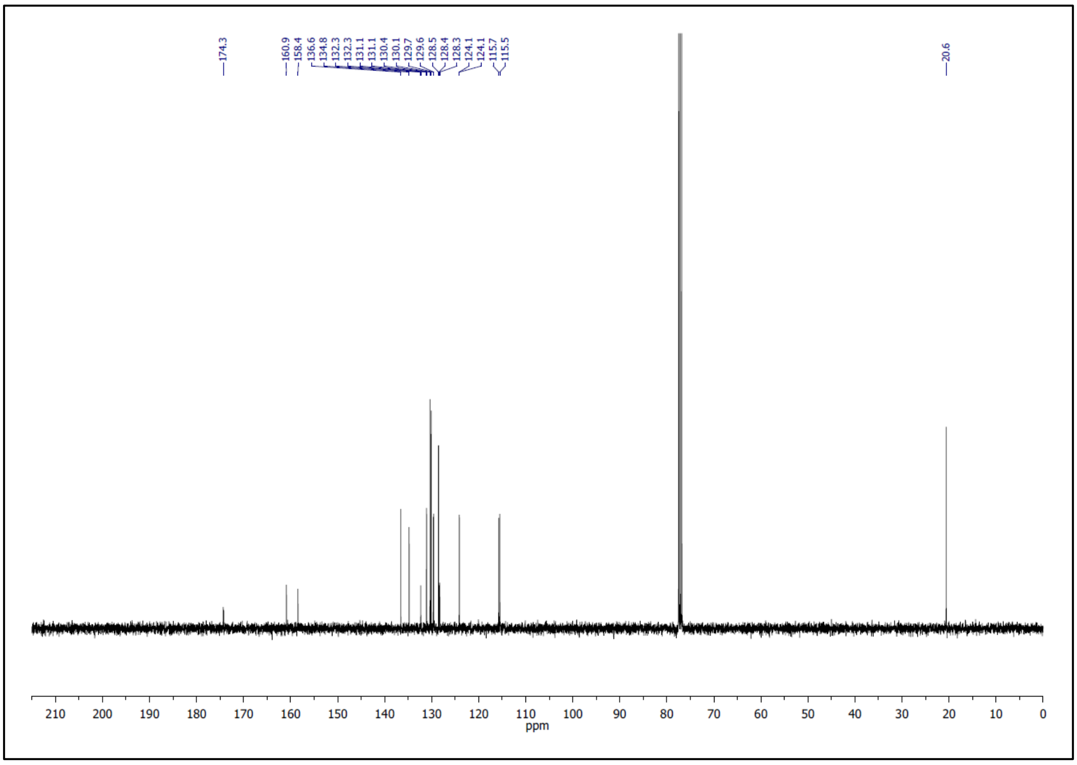

^{19}F NMR ($CDCl_3$)

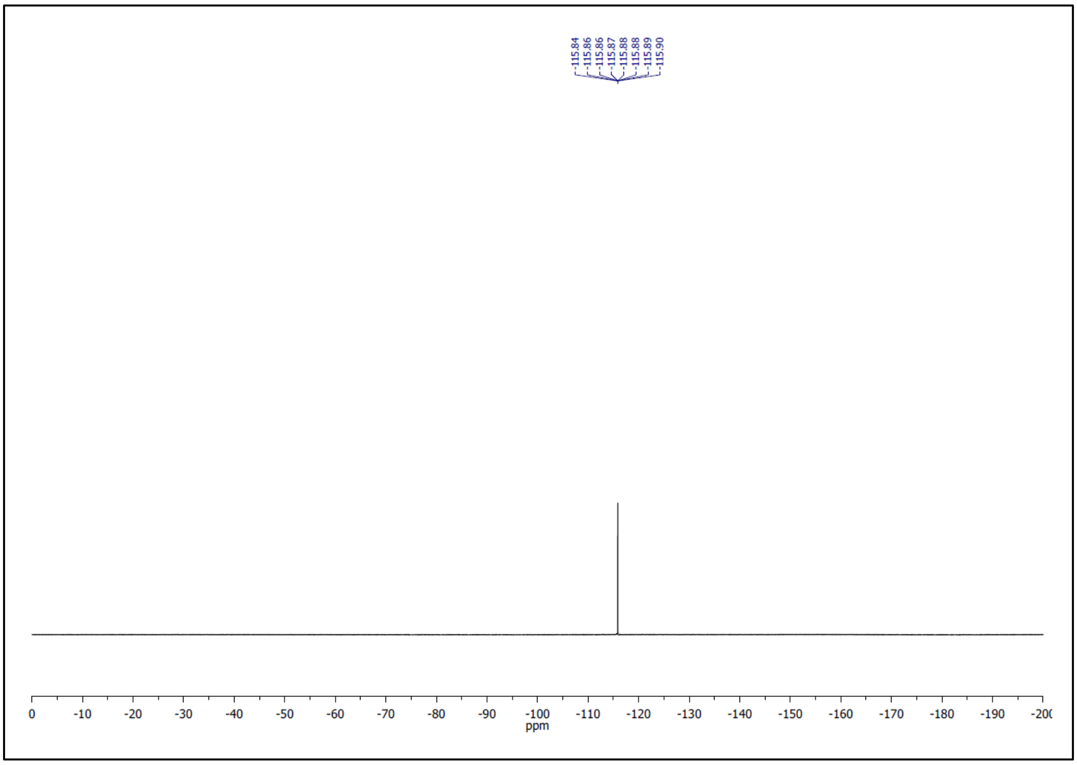

3'-chloro-3-methyl-[1,1'-biphenyl]-2-carboxylic acid (**3an**)

^{1}H NMR (CD_3CN)

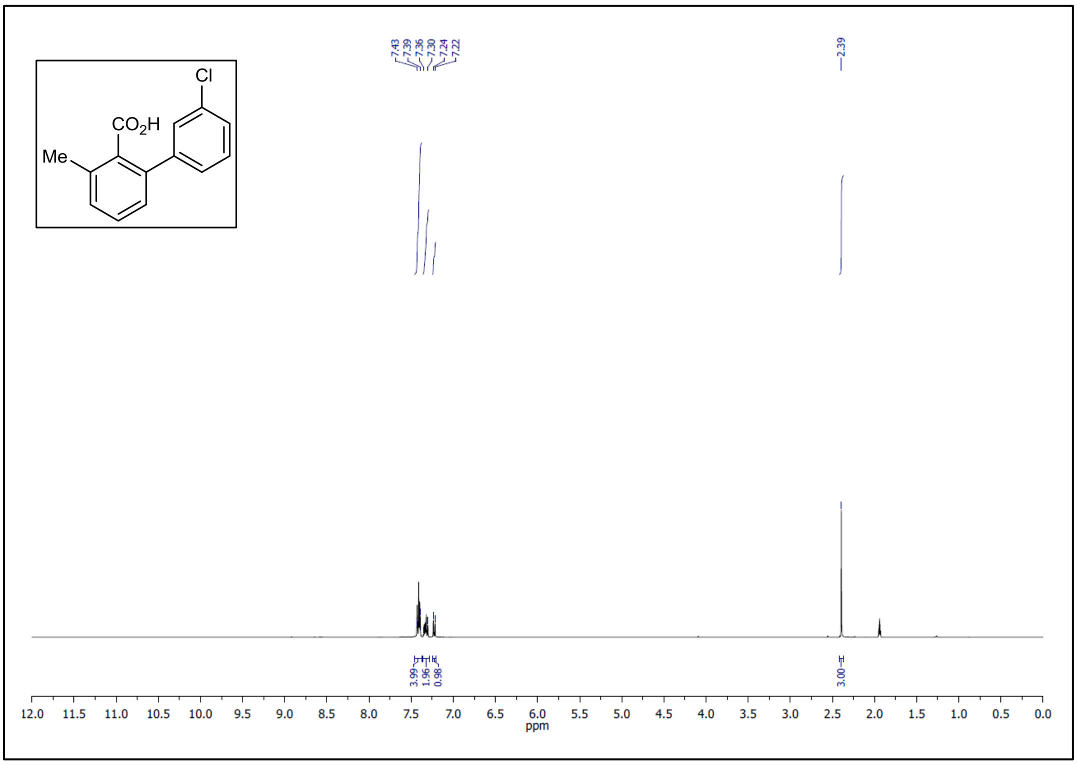

^{13}C NMR ($(CD_3)_2CO$)

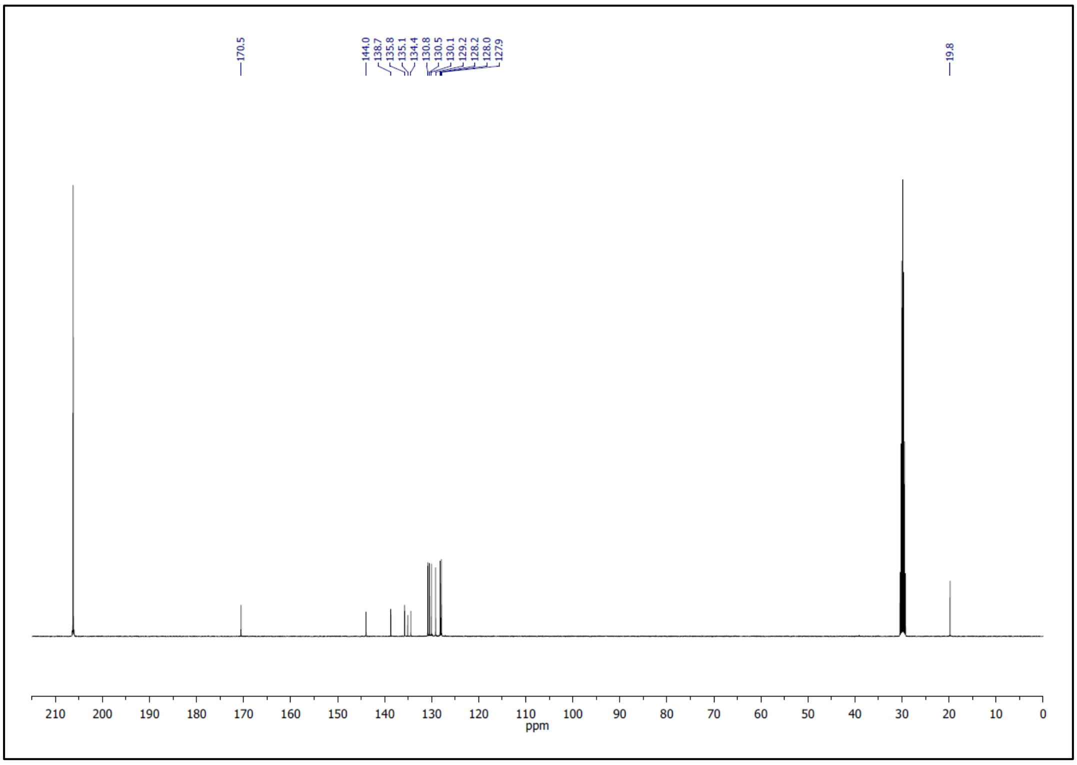

2-methyl-6-[4-(trifluoromethyl)phenyl]benzoic acid (**3ao**)

^{1}H NMR ($CDCl_3$)

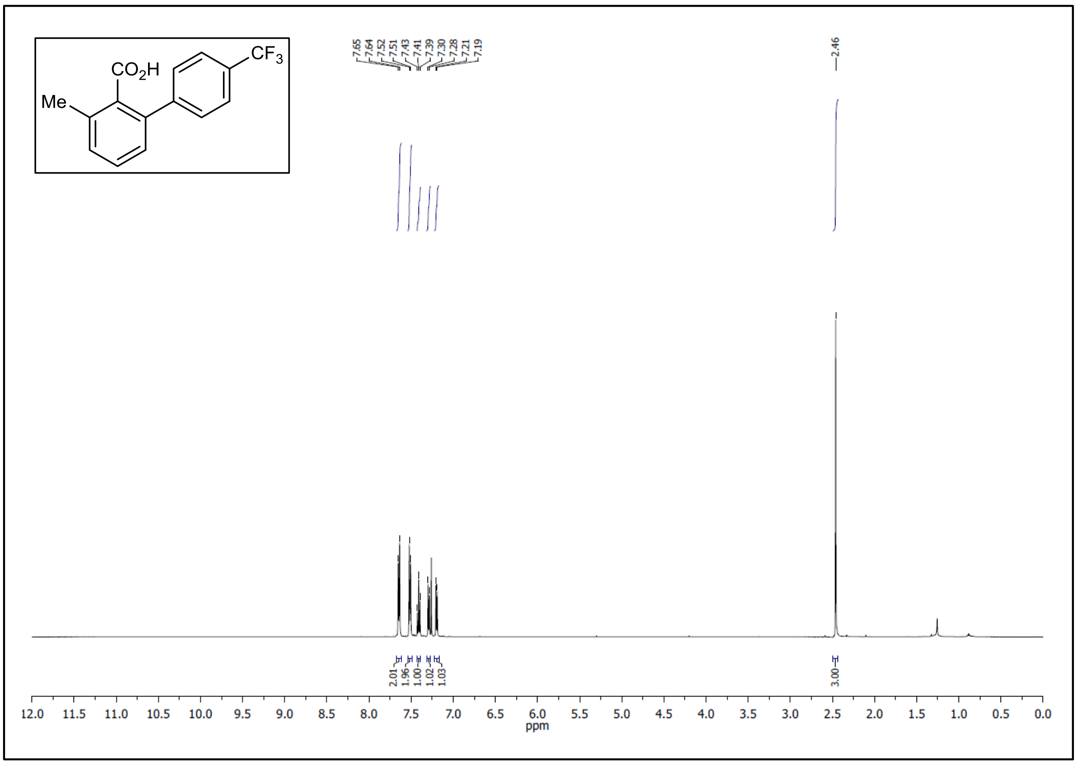

^{13}C NMR ($CDCl_3$)

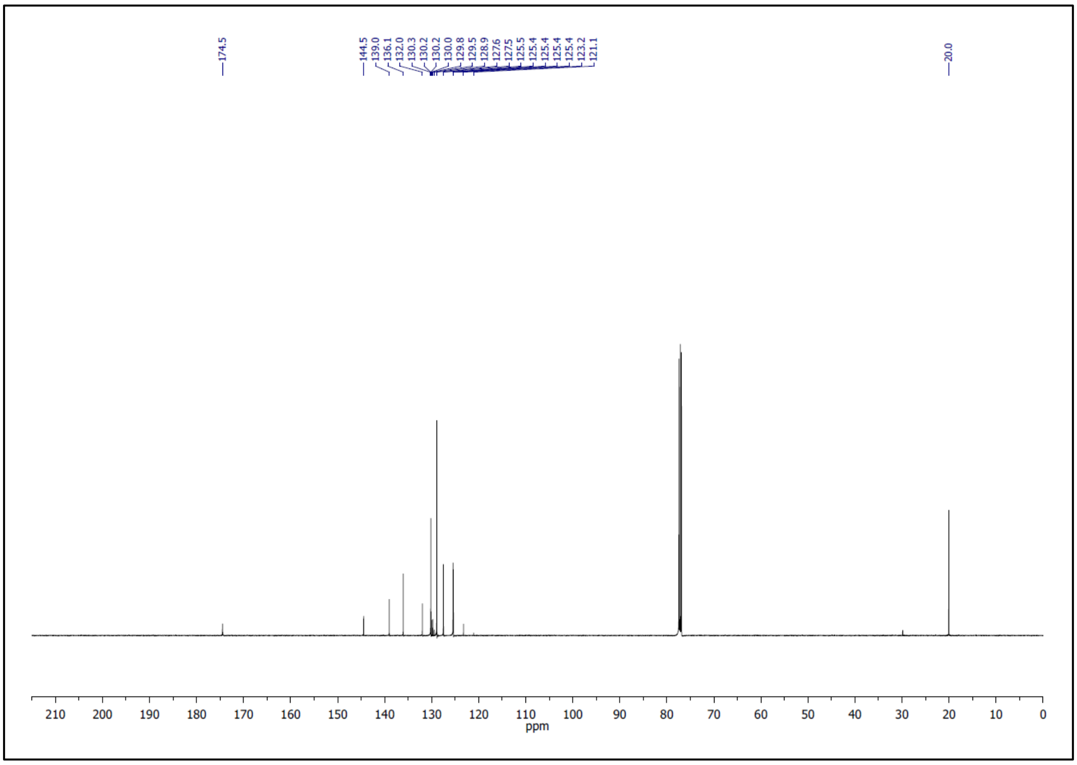

^{19}F NMR ($CDCl_3$)

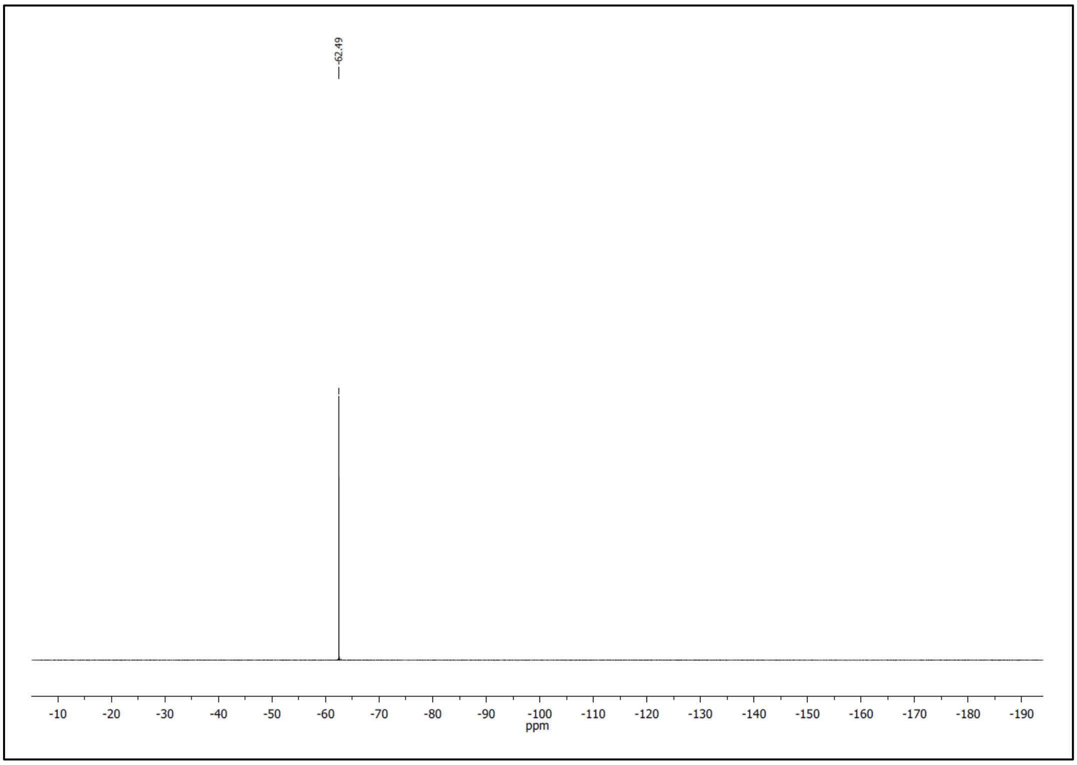

3-methyl-3'-(trifluoromethyl)-[1,1'-biphenyl]-2-carboxylic acid (**3ap**)

^{1}H NMR ($(CD_3)_3CO$)

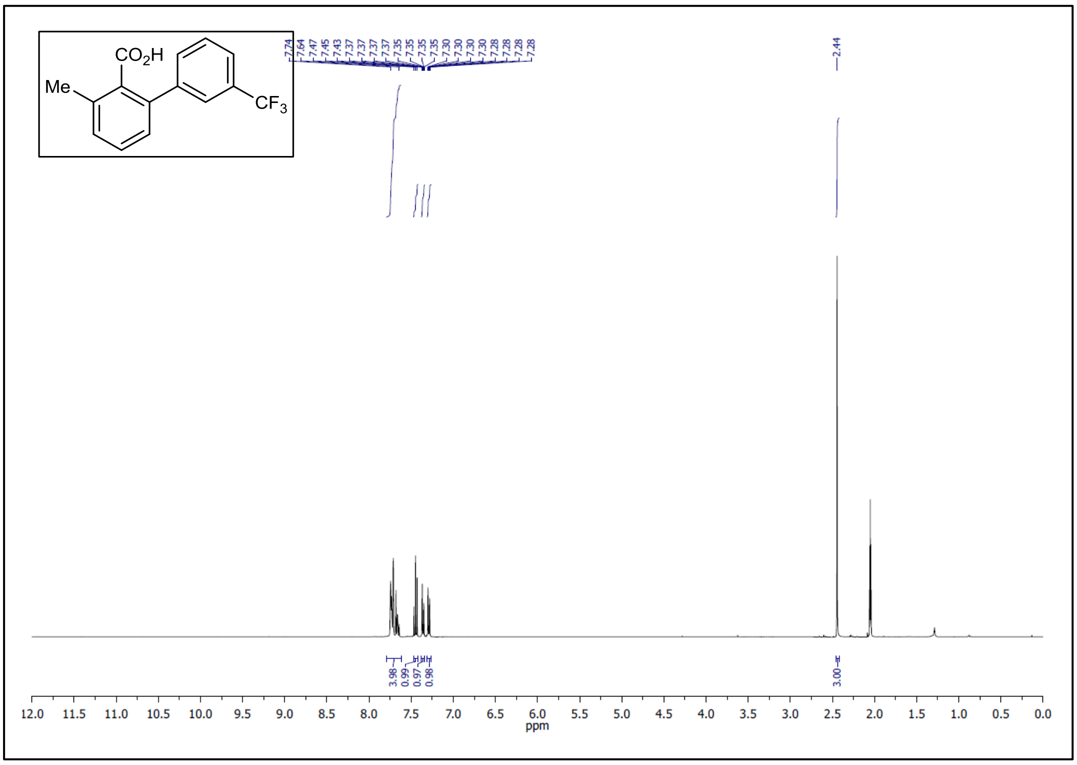

^{13}C NMR ($(CD_3)_3CO$)

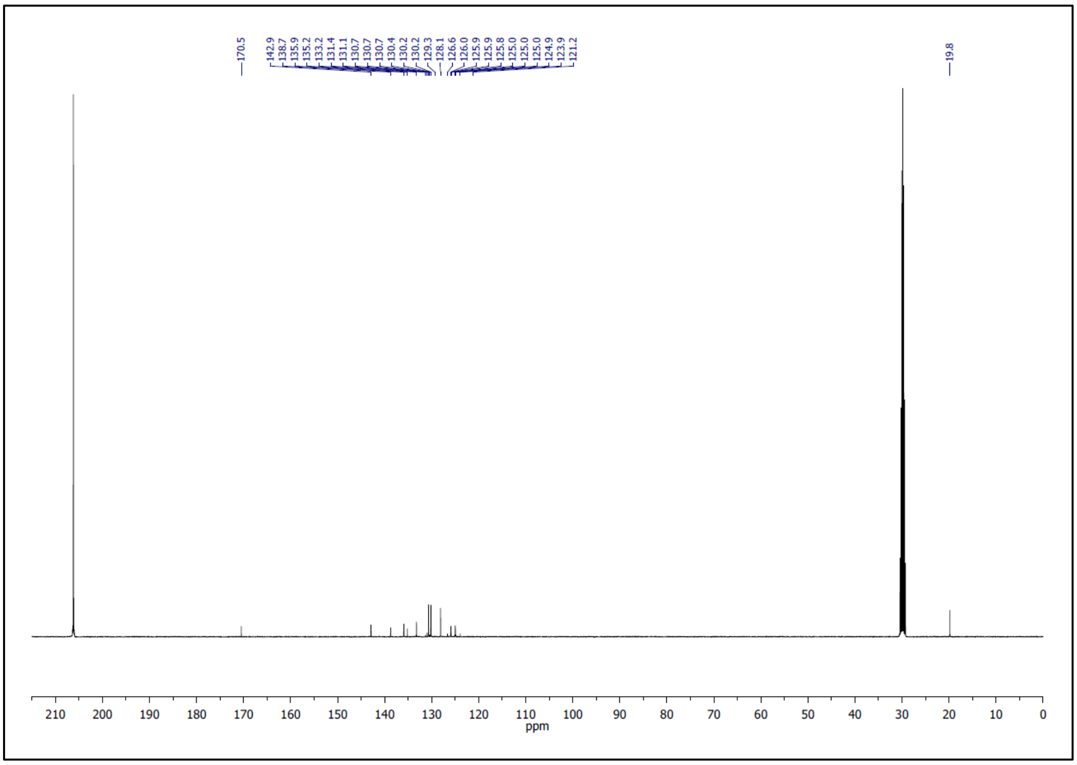

^{19}F NMR ($(CD_3)_3CO$)

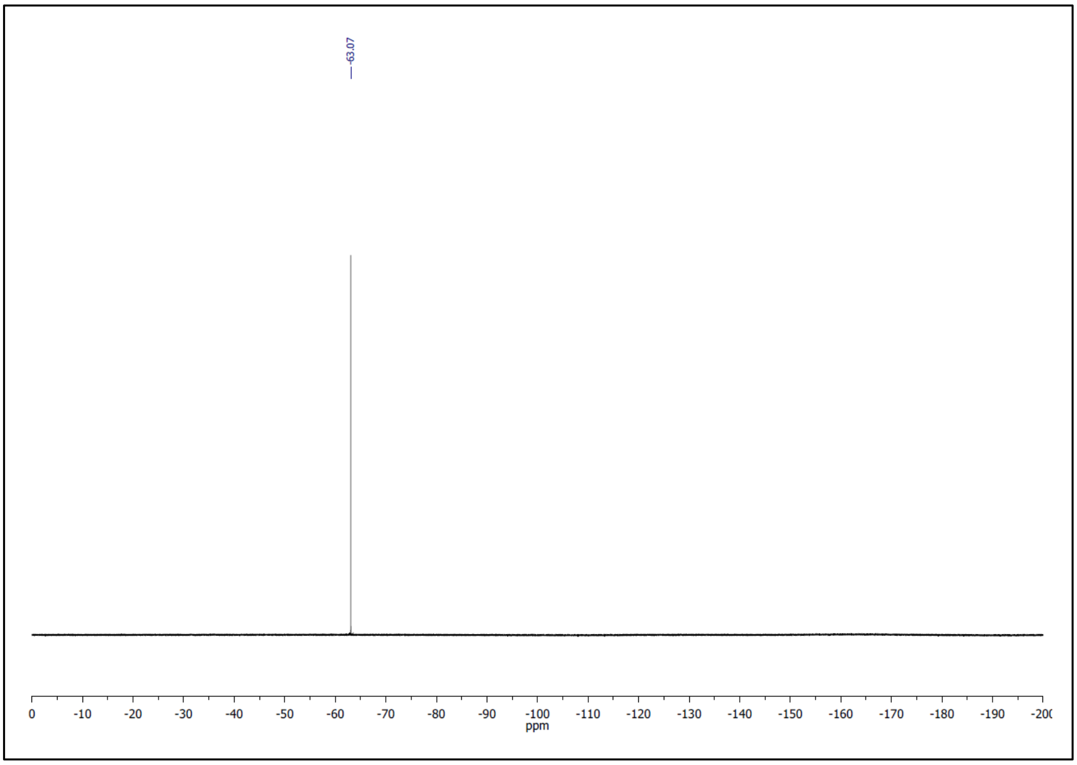

4'-acetyl-3-methyl-[1,1'-biphenyl]-2-carboxylic acid (**3aq**

^{1}H NMR ($CDCl_3$)

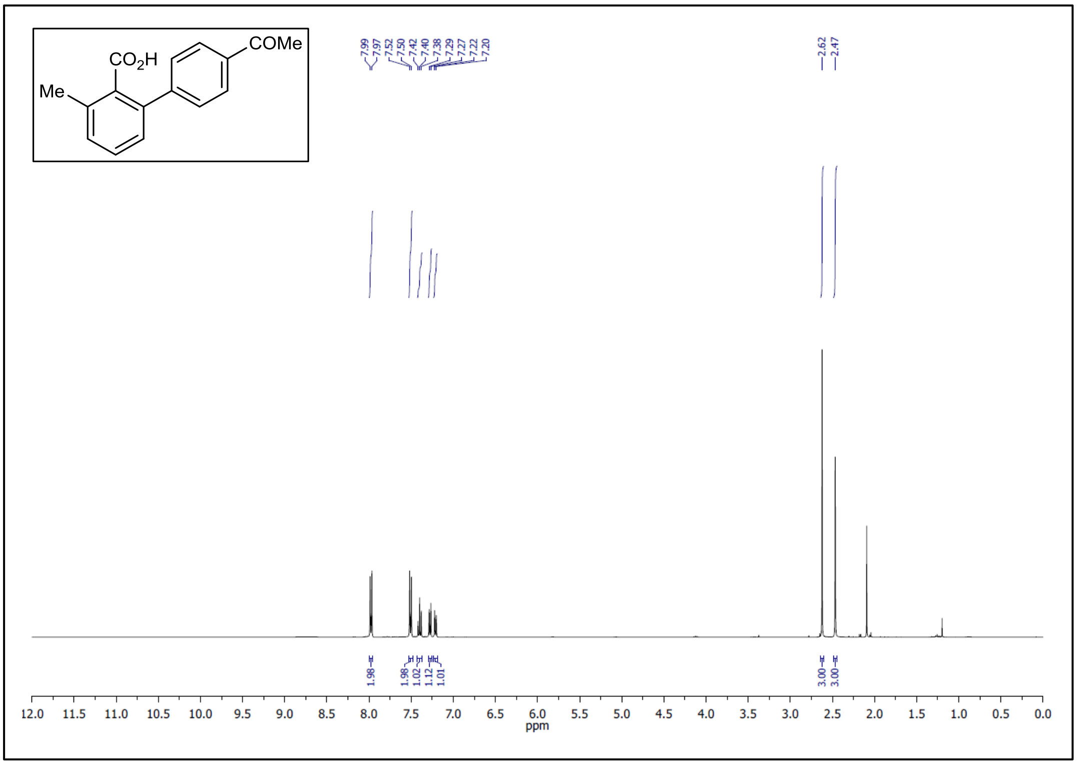

^{13}C NMR ($CDCl_3$)

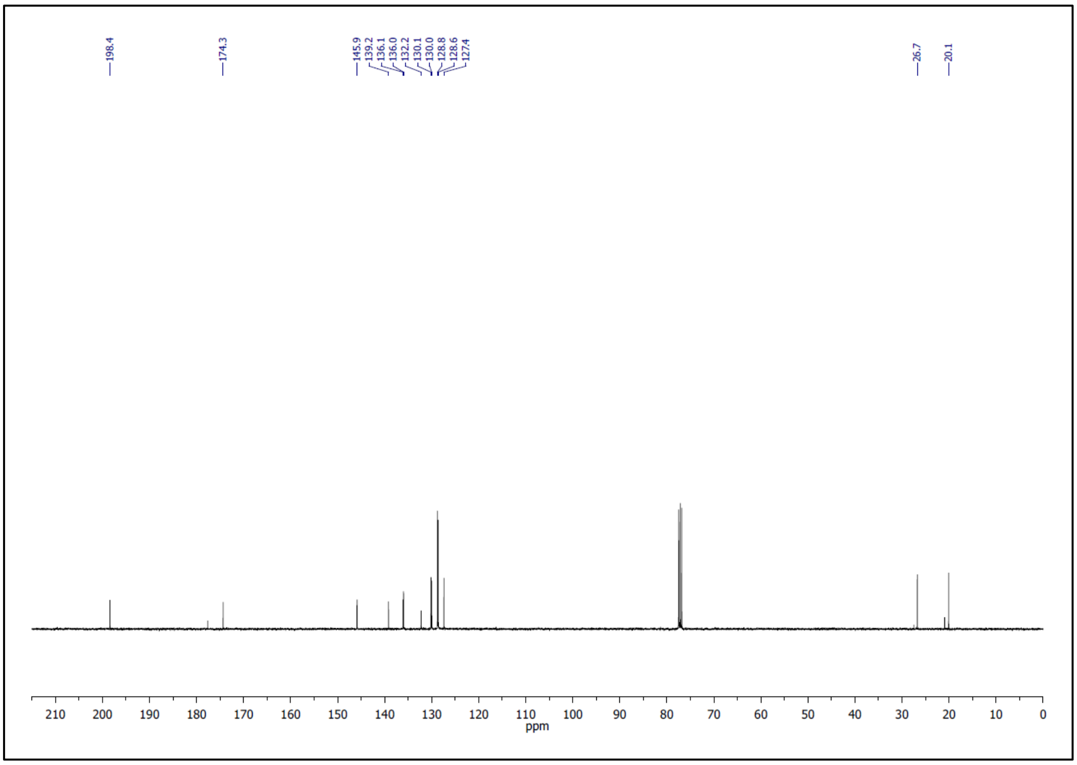

3'-acetyl-3-methyl-[1,1'-biphenyl]-2-carboxylic acid (**3ar**)

^{1}H NMR ($CDCl_3$)

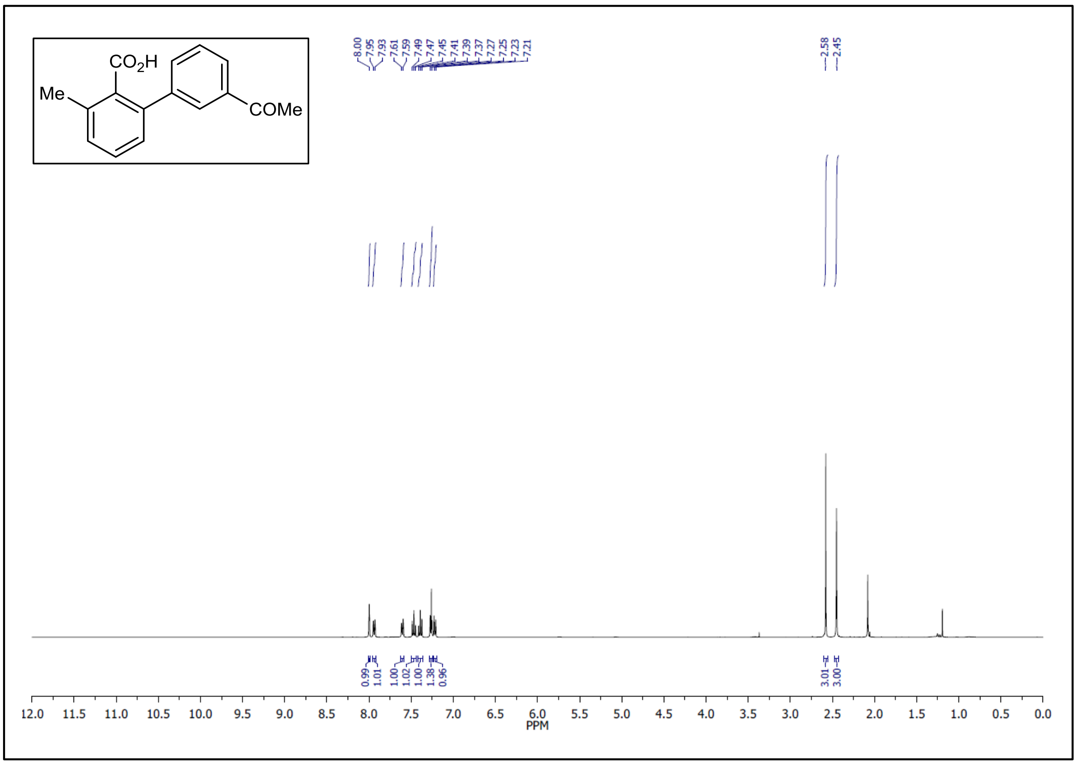

^{13}C NMR ($CDCl_3$)

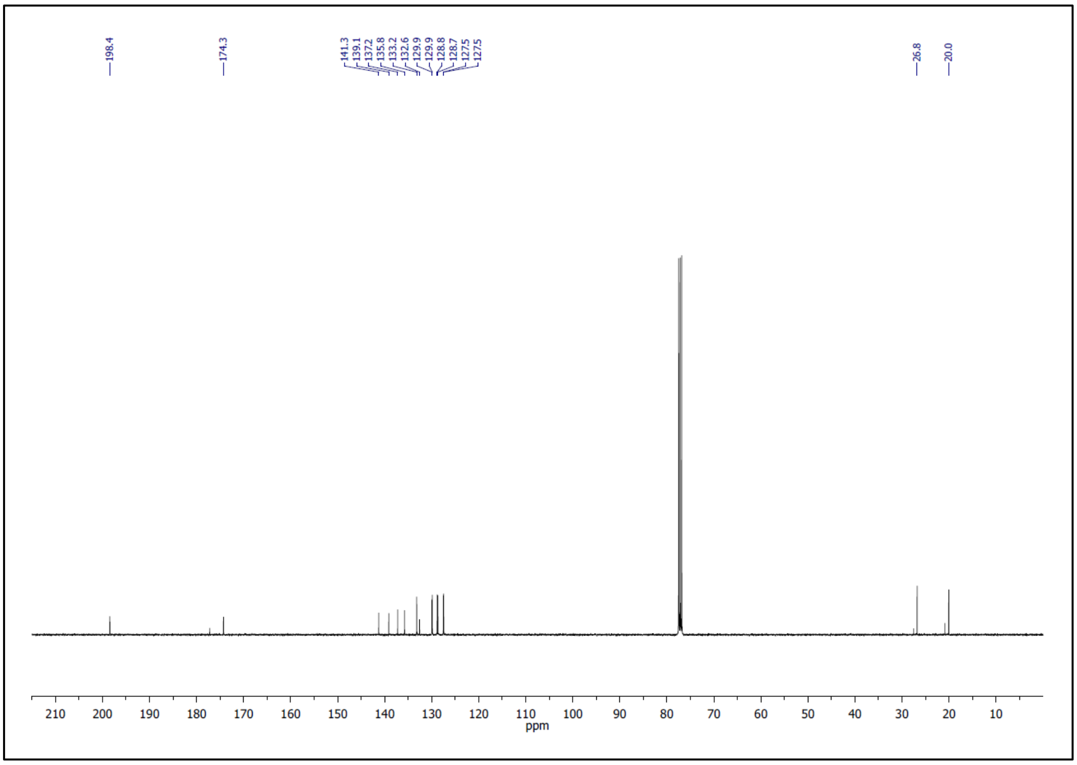

2-[4-(methoxycarbonyl)phenyl]-6-methylbenzoic acid (**3as**)

^{1}H NMR ($CDCl_3$)

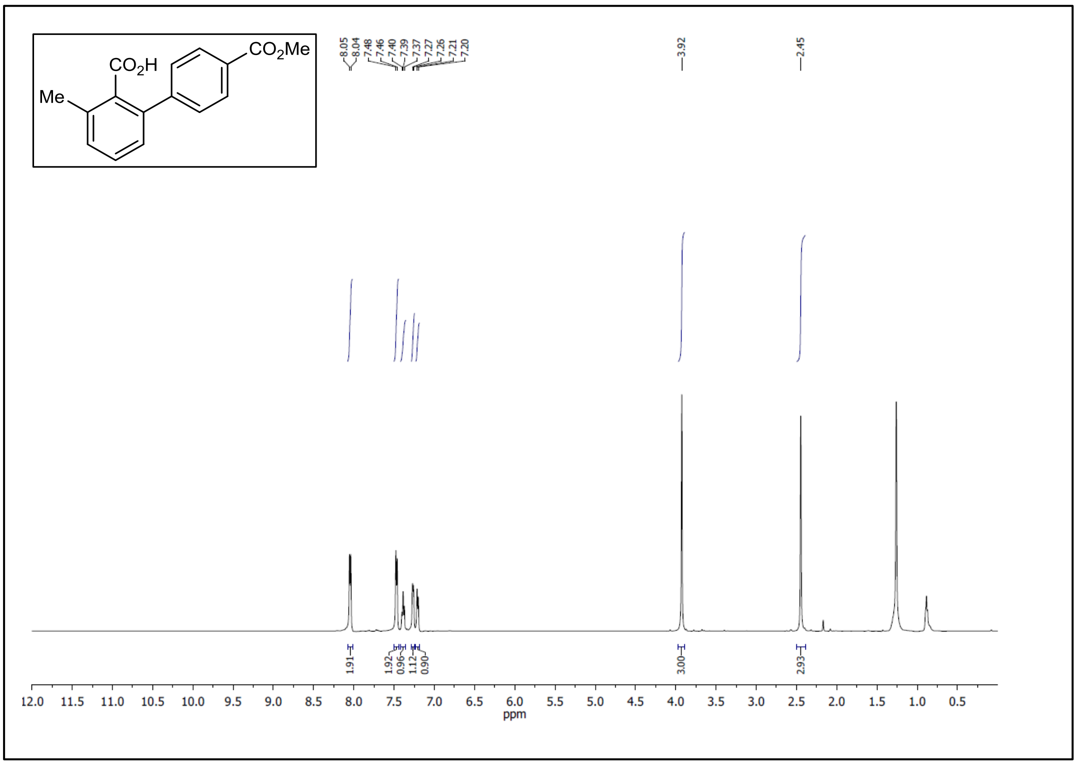

^{13}C NMR ($CDCl_3$)

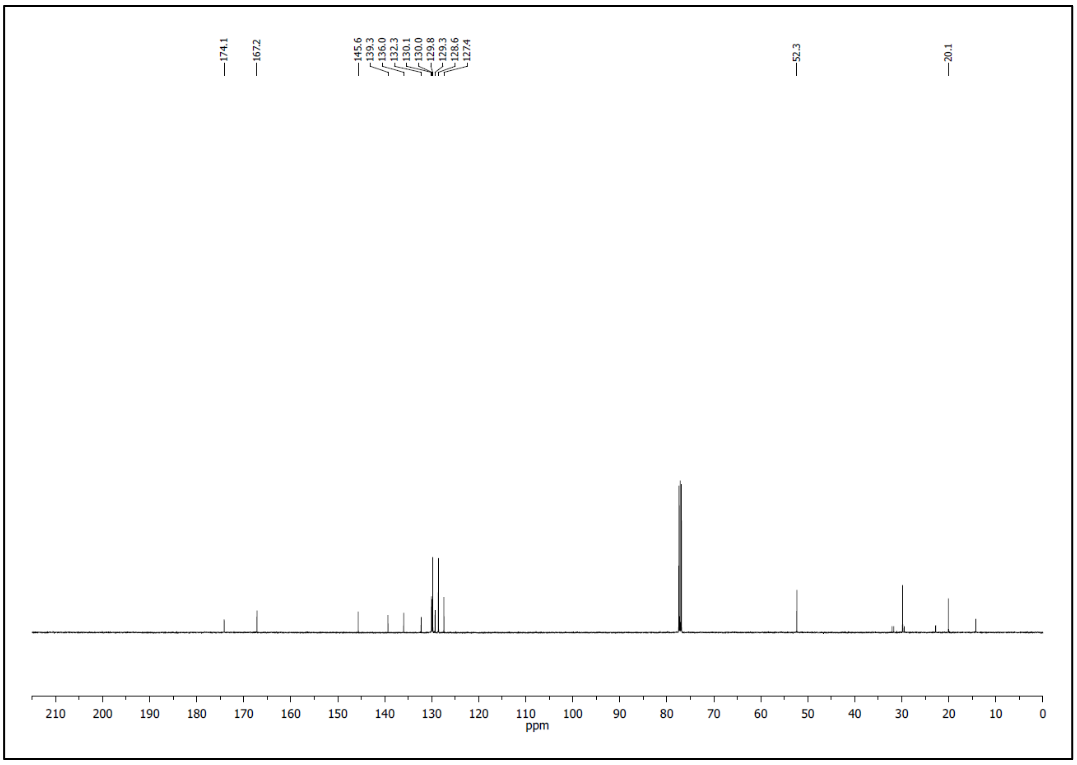

3-methyl-4'-(methylthio)-[1,1'-biphenyl]-2-carboxylic acid (**3at**)

^{1}H NMR ($(CD_3)_2CO$)

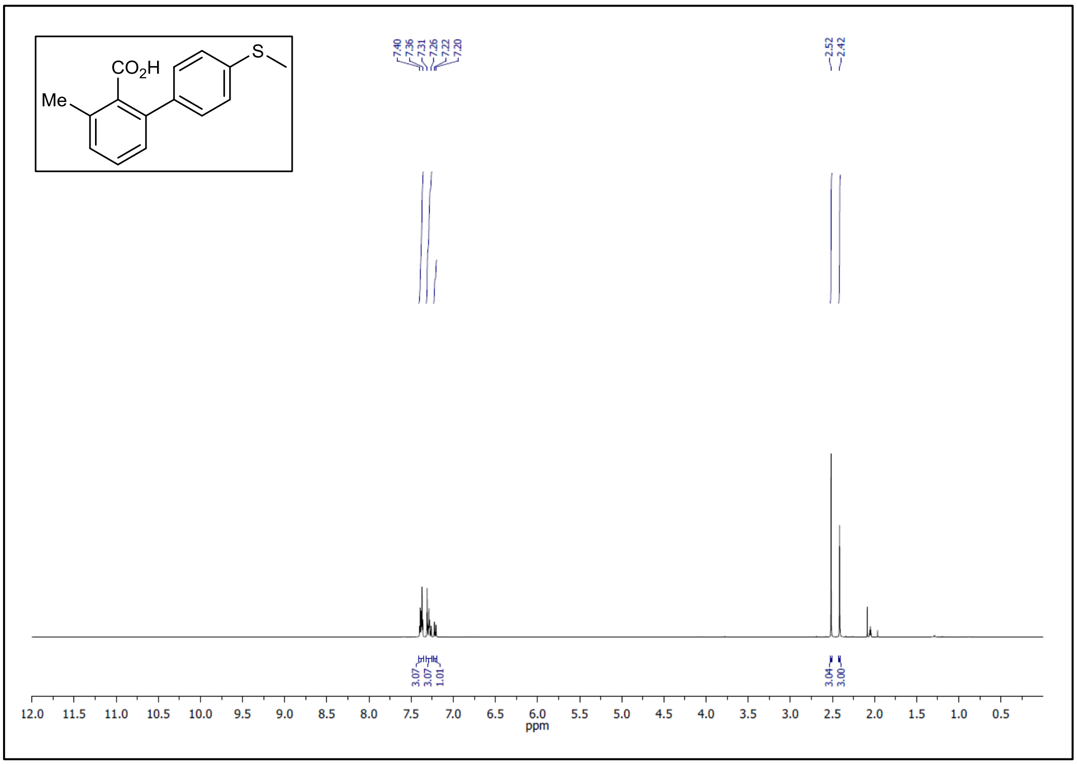

^{13}C NMR ($(CD_3)_2CO$)

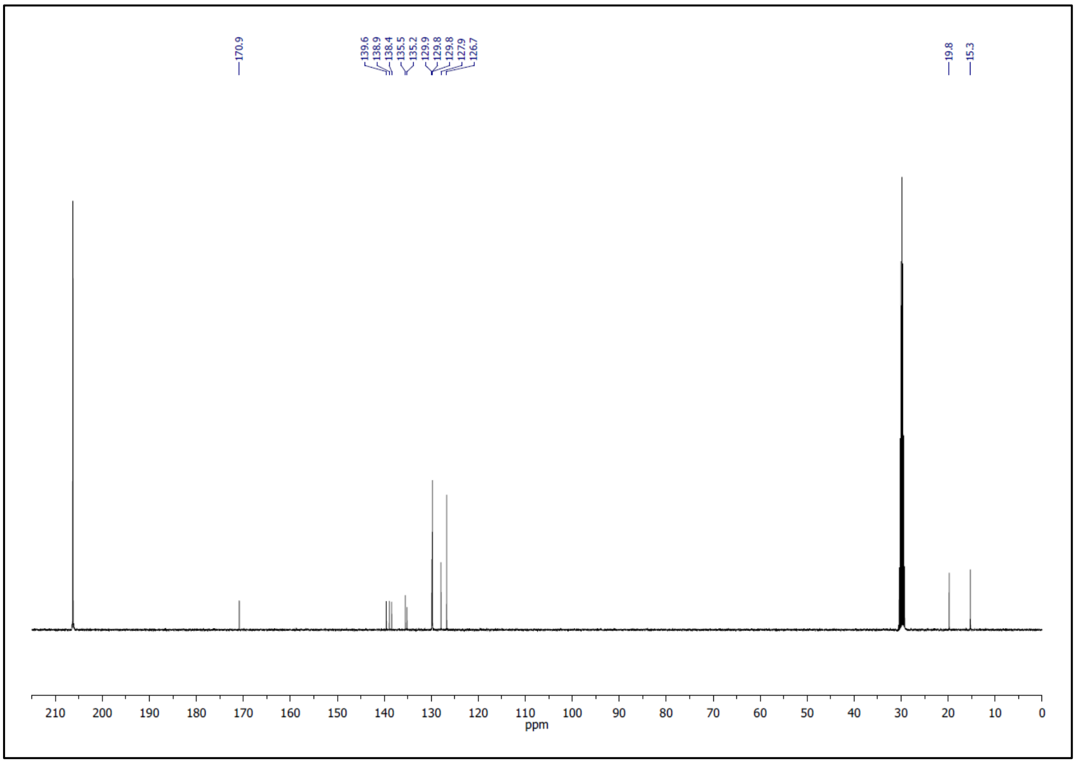

2-methyl-6-(naphthalen-1-yl)benzoic acid (**3au**)

^{1}H NMR ($(CD_3)_2CO$)

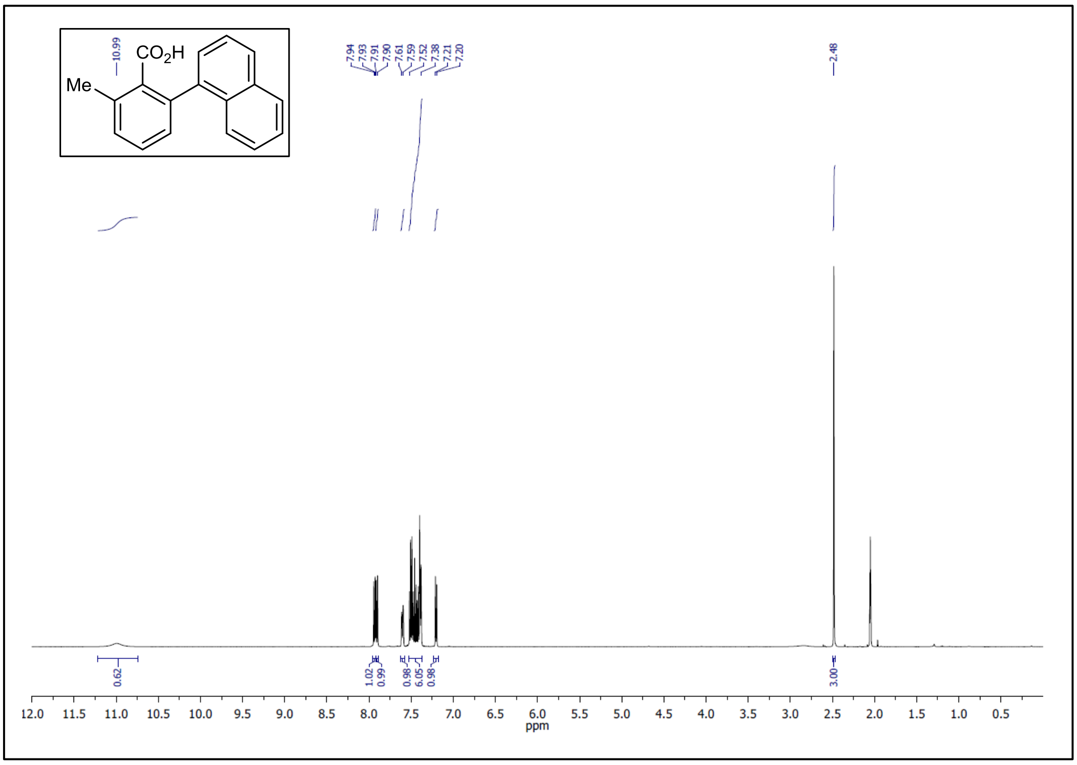

^{13}C NMR ($(CD_3)_2CO$)

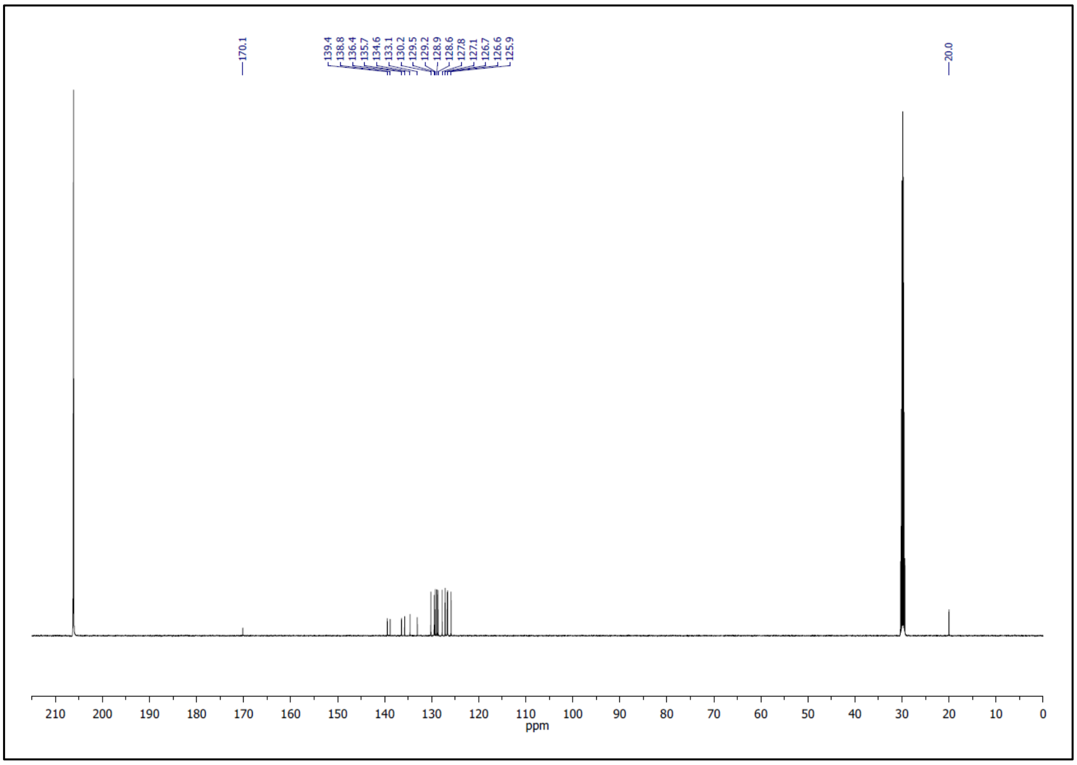

4'-cyano-3-methyl-[1,1'-biphenyl]-2-carboxylic acid (**3av**)

^{1}H NMR ($(CD_3)_2CO$)

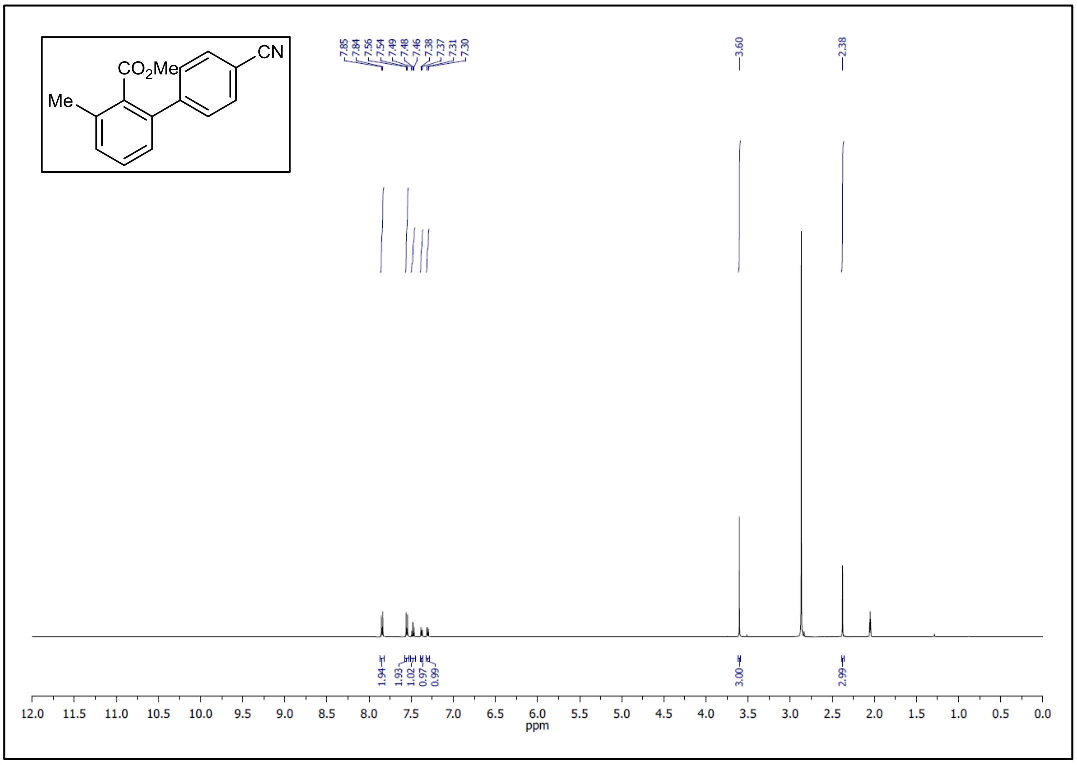

^{13}C NMR ($(CD_3)_2CO$)

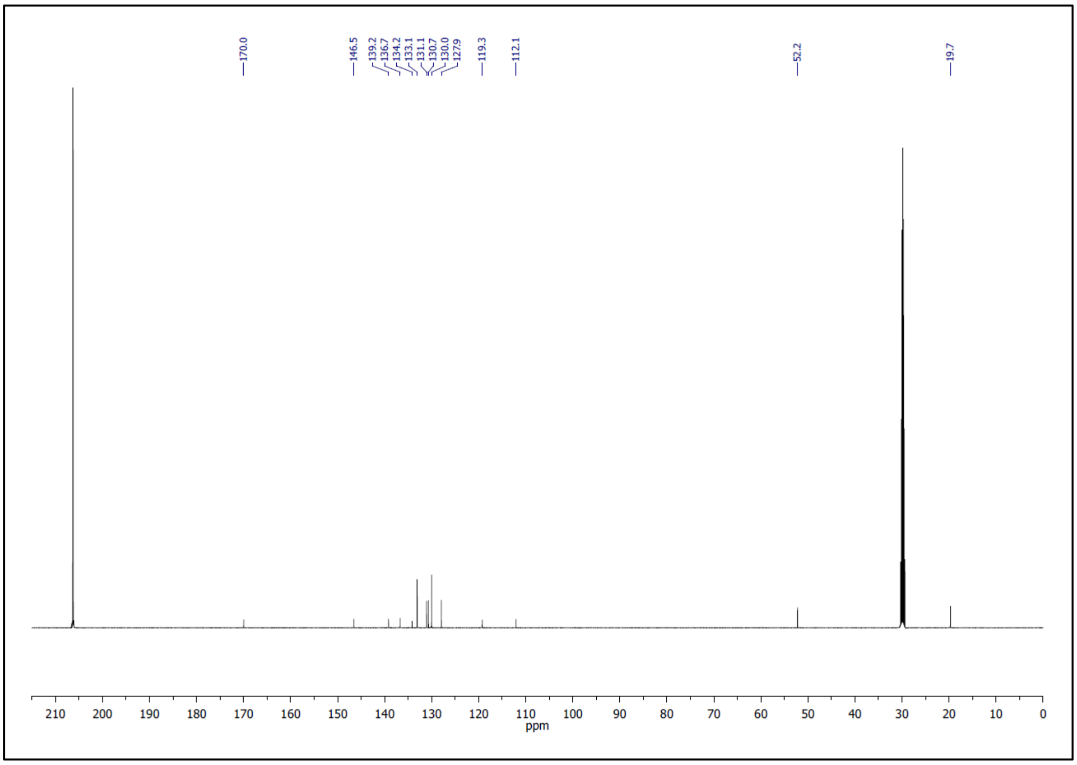

2-methyl-6-(1-methyl-1H-indol-5-yl)benzoic acid (**3aw**)

^{1}H NMR ($(CD_3)_2CO$)

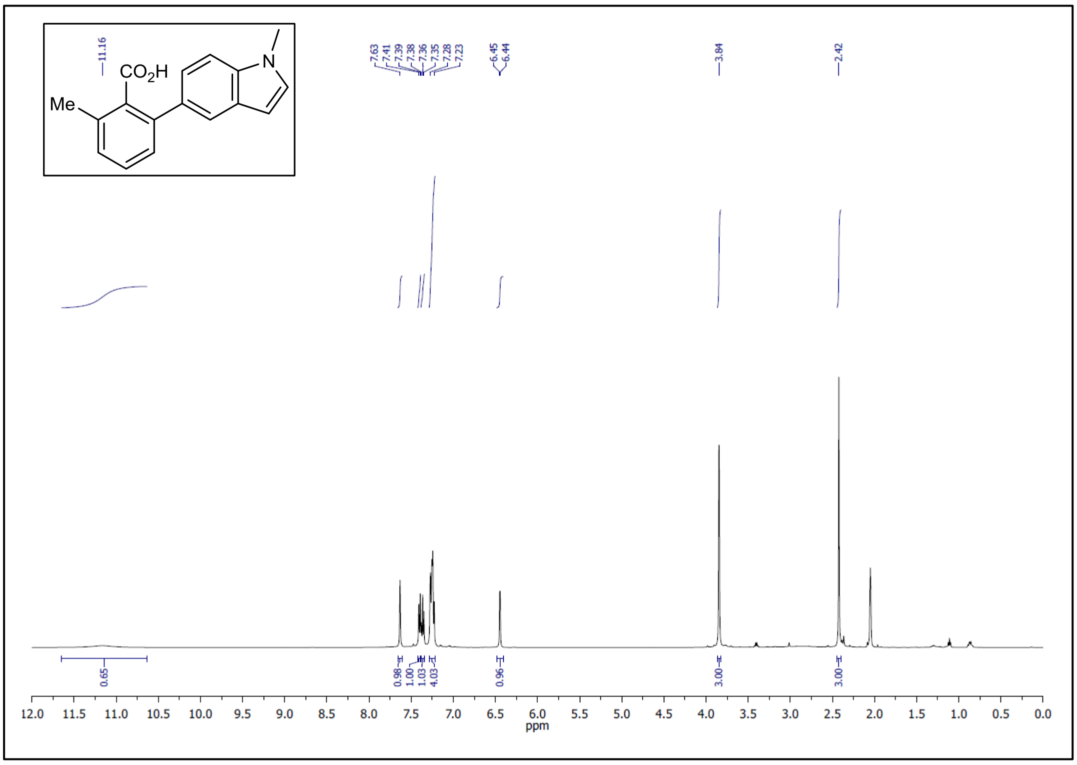

^{13}C NMR ($(CD_3)_2CO$)

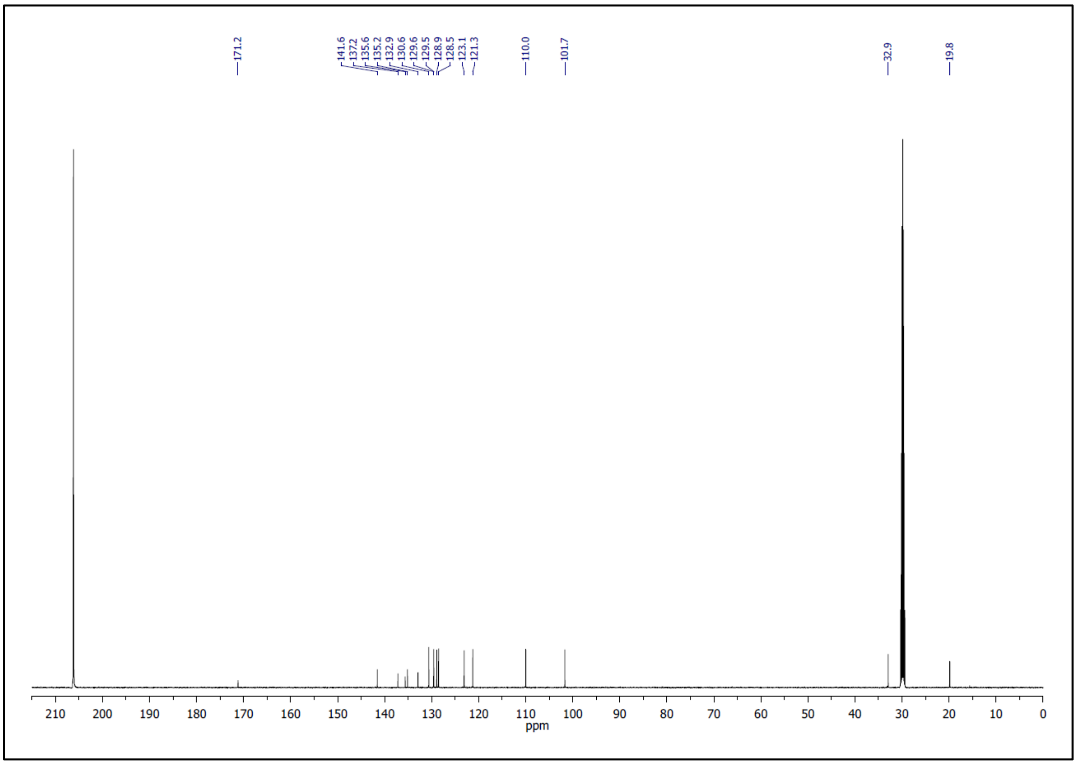

2-methyl-6-(thiophen-2-yl)benzoic acid (**3ax**)

^{1}H NMR ($(CD_3)_2CO$)

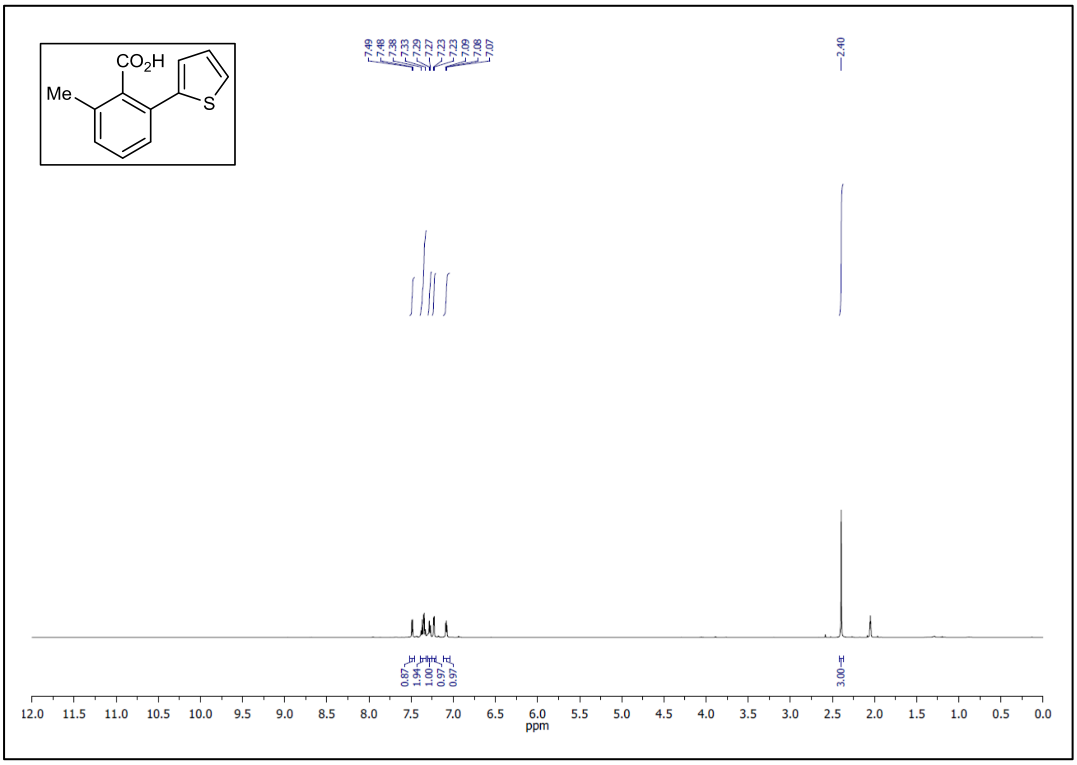

^{13}C NMR ($(CD_3)_2CO$)

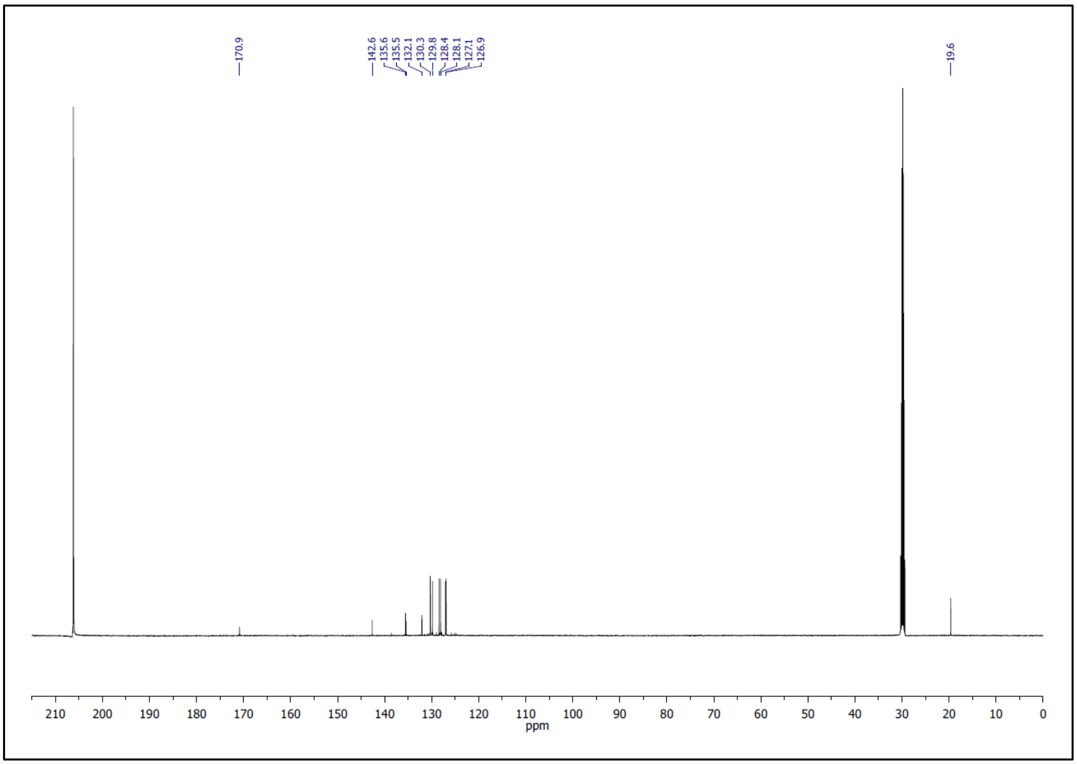

2-methoxy-6-(4-methoxyphenyl)benzoic acid (**3ba**)

^{1}H NMR ($CDCl_3$)

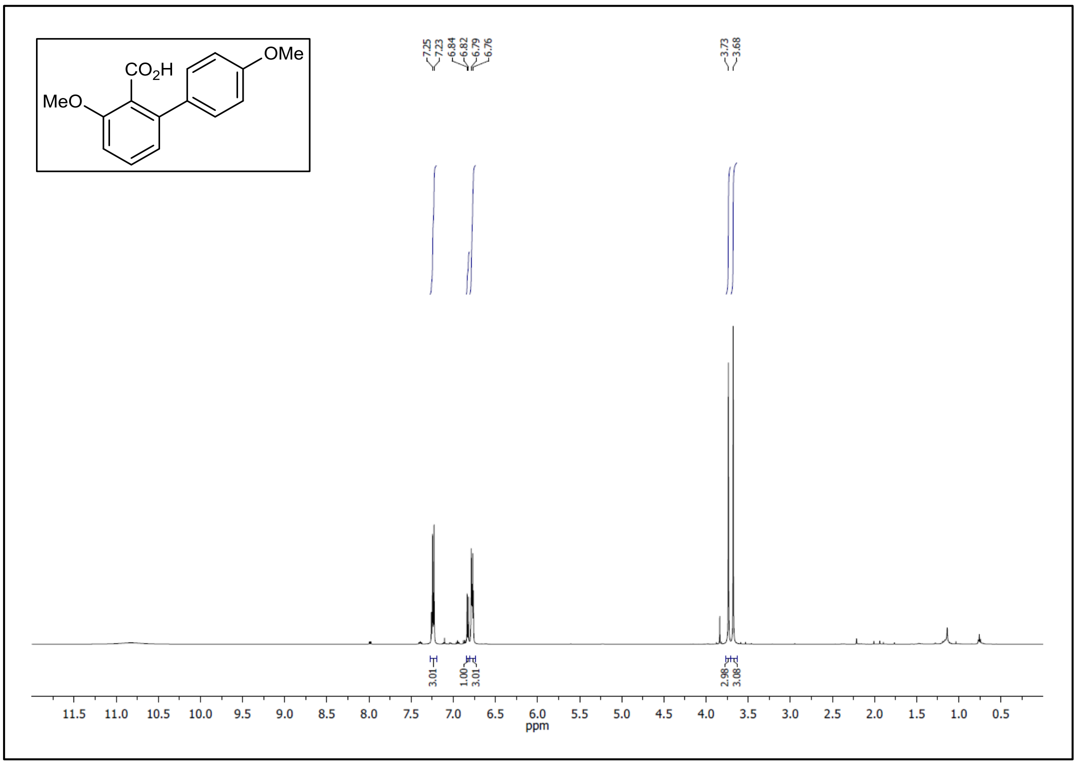

^{13}C NMR ($CDCl_3$)

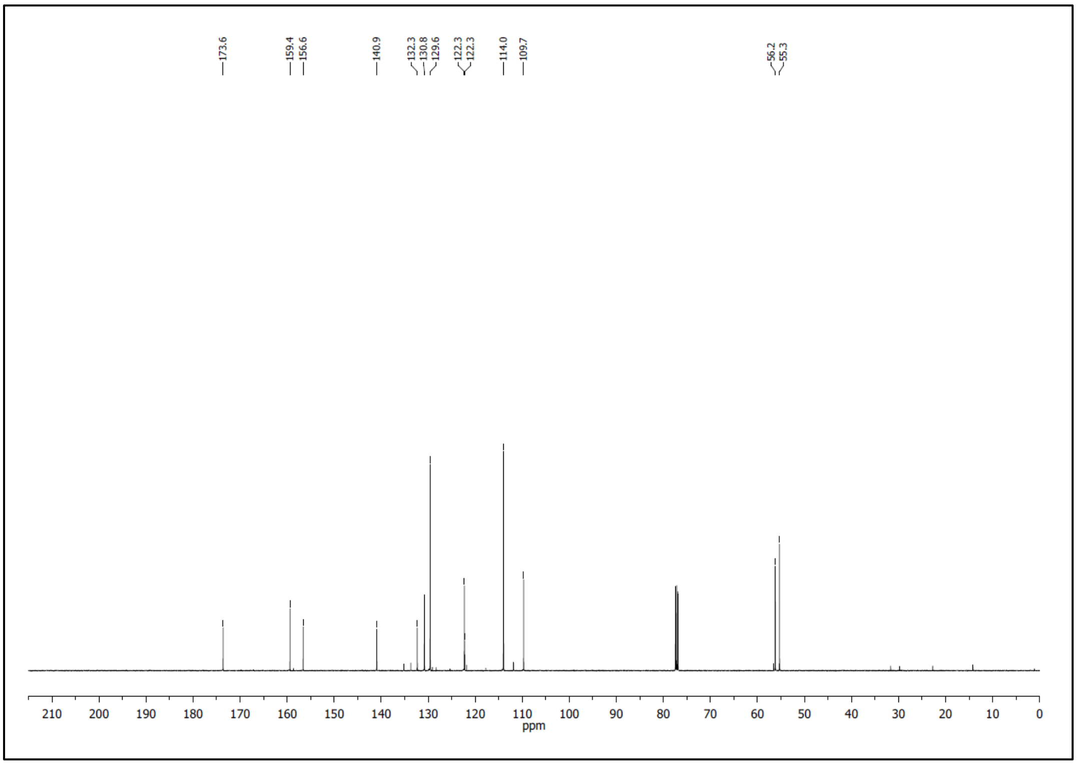

2-fluoro-6-(4-methoxyphenyl)benzoic acid (**3ca**)

^{1}H NMR ($CDCl_3$)

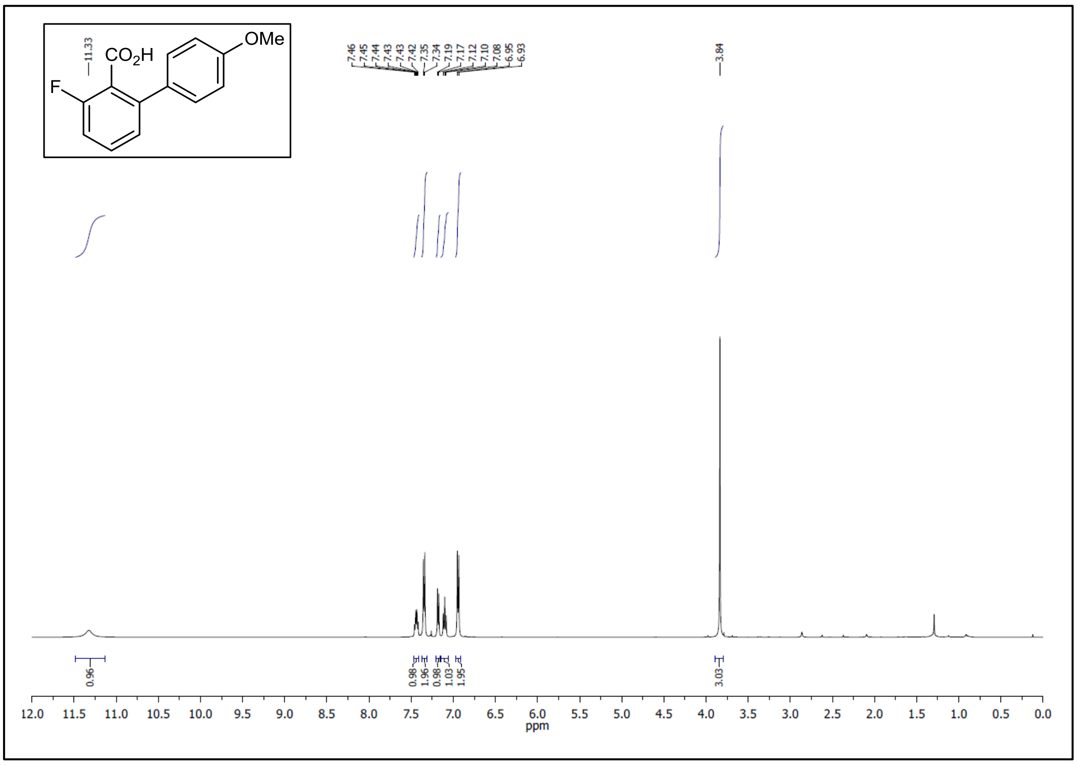

^{13}C NMR ($CDCl_3$)

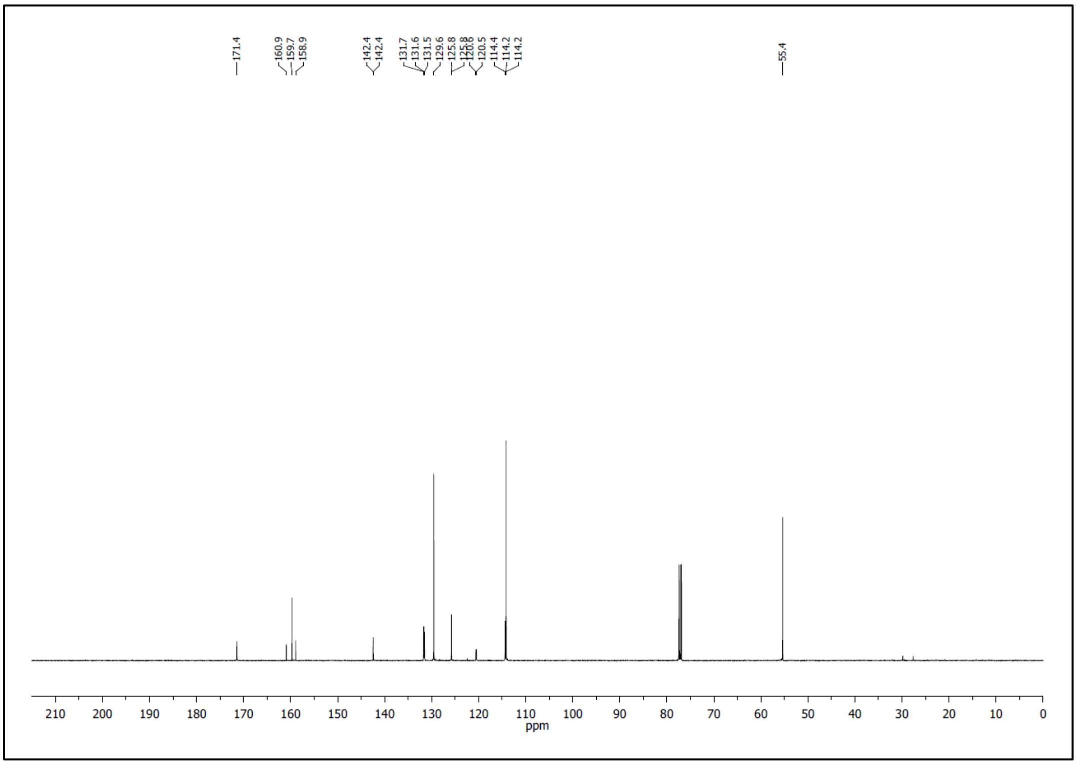

^{19}F NMR ($CDCl_3$)

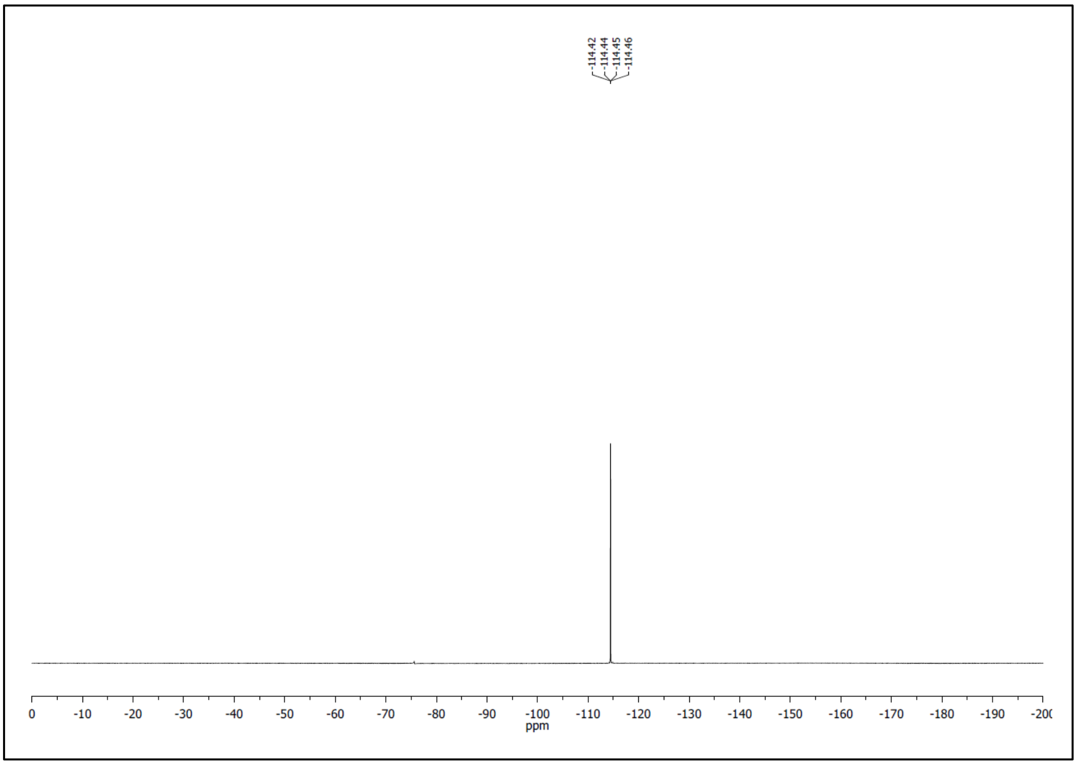

2-(4-methoxyphenyl)-6-(trifluoromethyl)benzoic acid (**3da**)

^{1}H NMR ($CDCl_3$)

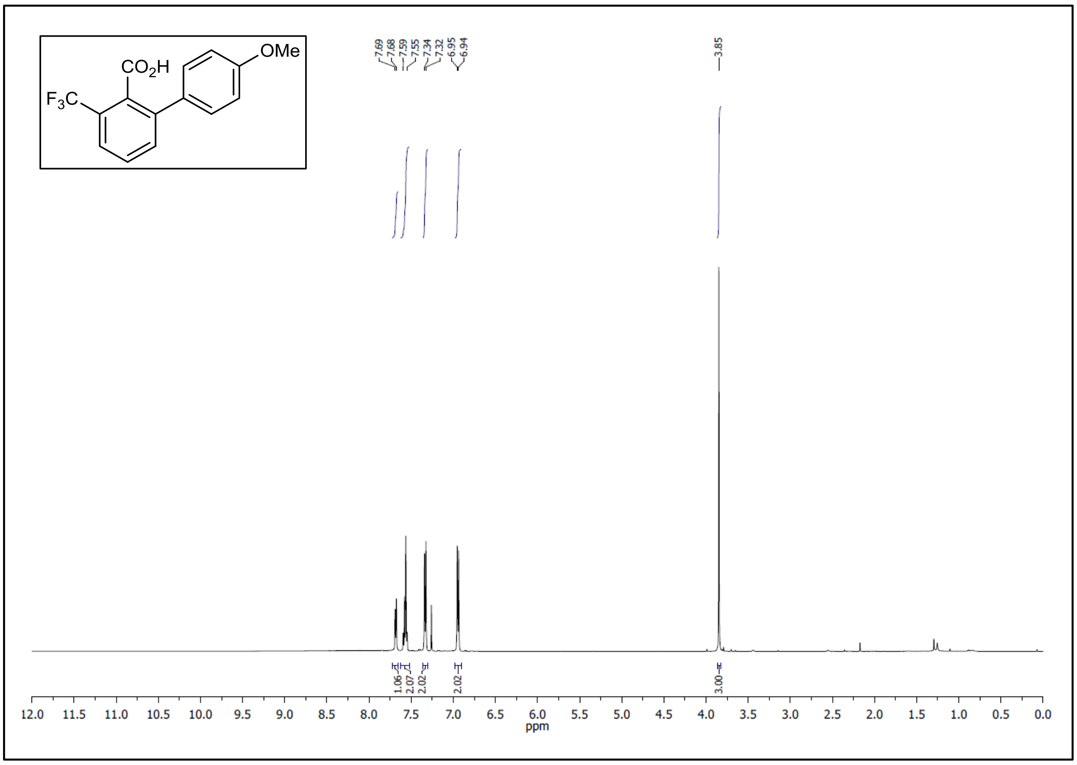

^{13}C NMR ($CDCl_3$)

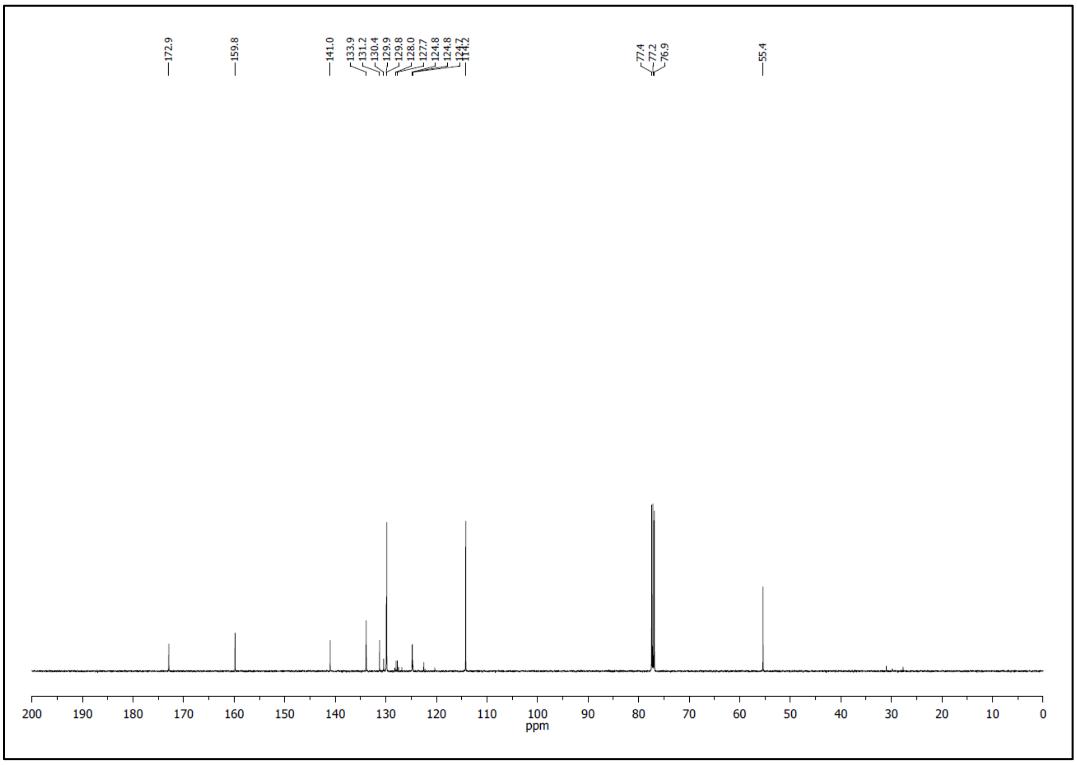

^{19}F NMR ($CDCl_3$)

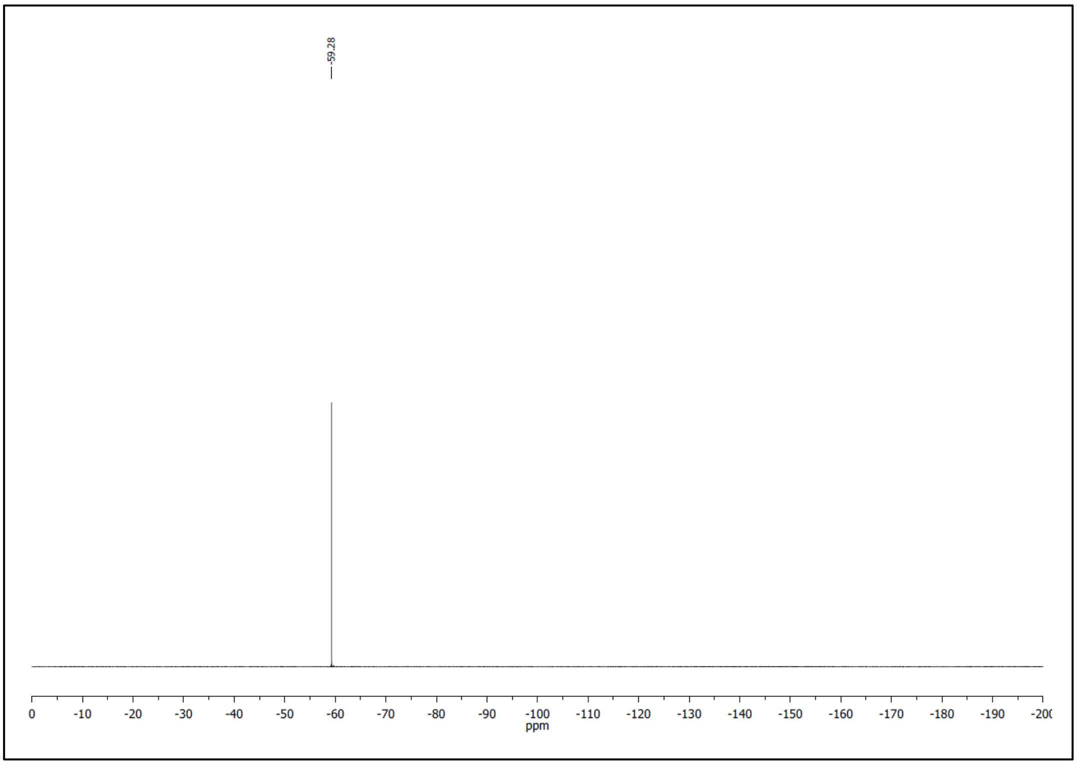

methyl 3-benzoyl-4'-methoxy-[1,1'-biphenyl]-2-carboxylate (**3ea**)

^{1}H NMR ($(CD_3)_2CO$)

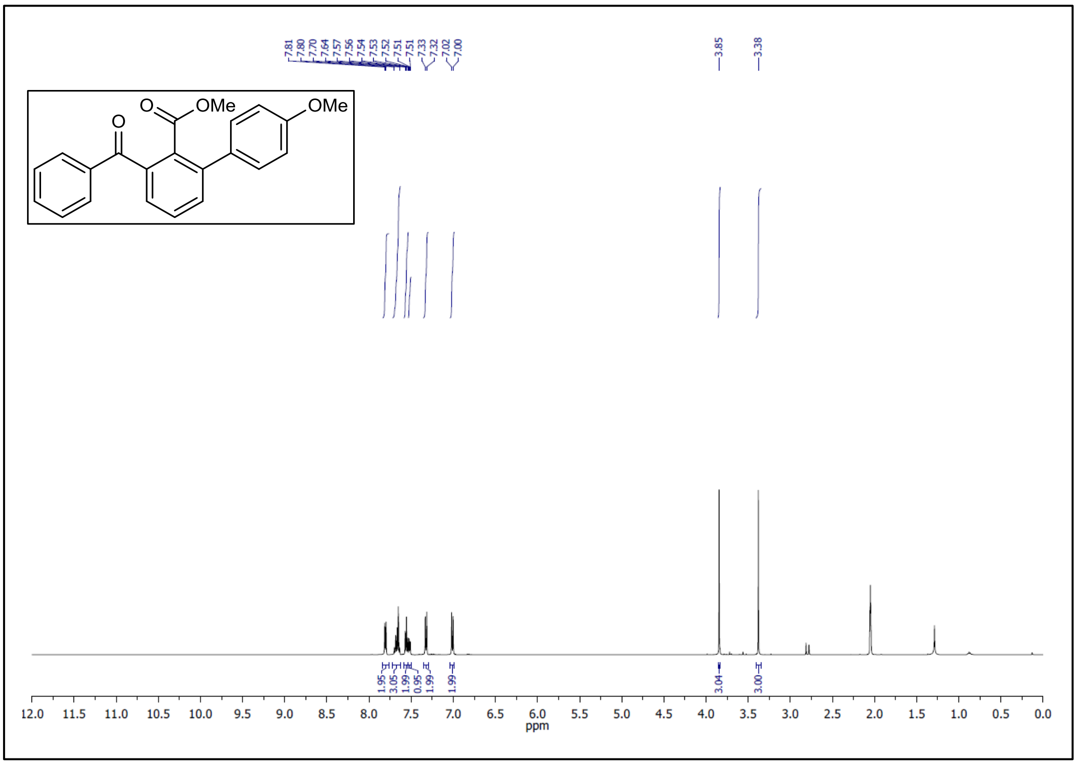

^{13}C NMR ($(CD_3)_2CO$)

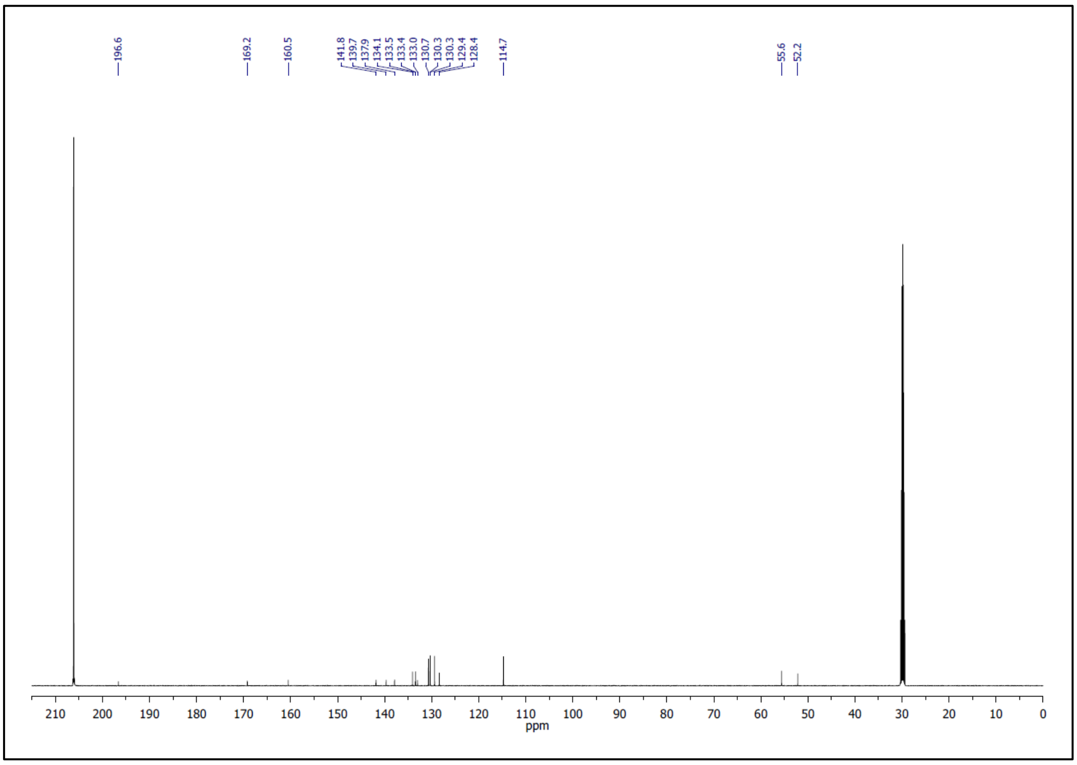

2-(4-methoxyphenyl)naphthalene-1-carboxylic acid (**3fa**)

^{1}H NMR ($CDCl_3$)

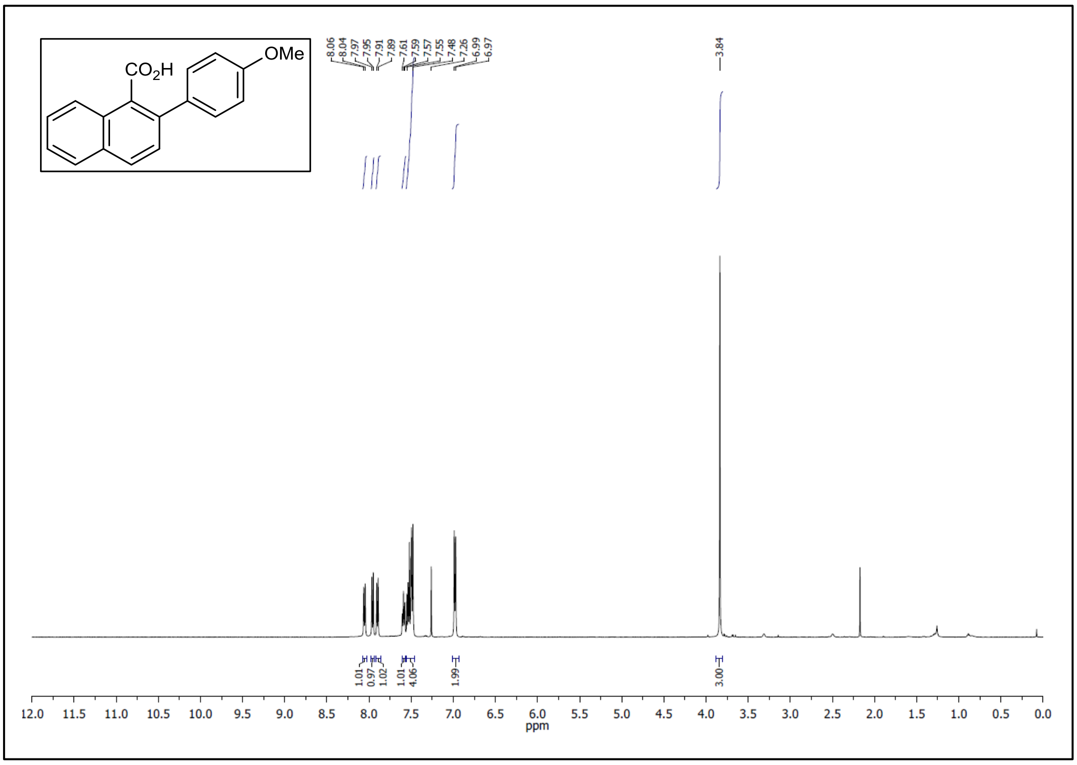

^{13}C NMR ($CDCl_3$)

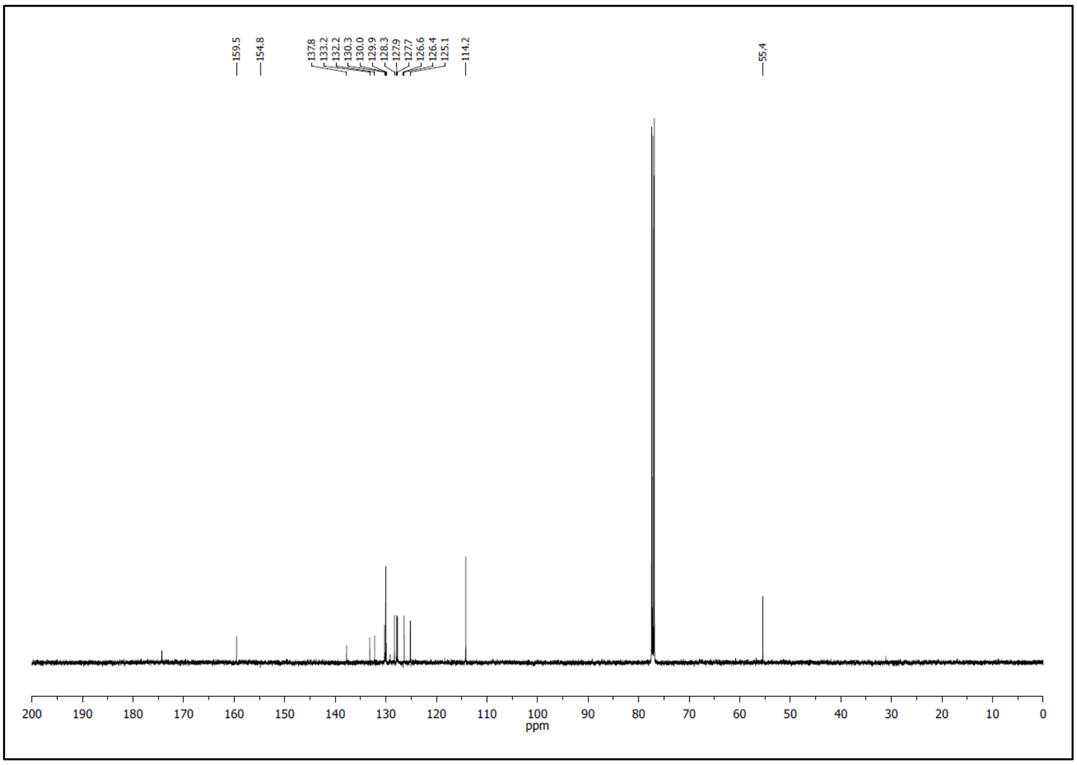

3-methoxy-2-(4-methoxyphenyl)-6-methylbenzoic acid (**3ga**)

^{1}H NMR ($CDCl_3$)

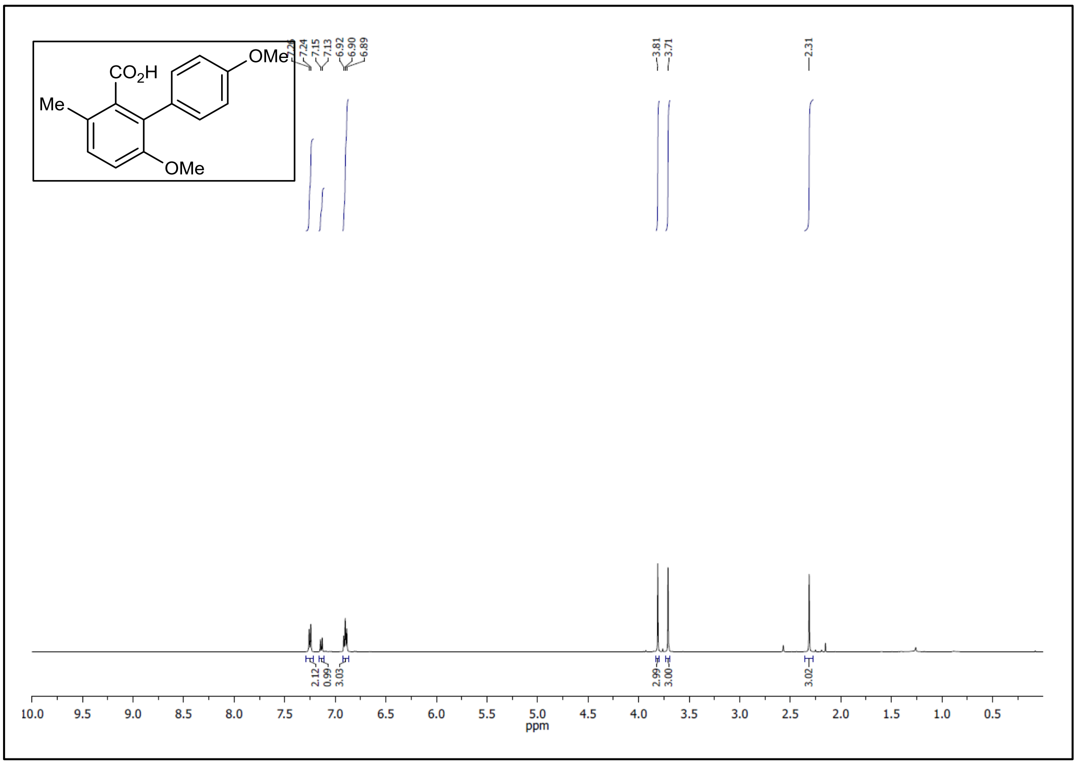

^{13}C NMR ($CDCl_3$)

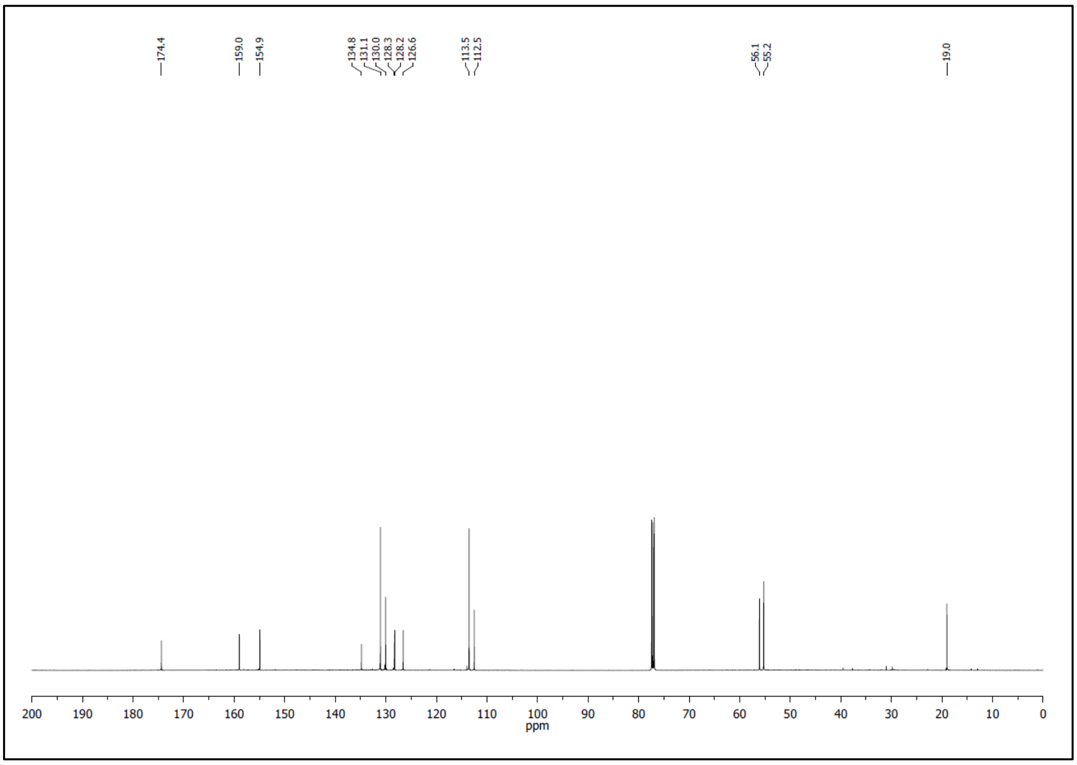

6-chloro-3,4'-dimethoxy-[1,1'-biphenyl]-2-carboxylic acid (**3ha**)

^{1}H NMR ($CDCl_3$)

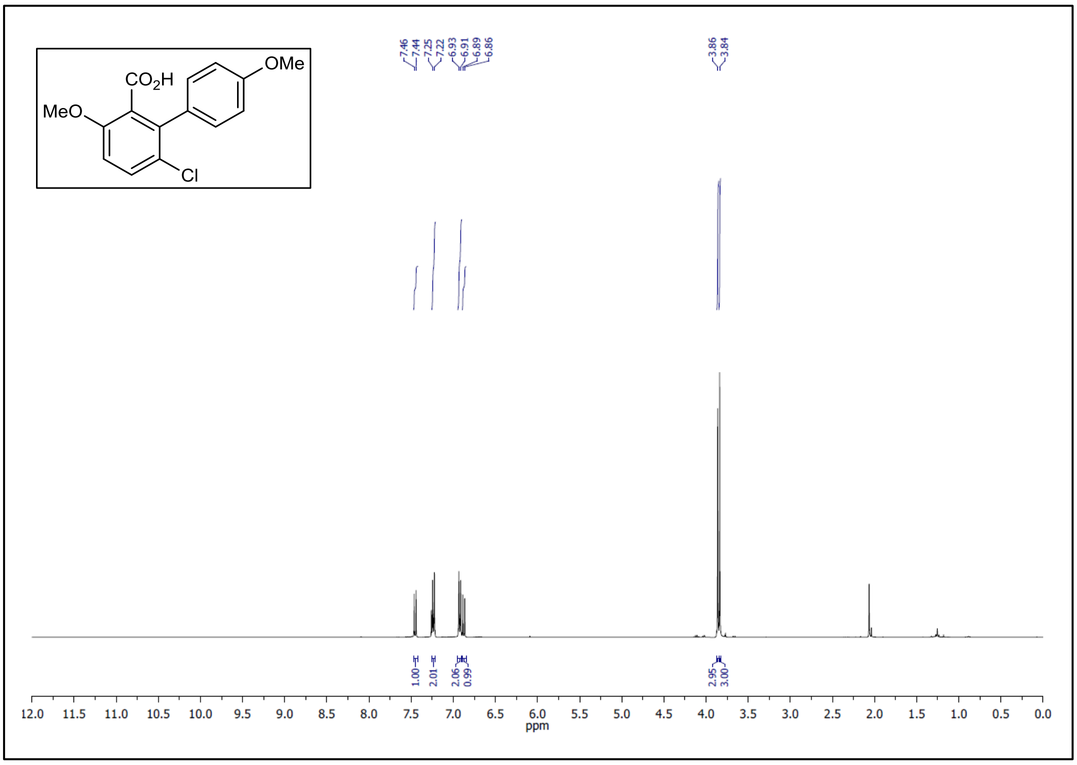

^{13}C NMR ($CDCl_3$)

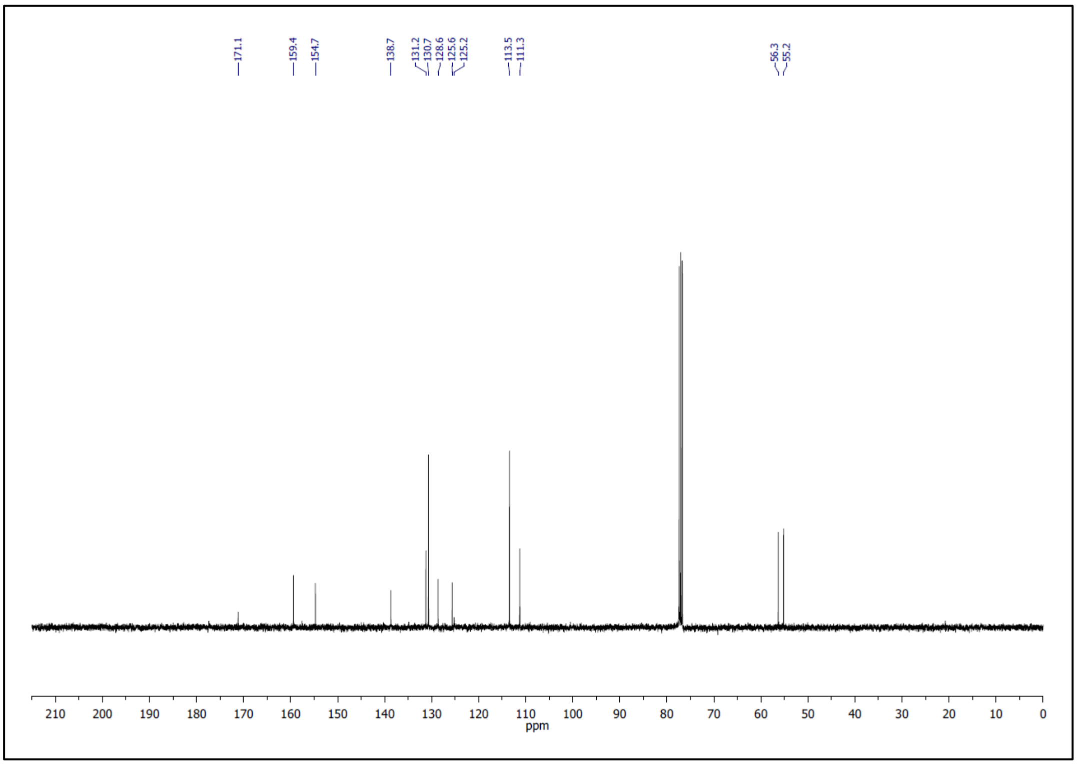

6-bromo-3,4'-dimethoxy-[1,1'-biphenyl]-2-carboxylic acid (**3ia**)

^{1}H NMR ($CDCl_3$)

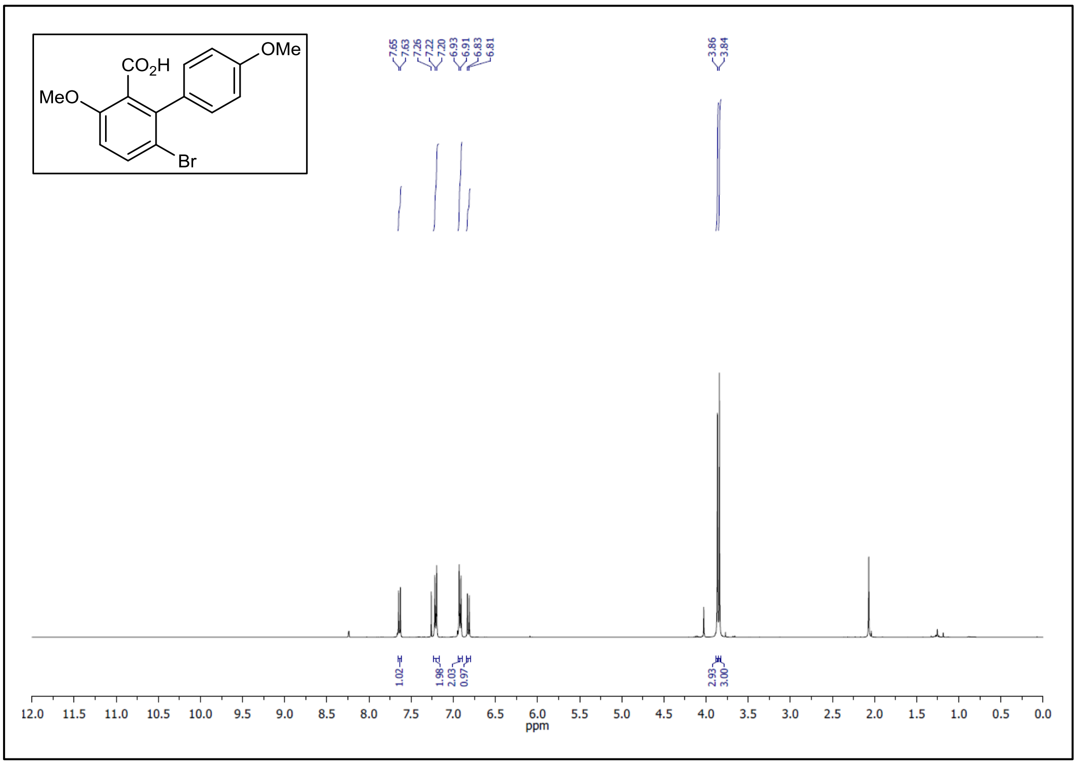

^{13}C NMR ($CDCl_3$)

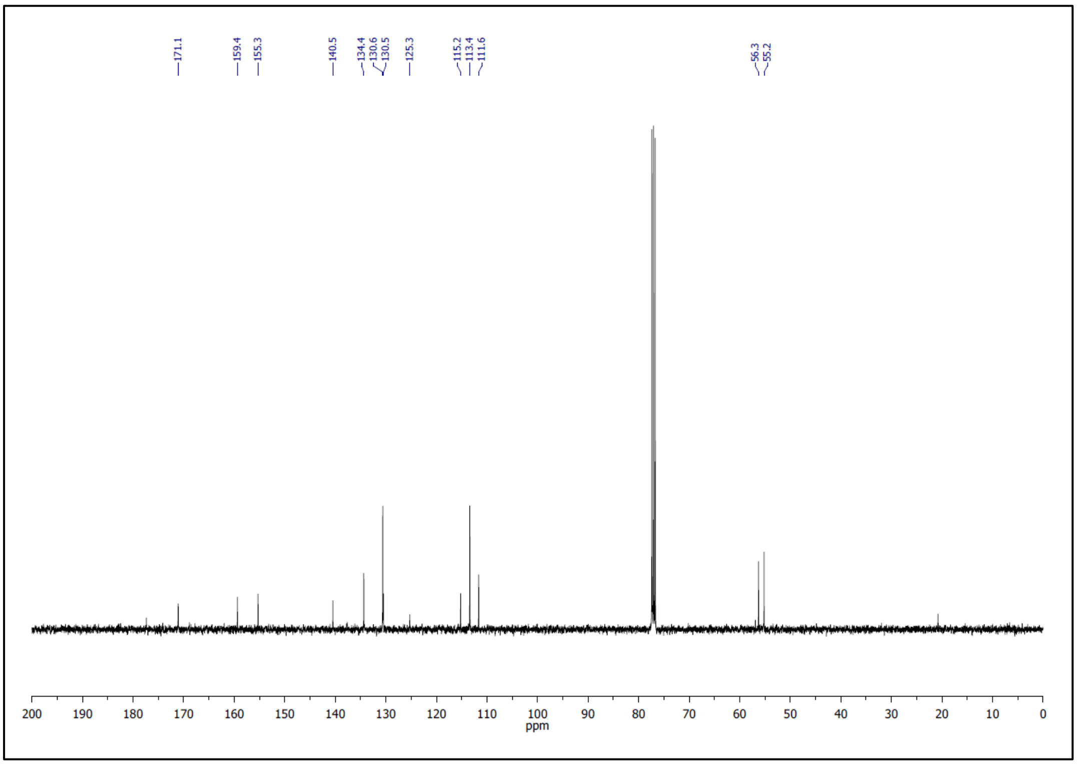

3,4'-dimethoxy-6-methyl-[1,1'-biphenyl]-2-carboxylic acid (**3ja**)

^{1}H NMR ($CDCl_3$)

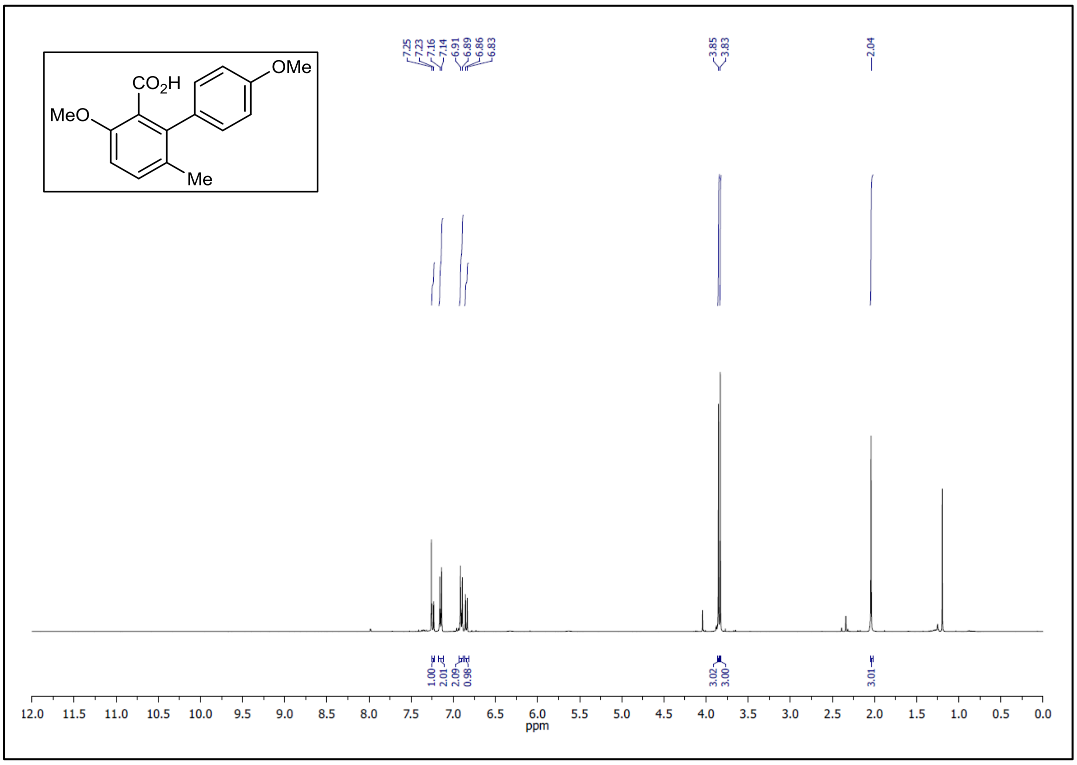

^{13}C NMR ($CDCl_3$)

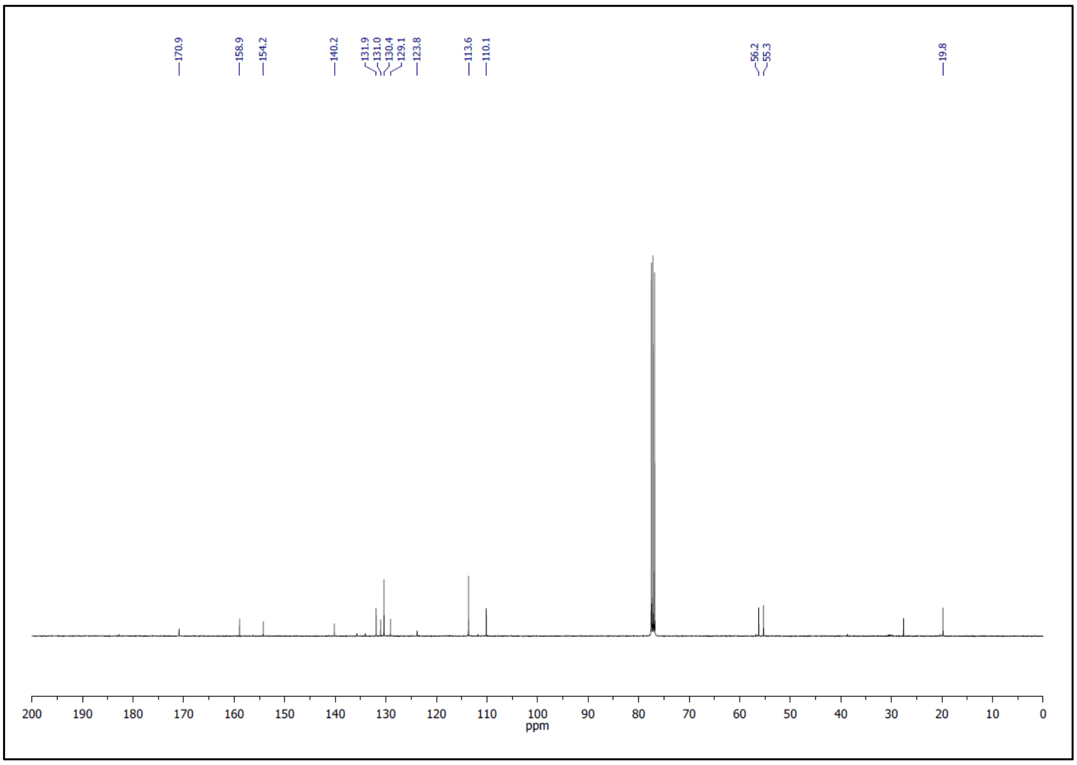

2-(4-methoxyphenyl)-5-(trifluoromethyl)benzoic acid (**3ka**)

^{1}H NMR ($CDCl_3$)

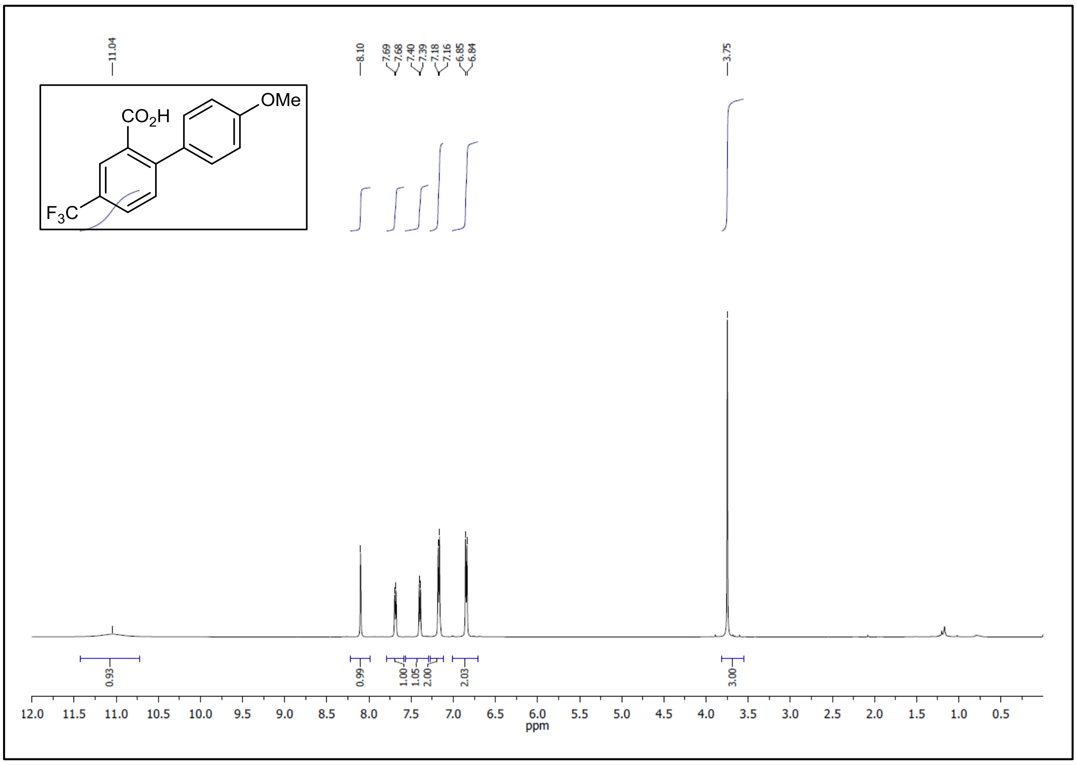

^{13}C NMR ($CDCl_3$)

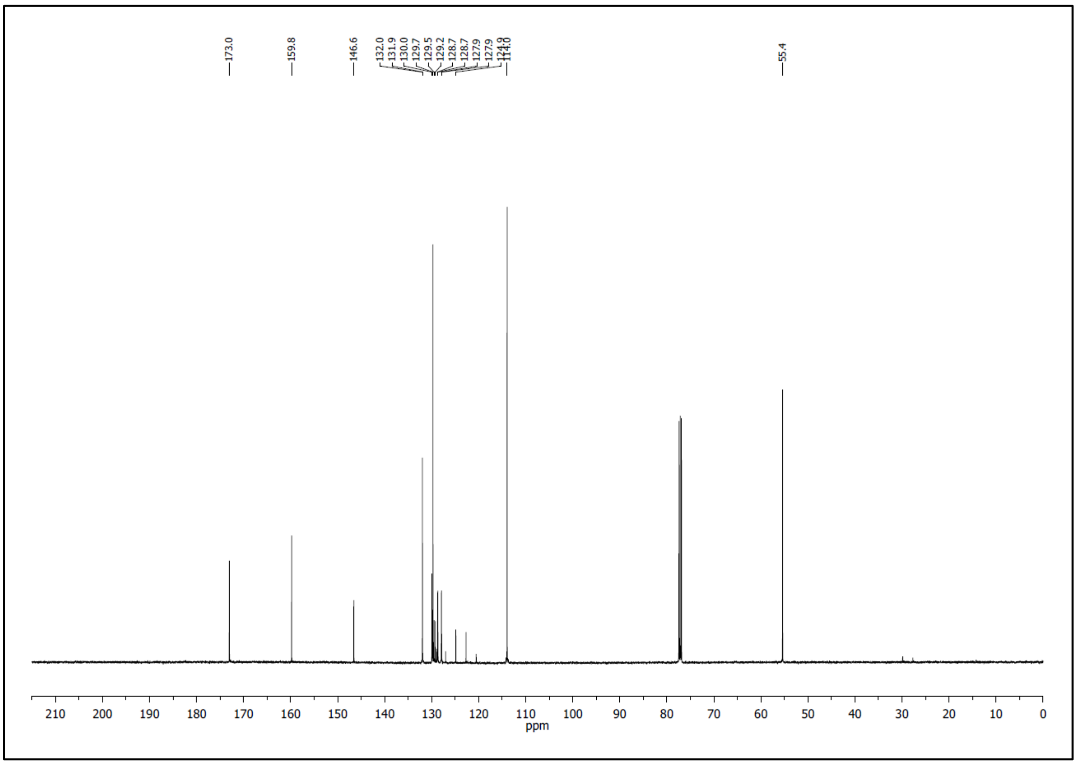

^{19}F NMR ($CDCl_3$)

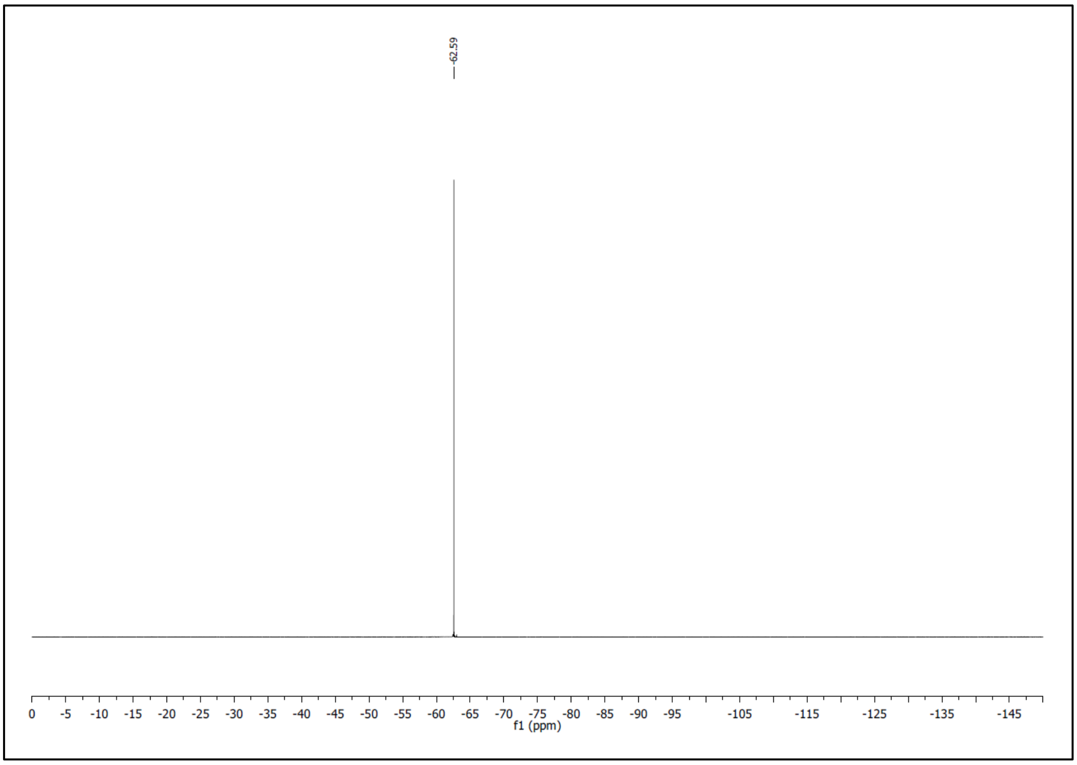

4-methoxy-[1,1':4',1''-terphenyl]-2'-carboxylic acid (**3la**)

^{1}H NMR ($CDCl_3$)

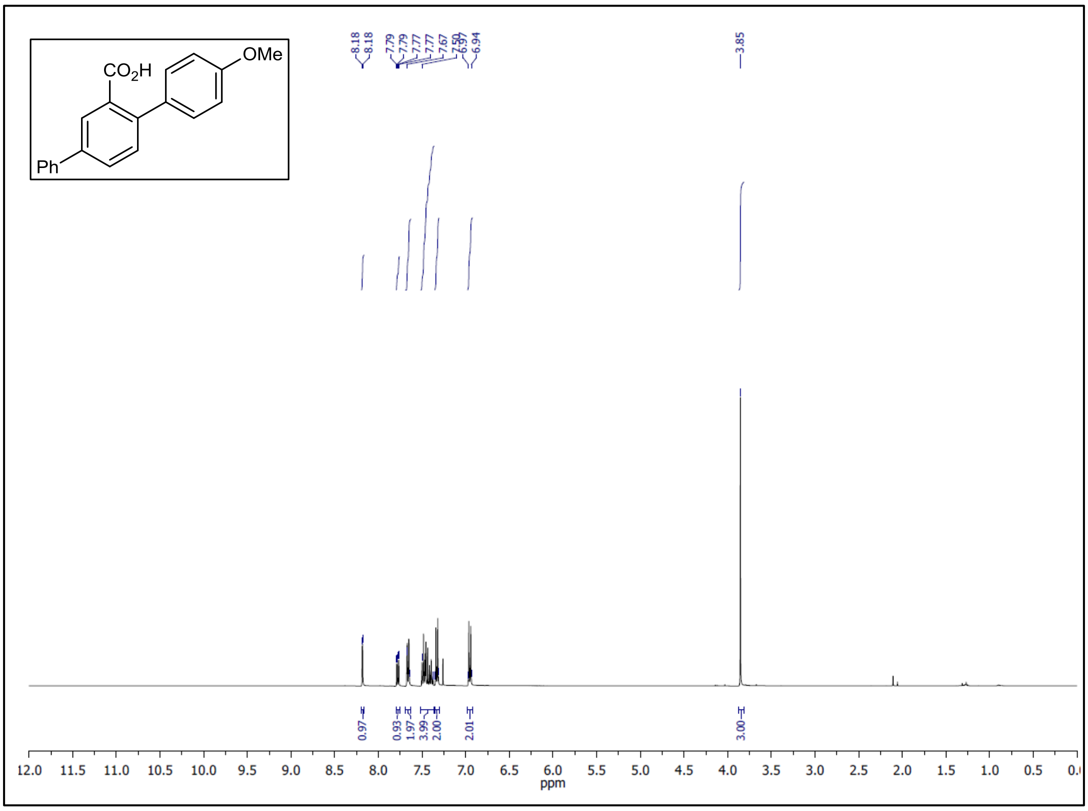

^{13}C NMR ($CDCl_3$)

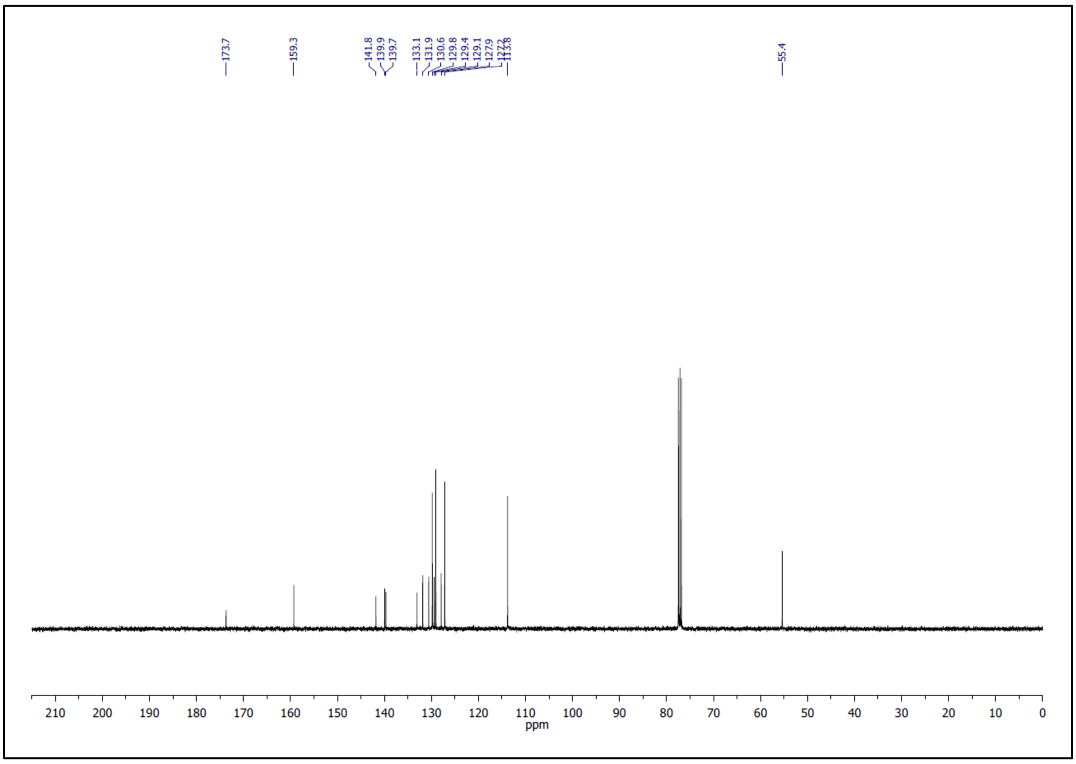

4'-methoxy-4-(methoxycarbonyl)-[1,1'-biphenyl]-2-carboxylic acid (**3ma**)

^{1}H NMR ($CDCl_3$)

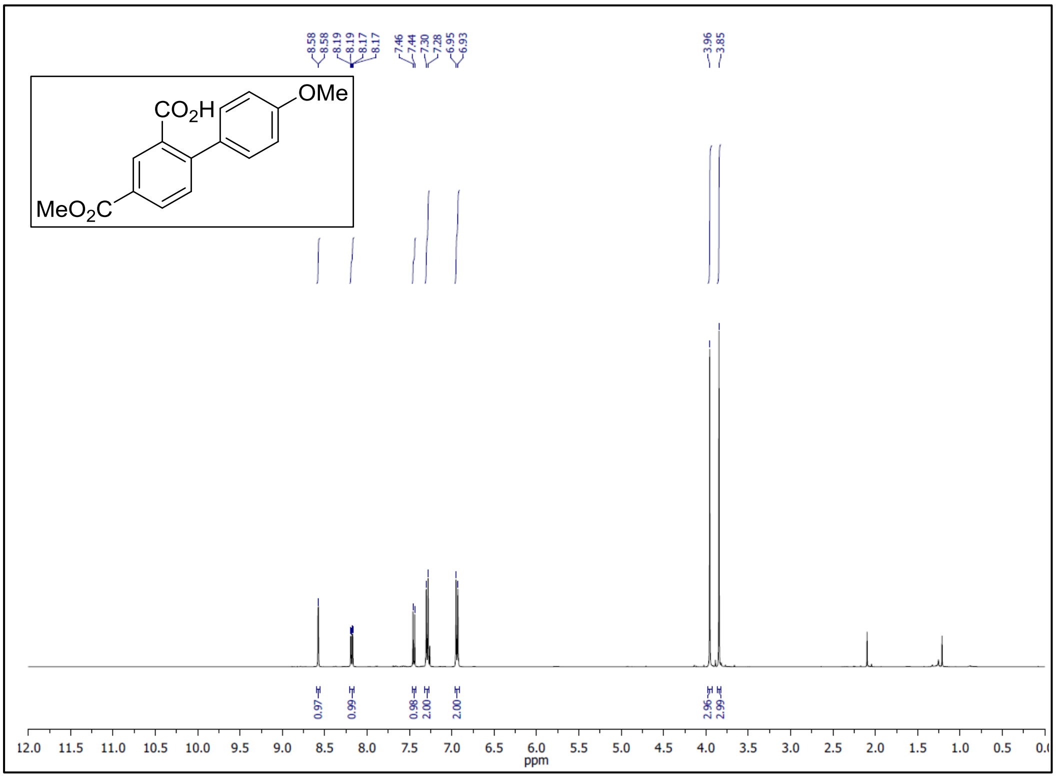

^{13}C NMR ($CDCl_3$)

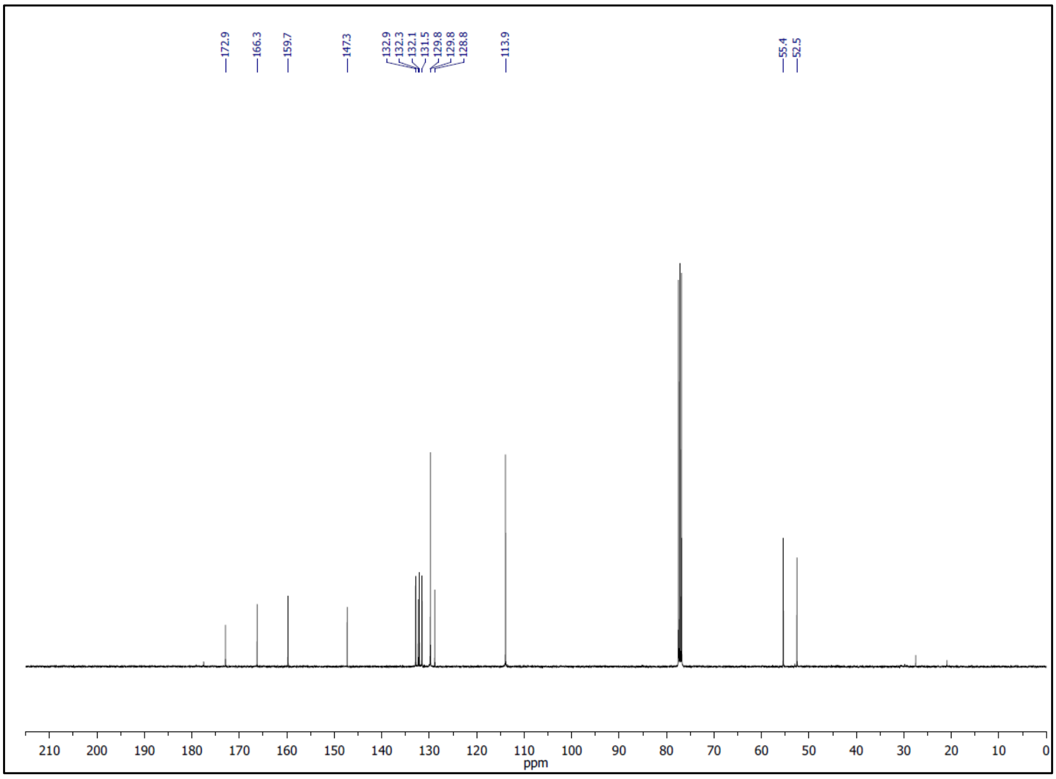

methyl 4'-methoxy-4-methyl-[1,1'-biphenyl]-2-carboxylate (**3na**)

^{1}H NMR ($CDCl_3$)

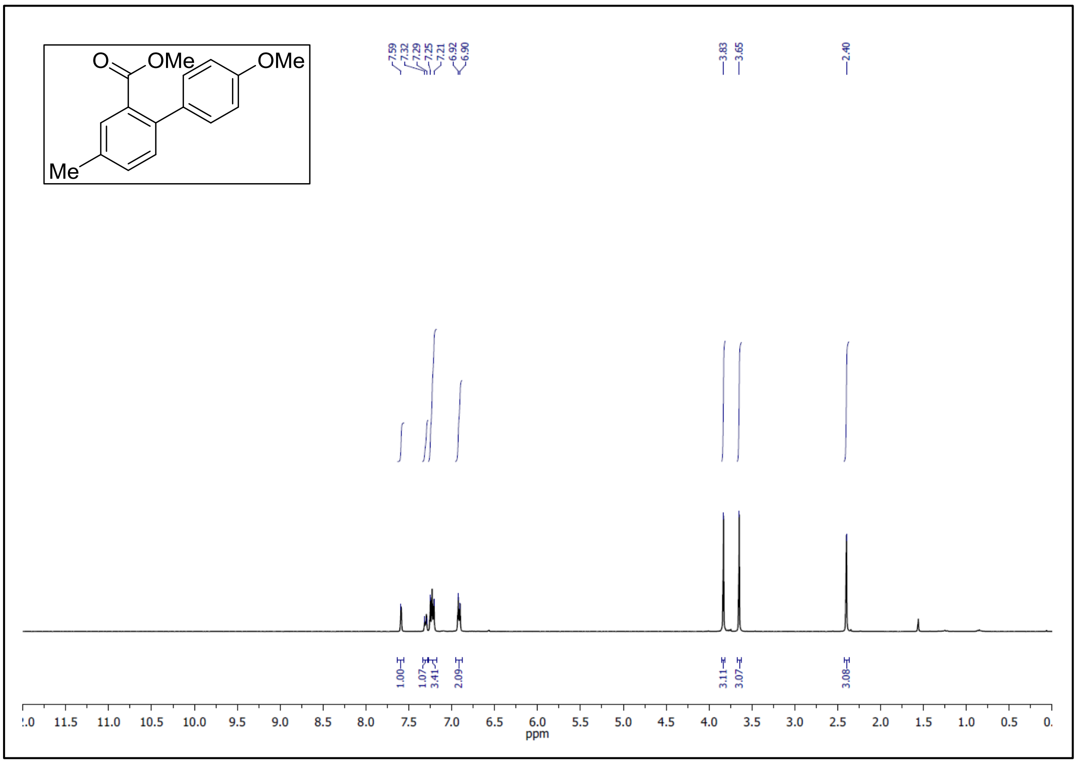

^{13}C NMR ($CDCl_3$)

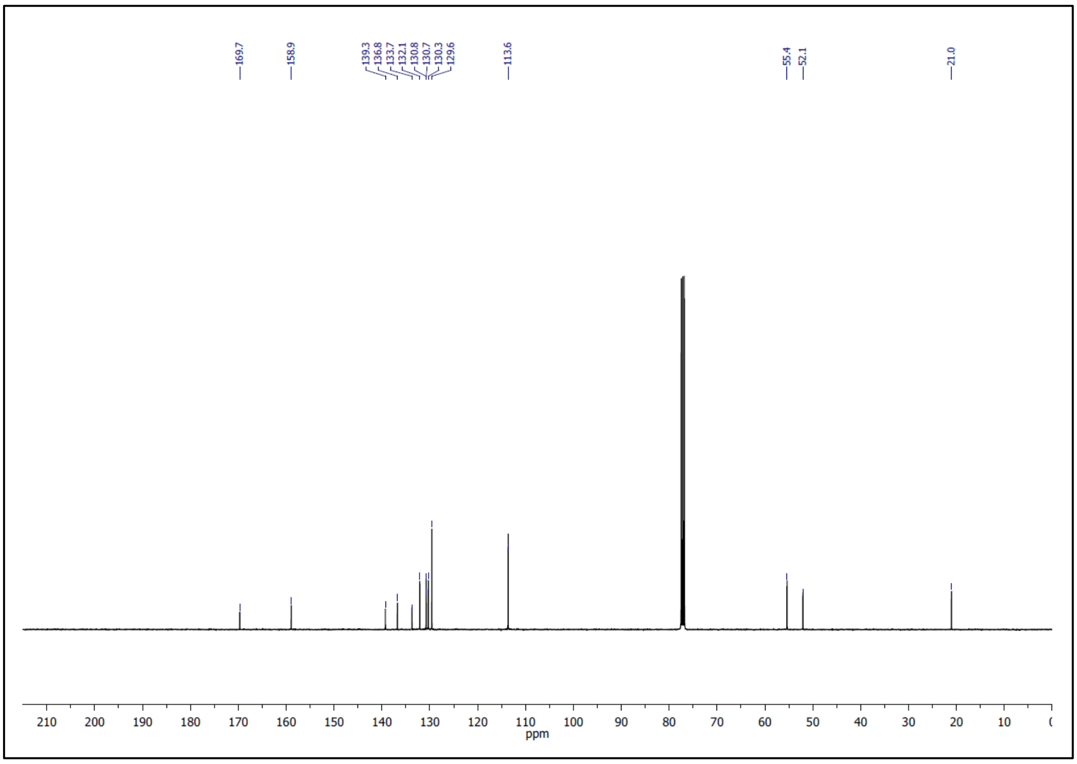

methyl 4,4''-dimethoxy-4'-methyl-[1,1':3',1''-terphenyl]-2'-carboxylate (**3na'**)

^{1}H NMR ($CDCl_3$)

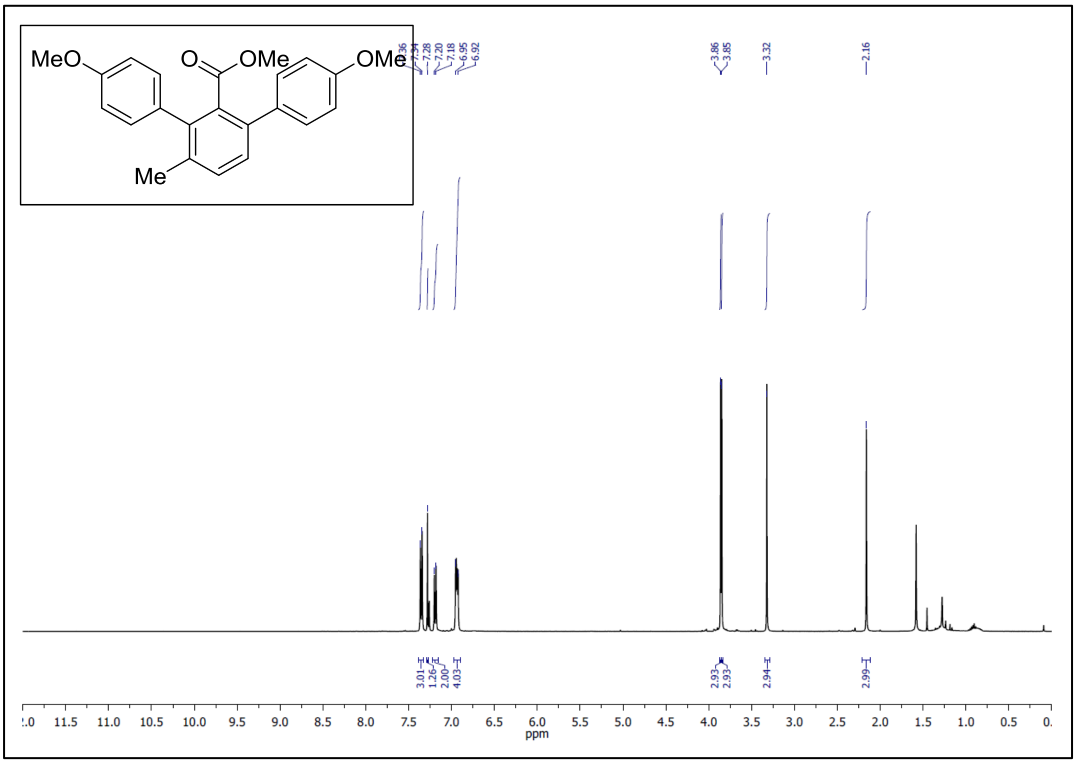

^{13}C NMR ($CDCl_3$)

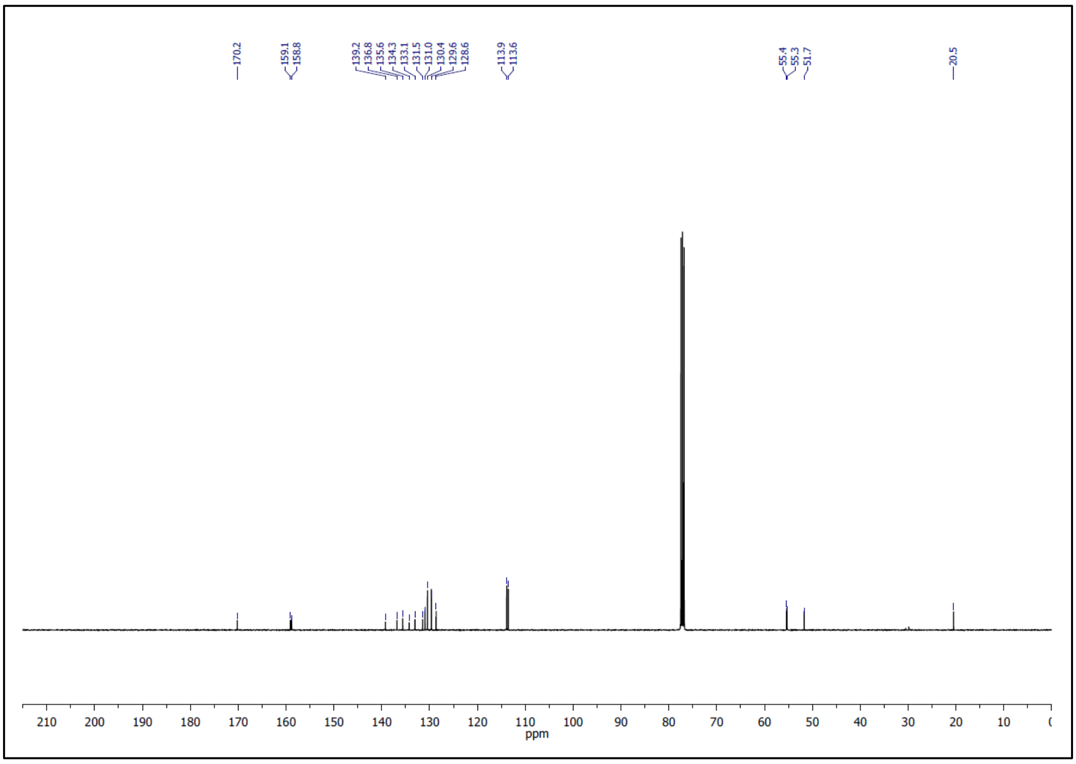

4'-fluoro-4,4''-dimethoxy-[1,1':3',1''-terphenyl]-2'-carboxylic acid (**3oa**)

^{1}H NMR ($(CD_3)_2CO$)

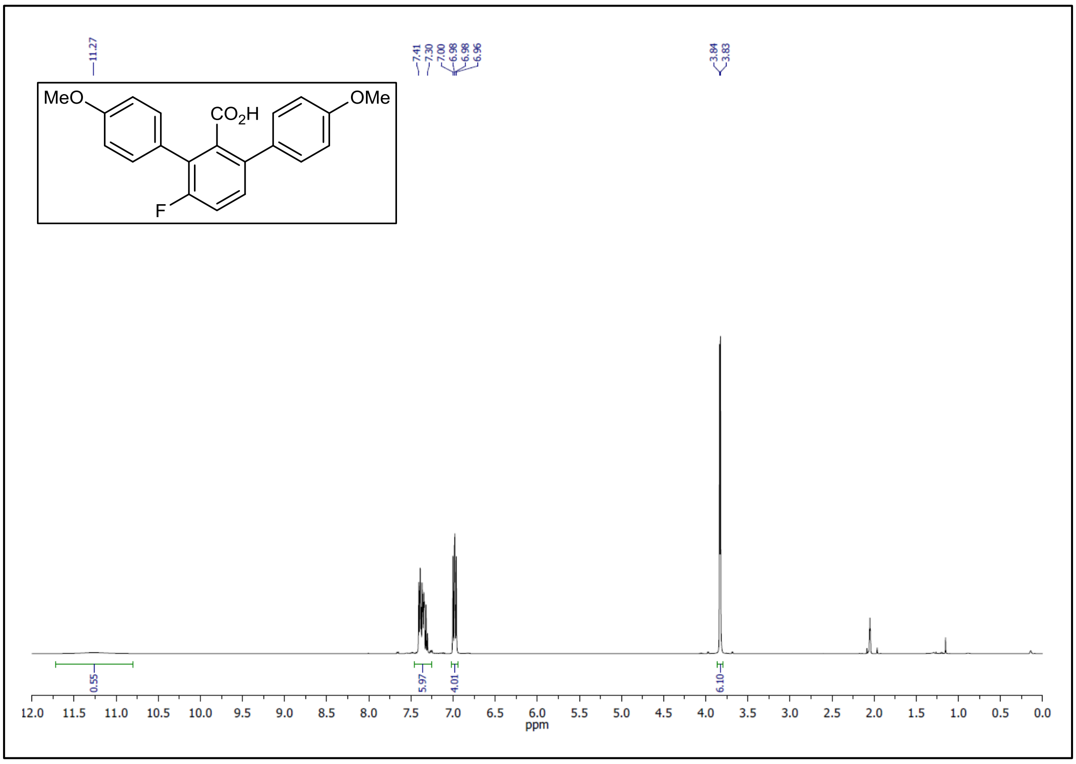

^{13}C NMR ($(CD_3)_2CO$)

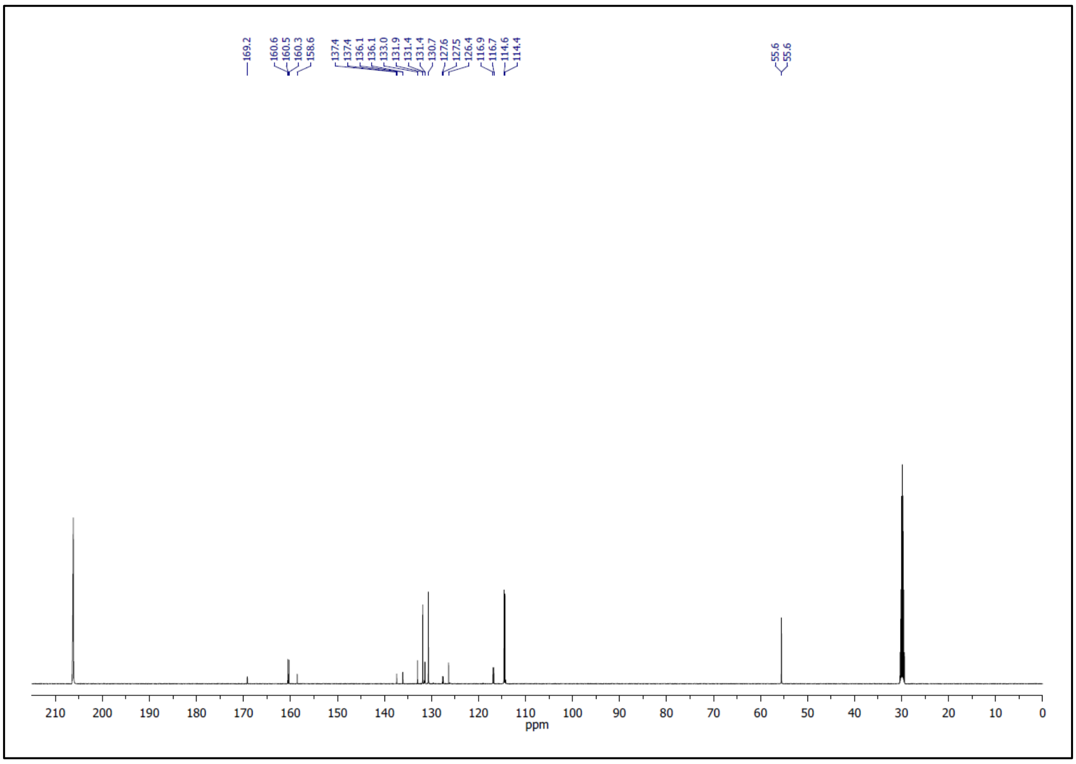

3-methoxy-2,6-bis(4-methoxyphenyl)benzoic acid (**3pa**)

^{1}H NMR ($CDCl_3$)

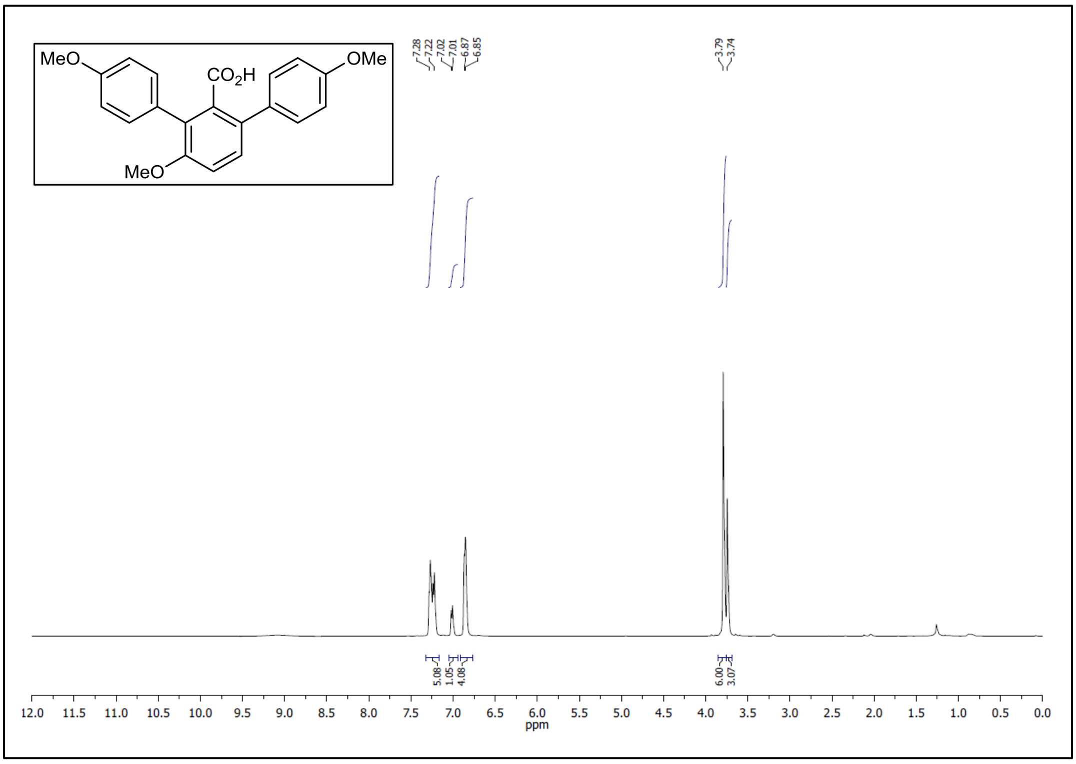

^{13}C NMR ($CDCl_3$)

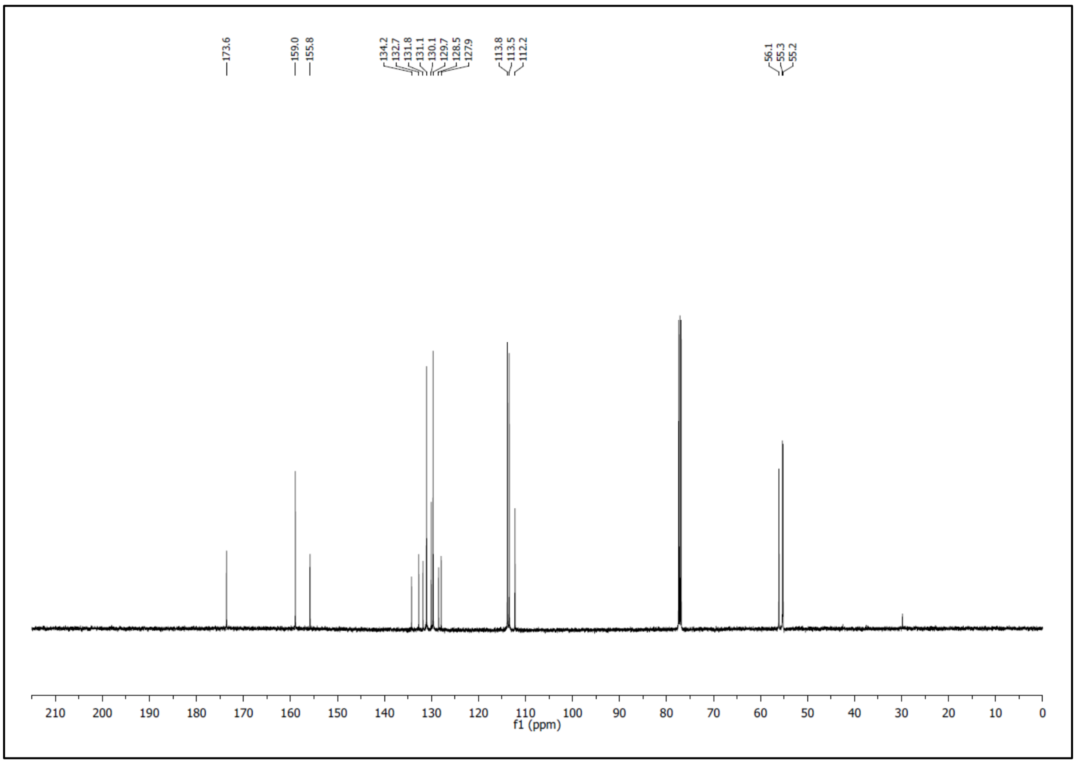

4'-chloro-4,4''-dimethoxy-[1,1':3',1''-terphenyl]-2'-carboxylic acid (**3qa**)

^{1}H NMR ($CDCl_3$)

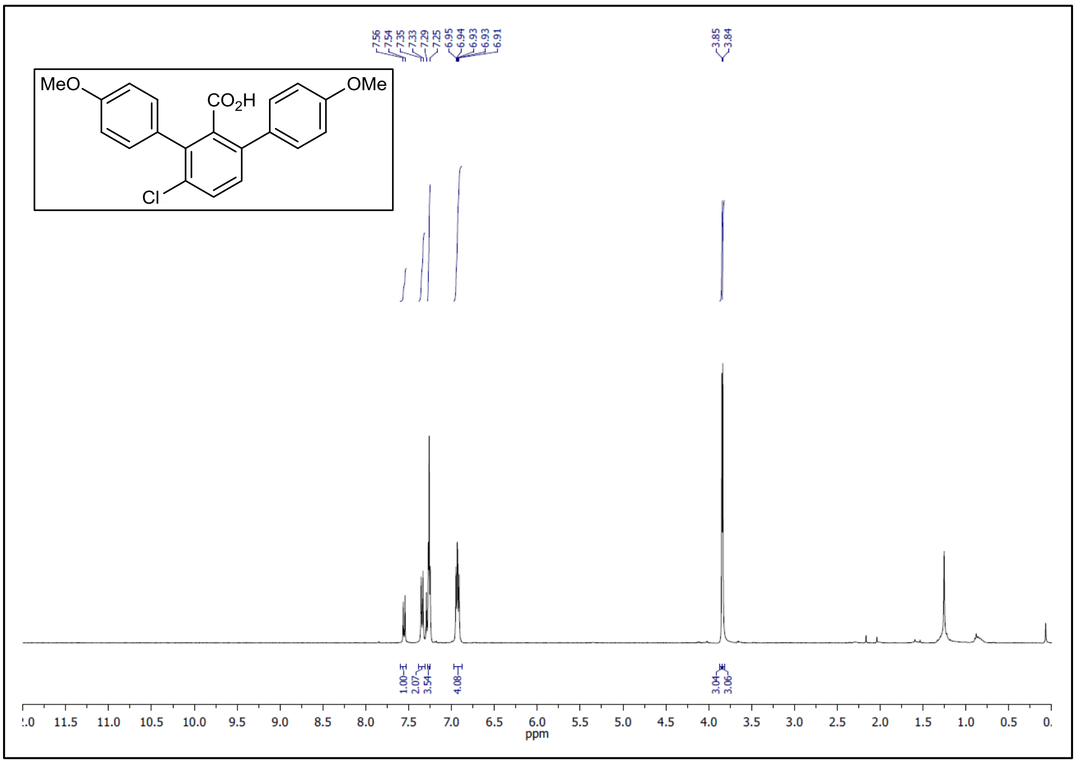

^{13}C NMR ($CDCl_3$)

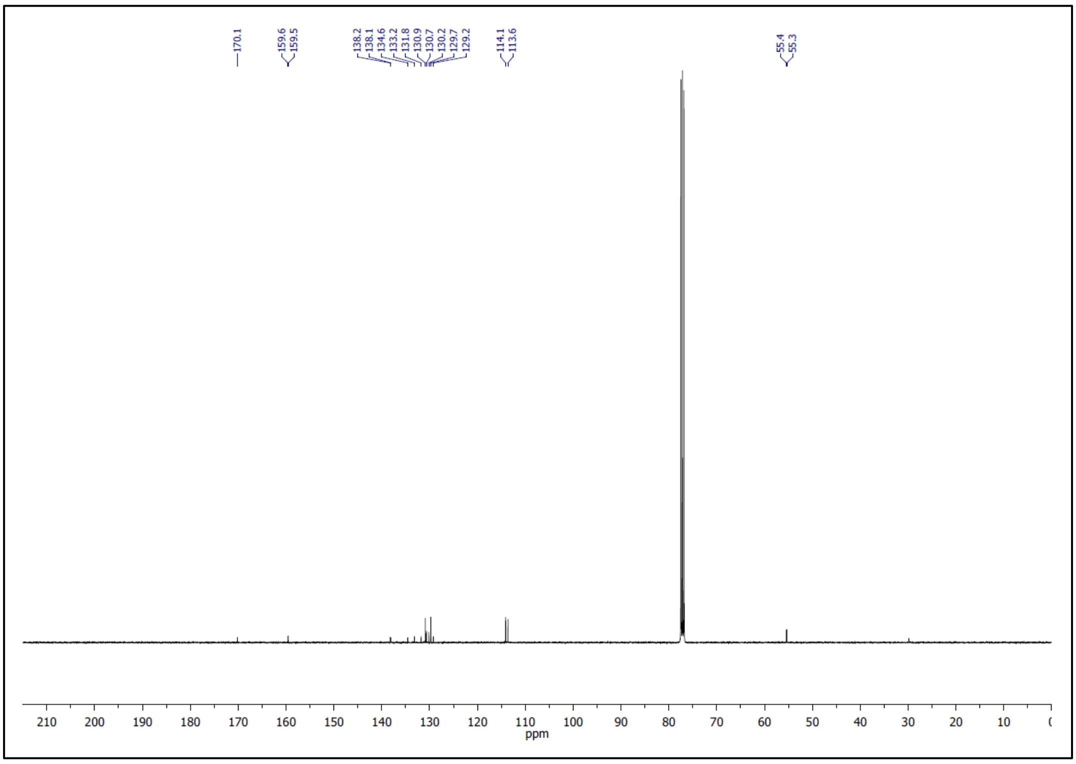

2,6-bis(4-methoxyphenyl)benzoic acid (**3ra**)

^{1}H NMR ($CDCl_3$)

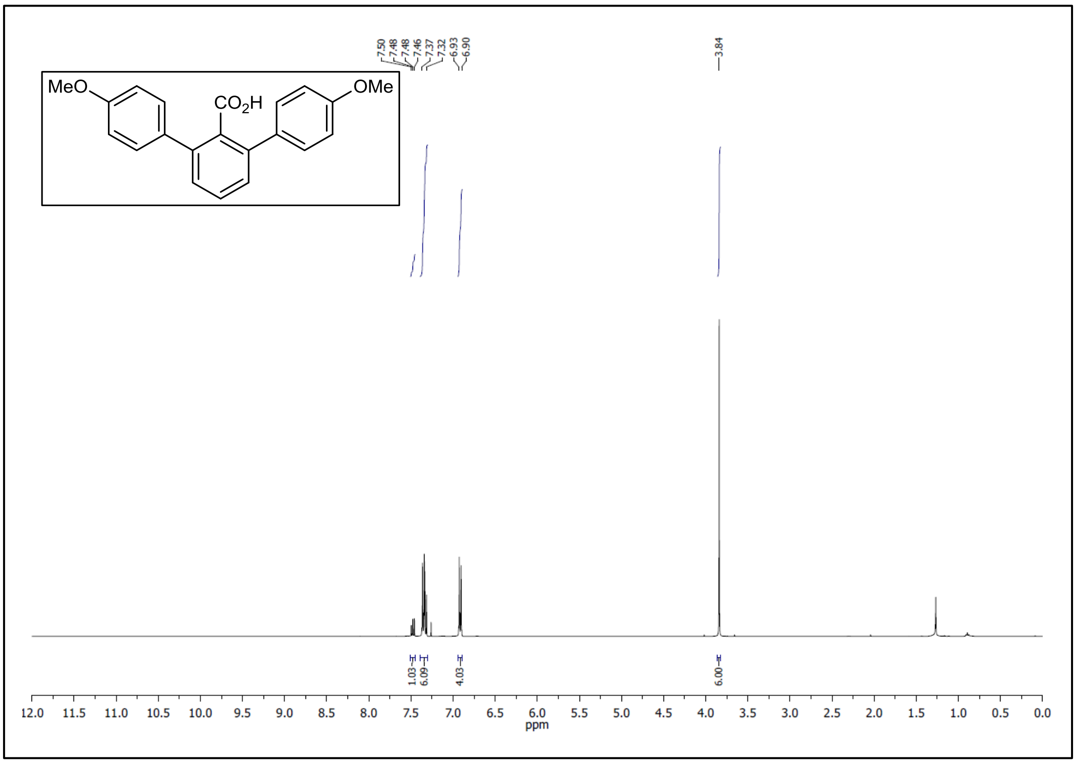

^{13}C NMR ($CDCl_3$)

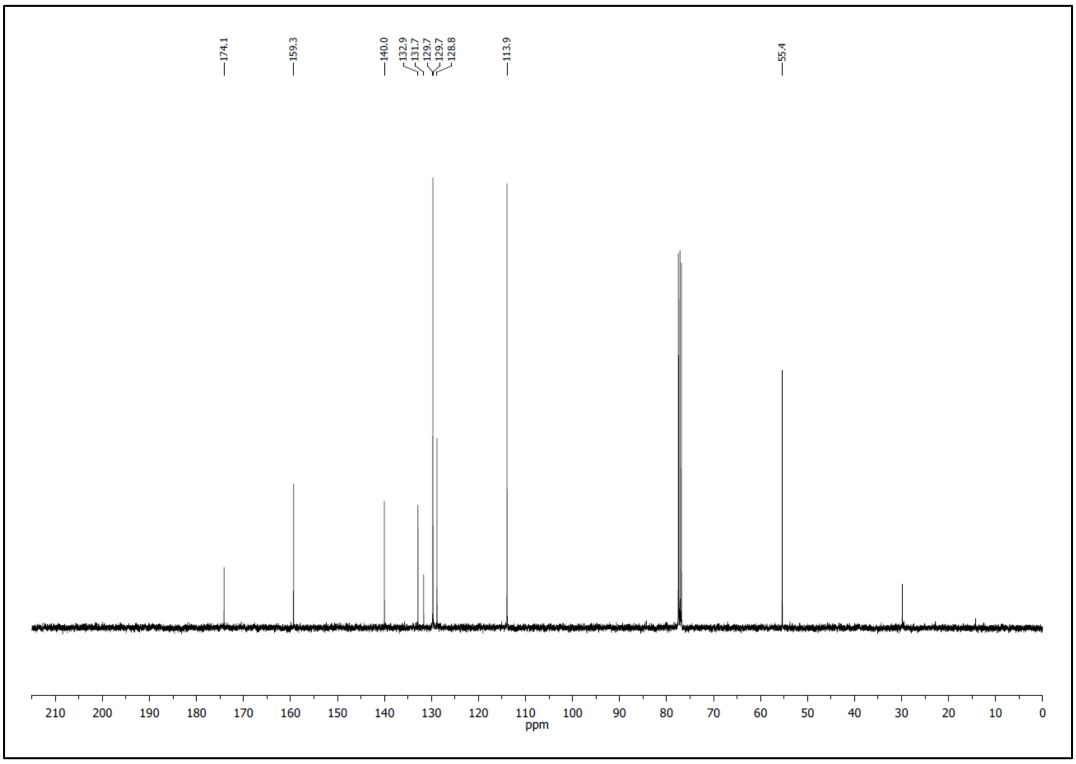

2,6-bis(4-methoxyphenyl)-4-methylbenzoic acid (**3sa**)

^{1}H NMR ($CDCl_3$)

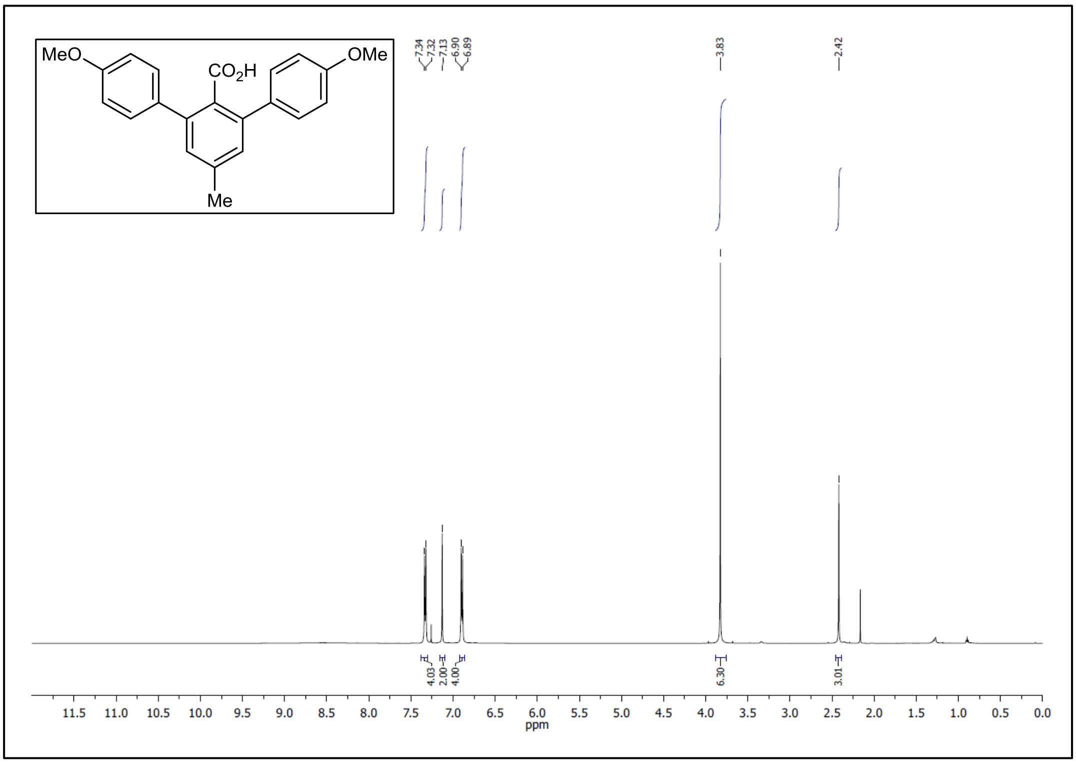

^{13}C NMR ($CDCl_3$)

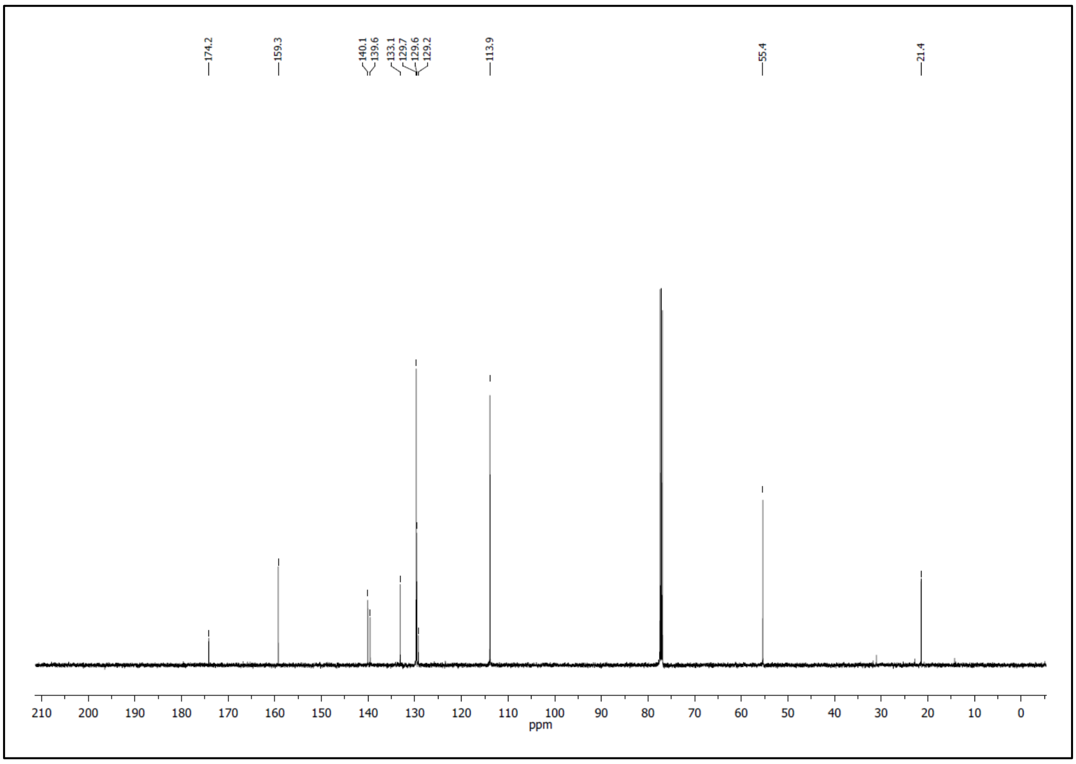

4,4'',5'-trimethoxy-[1,1':3',1''-terphenyl]-2'-carboxylic acid (**3ta**)

^{1}H NMR ($(CD_3)_2CO$)

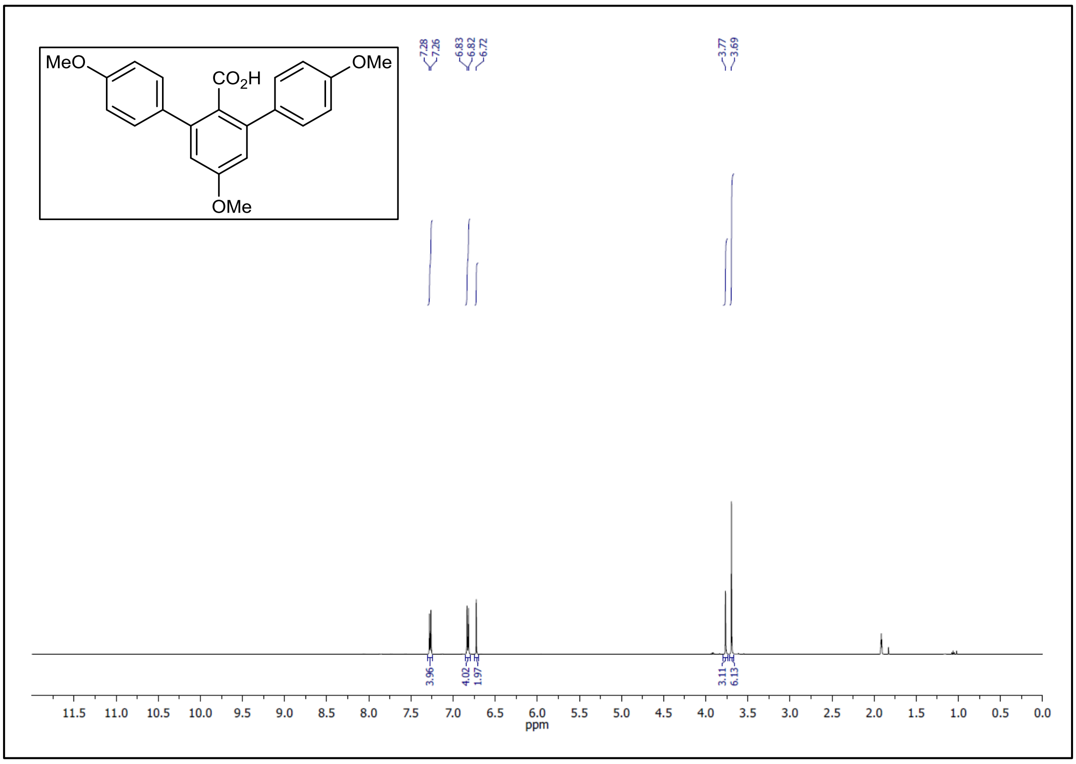

^{13}C NMR ($(CD_3)_2CO$)

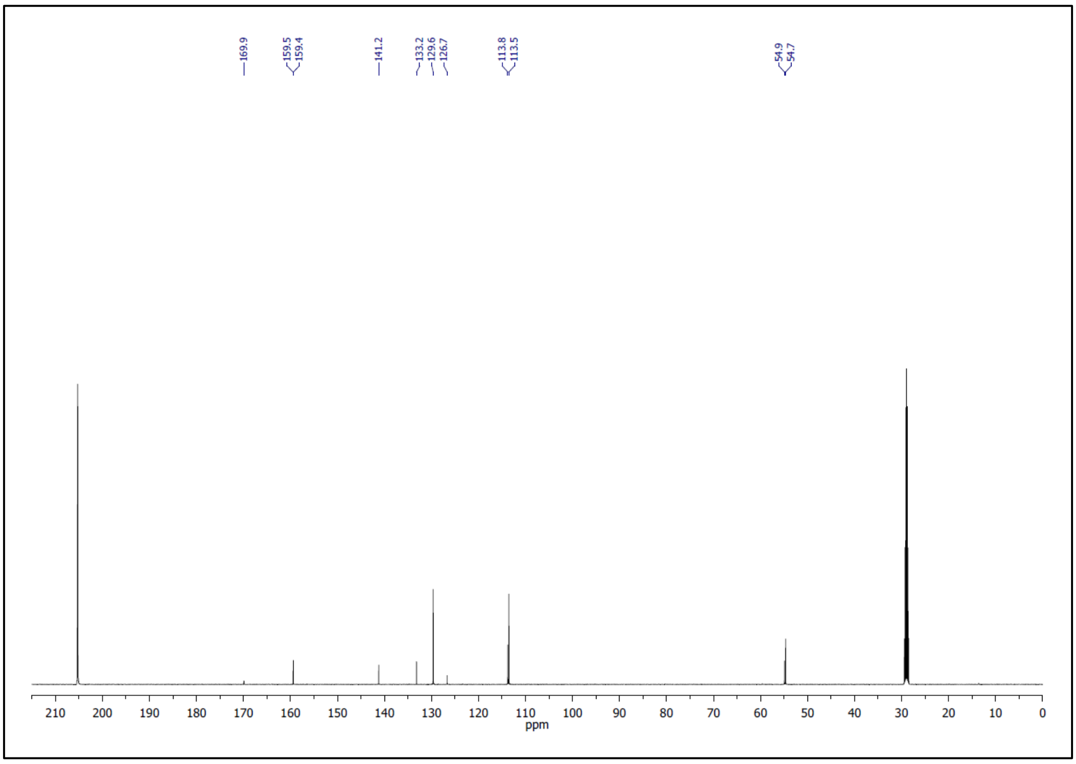

4,4''-dimethoxy-5'-(methylthio)-[1,1':3',1''-terphenyl]-2'-carboxylic acid (**3ua**)

^{1}H NMR ($(CD_3)_2CO$)

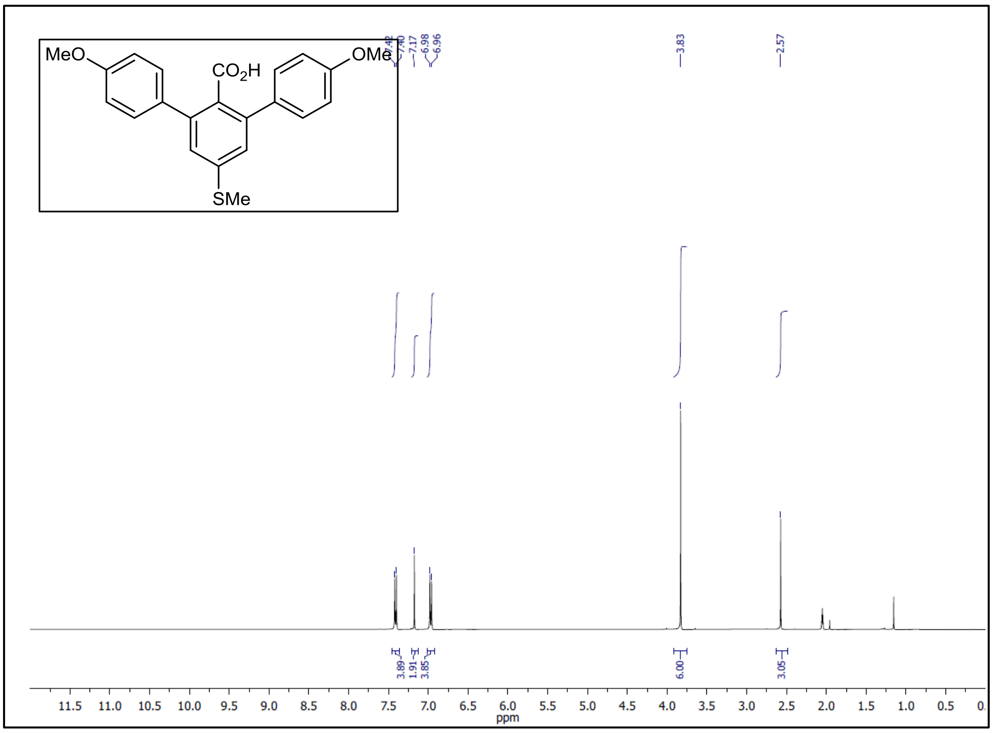

^{13}C NMR ($(CD_3)_2CO$)

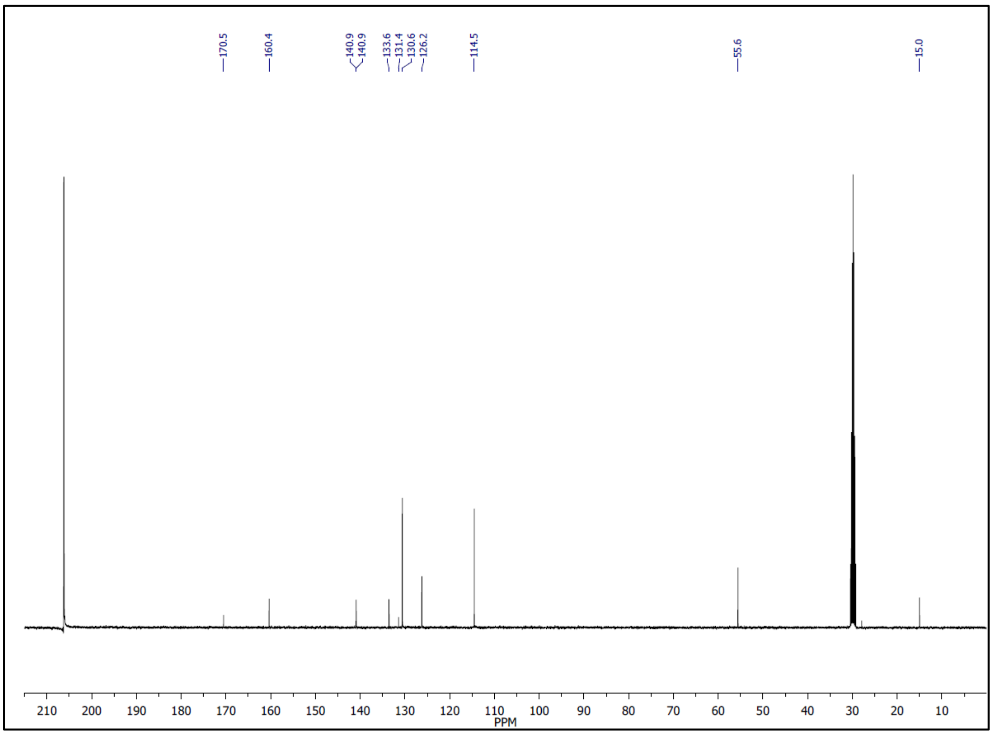

5'-(dimethylamino)-4,4''-dimethoxy-[1,1':3',1''-terphenyl]-2'-carboxylic acid (**3va**)

^{1}H NMR ($(CD_3)_2CO$)

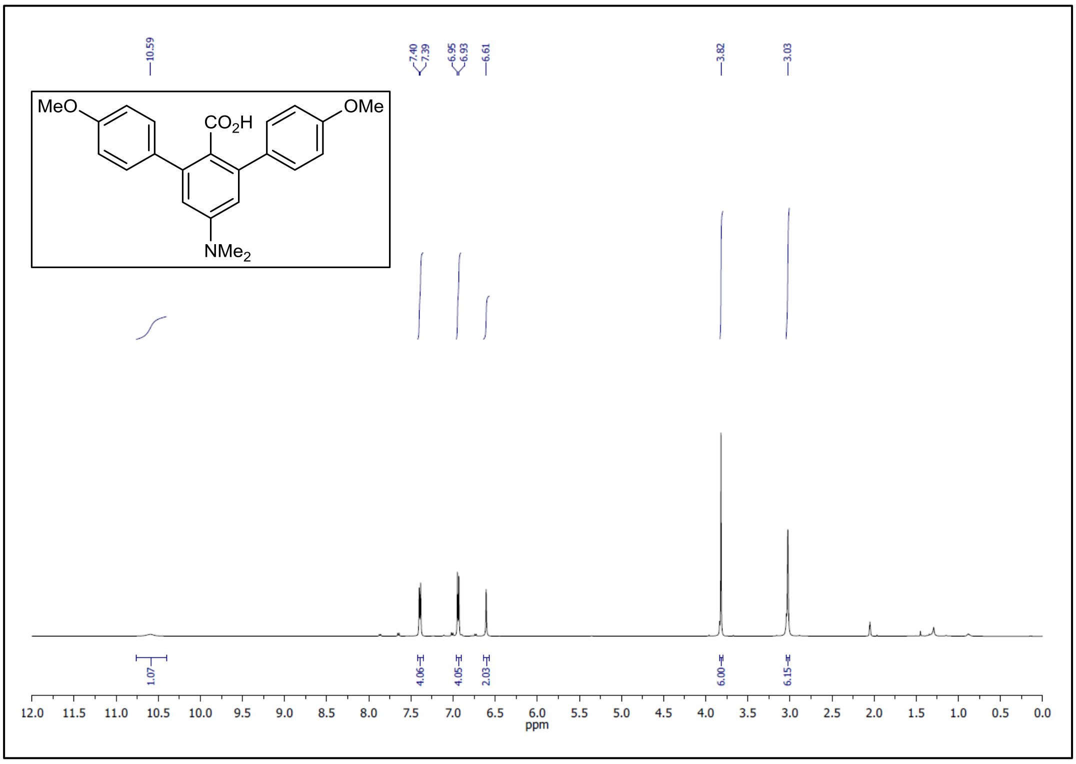

^{13}C NMR ($(CD_3)_2CO$)

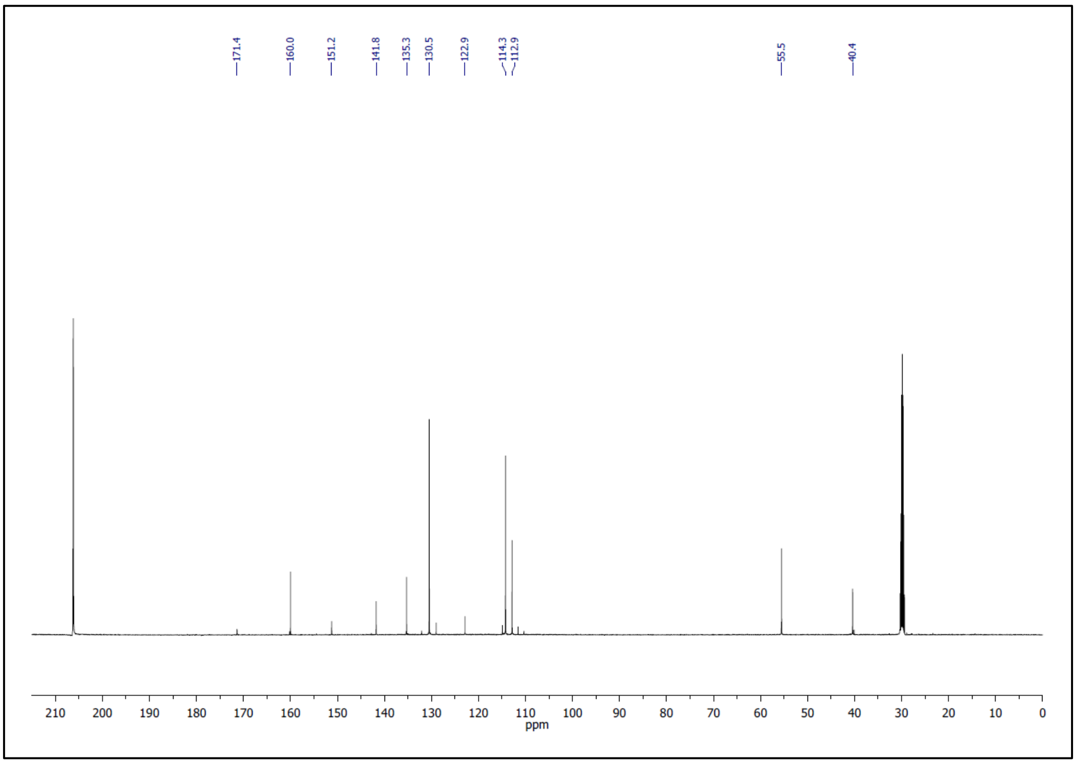

2,6-bis(4-methoxyphenyl)-4-(trifluoromethyl)benzoic acid (**3wa**)

^1H NMR ($CDCl_3$)

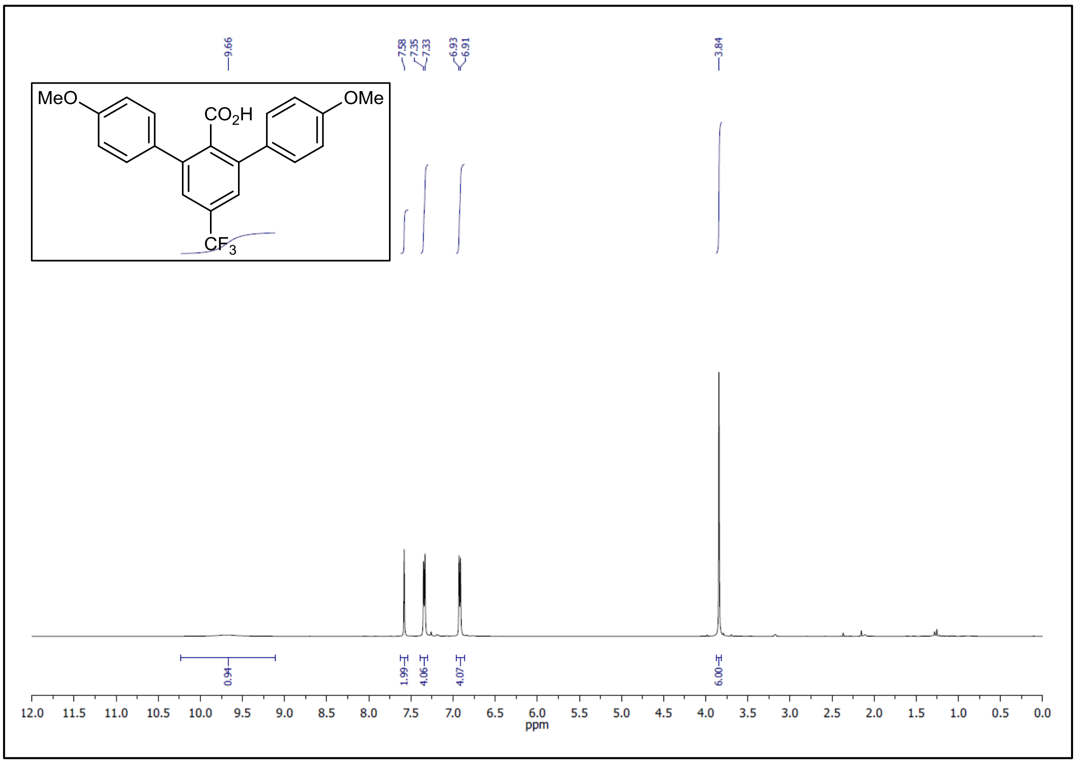

^{13}C NMR ($CDCl_3$)

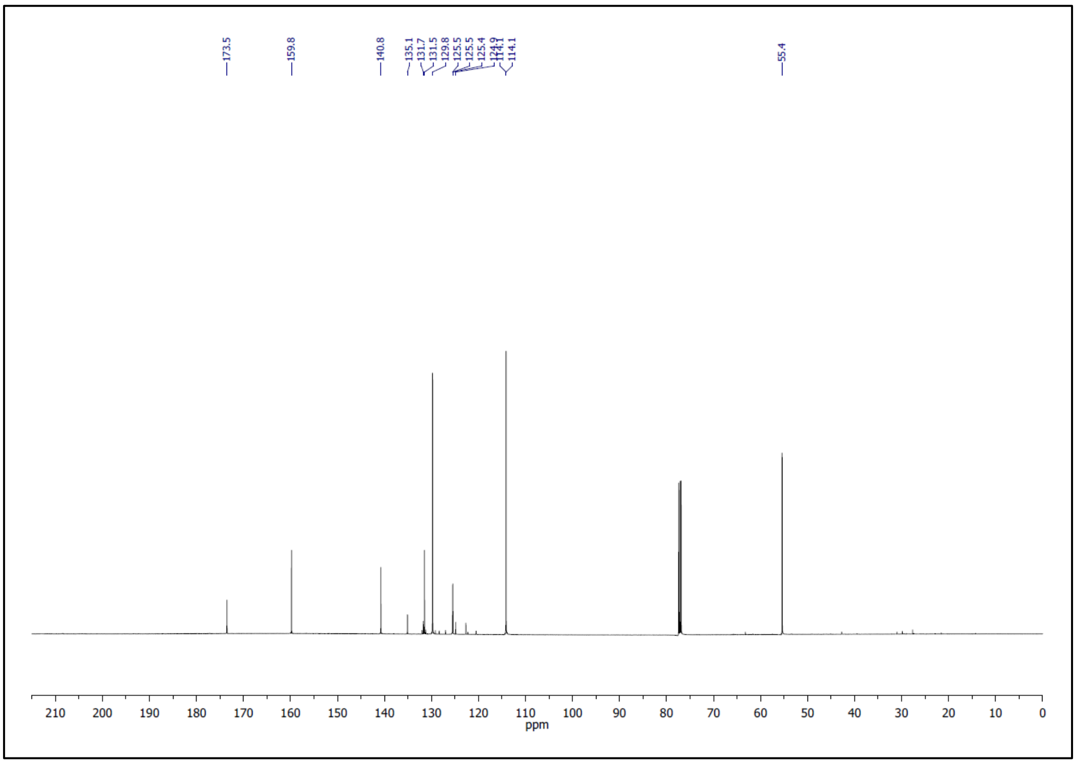

^{19}F NMR ($CDCl_3$)

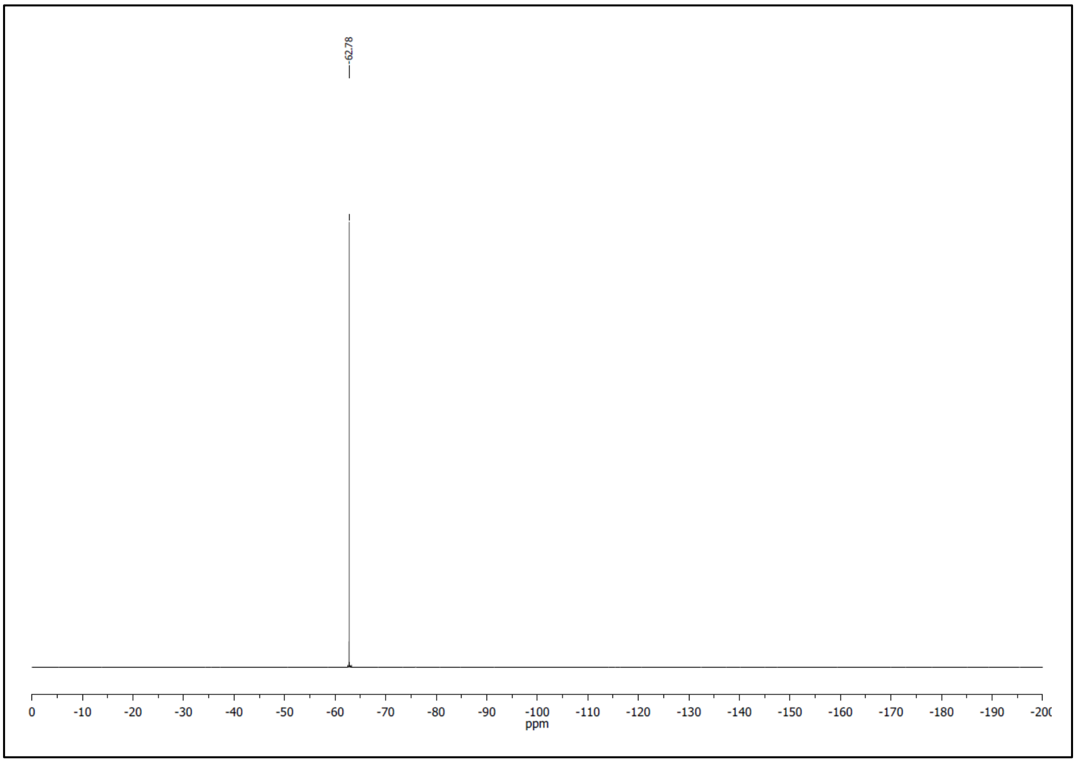

5'-chloro-4,4''-dimethoxy-[1,1':3',1''-terphenyl]-2'-carboxylic acid (**3xa**)

^1H NMR ($(CD_3)_2CO$)

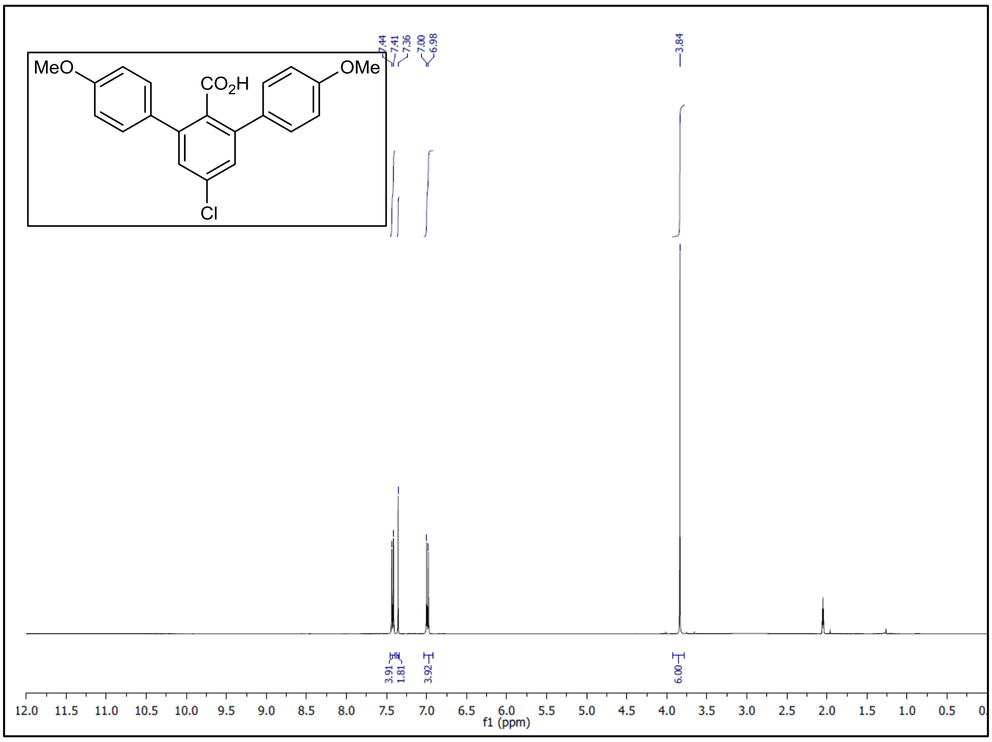

^{13}C NMR ($(CD_3)_2CO$)

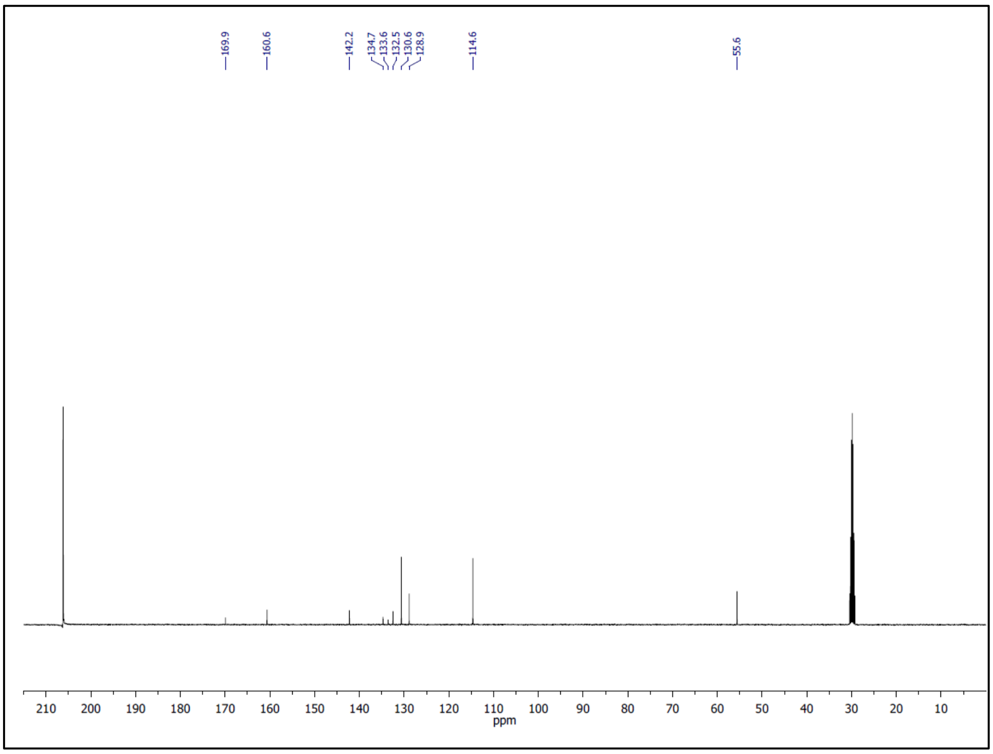

5'-bromo-4,4''-dimethoxy-[1,1':3',1''-terphenyl]-2'-carboxylic acid (**3ya**)

^{1}H NMR ($(CD_3)_2CO$)

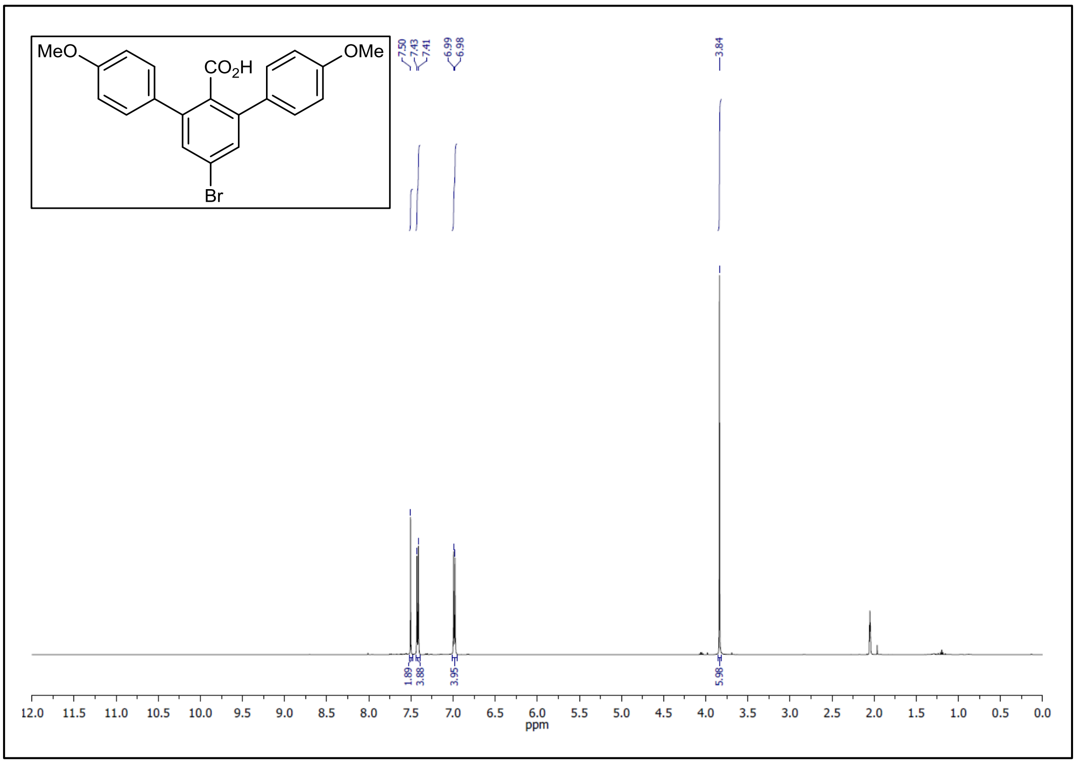

^{13}C NMR ($(CD_3)_2CO$)

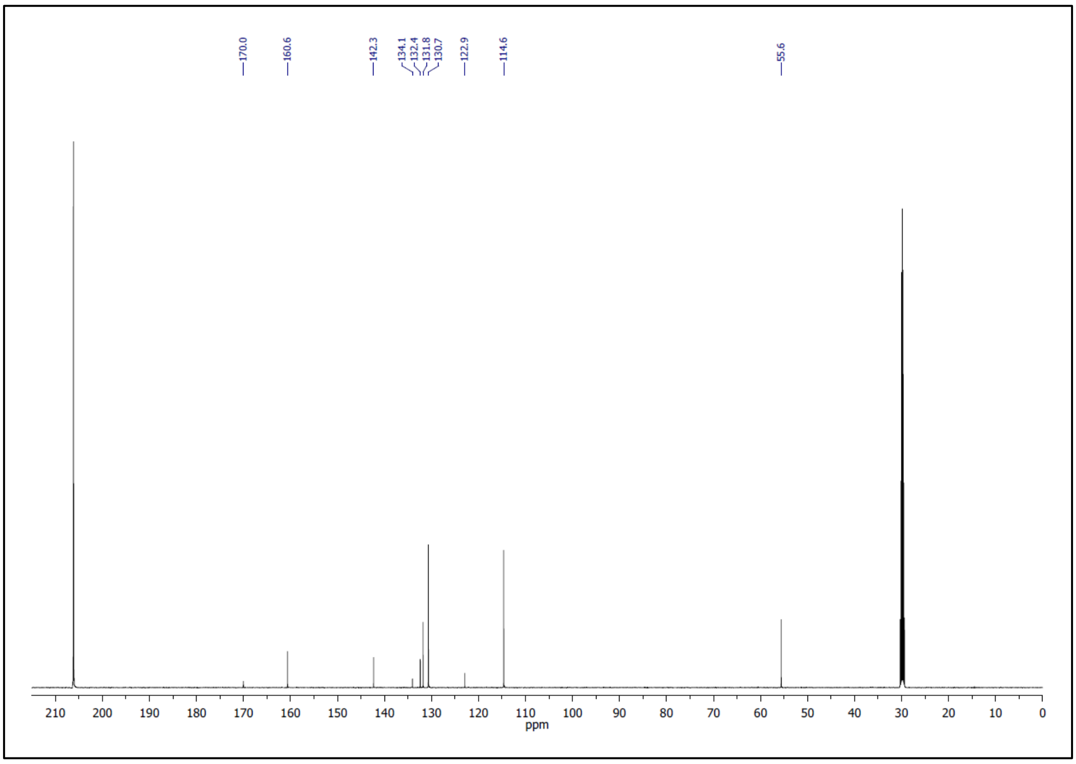

5'-iodo-4,4''-dimethoxy-[1,1':3',1''-terphenyl]-2'-carboxylic acid (**3za**)

^{1}H NMR ($(CD_3)_2CO$)

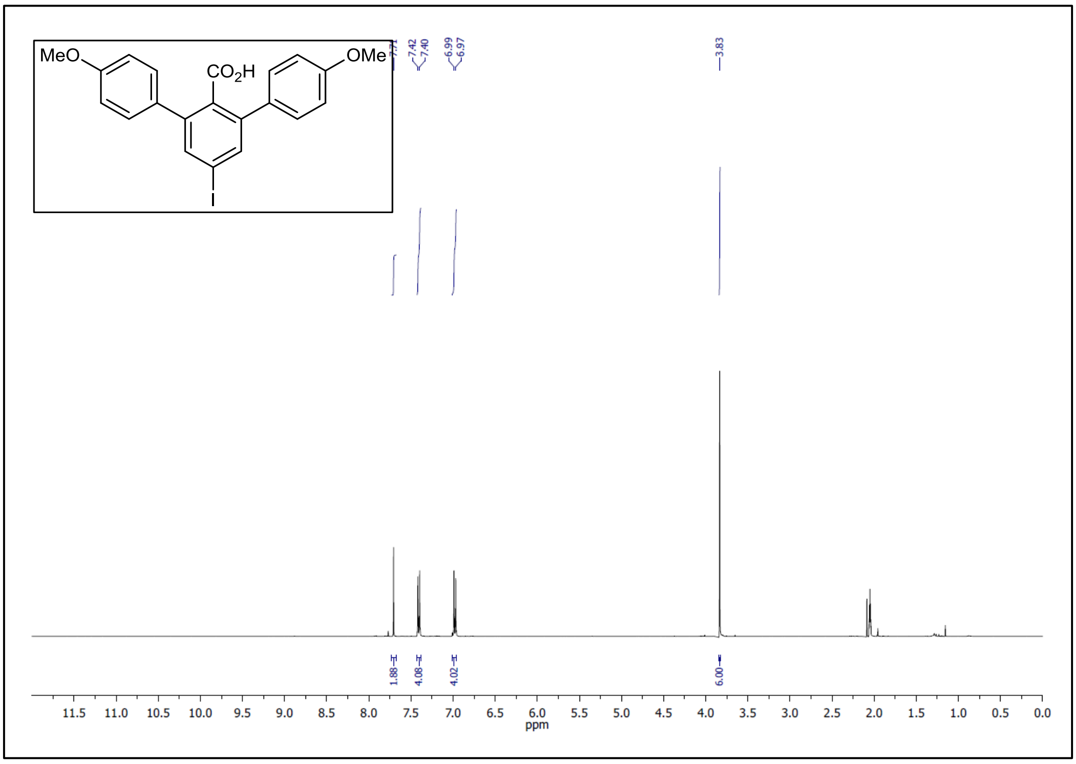

^{13}C NMR ($(CD_3)_2CO$)

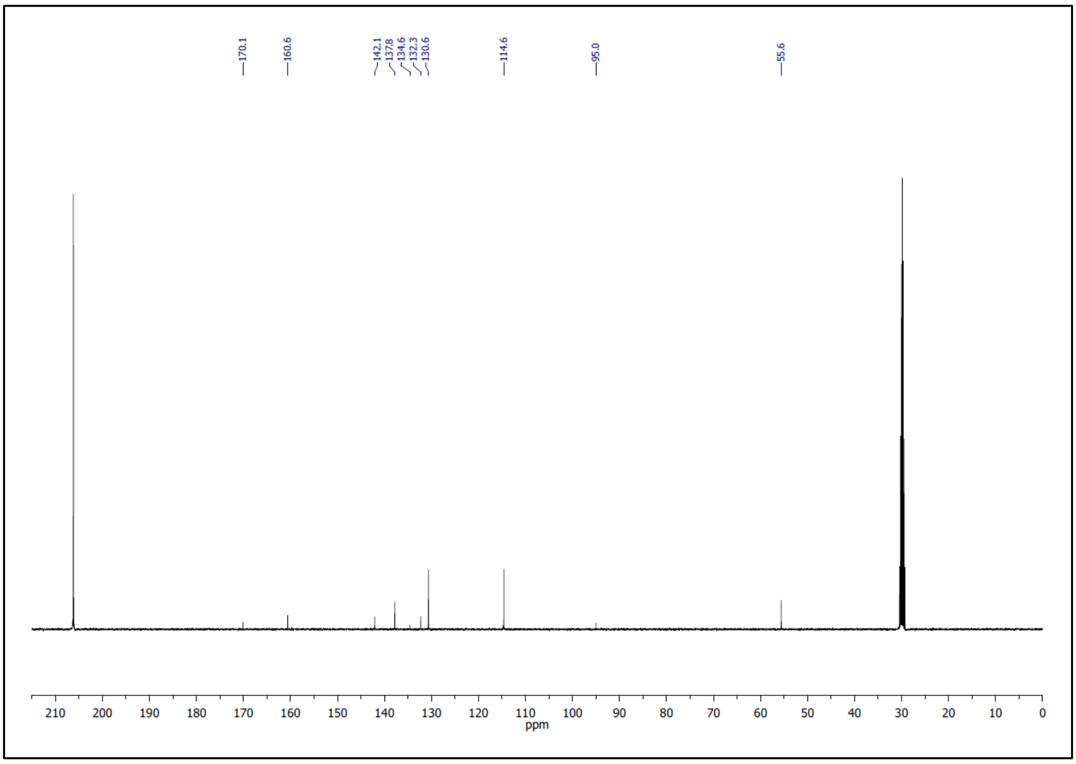

6-(4-methoxyphenyl)-1-methyl-1H-indole-7-carboxylic acid (**5aa**)

^{1}H NMR ($(CD_3)_2CO$)

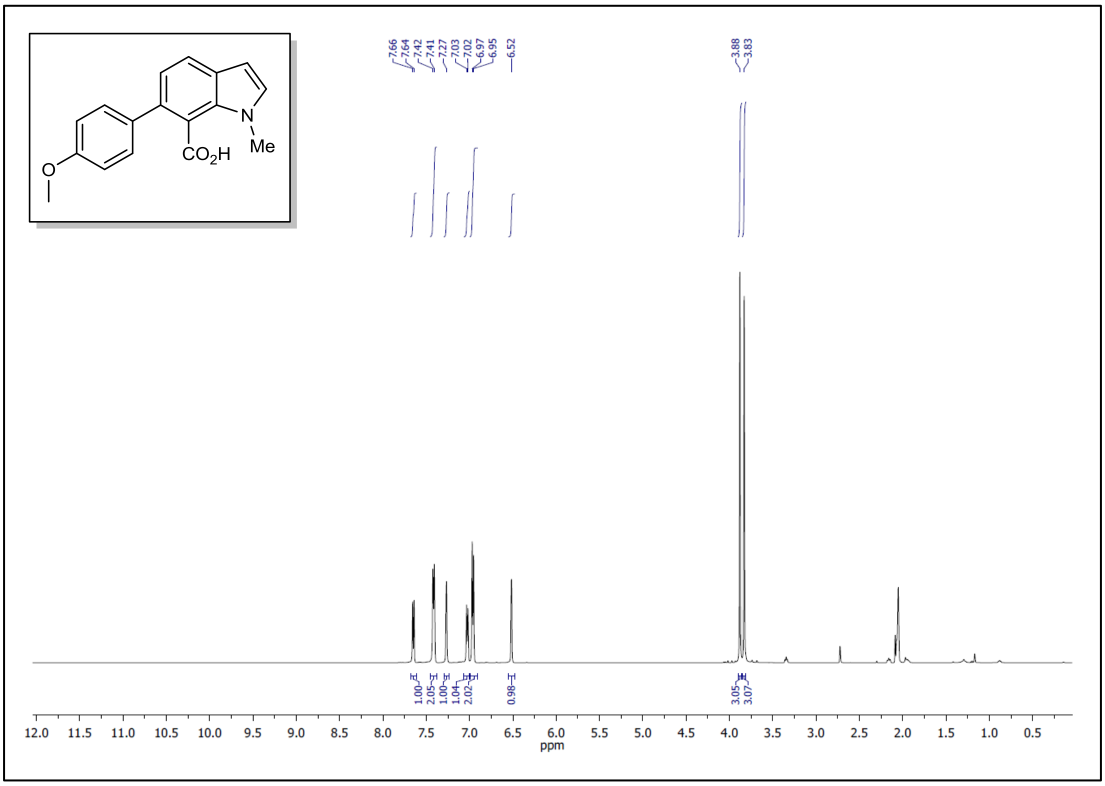

^{13}C NMR ($(CD_3)_2CO$)

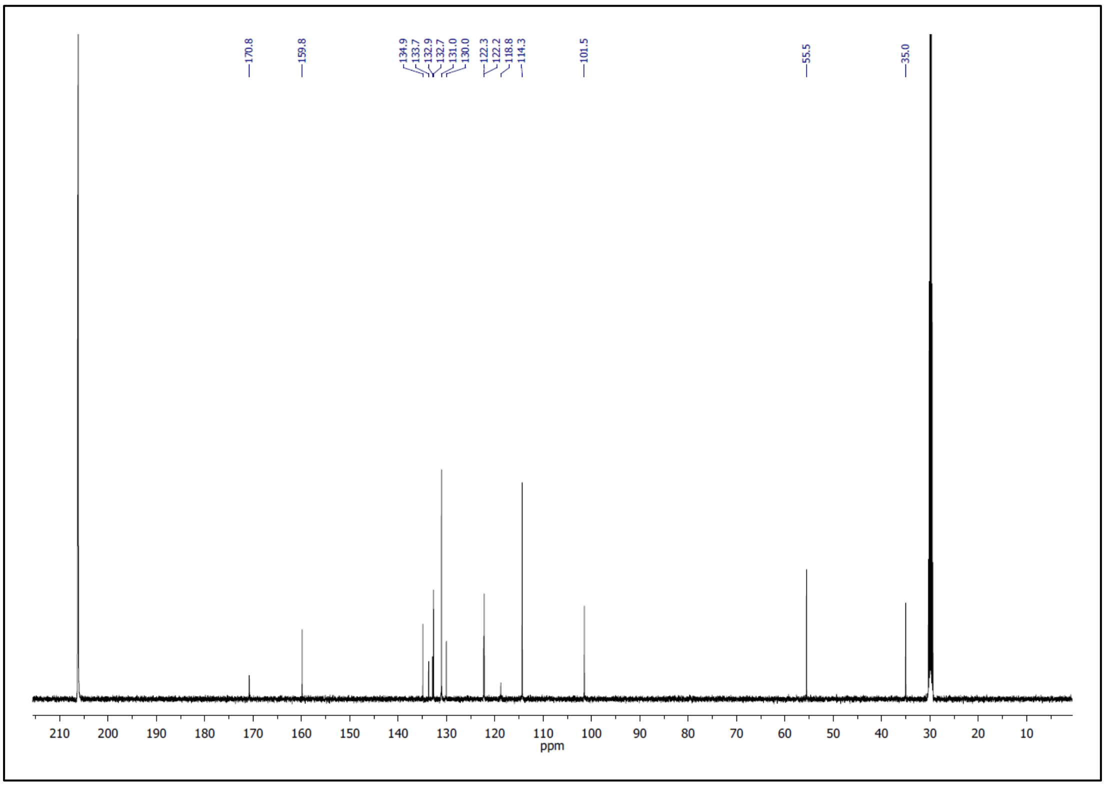

1-methyl-6-phenyl-1H-indole-7-carboxylic acid (**5ae**)

^{1}H NMR ($(CD_3)_2CO$)

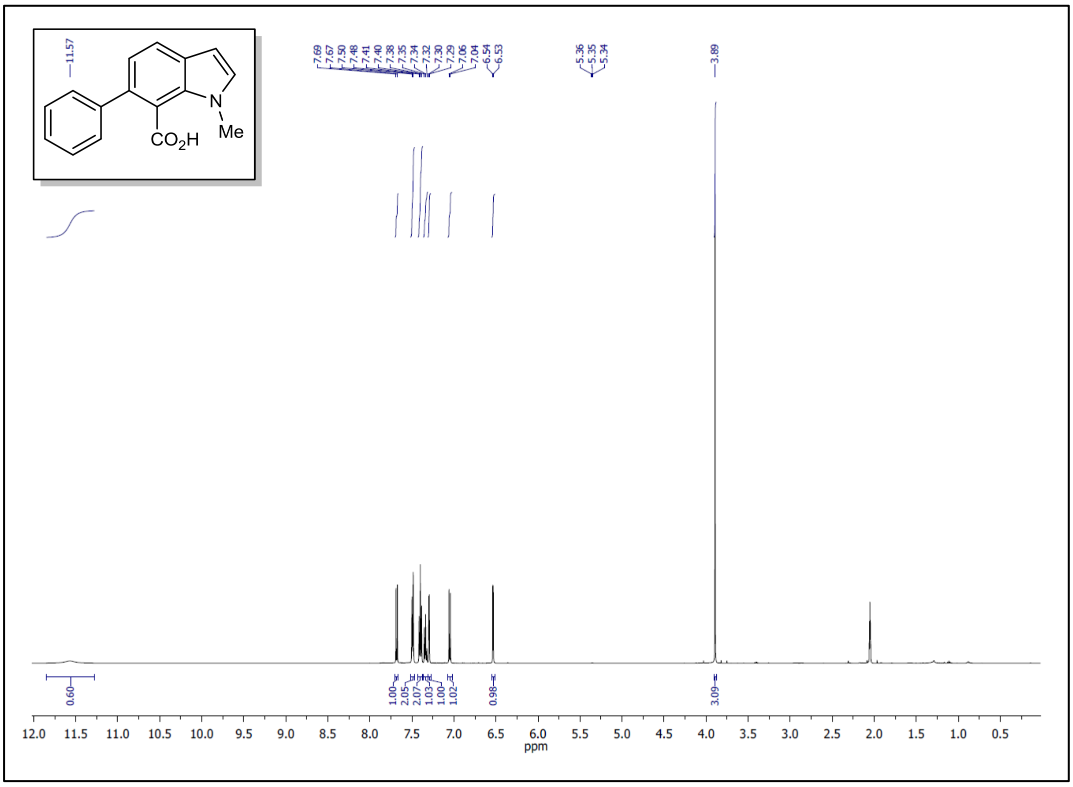

^{13}C NMR ($(CD_3)_2CO$)

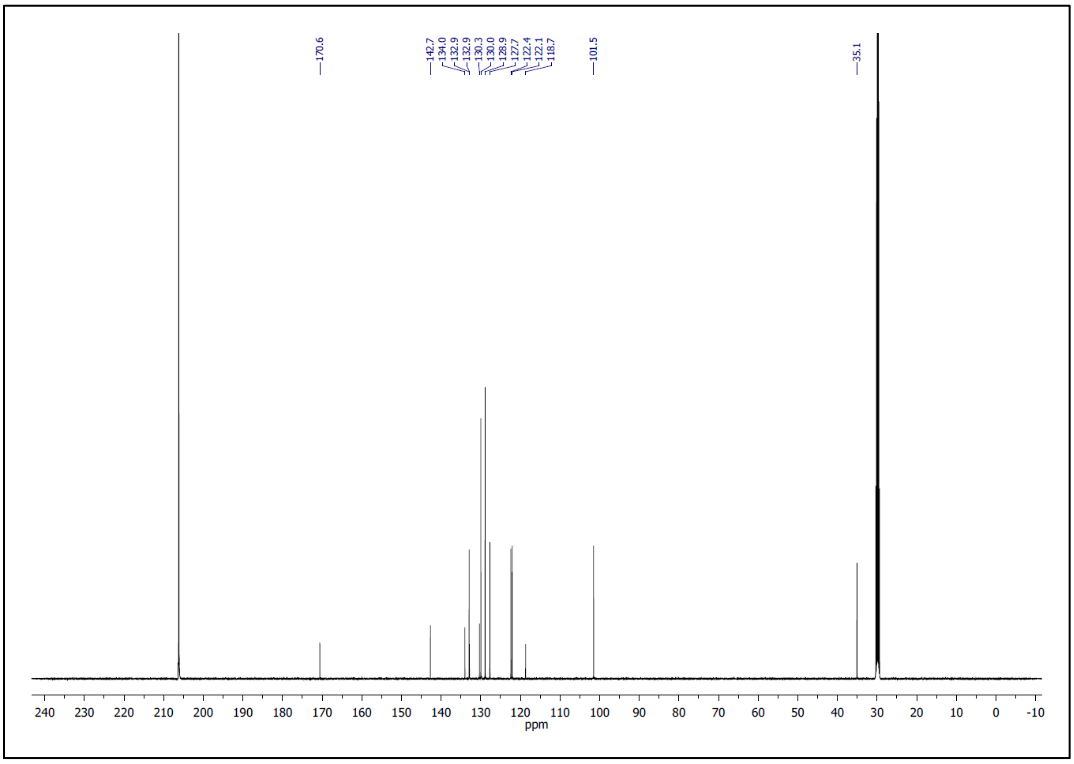

1-methyl-6-(p-tolyl)-1H-indole-7-carboxylic acid (**5af**)

^{1}H NMR ($(CD_3)_2CO$)

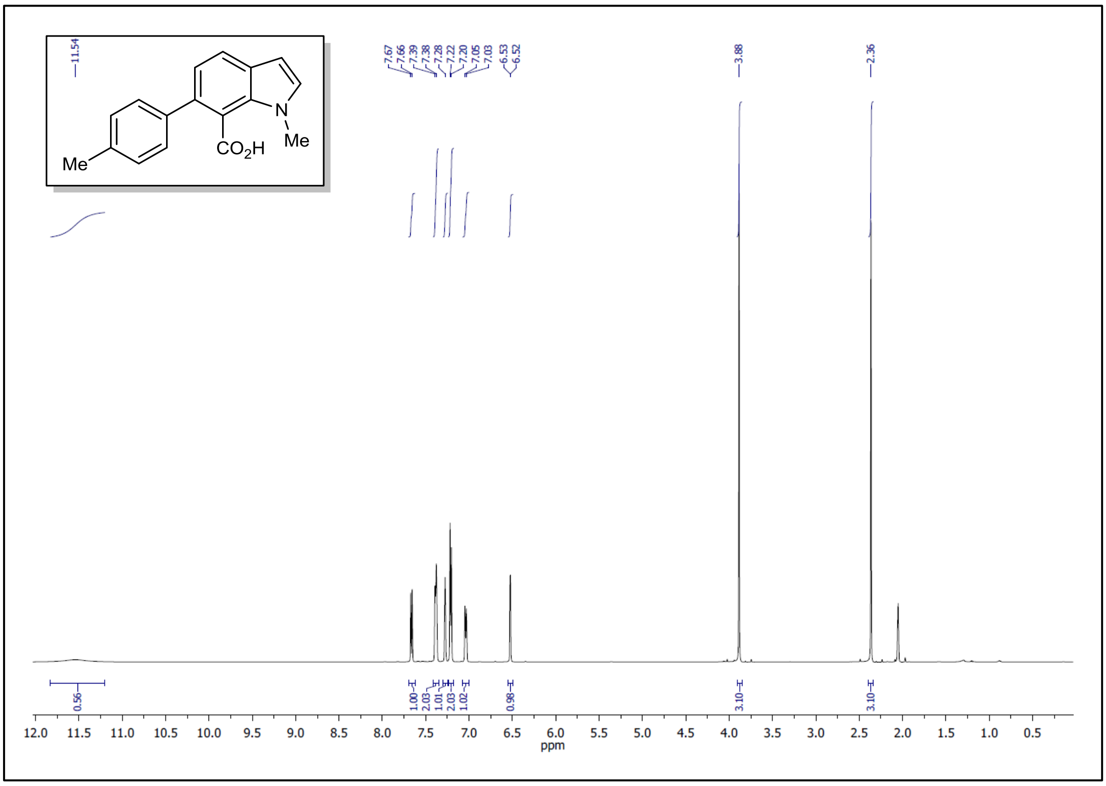

^{13}C NMR ($(CD_3)_2CO$)

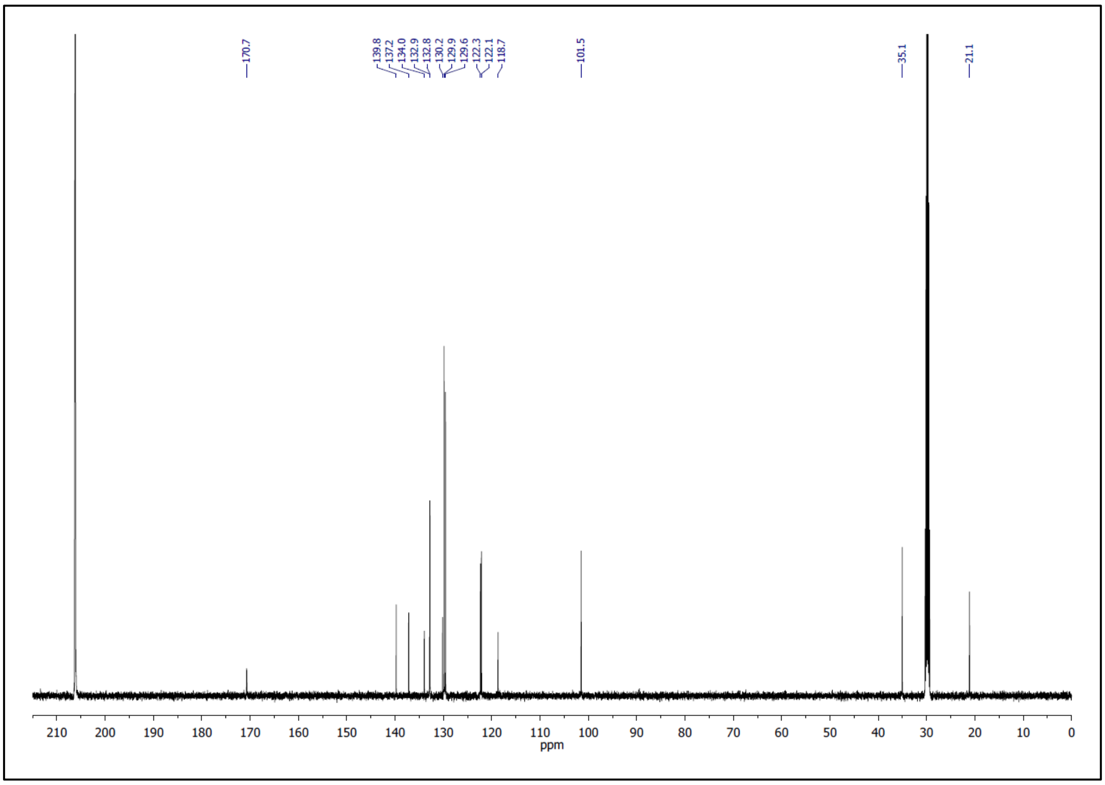

1-methyl-6-(4-(trifluoromethyl)phenyl)-1H-indole-7-carboxylic acid (**5ao**)

^{1}H NMR ($(CD_3)_2CO$)

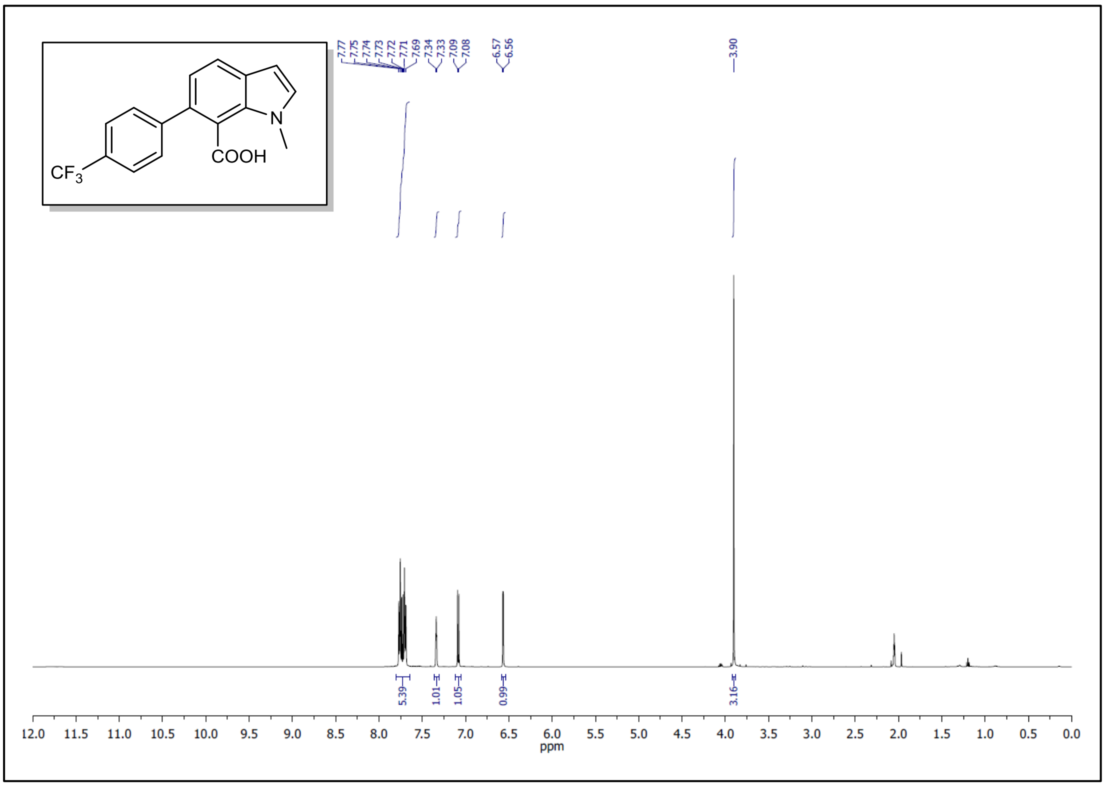

^{13}C NMR ($(CD_3)_2CO$)

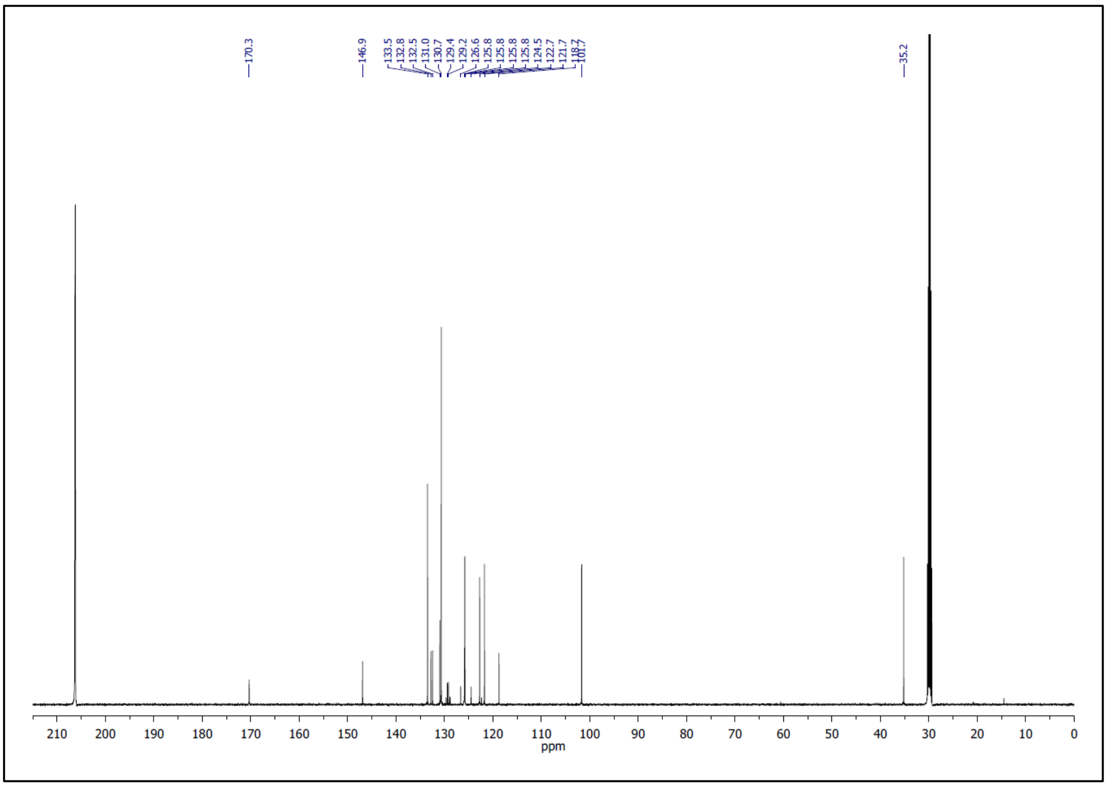

^{19}F NMR ($(CD_3)_2CO$)

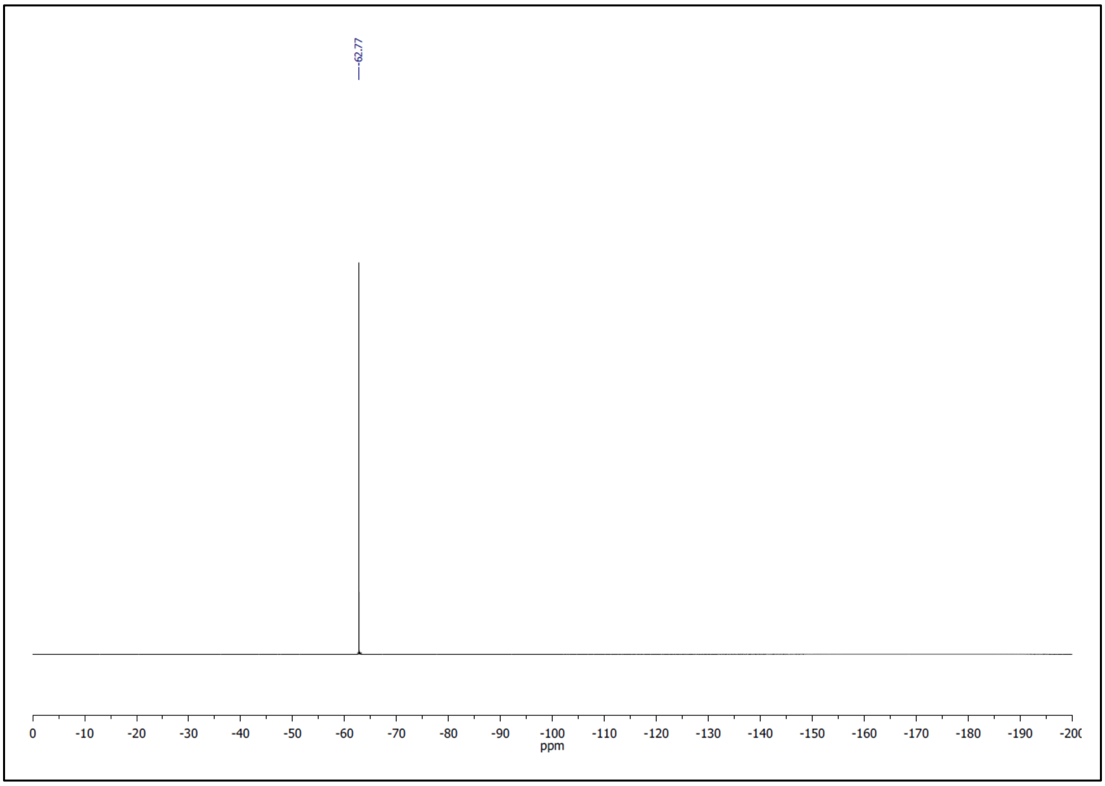

6-(4-fluorophenyl)-1-methyl-1H-indole-7-carboxylic acid (**5ai**)

^{1}H NMR ($(CD_3)_2CO$)

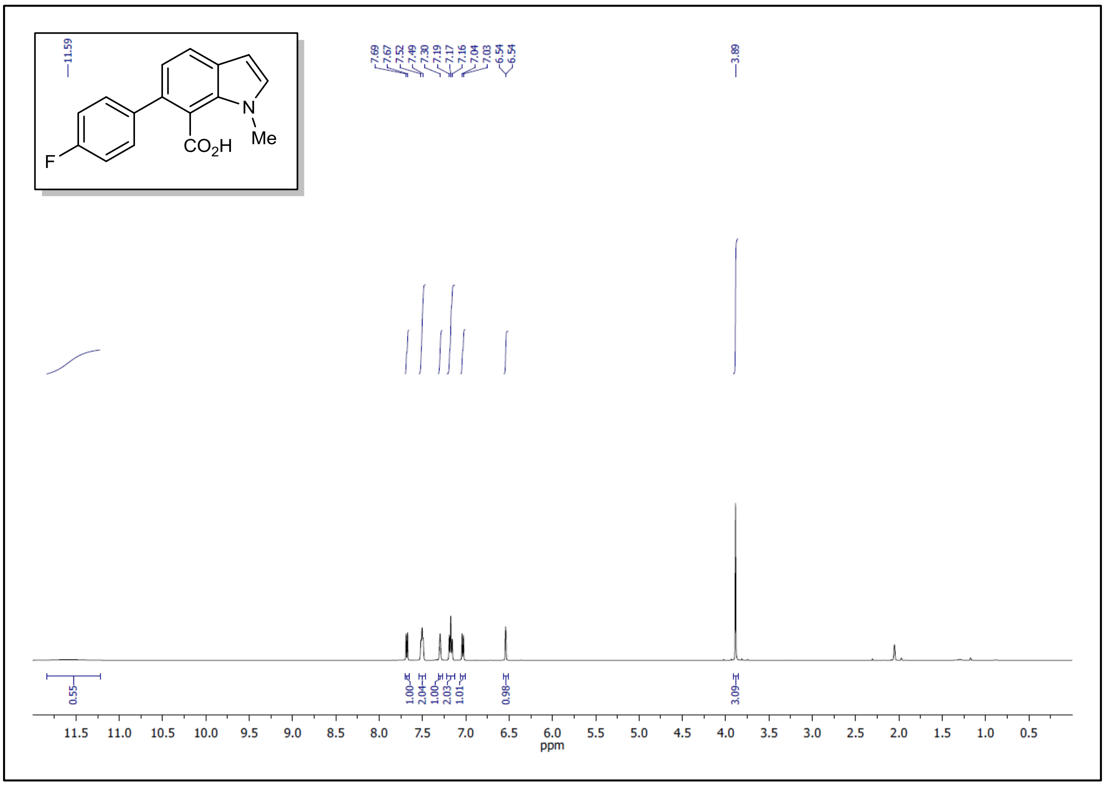

^{13}C NMR ($(CD_3)_2CO$)

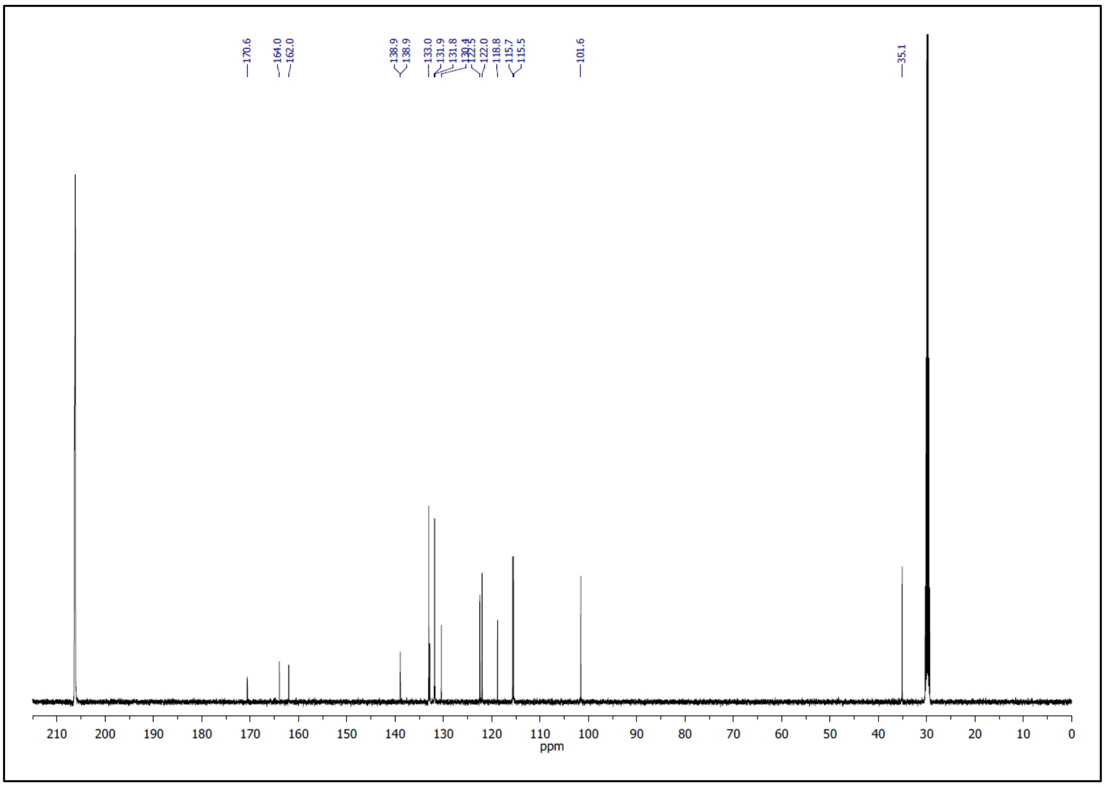

^{19}F NMR ($(CD_3)_2CO$)

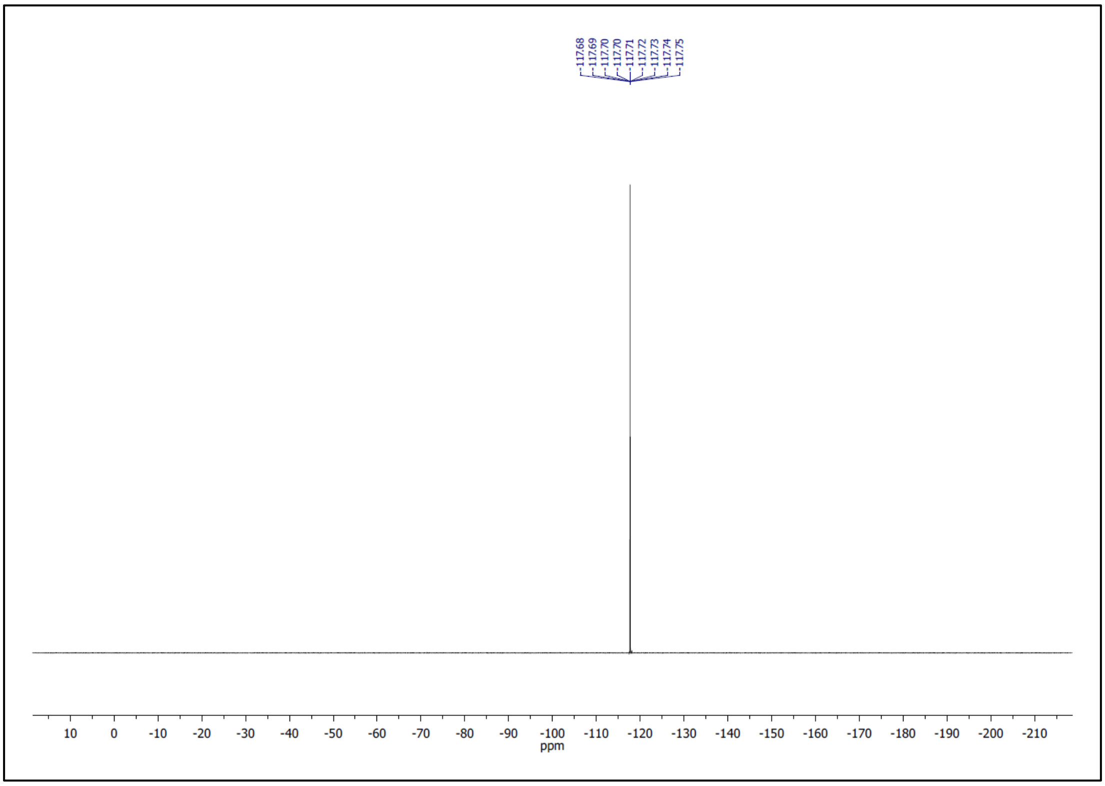

6-(4-chlorophenyl)-1-methyl-1H-indole-7-carboxylic acid (**5aj**)

^{1}H NMR ($(CD_3)_2CO$)

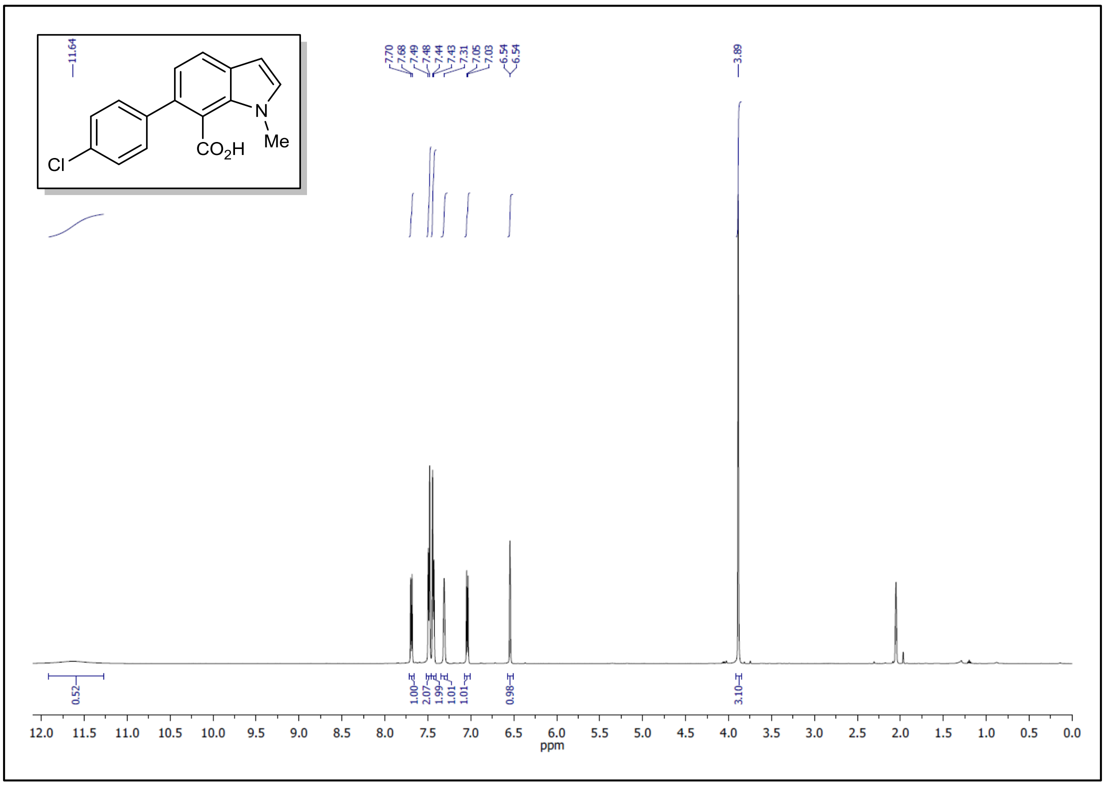

^{13}C NMR ($(CD_3)_2CO$)

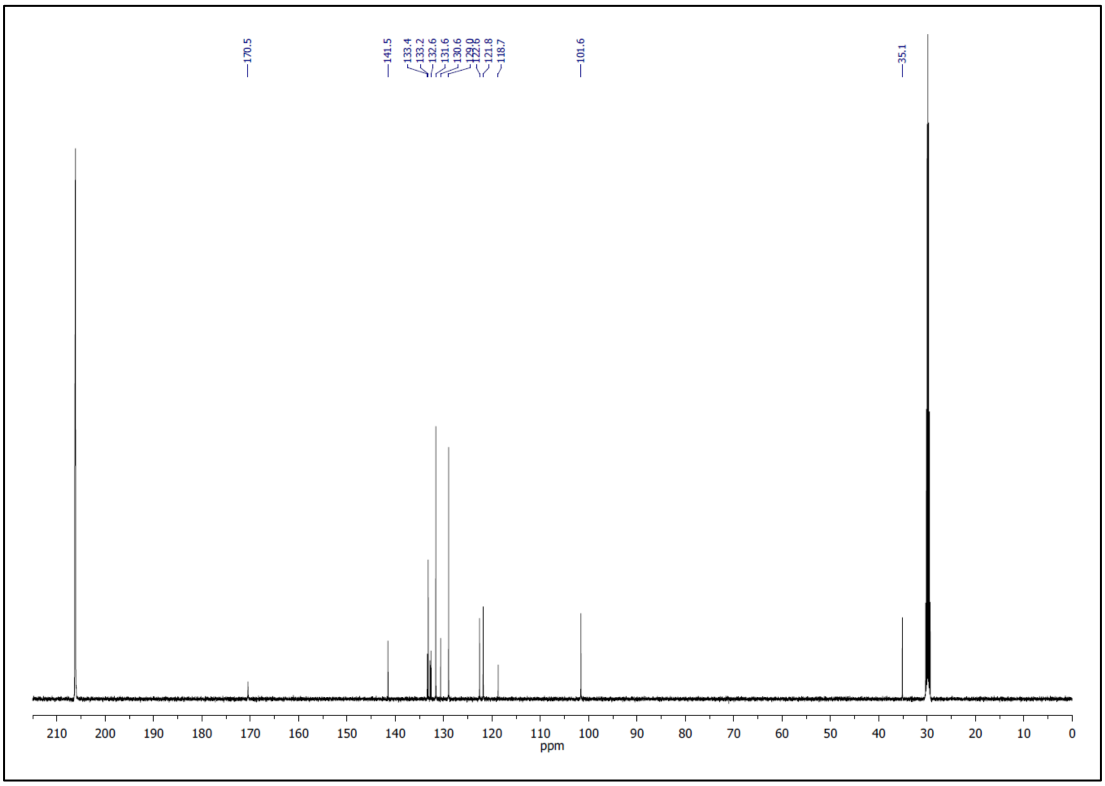

6-(4-bromophenyl)-1-methyl-1H-indole-7-carboxylic acid (**5ak**)

^{1}H NMR ($(CD_3)_2CO$)

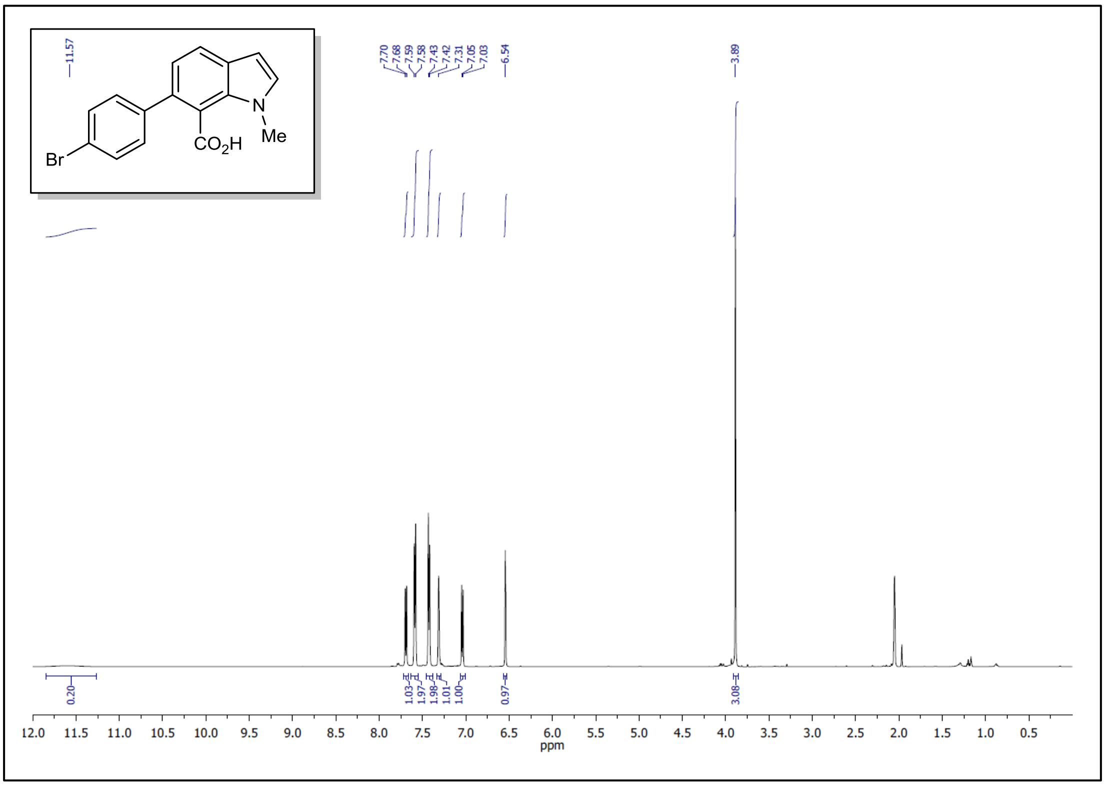

^{13}C NMR ($(CD_3)_2CO$)

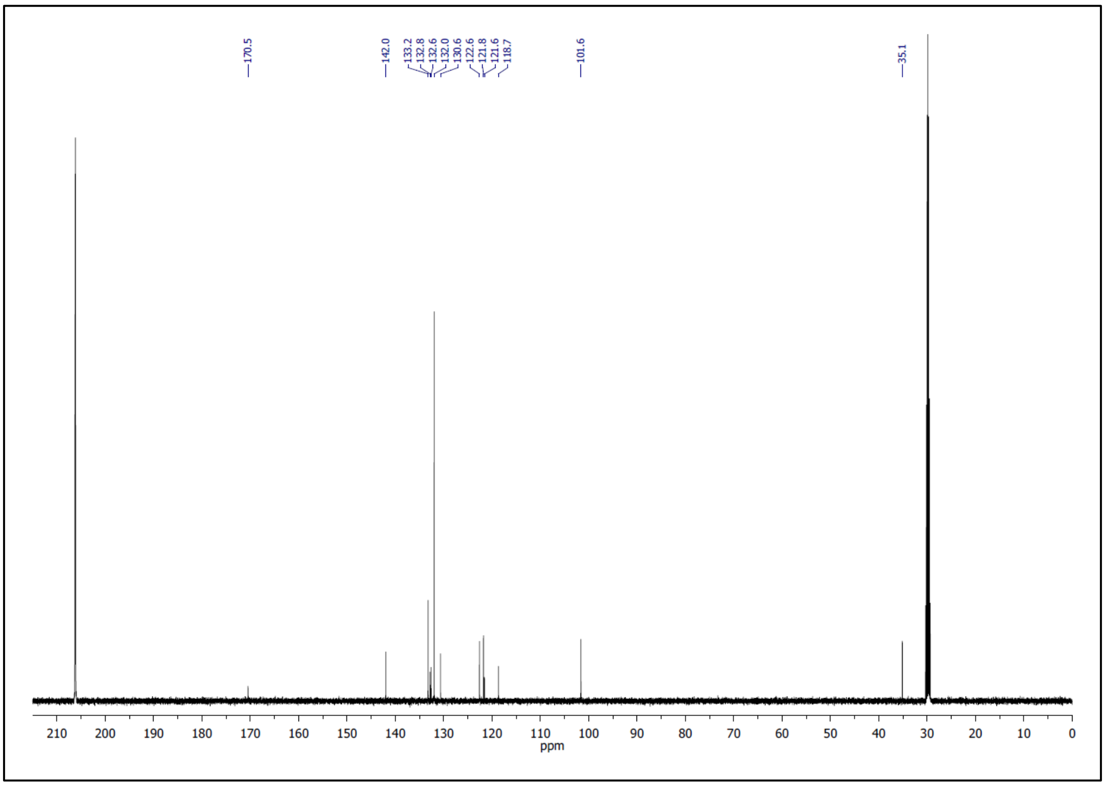

1-methyl-6-(2-phenoxyphenyl)-1H-indole-7-carboxylic acid (**5ad**)

^{1}H NMR ($CDCl_3$)

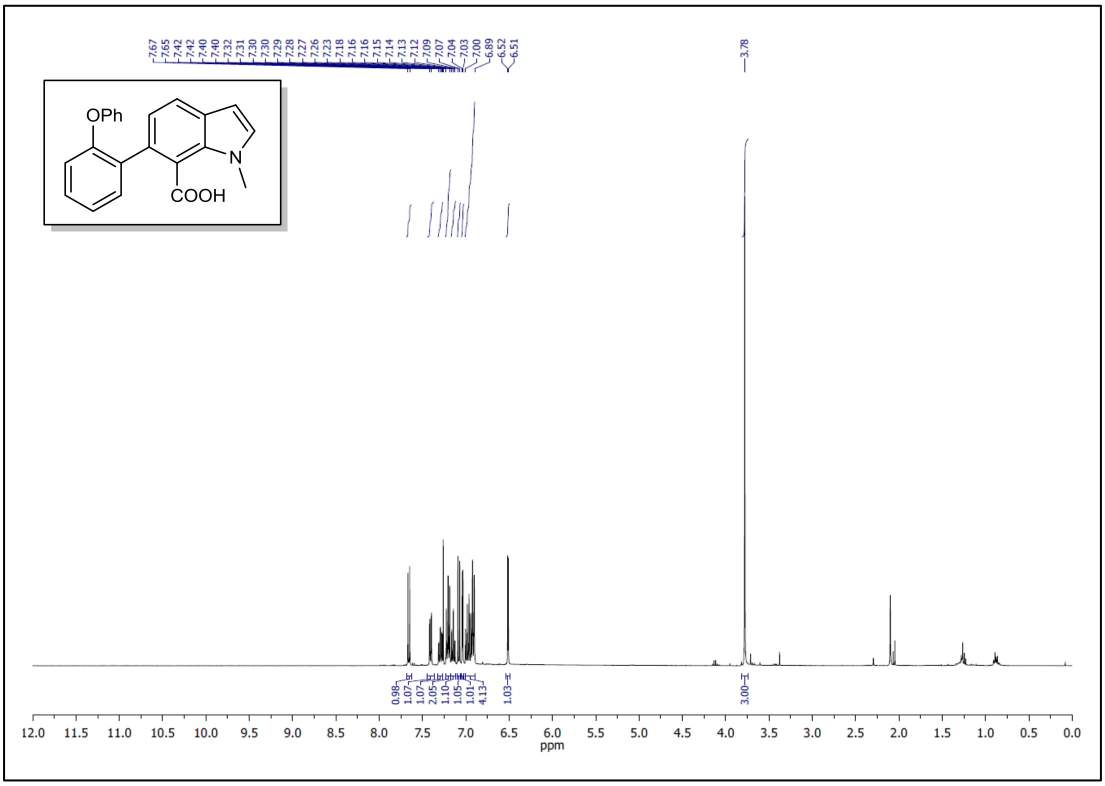

^{13}C NMR ($CDCl_3$)

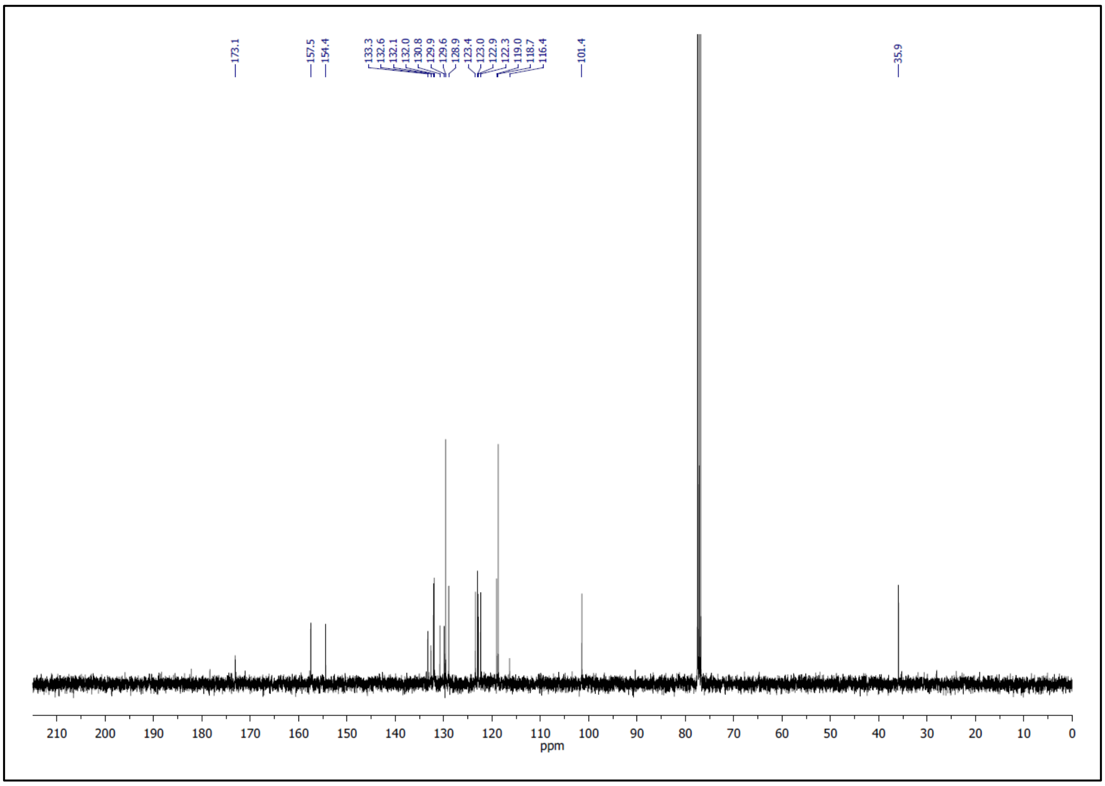

6-(2-fluorophenyl)-1-methyl-1H-indole-7-carboxylic acid (**5am**)

^{1}H NMR ($(CD_3)_2CO$)

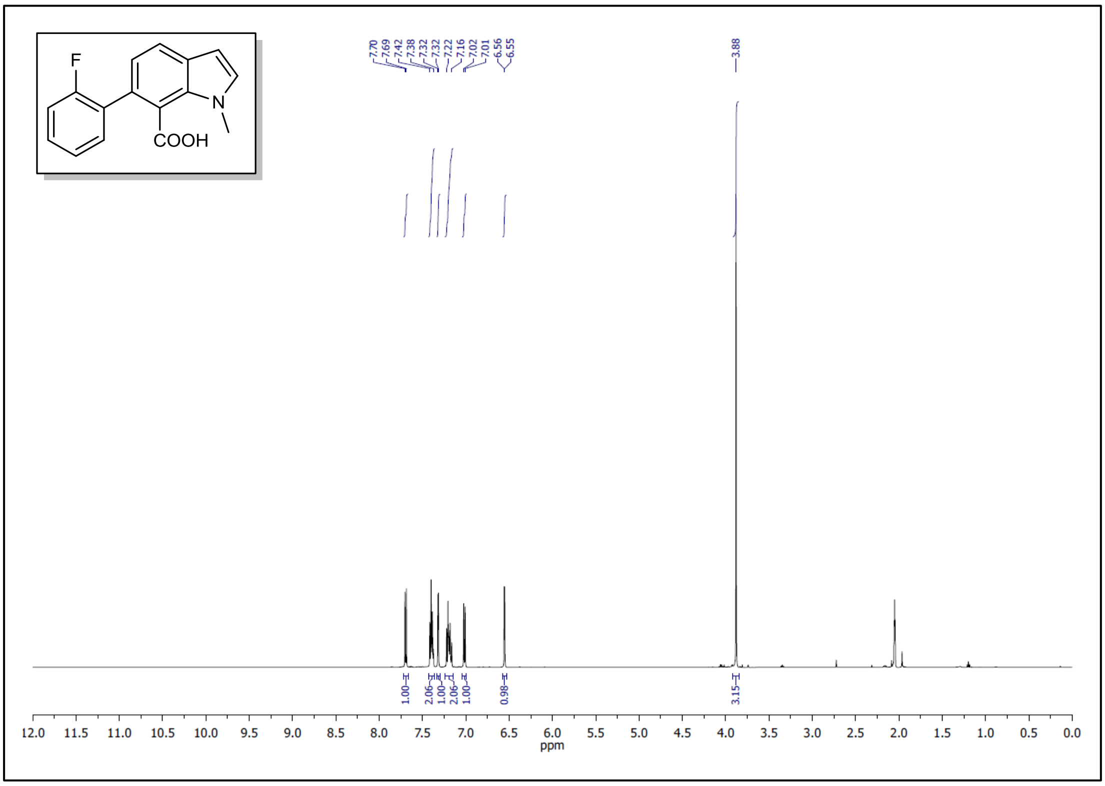

^{13}C NMR ($(CD_3)_2CO$)

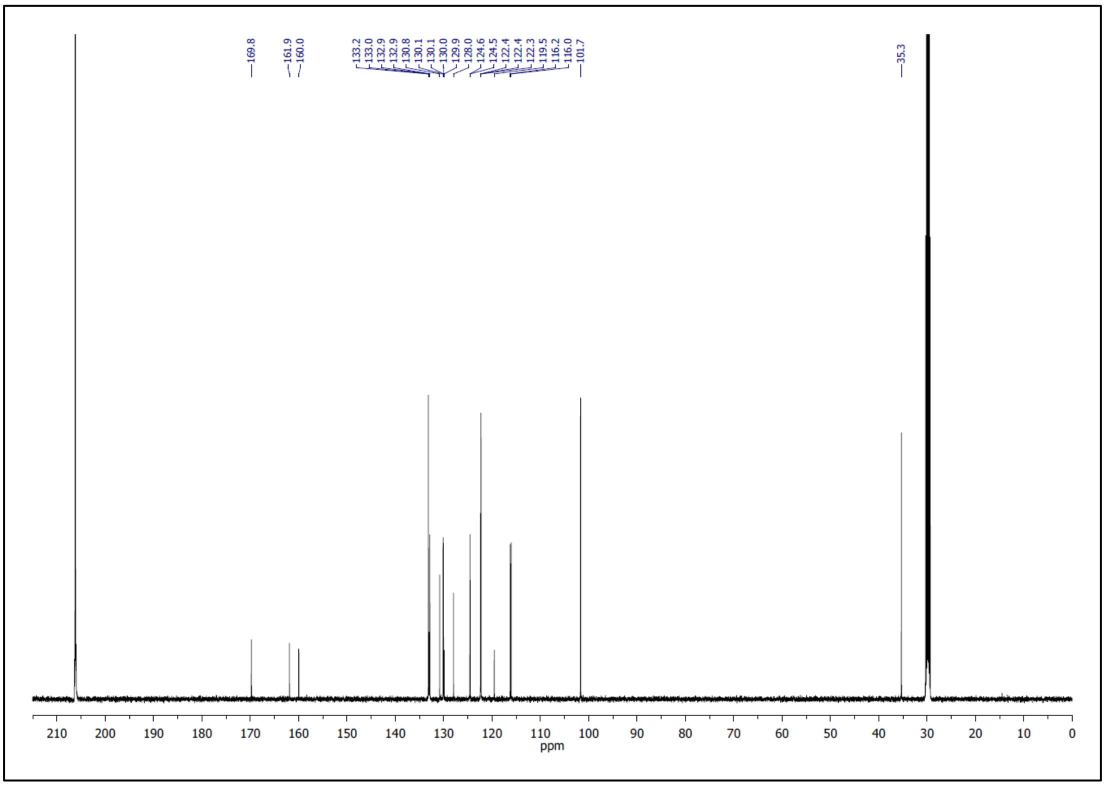

^{19}F NMR ($(CD_3)_2CO$)

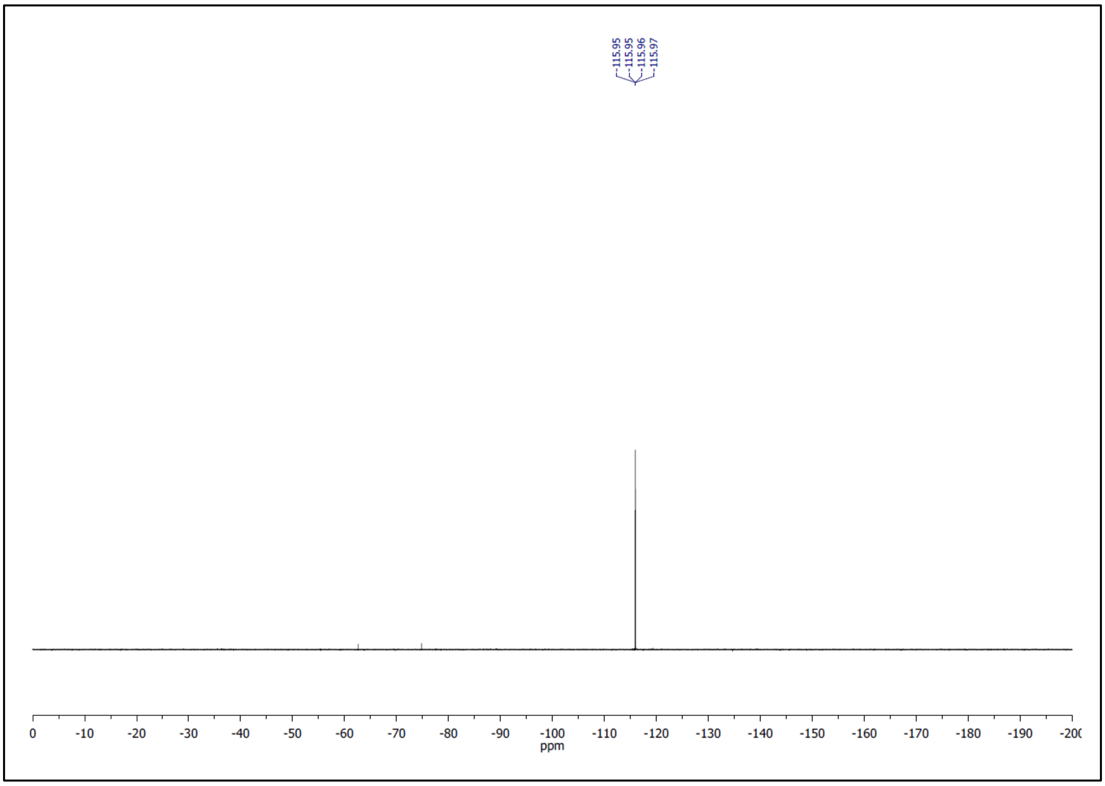

6-(3,5-dimethylphenyl)-1-methyl-1H-indole-7-carboxylic acid (**5aaa**)

^{1}H NMR ($(CD_3)_2CO$)

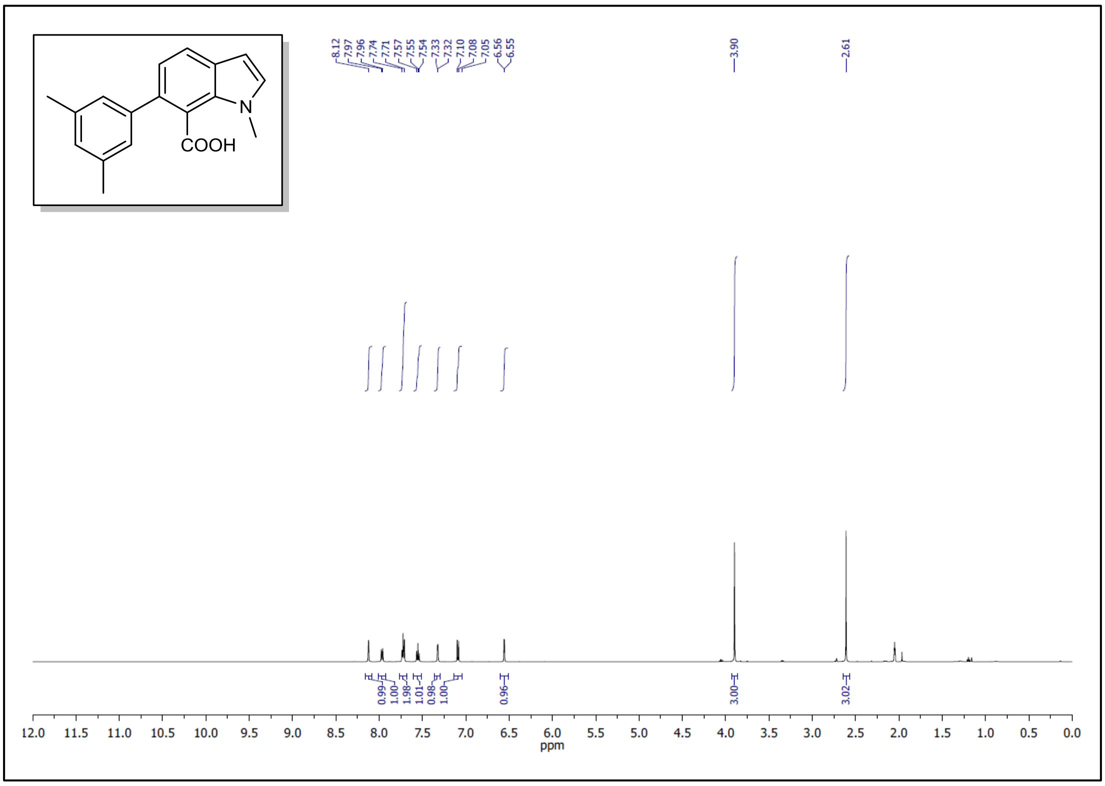

^{13}C NMR ($(CD_3)_2CO$)

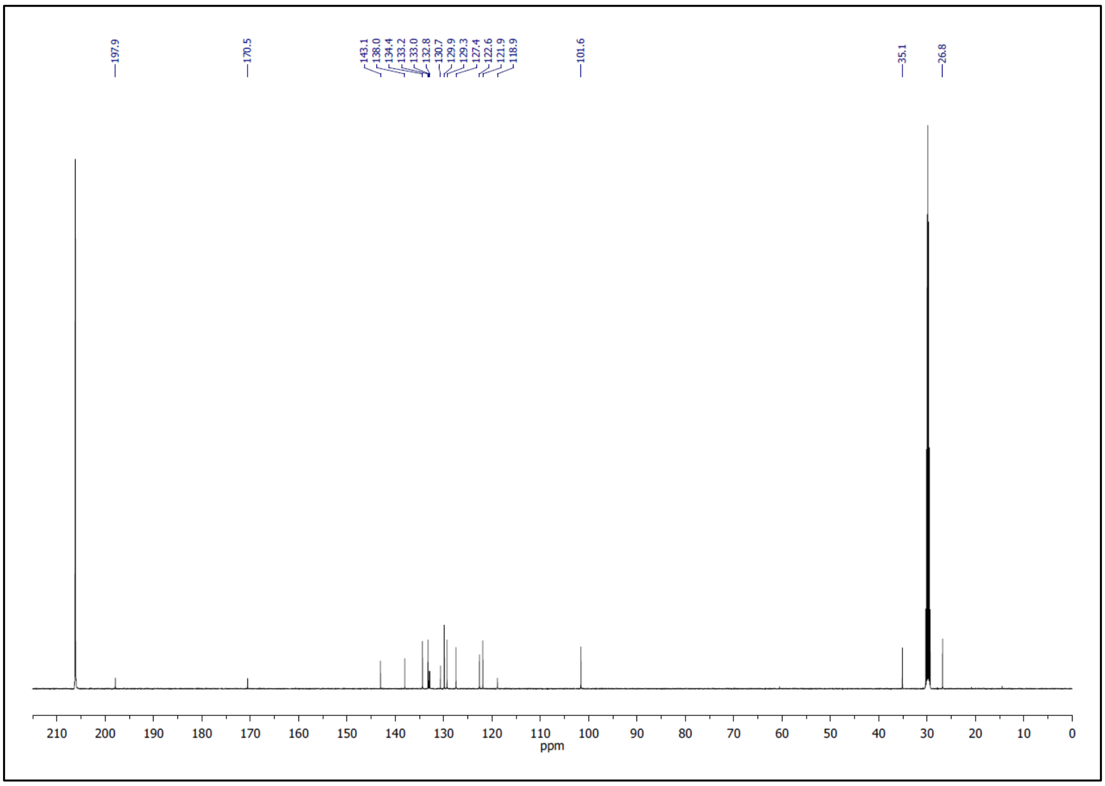

6-(3-(methoxycarbonyl)phenyl)-1-methyl-1H-indole-7-carboxylic acid (**5aba**)

^{1}H NMR ($(CD_3)_2CO$)

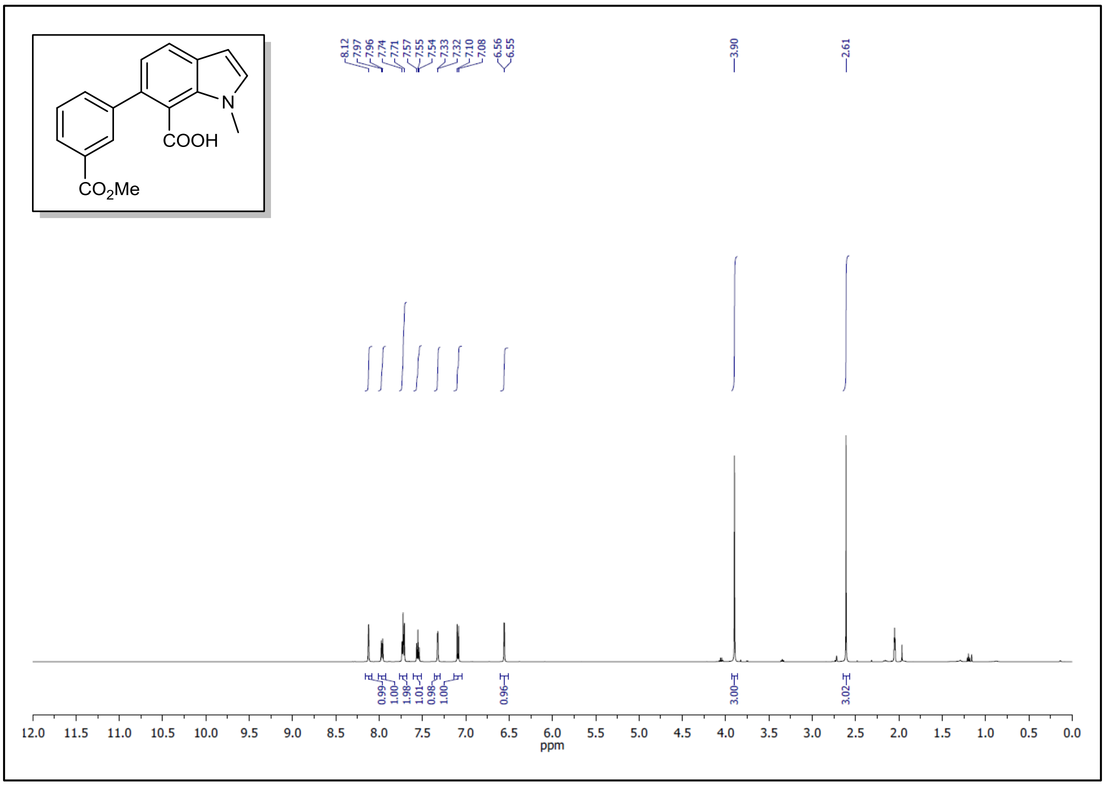

^{13}C NMR ($(CD_3)_2CO$)

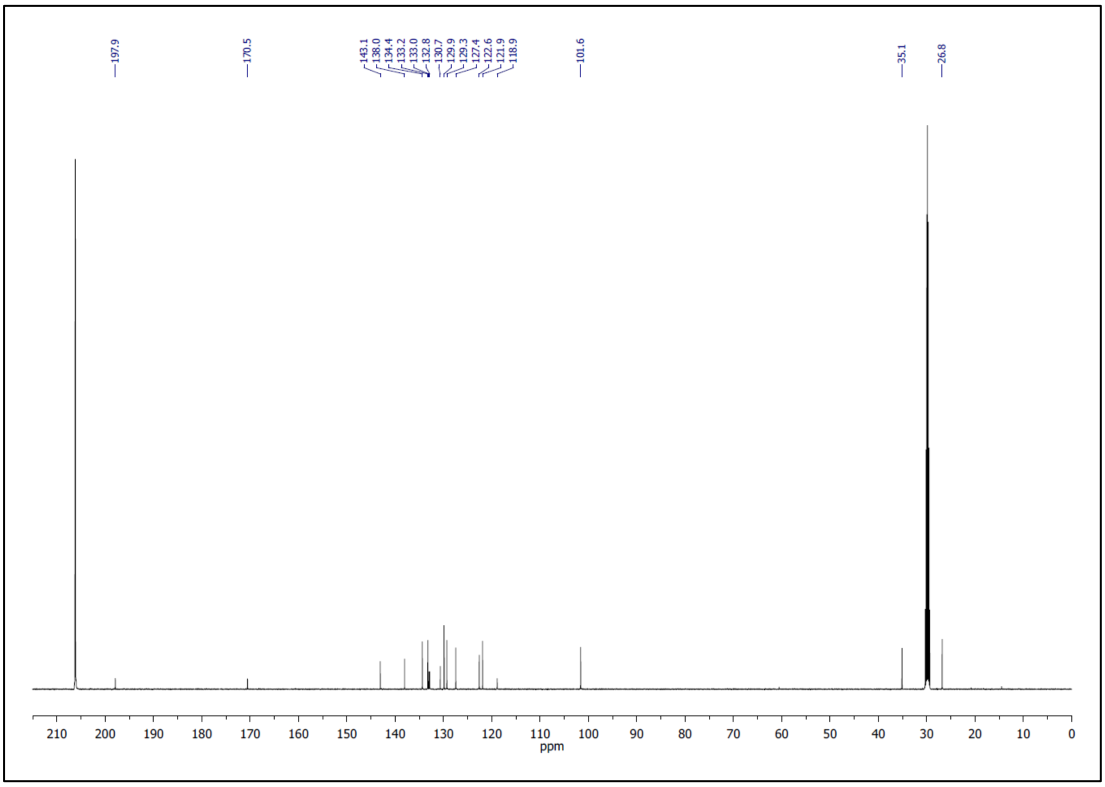

6-(3-acetylphenyl)-1-methyl-1H-indole-7-carboxylic acid (**5ar**)

^{1}H NMR ($(CD_3)_2CO$)

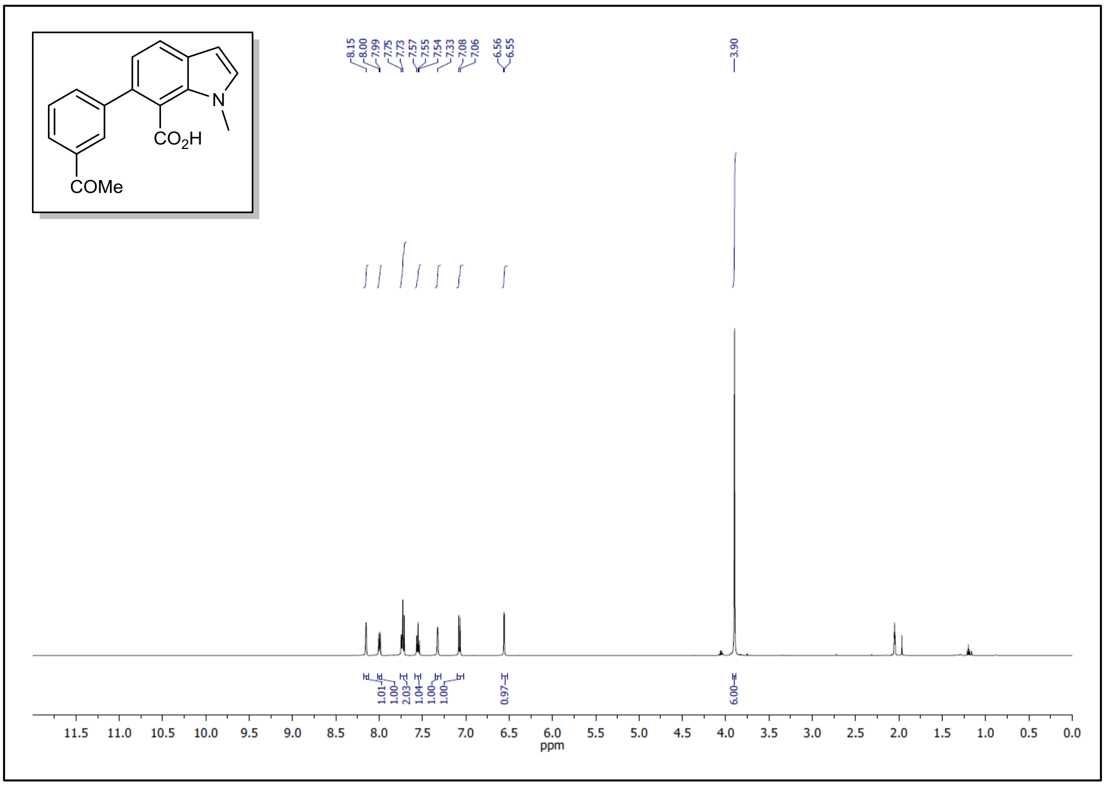

^{13}C NMR ($(CD_3)_2CO$)

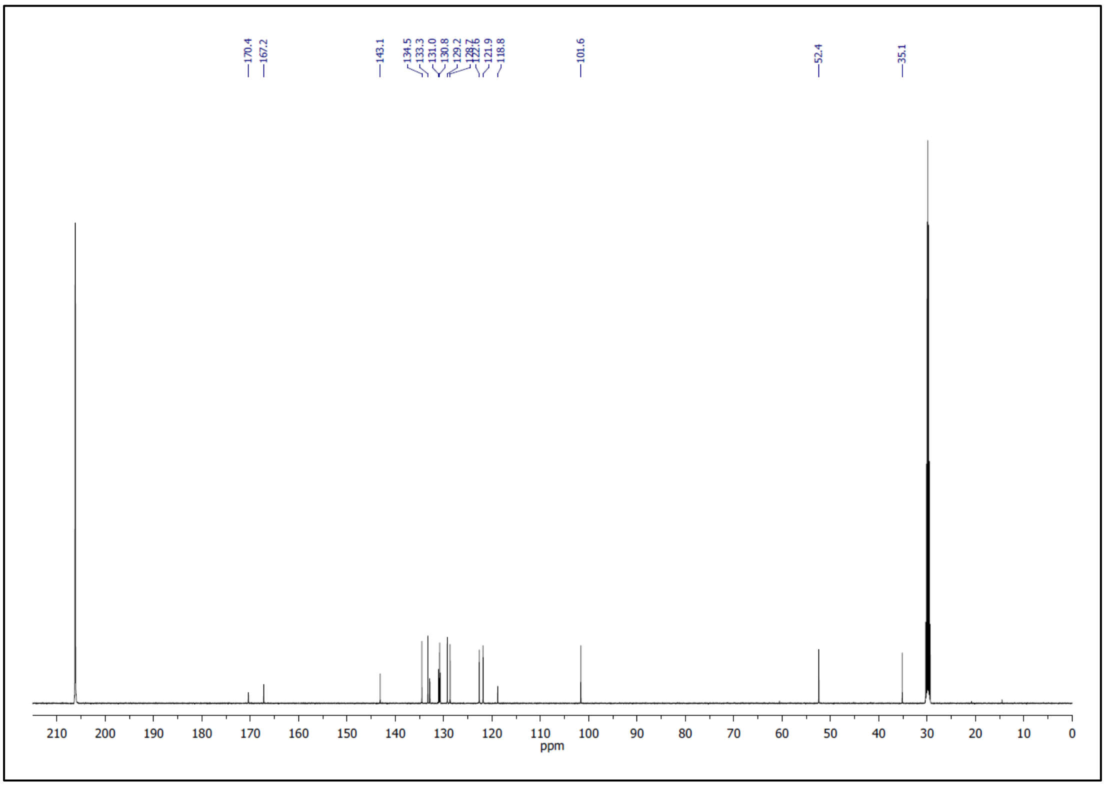

1-methyl-6-(3-(trifluoromethyl)phenyl)-1H-indole-7-carboxylic acid (**5ap**)

^{1}H NMR $((CD_3)_2CO)$

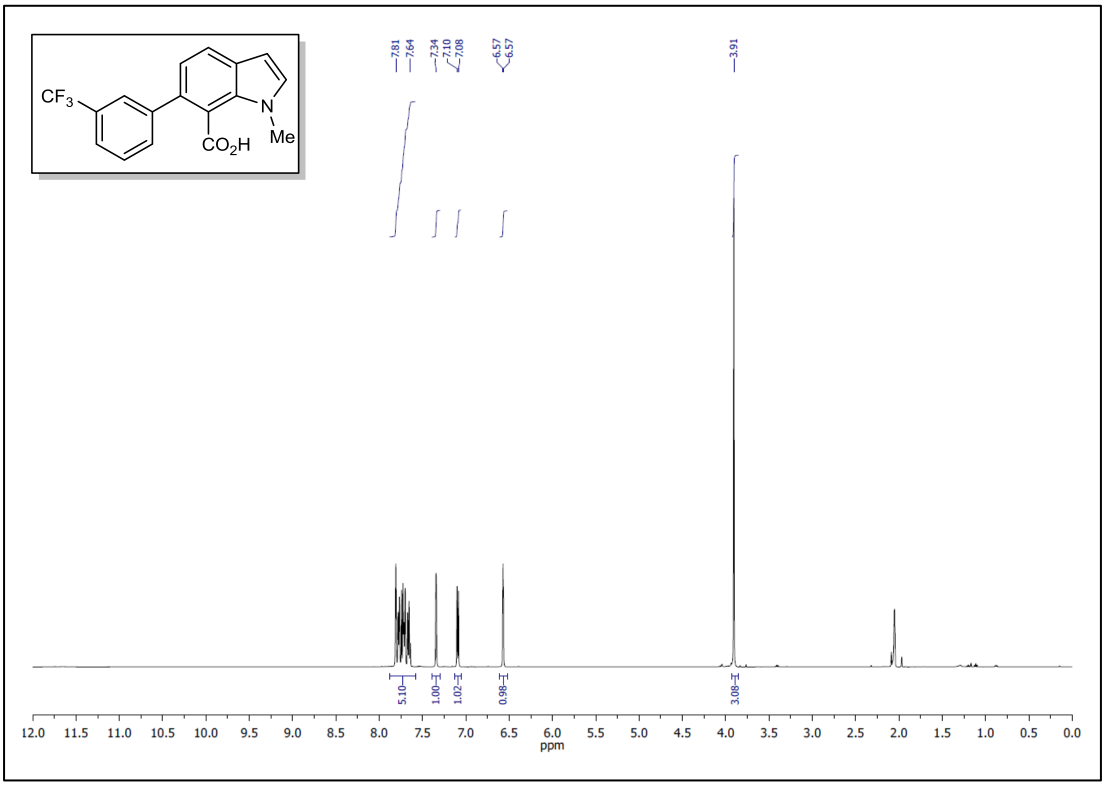

^{13}C NMR $((CD_3)_2CO)$

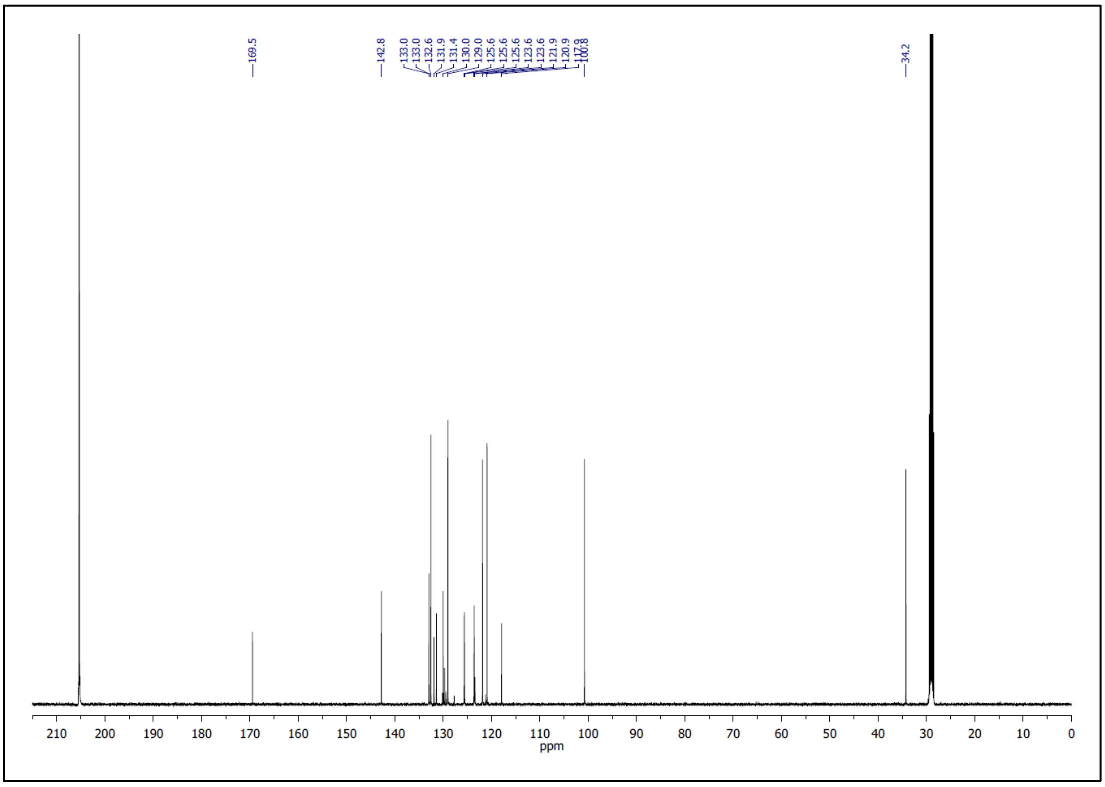

^{19}F NMR ($(CD_3)_2CO$)

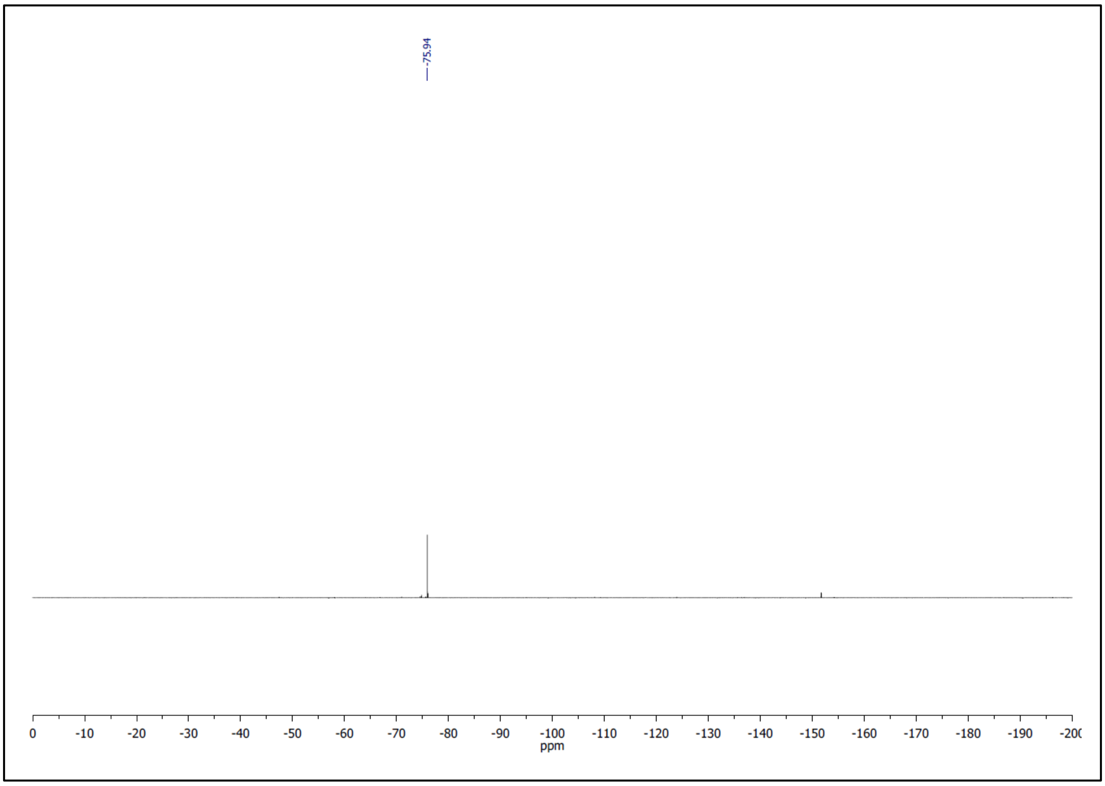

1-(4-methoxyphenyl)-1H-indole-7-carboxylic acid (**5ba**)

^{1}H NMR ($(CD_3)_2CO$)

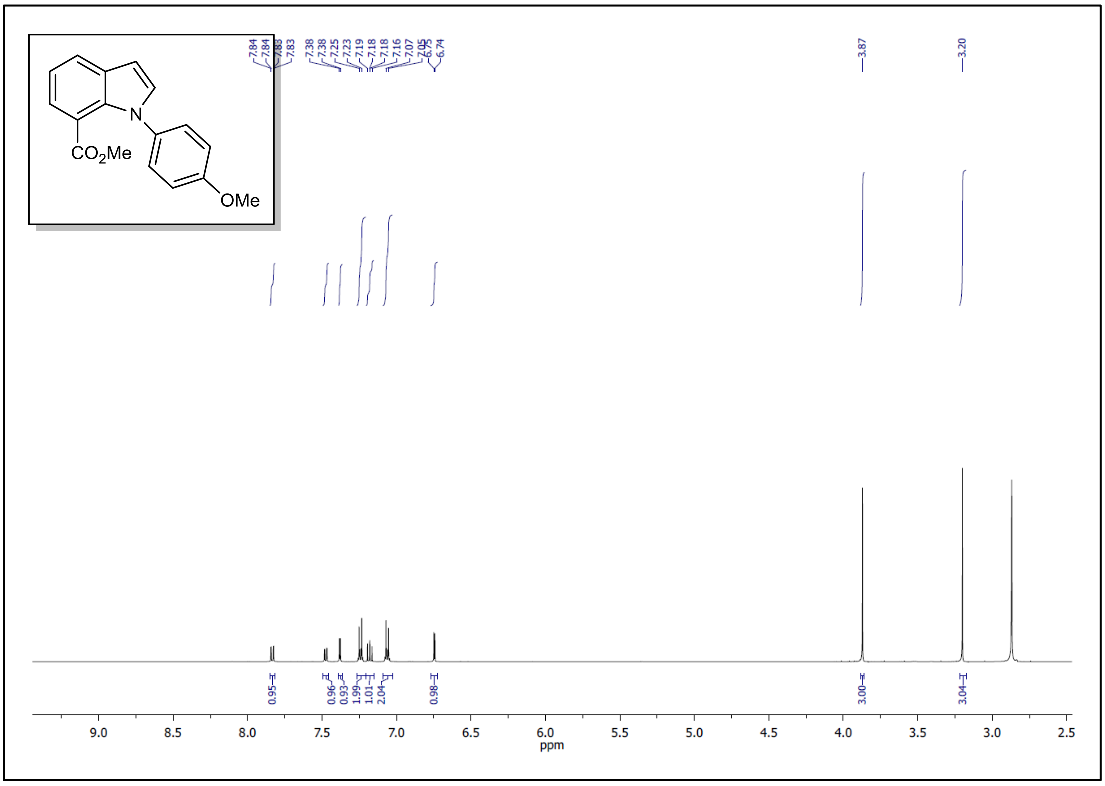

^{13}C NMR ($(CD_3)_2CO$)

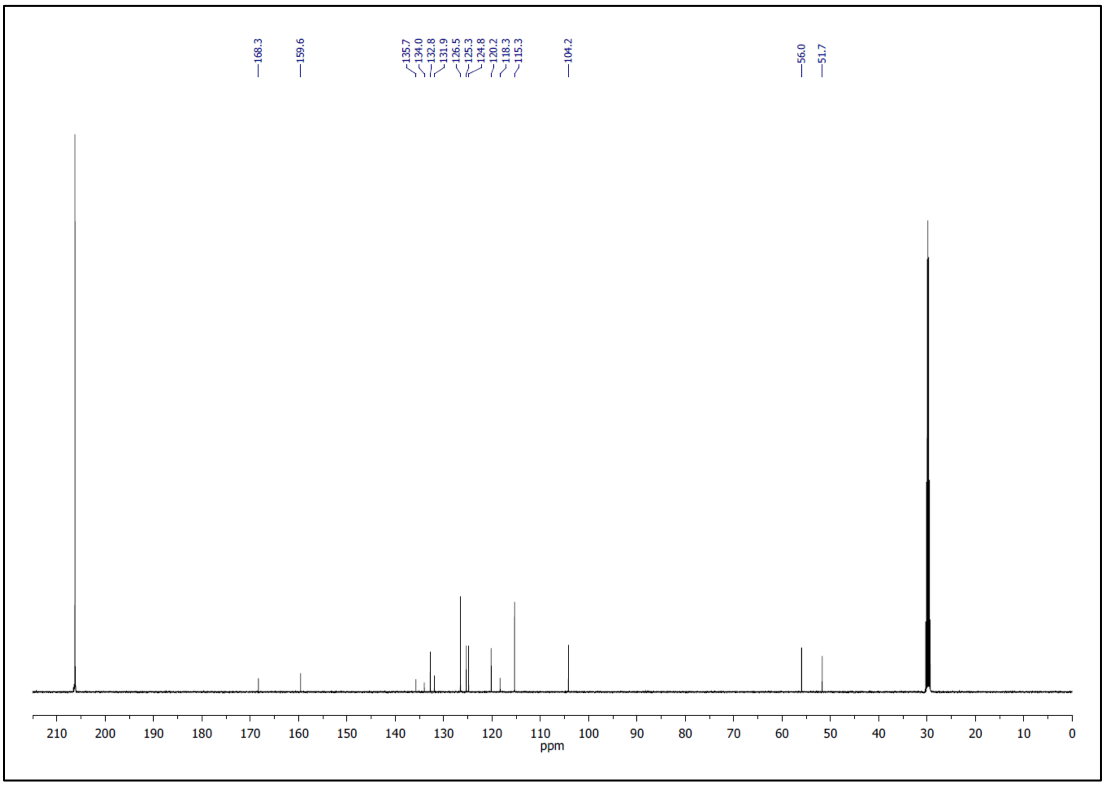

5-(4-methoxyphenyl)-1-methyl-1H-indole-6-carboxylic acid (**5ca**)

^{1}H NMR ($(CD_3)_2CO$)

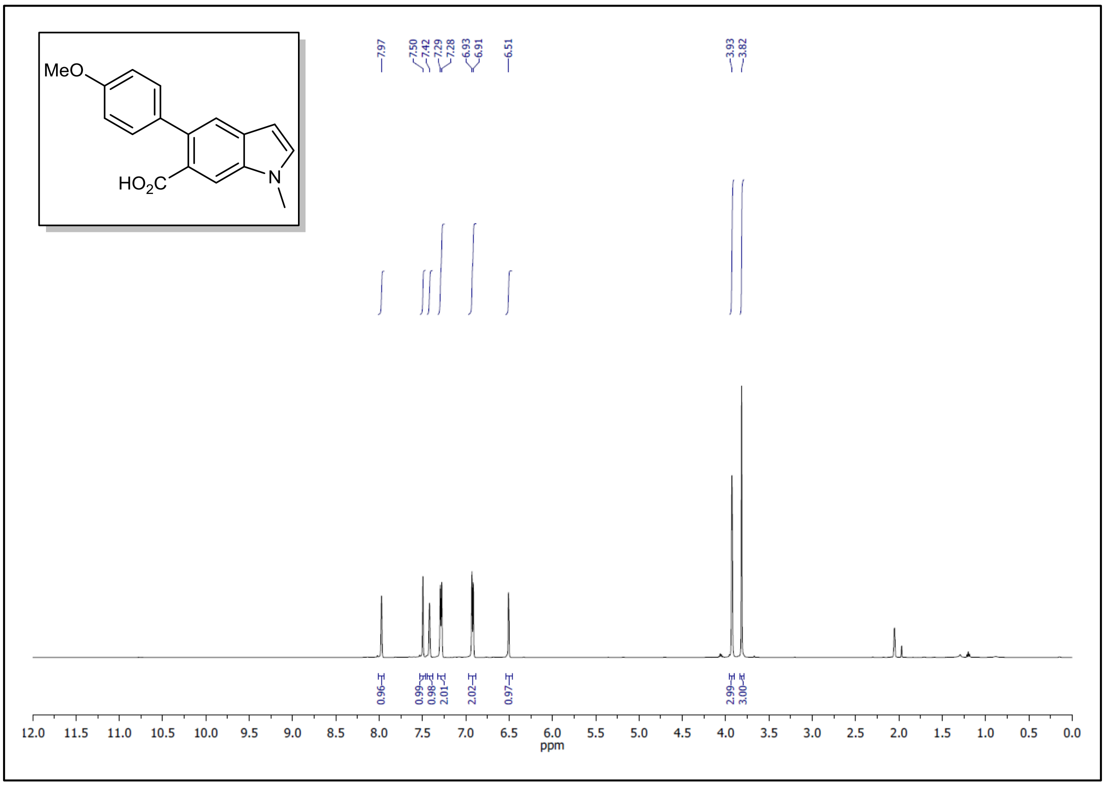

^{13}C NMR ($(CD_3)_2CO$)

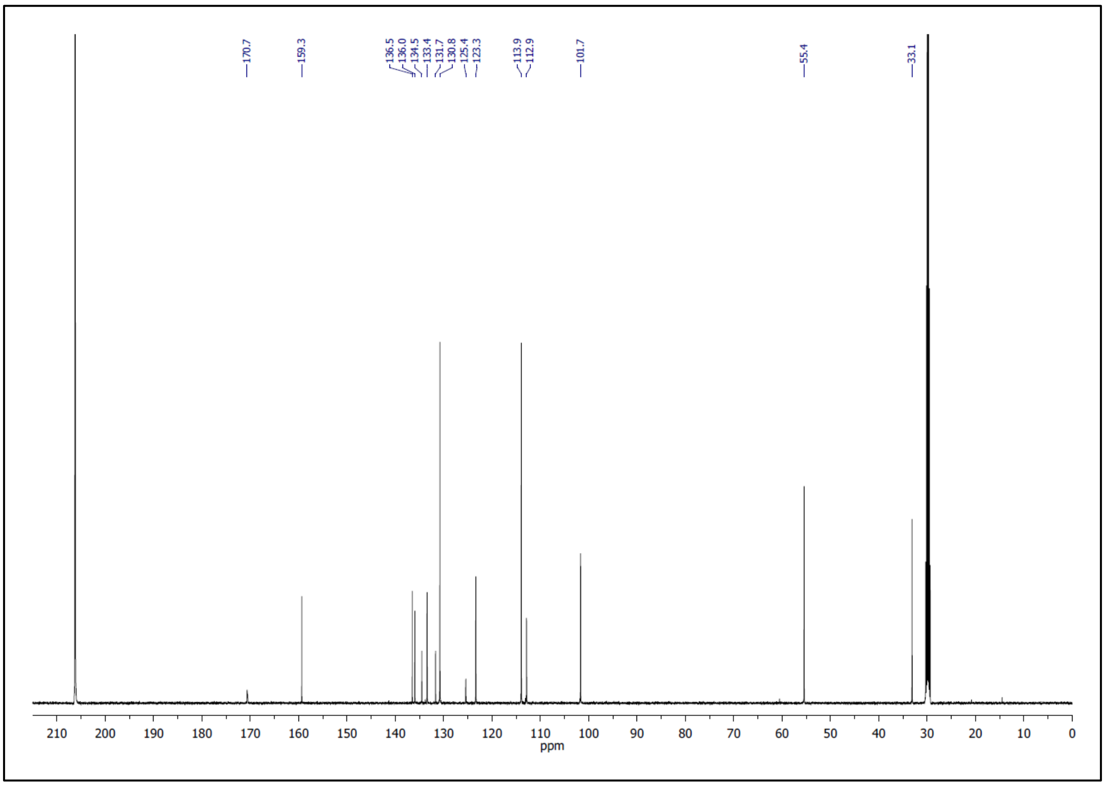

1-methyl-5-(4-(trifluoromethyl)phenyl)-1H-indole-6-carboxylic acid (**5co**)

^{1}H NMR ($(CD_3)_2CO$)

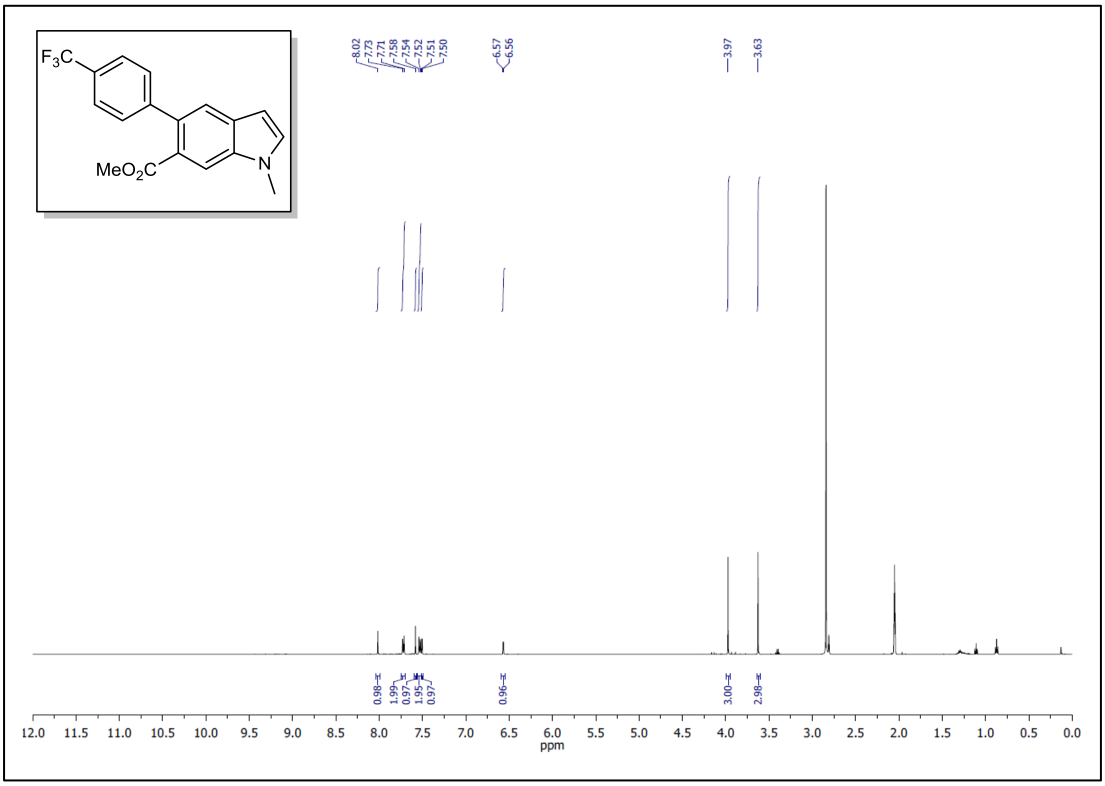

^{13}C NMR ($(CD_3)_2CO$)

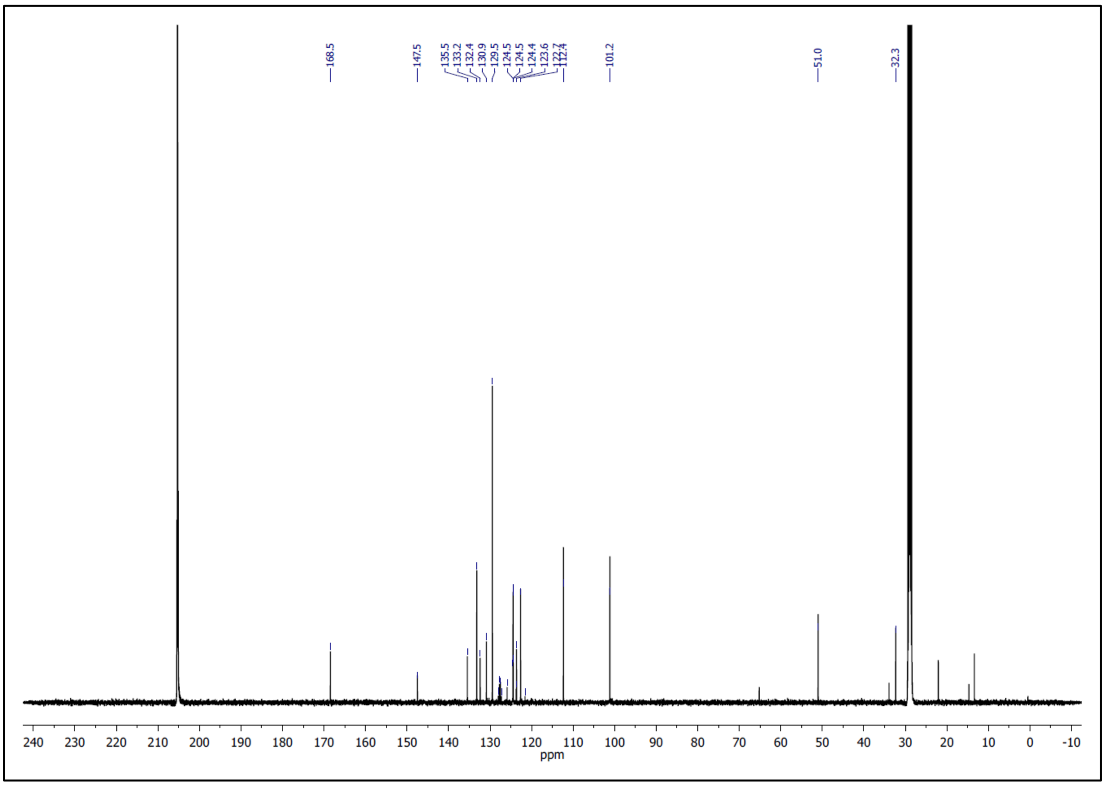

^{19}F NMR ($(CD_3)_2CO$)

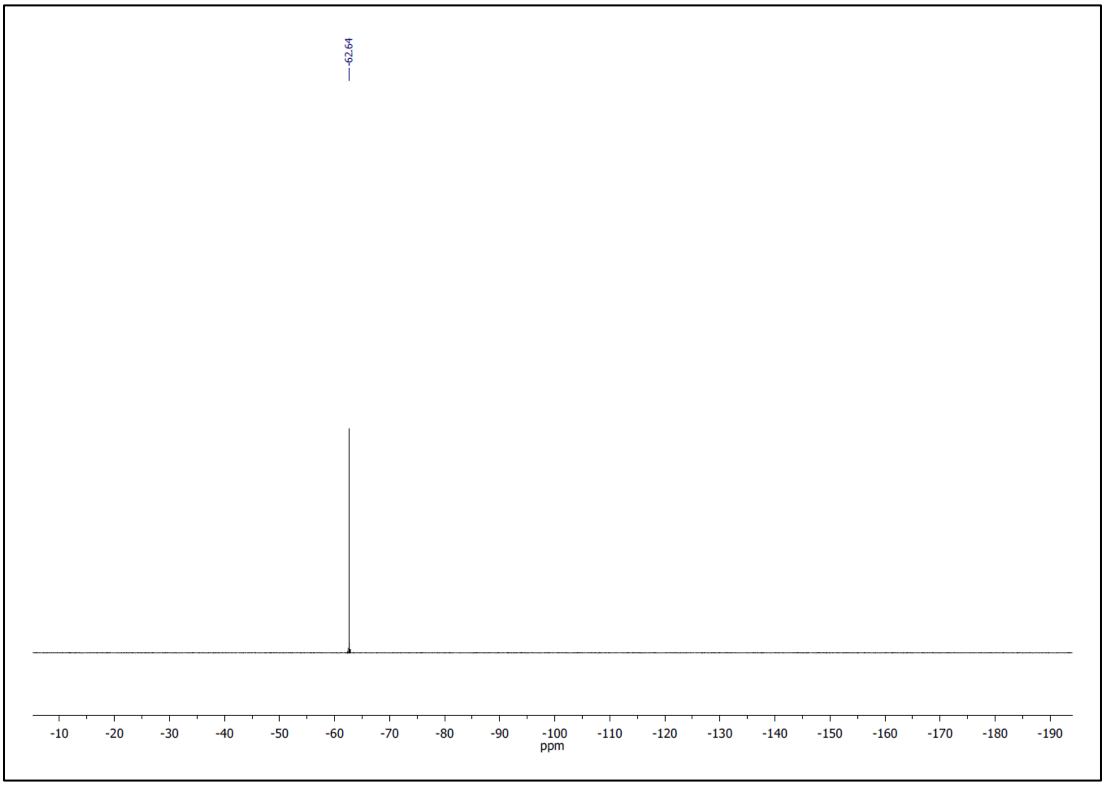

5-(4-fluorophenyl)-1-methyl-1H-indole-6-carboxylic acid (**5ci**)

^{1}H NMR ($(CD_3)_2CO$)

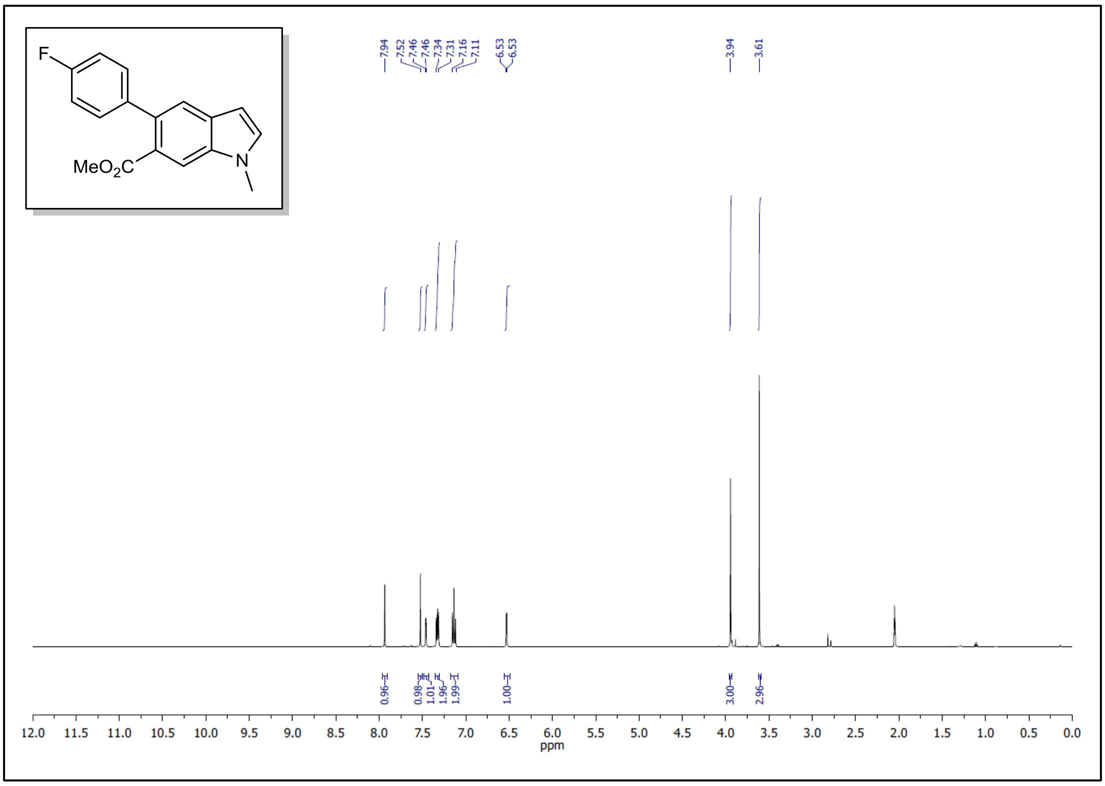

^{13}C NMR ($(CD_3)_2CO$)

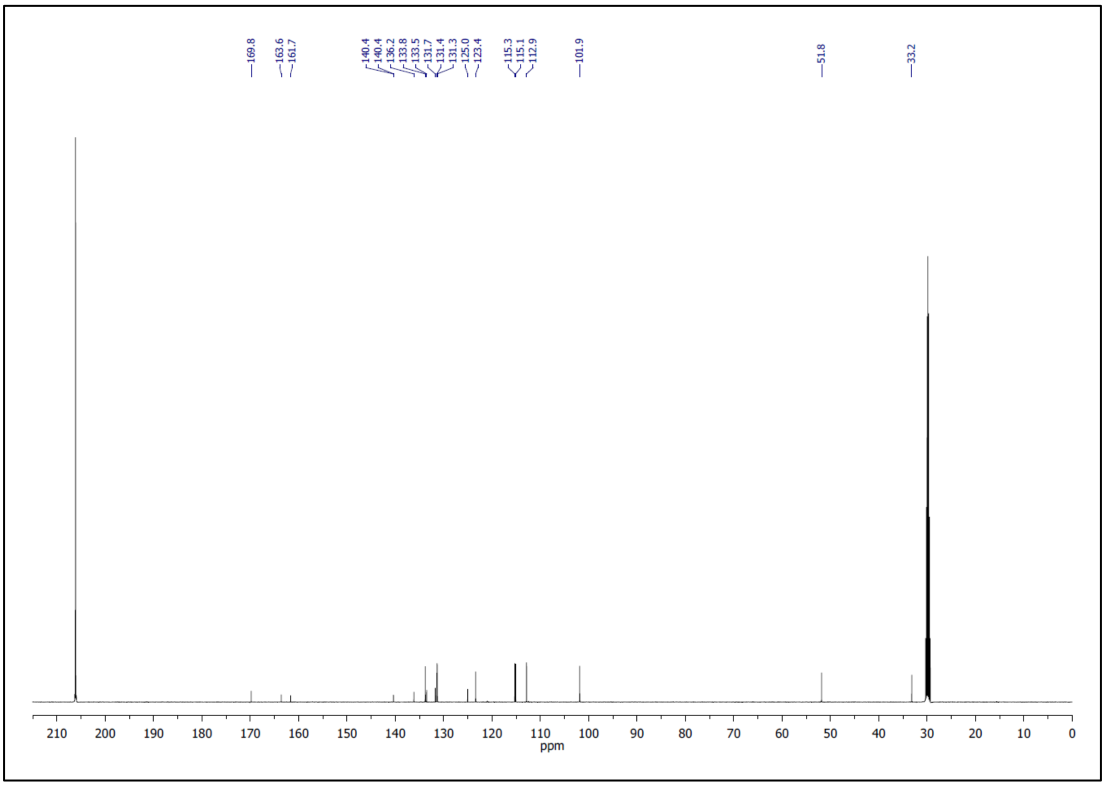

^{19}F NMR ($(CD_3)_2CO$)

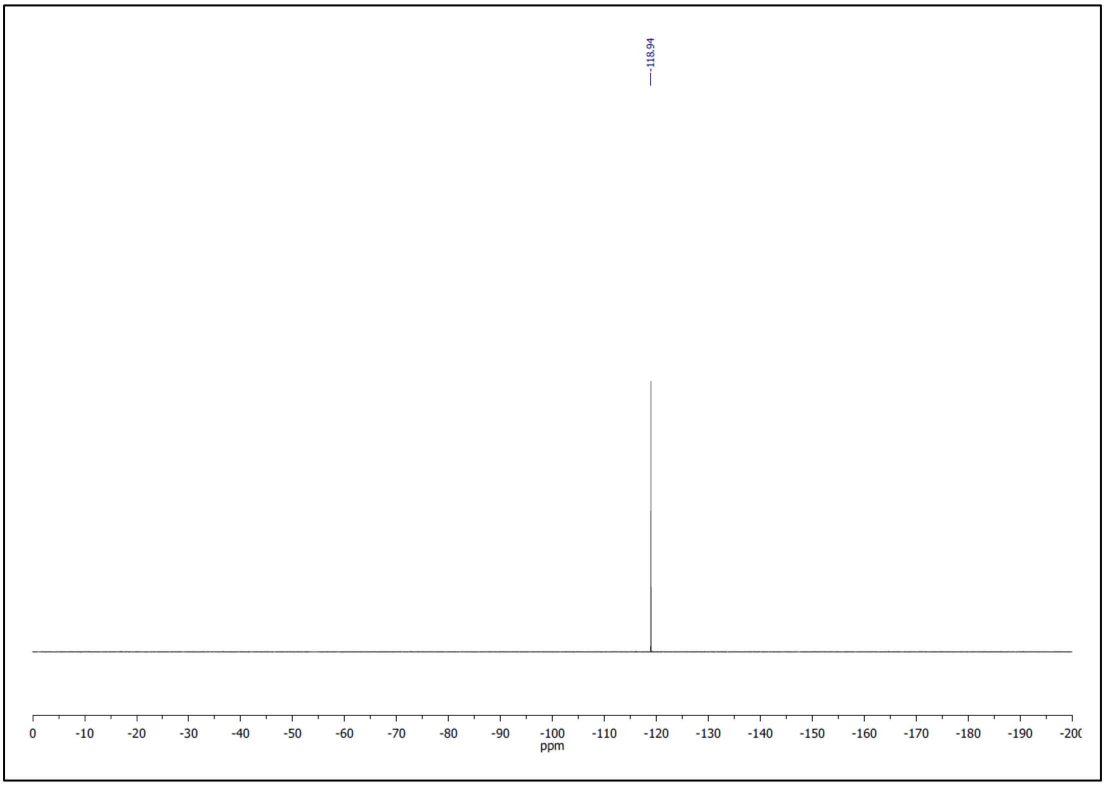

1-methyl-5-phenyl-1H-indole-6-carboxylic acid (**5ce**)

^{1}H NMR ($(CD_3)_2CO$)

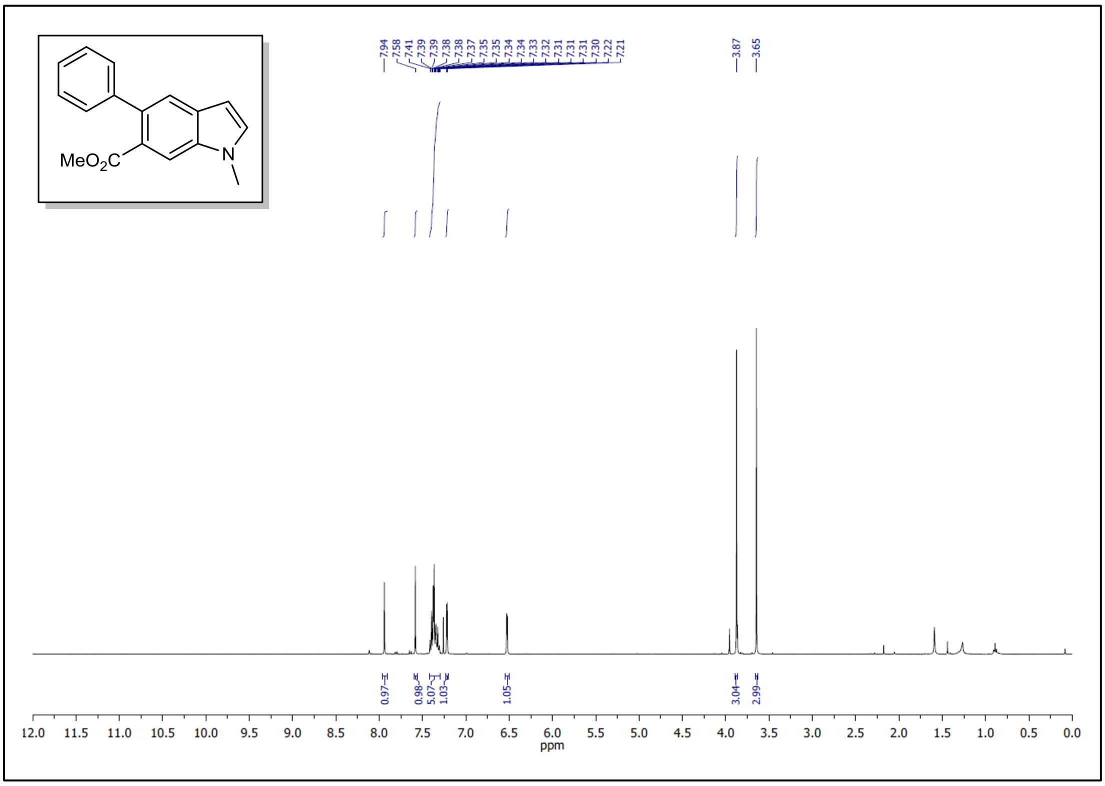

^{13}C NMR ($(CD_3)_2CO$)

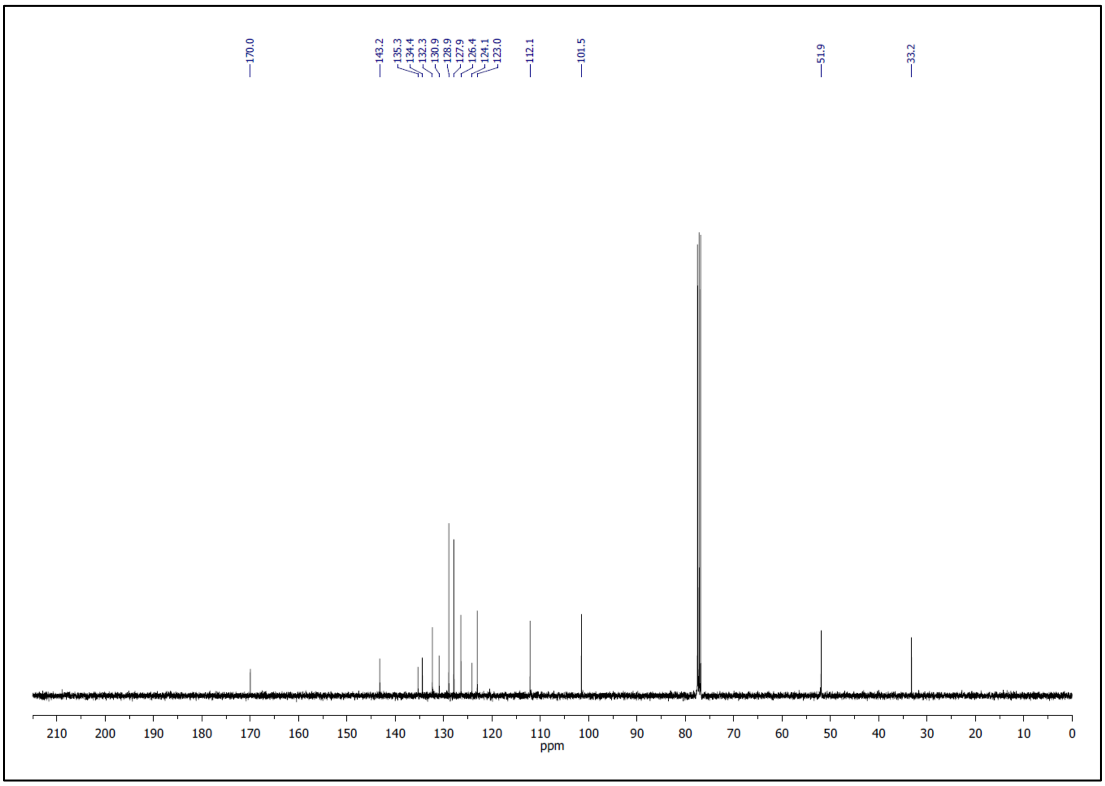

Benzyl 3-(2-(dimethylamino)-2-oxoacetyl)-5-(4-methoxyphenyl)-1-methyl-1H-indole-6-carboxylate (**5da**)

^{1}H NMR ($(CD_3)_2CO$)

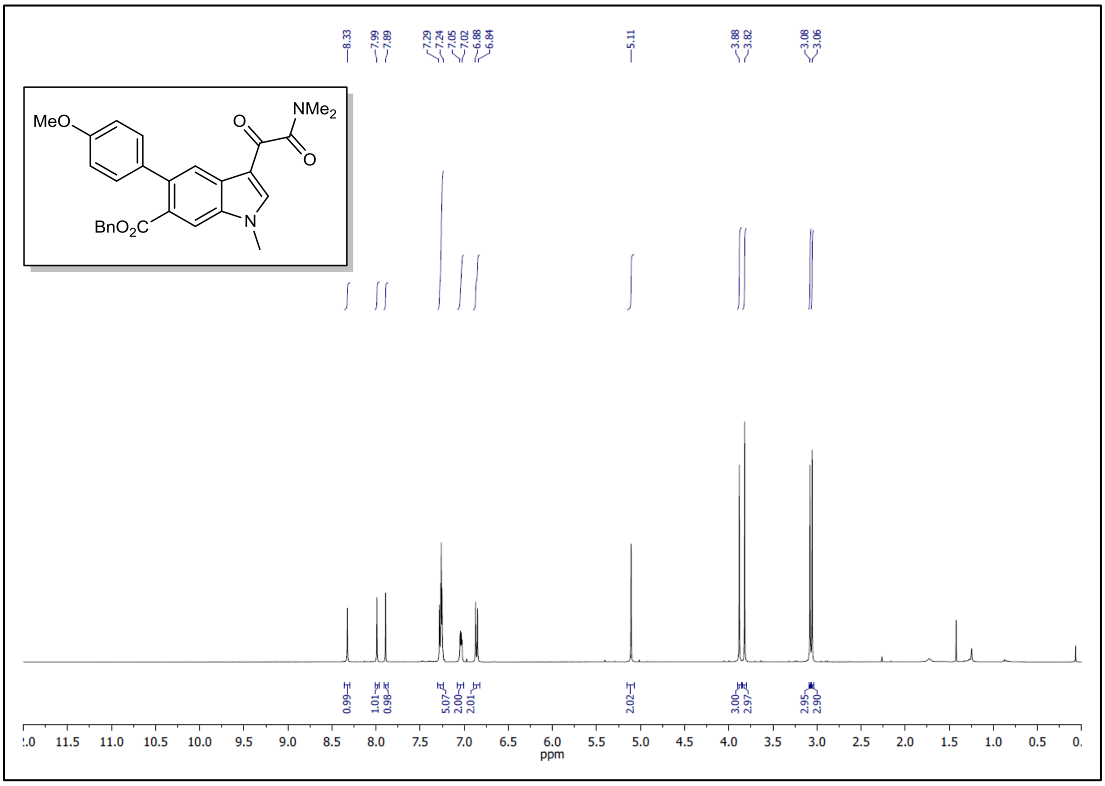

^{13}C NMR ($(CD_3)_2CO$)

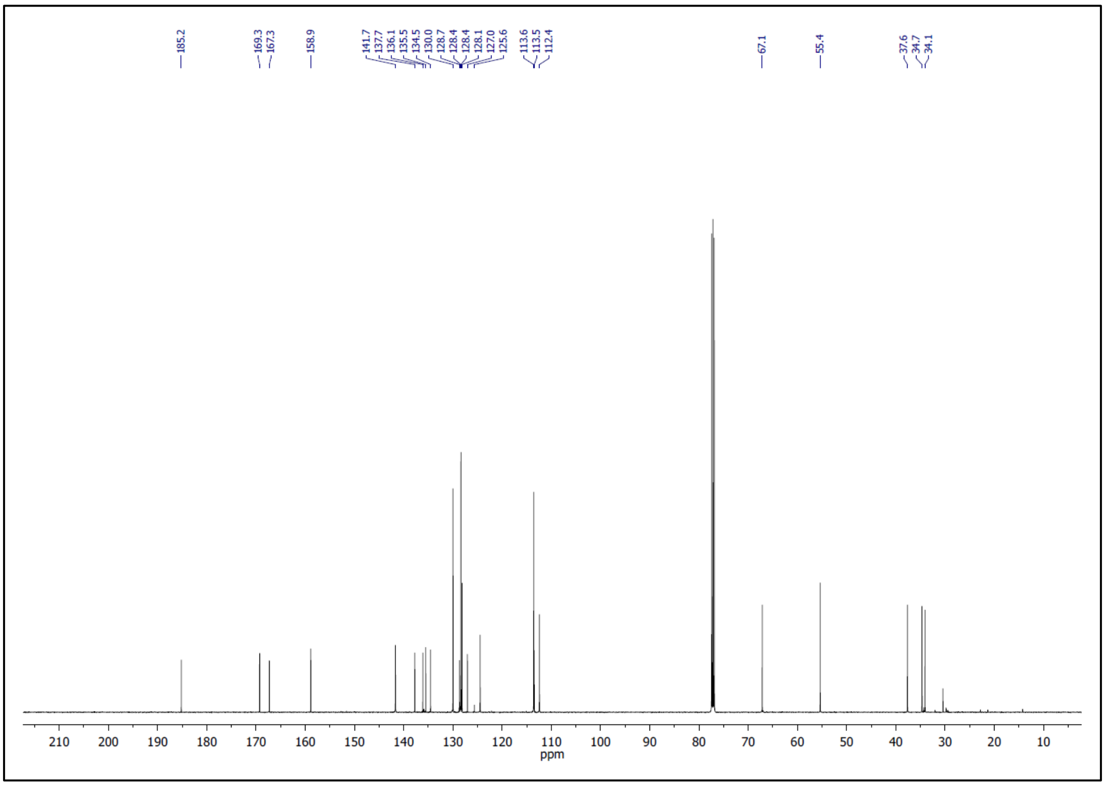

5,7-bis(4-methoxyphenyl)-1H-indole-6-carboxylic acid (**5ea**)

^{1}H NMR ($(CD_3)_2CO$)

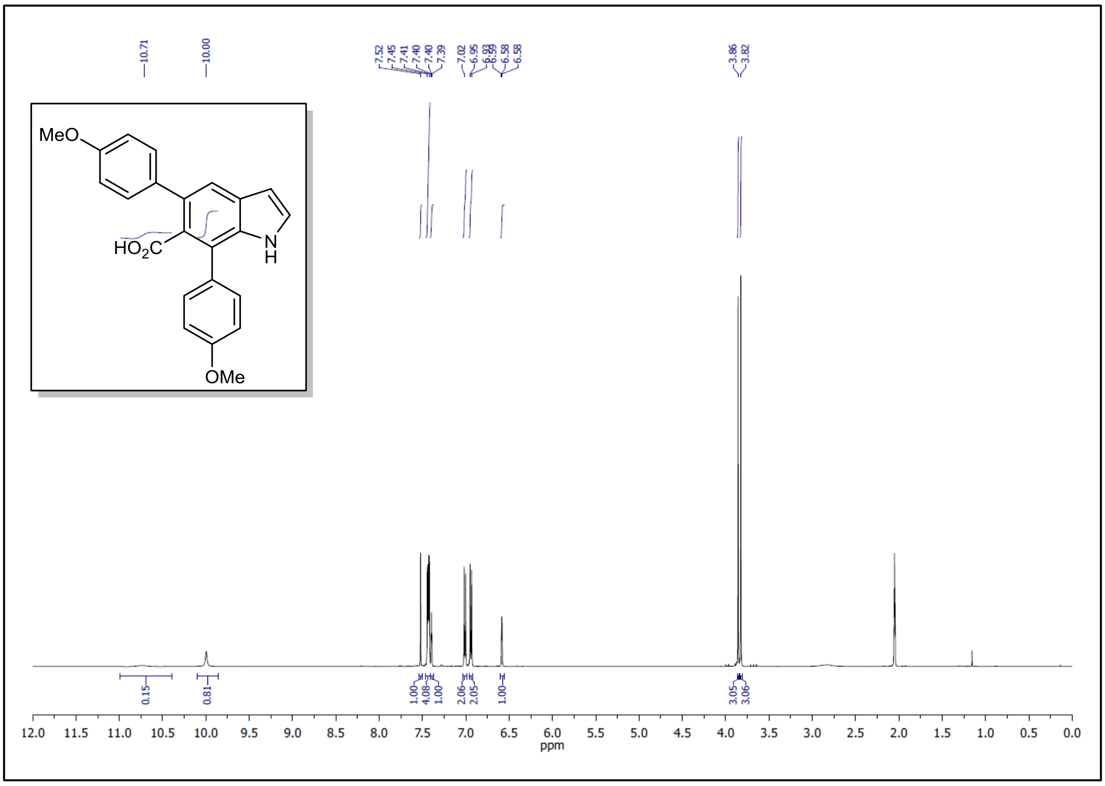

^{13}C NMR ($(CD_3)_2CO$)

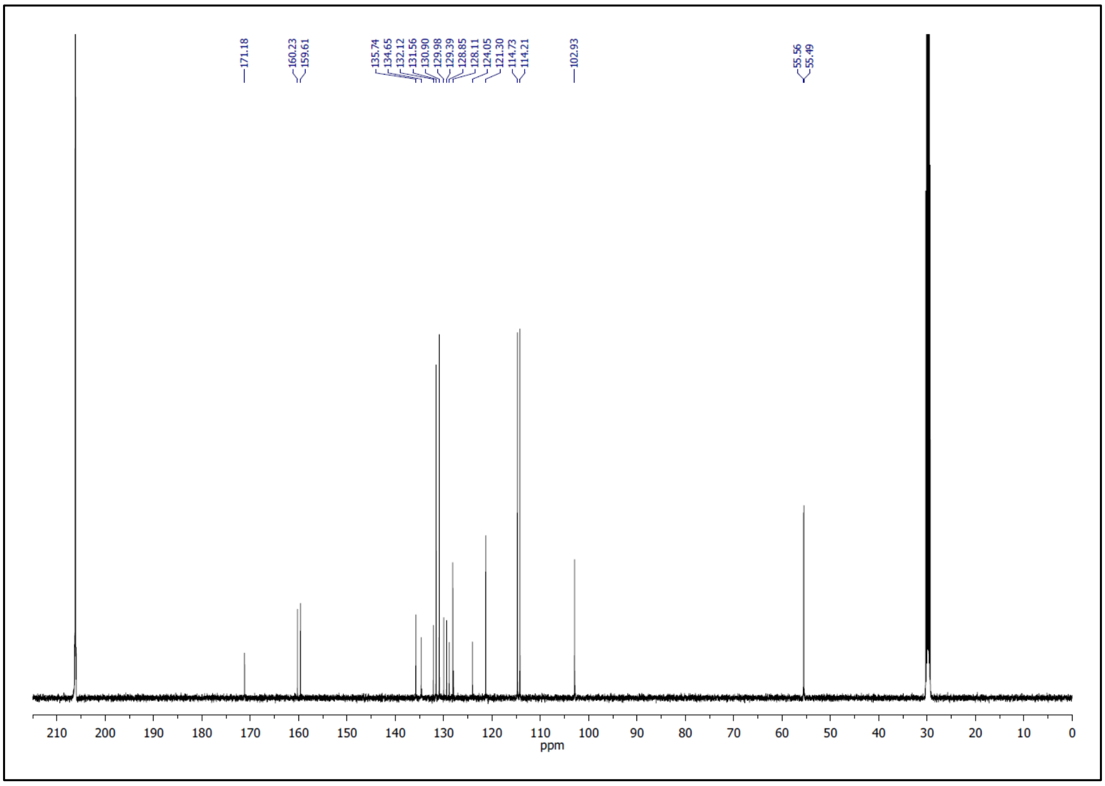

Methyl 3-cyclohexyl-5,7-bis(4-methoxyphenyl)-1H-indole-6-carboxylate (**5fa**)

^{1}H NMR ($(CD_3)_2CO$)

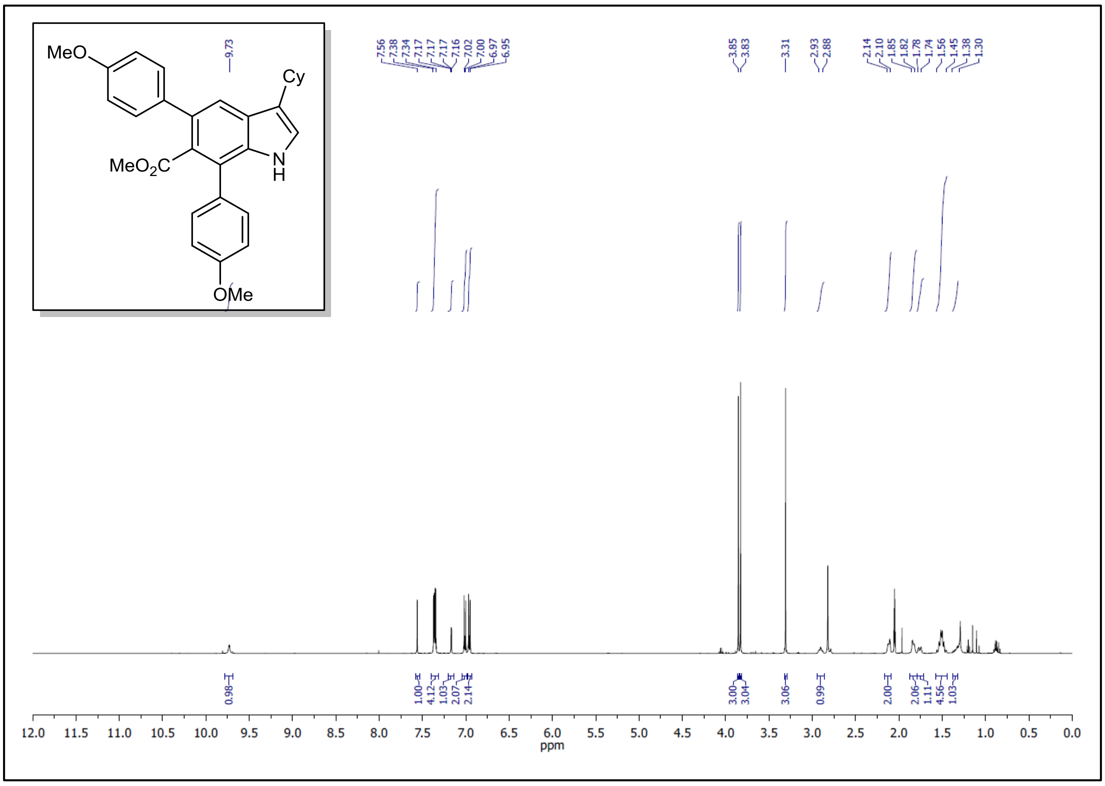

^{13}C NMR ($(CD_3)_2CO$)

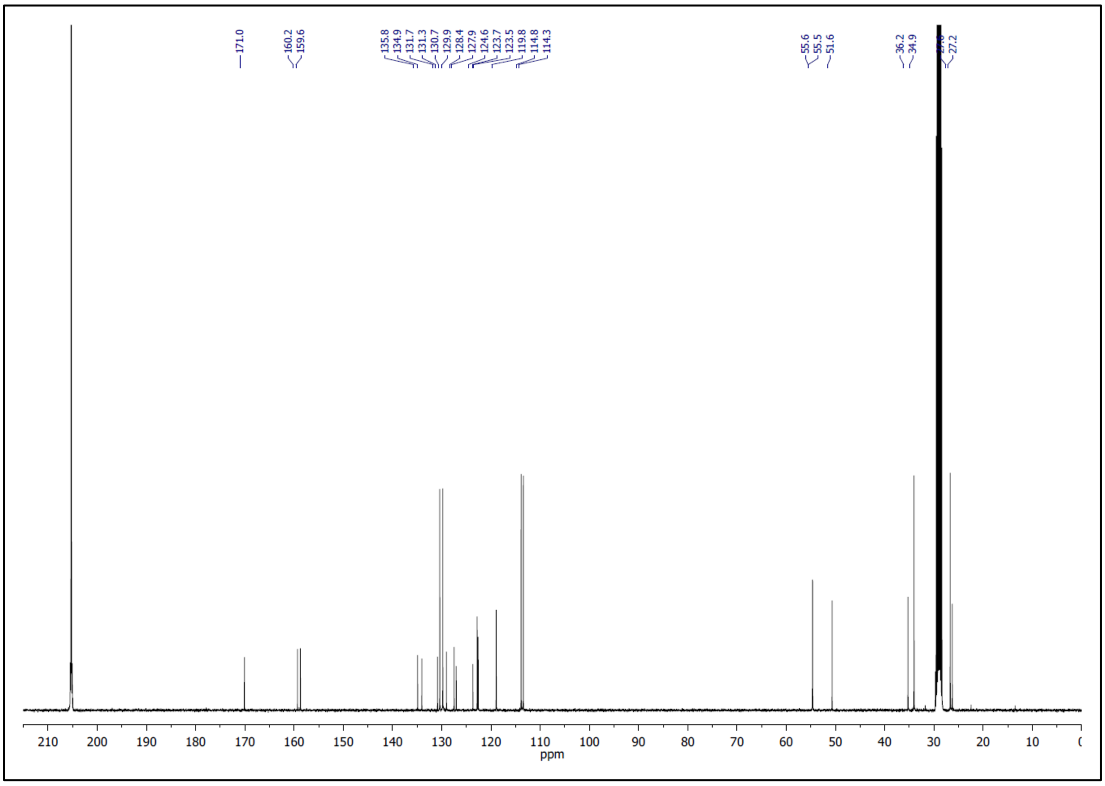

5-(4-methoxyphenyl)-1-methyl-1H-indole-4-carboxylic acid (**5ga**)

^{1}H NMR ($(CD_3)_2CO$)

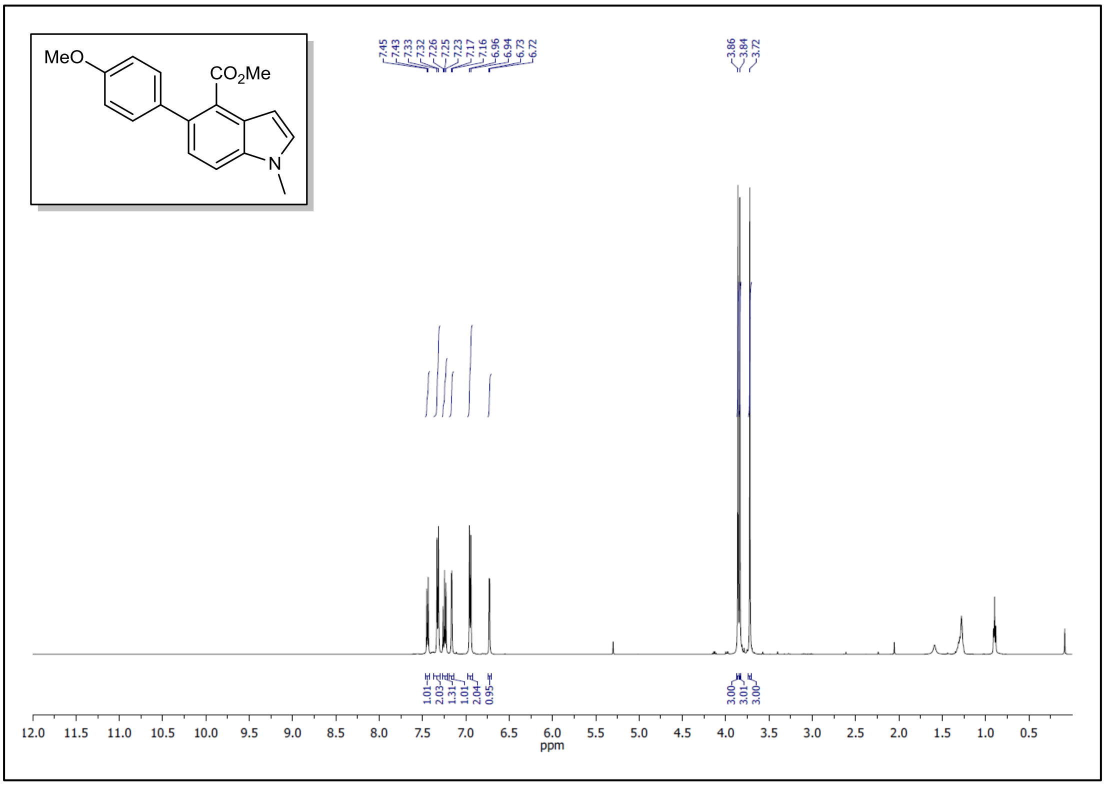

^{13}C NMR ($(CD_3)_2CO$)

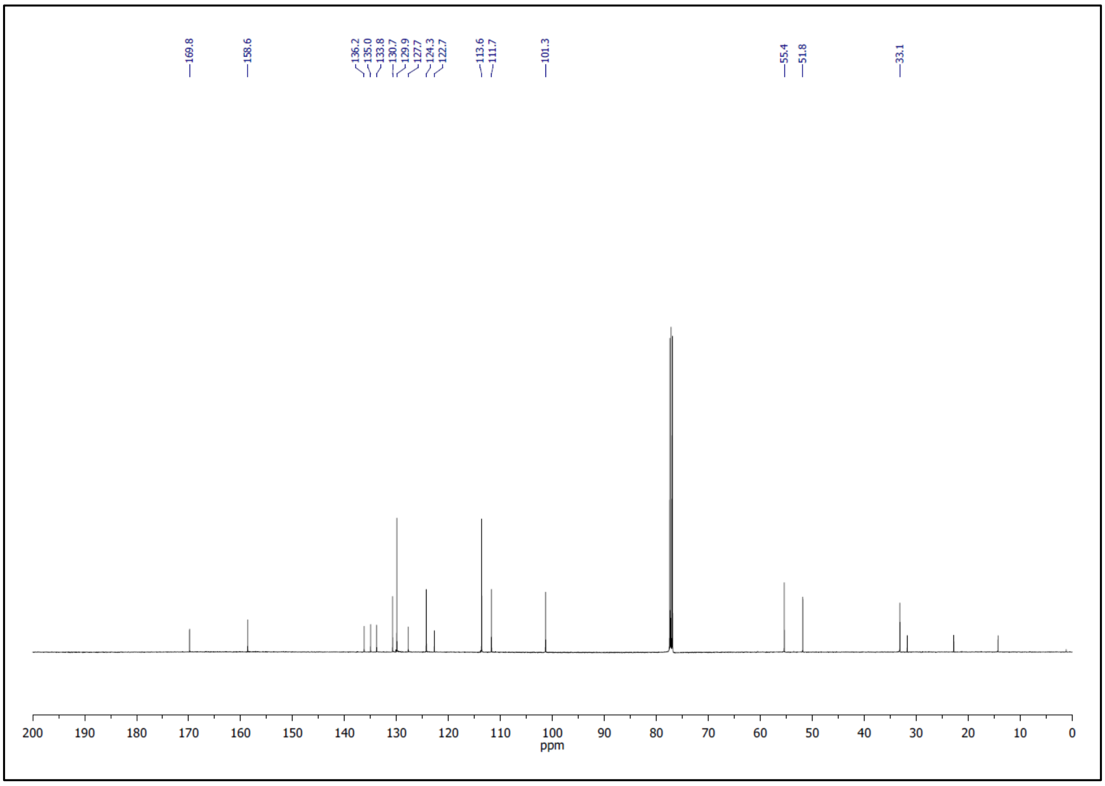

1-methyl-5-phenyl-1H-indole-4-carboxylic acid (**5ge**)

^{1}H NMR ($(CD_3)_2CO$)

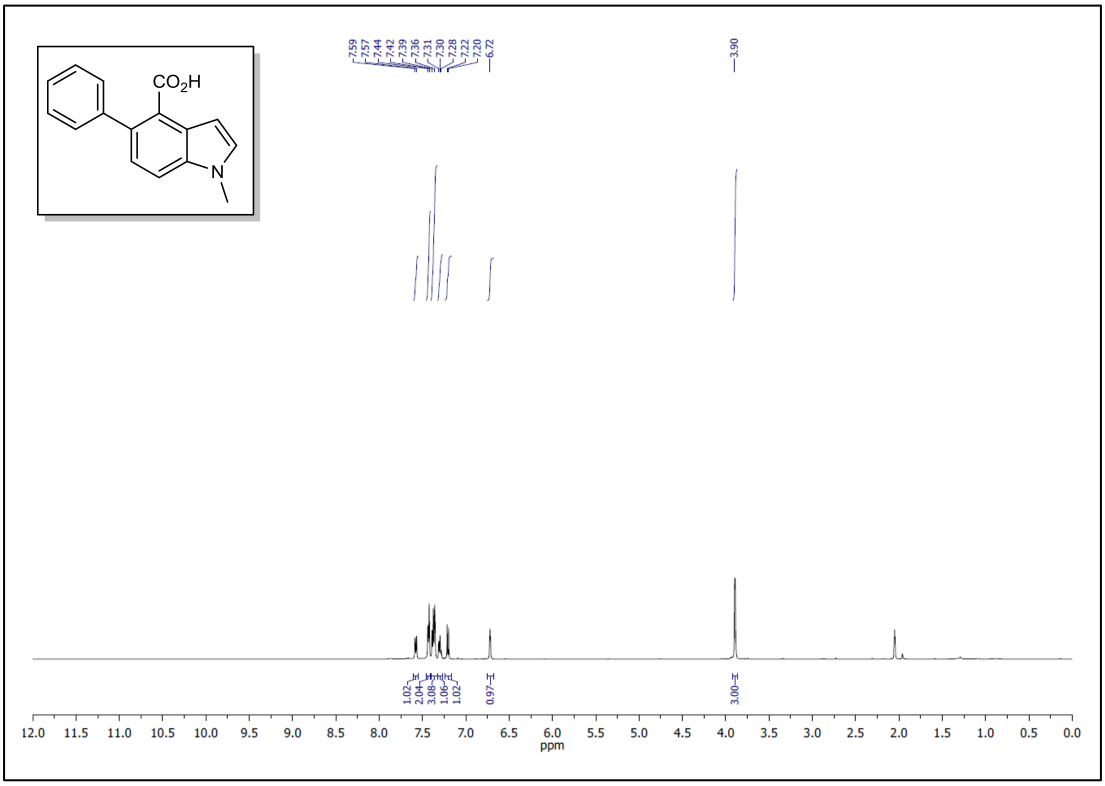

^{13}C NMR ($(CD_3)_2CO$)

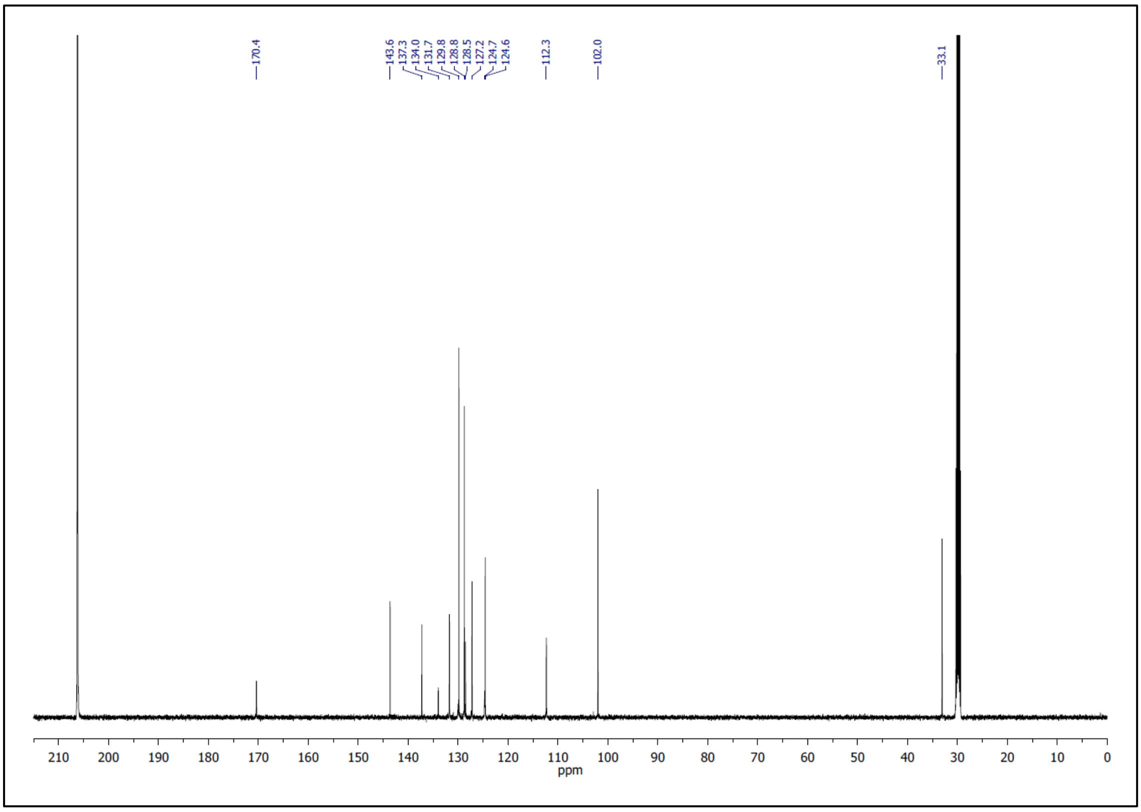

1-methyl-5-(4-(trifluoromethyl)phenyl)-1H-indole-4-carboxylic acid (**5go**)

^{1}H NMR ($(CD_3)_2CO$)

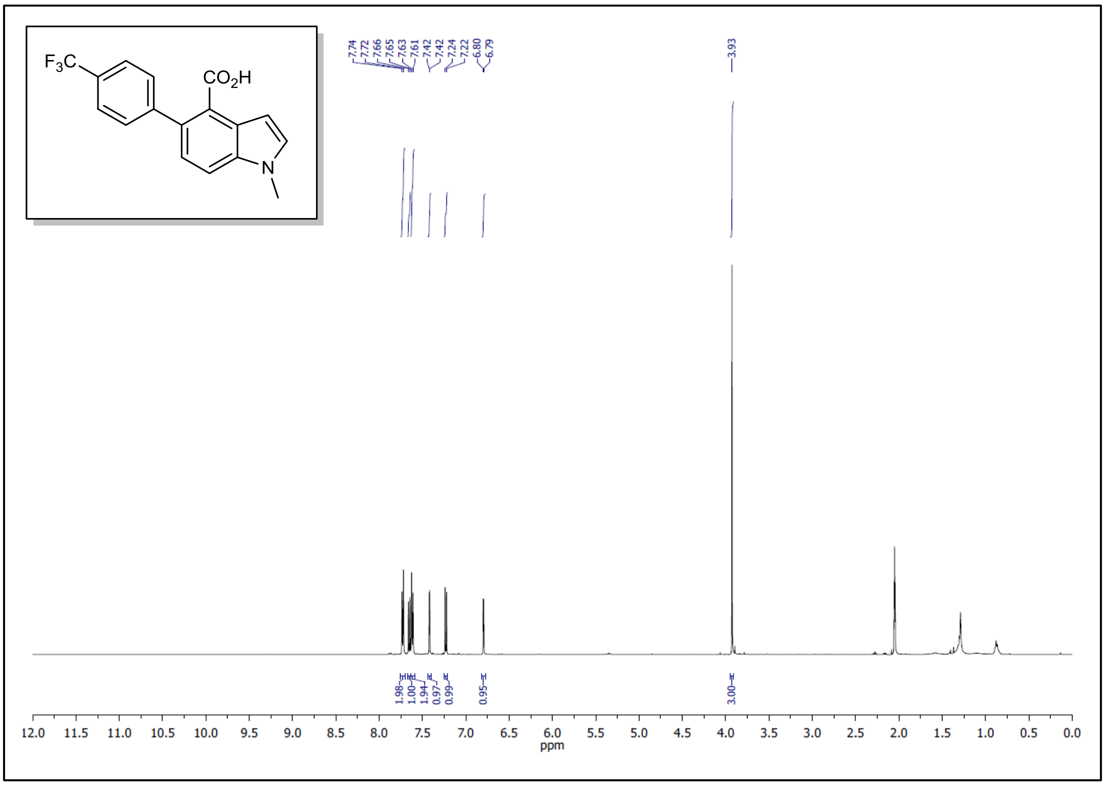

^{13}C NMR ($(CD_3)_2CO$)

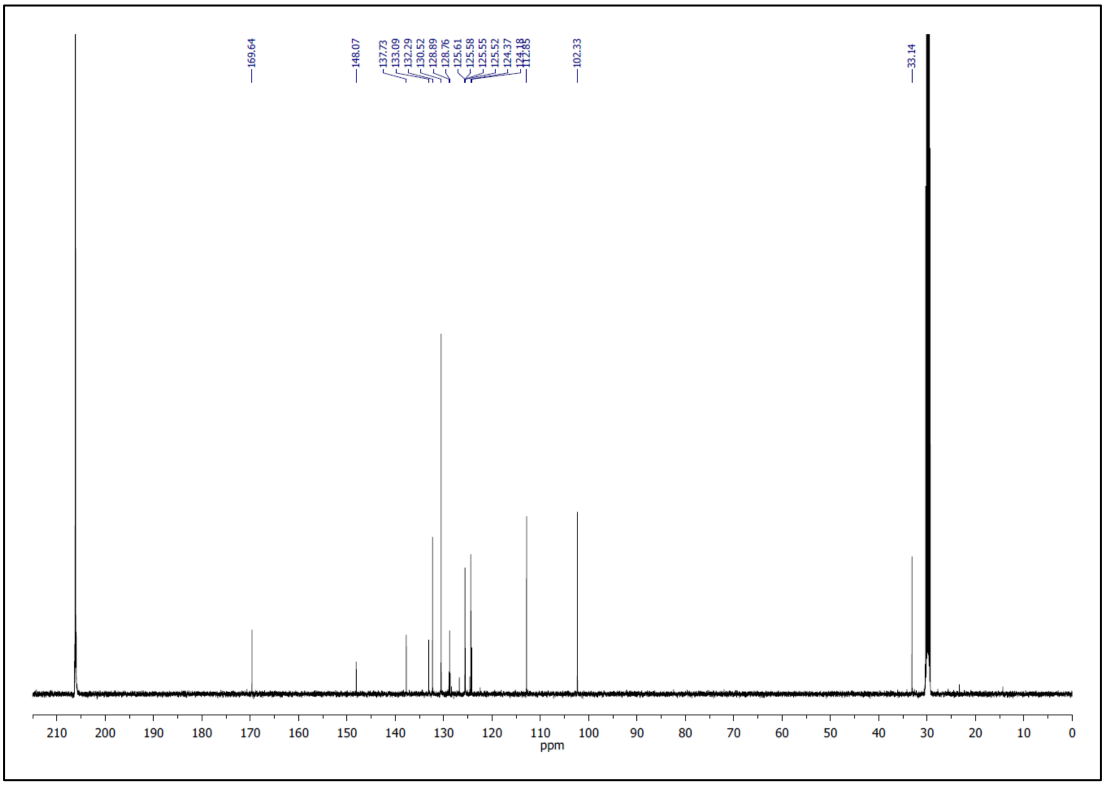

^{19}F NMR ($(CD_3)_2CO$)

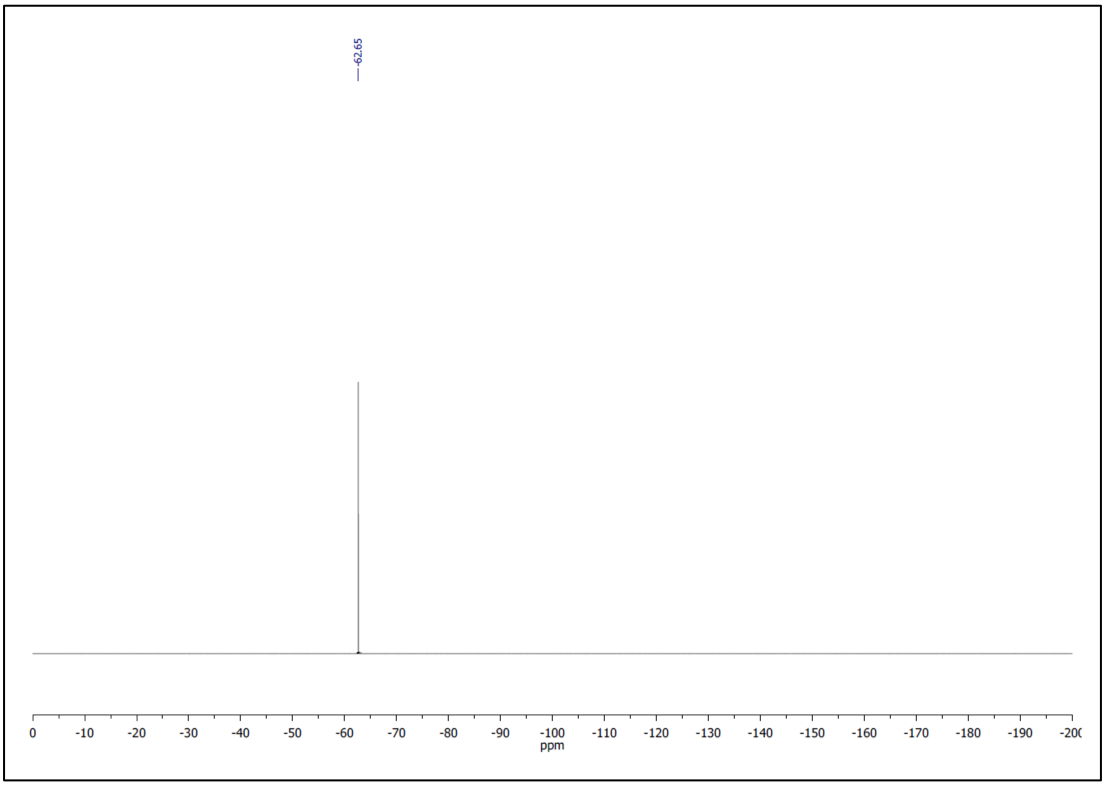

5-(4-fluorophenyl)-1-methyl-1H-indole-4-carboxylic acid (**5gi**)

^{1}H NMR ($(CD_3)_2CO$)

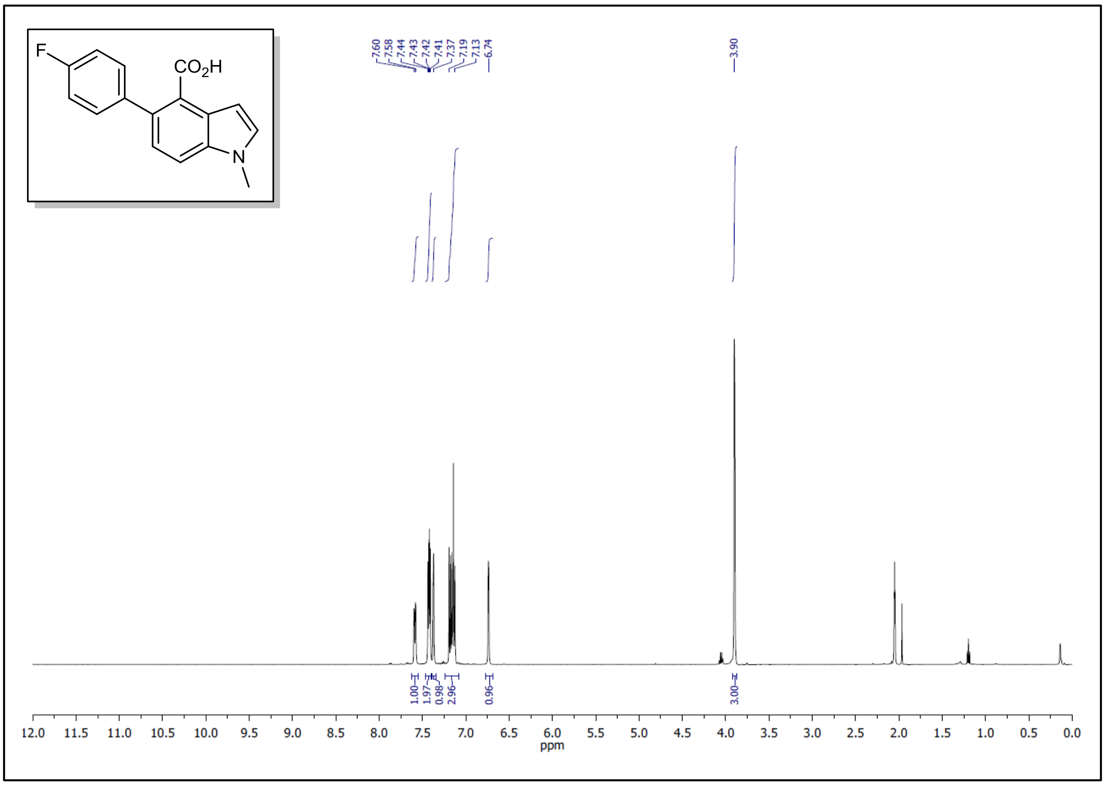

^{13}C NMR ($(CD_3)_2CO$)

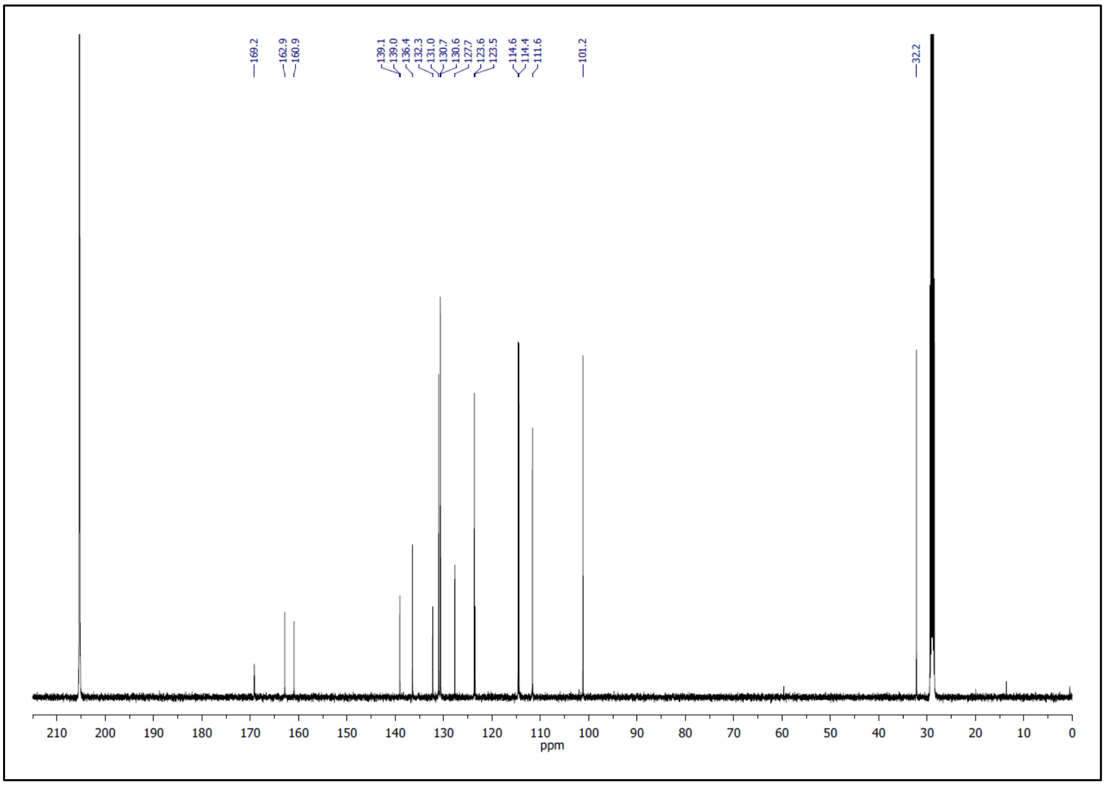

^{19}F NMR ($(CD_3)_2CO$)

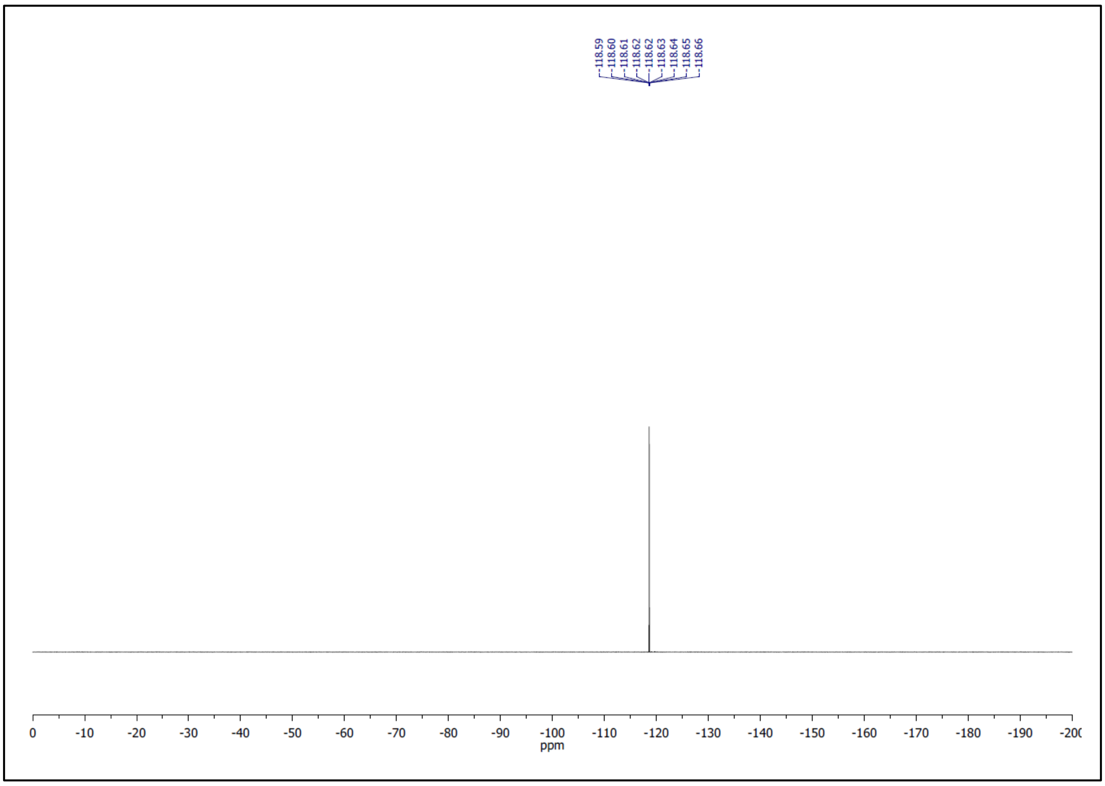

5-(4-bromophenyl)-1-methyl-1H-indole-4-carboxylic acid (**5gk**)

^{1}H NMR ($(CD_3)_2CO$)

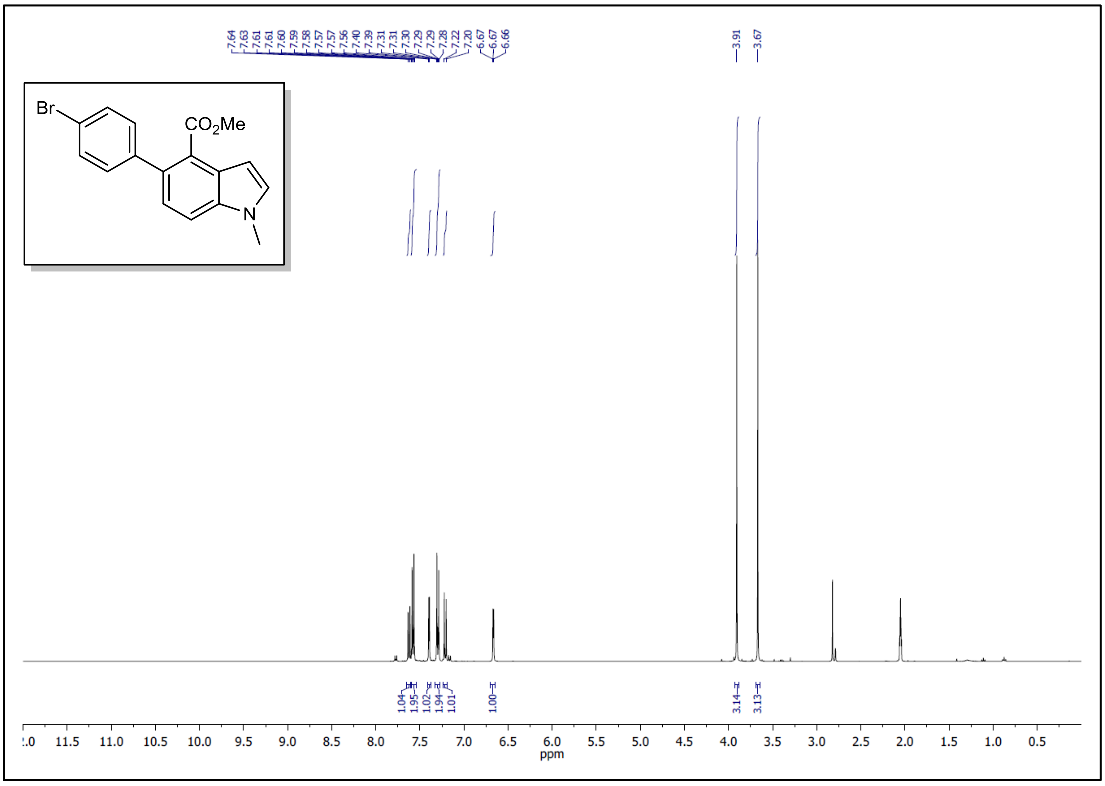

^{13}C NMR ($(CD_3)_2CO$)

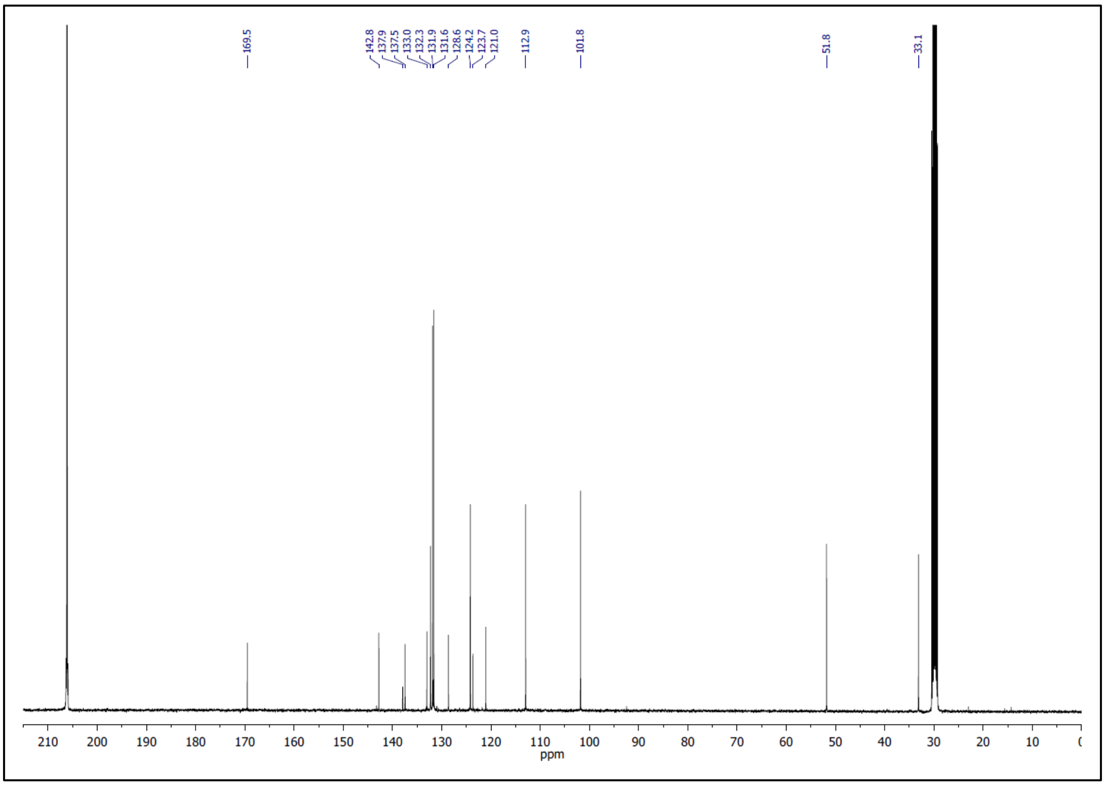

4,6-bis(4-methoxyphenyl)-1-methyl-1H-indole-5-carboxylic acid (**5ia**)

^{1}H NMR ($(CD_3)_2CO$)

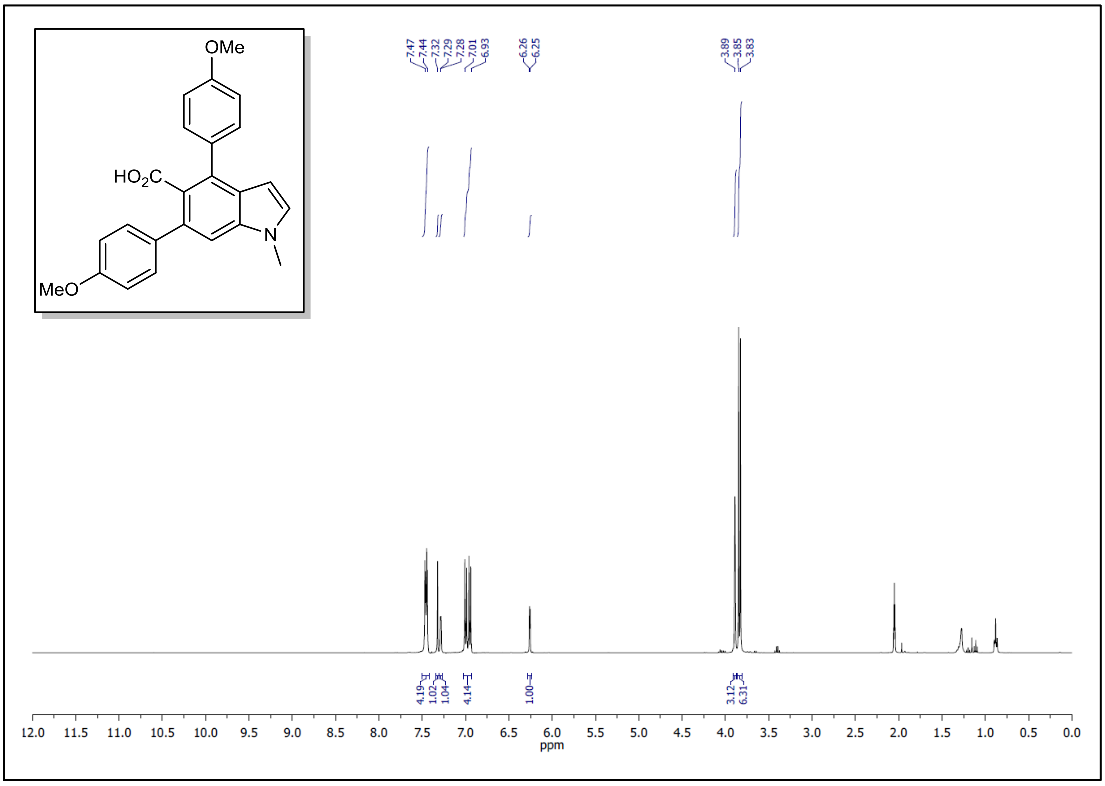

^{13}C NMR ($(CD_3)_2CO$)

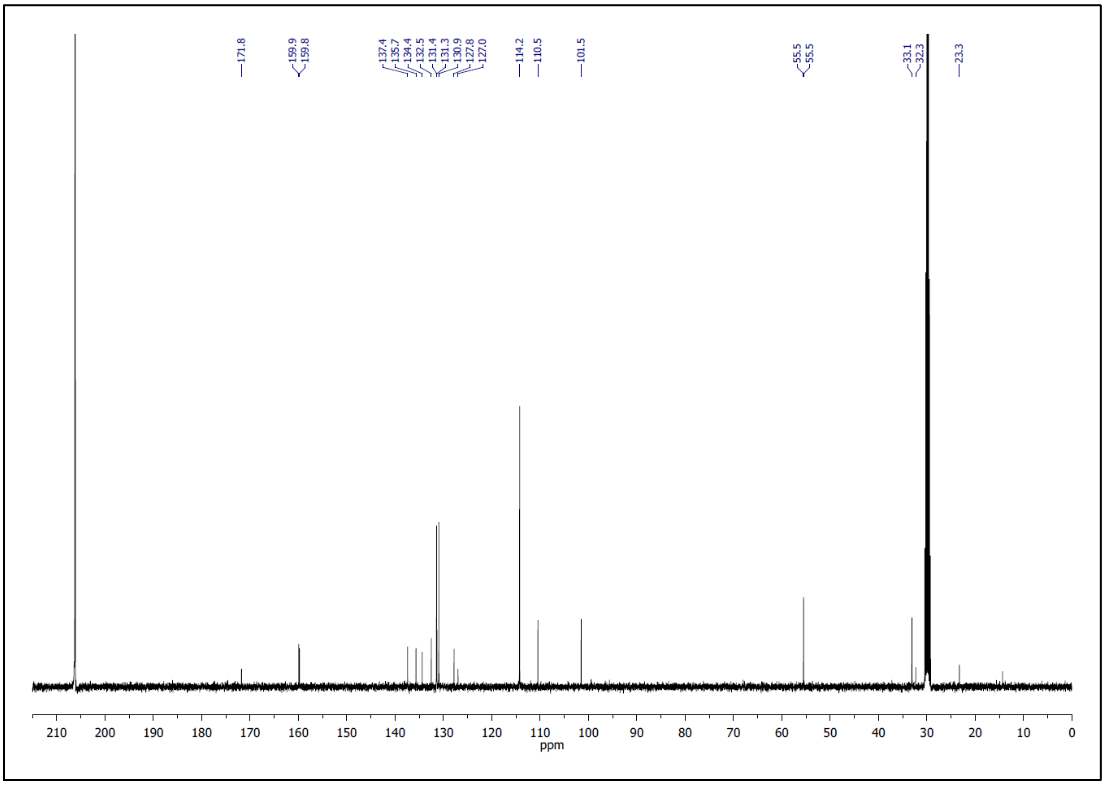

1-methyl-4,6-diphenyl-1H-indole-5-carboxylic acid (**5ie**)

^{1}H NMR ($(CD_3)_2CO$)

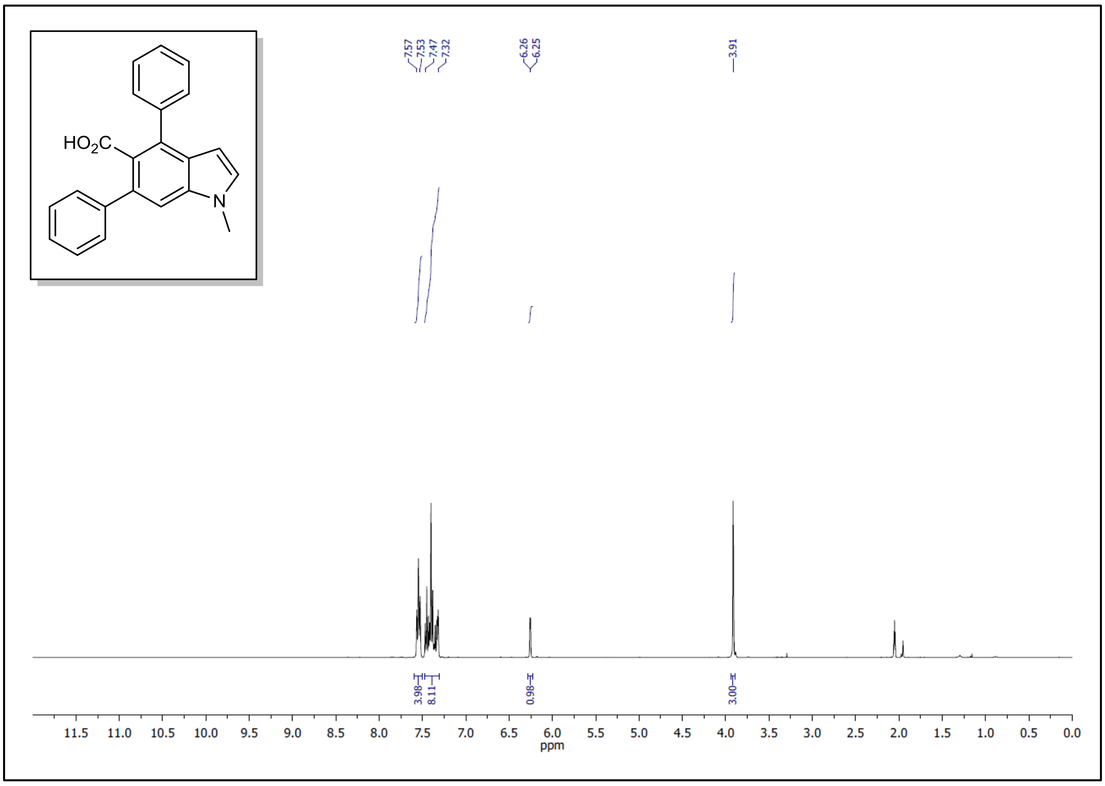

^{13}C NMR ($(CD_3)_2CO$)

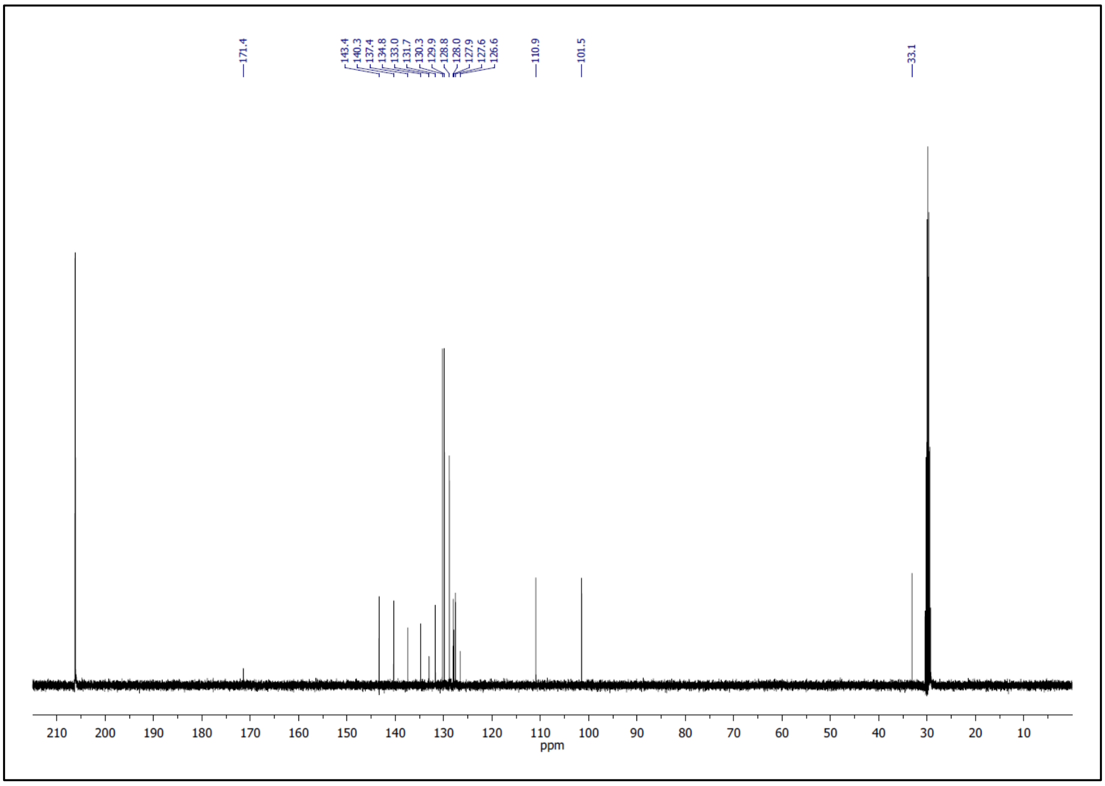

1-methyl-4,6-bis(4-(trifluoromethyl)phenyl)-1H-indole-5-carboxylic acid (**5io**)

^{1}H NMR ($(CD_3)_2CO$)

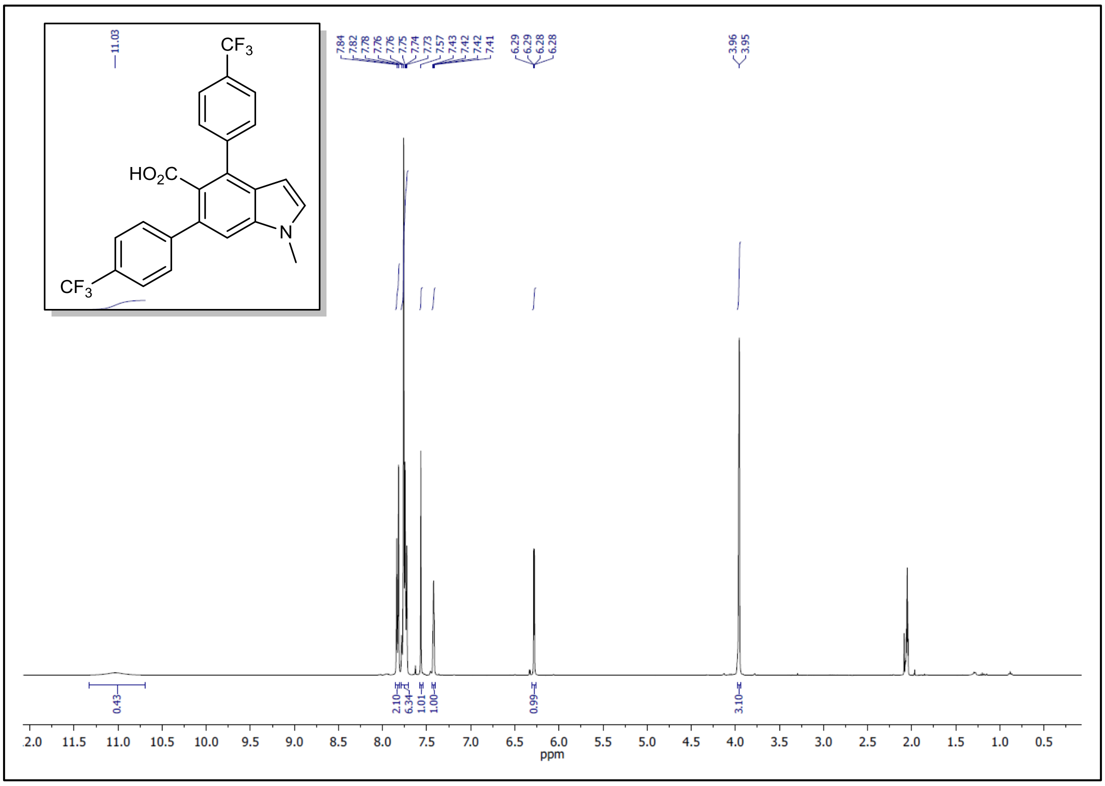

^{13}C NMR ($(CD_3)_2CO$)

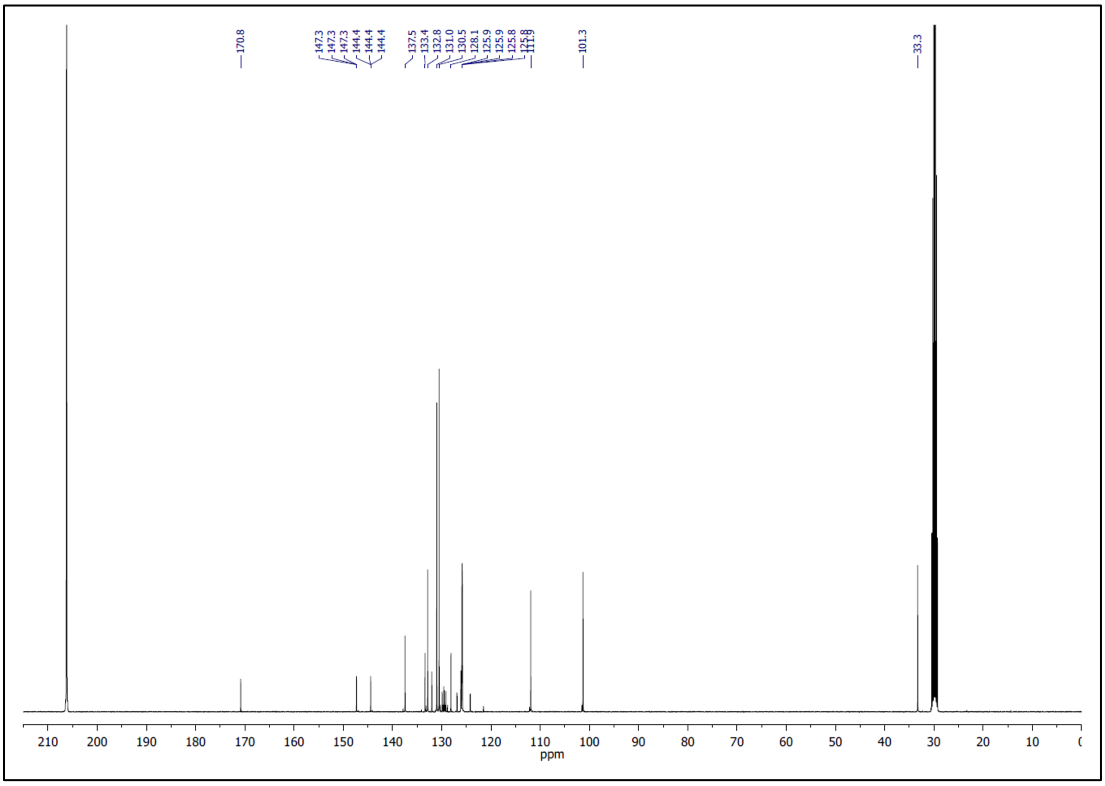

^{19}F NMR ($(CD_3)_2CO$)

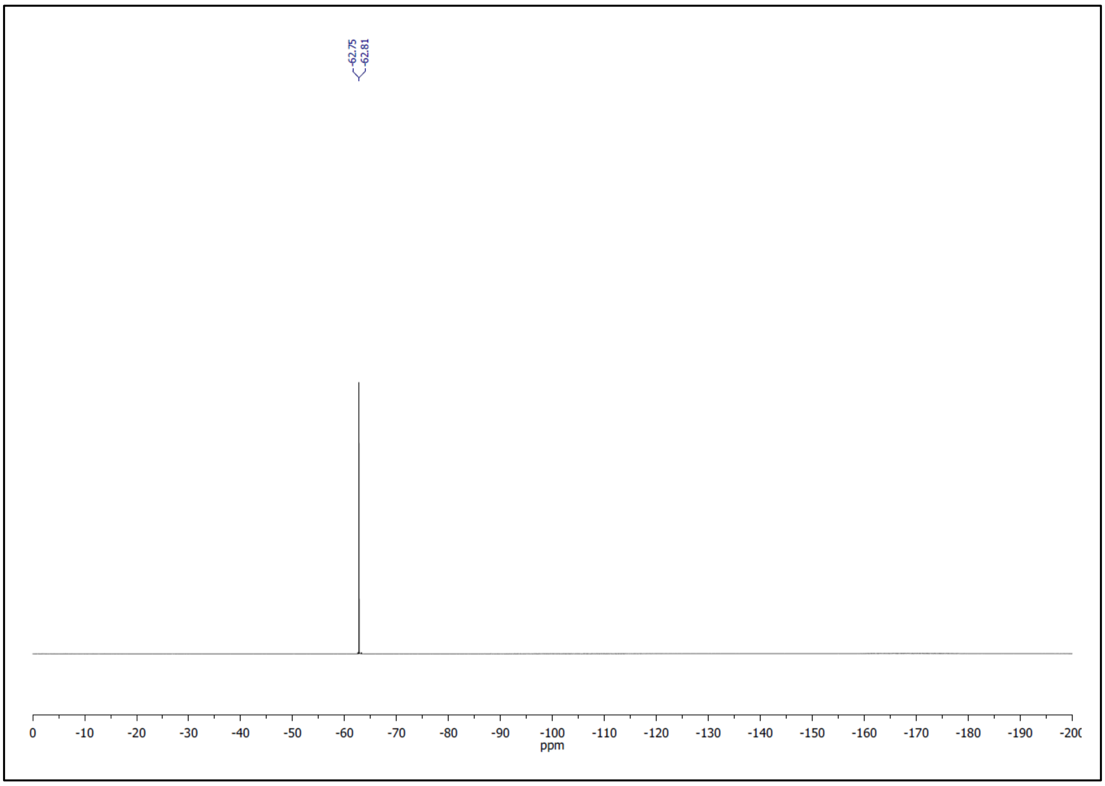

4,6-bis(4-chlorophenyl)-1-methyl-1H-indole-5-carboxylic acid (**5ij**)

^{1}H NMR ($(CD_3)_2CO$)

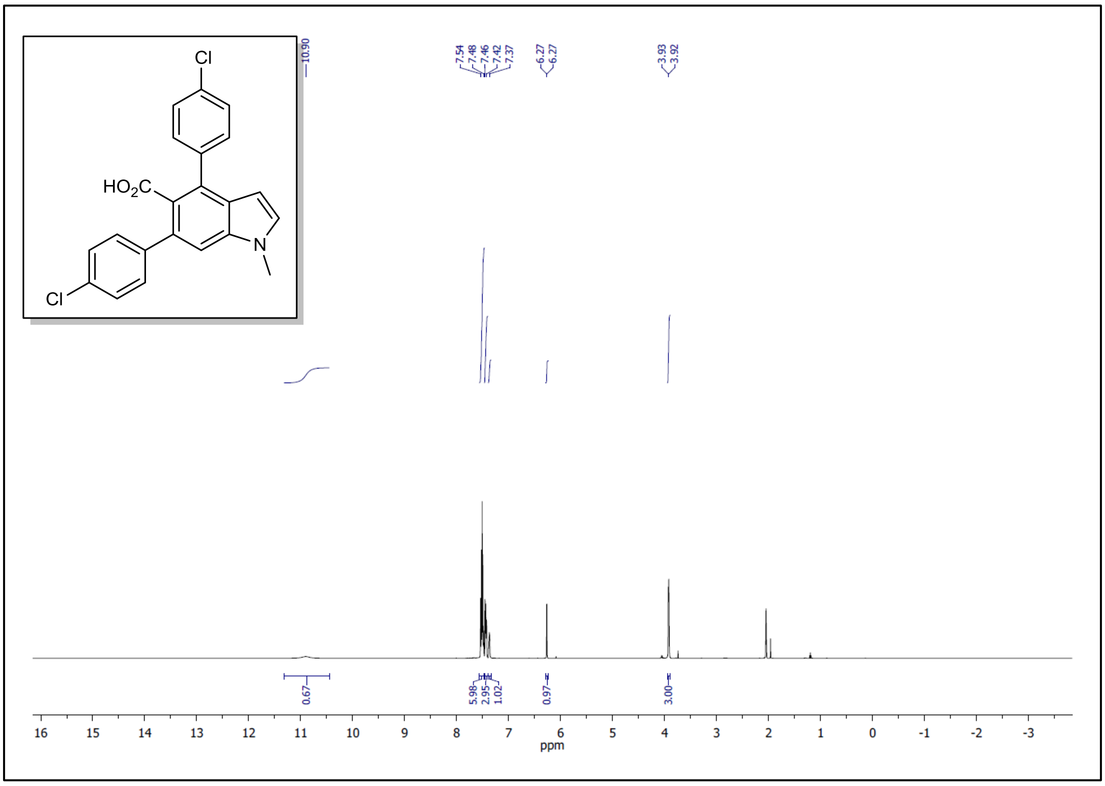

^{13}C NMR ($(CD_3)_2CO$)

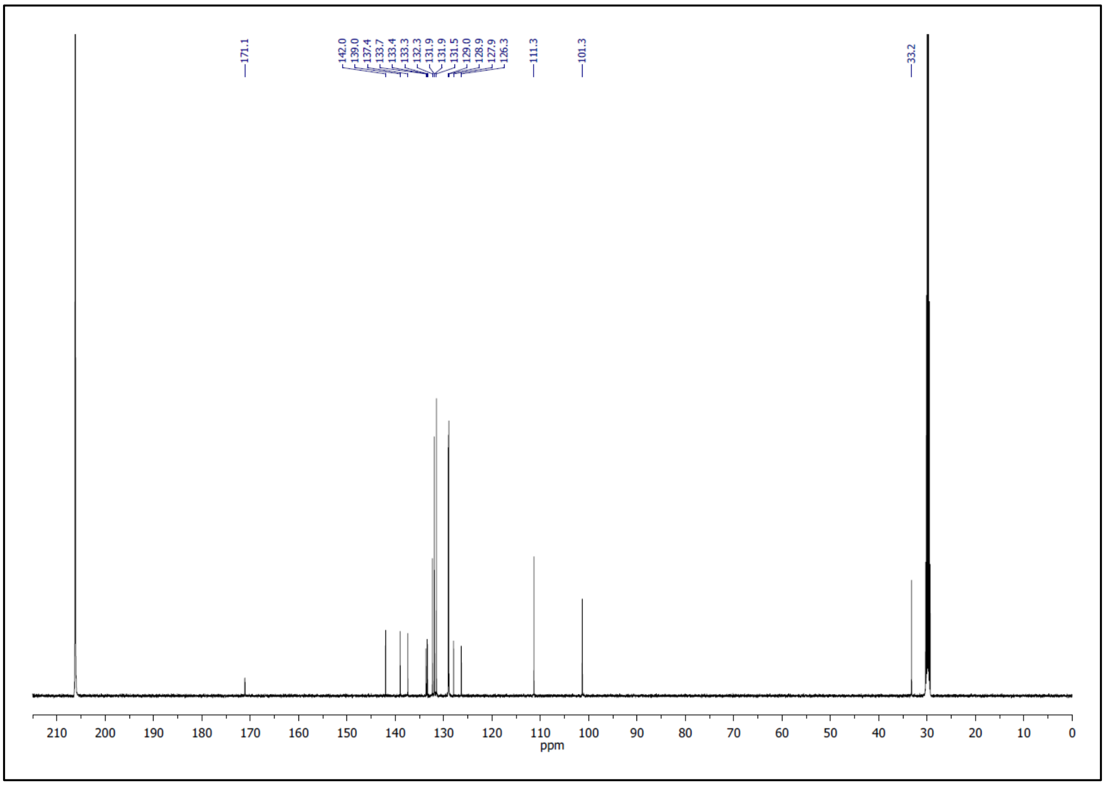

4,6-bis(4-bromophenyl)-1-methyl-1H-indole-5-carboxylic acid (**5ik**)

^{1}H NMR ($(CD_3)_2CO$)

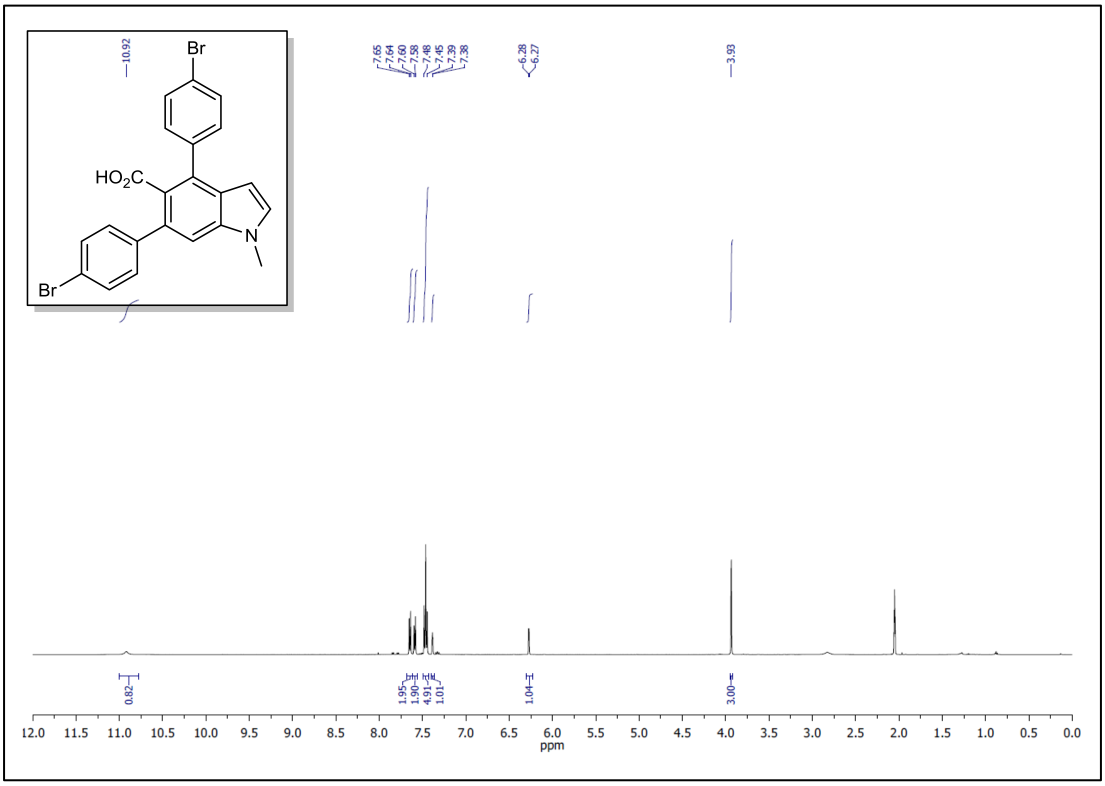

^{13}C NMR ($(CD_3)_2CO$)

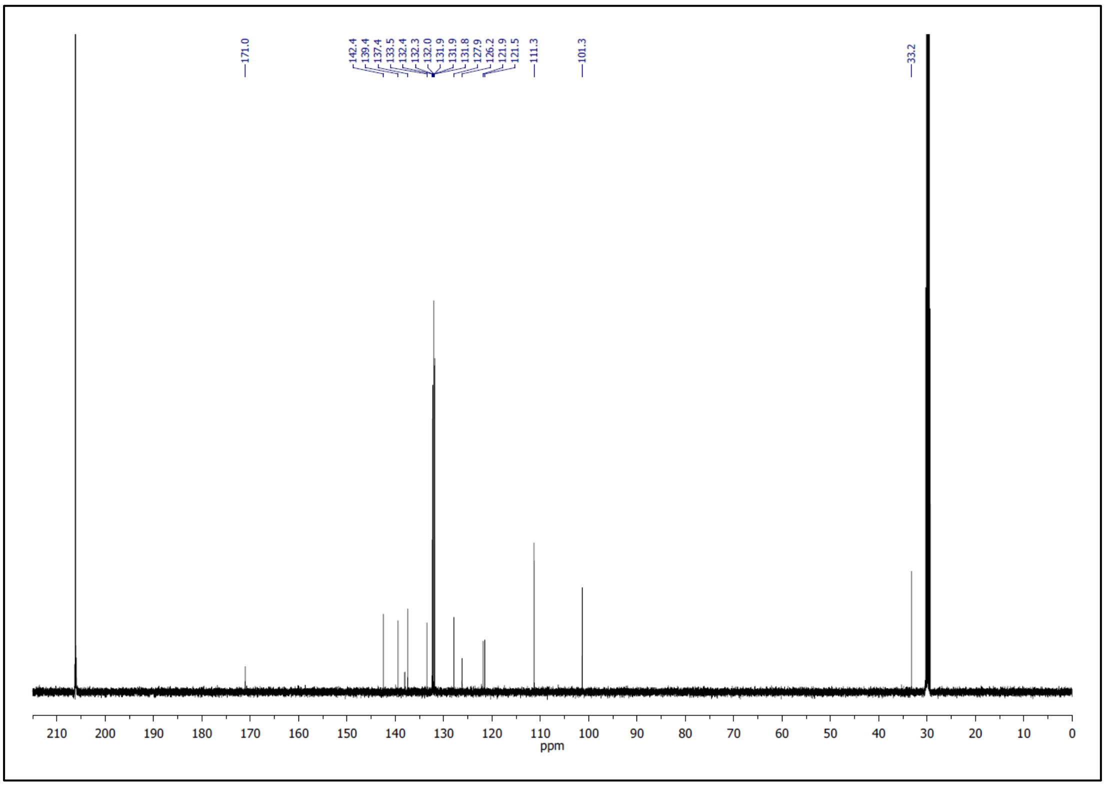

3-(2-(dimethylamino)-2-oxoacetyl)-1-methyl-1H-indole-6-carboxylate (**4d-ester**)

^{1}H NMR ($(CD_3)_2CO$)

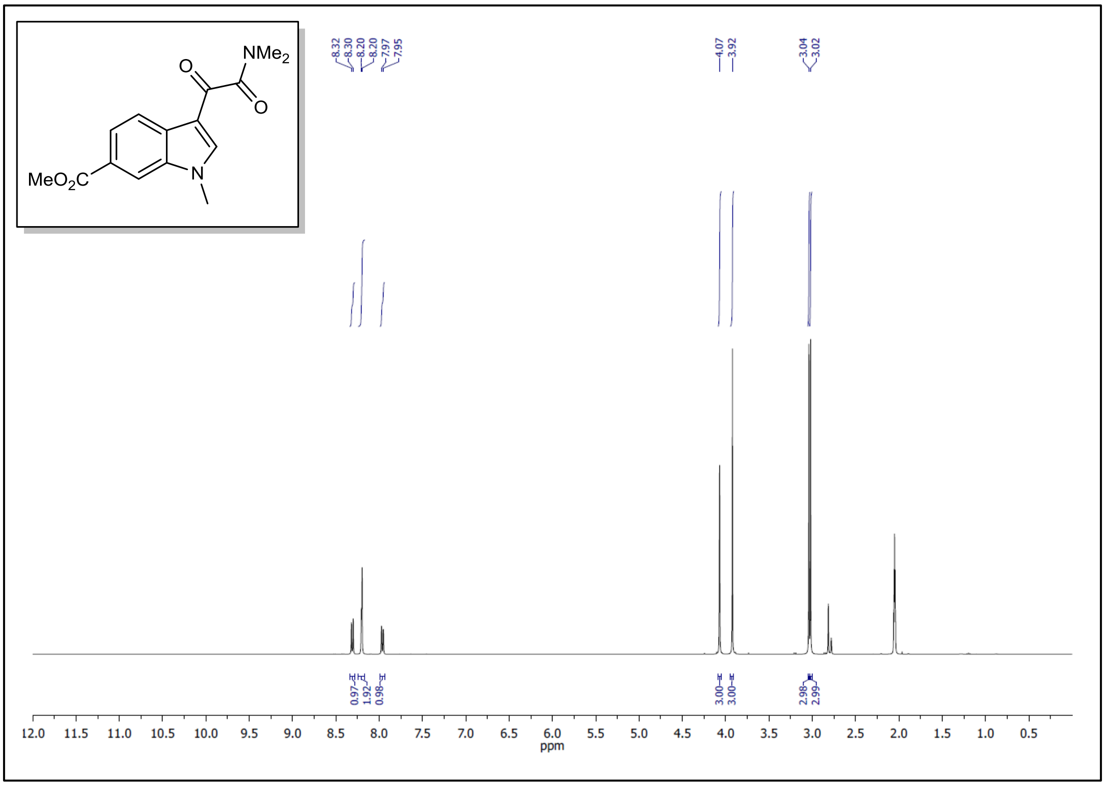

^{13}C NMR ($(CD_3)_2CO$)

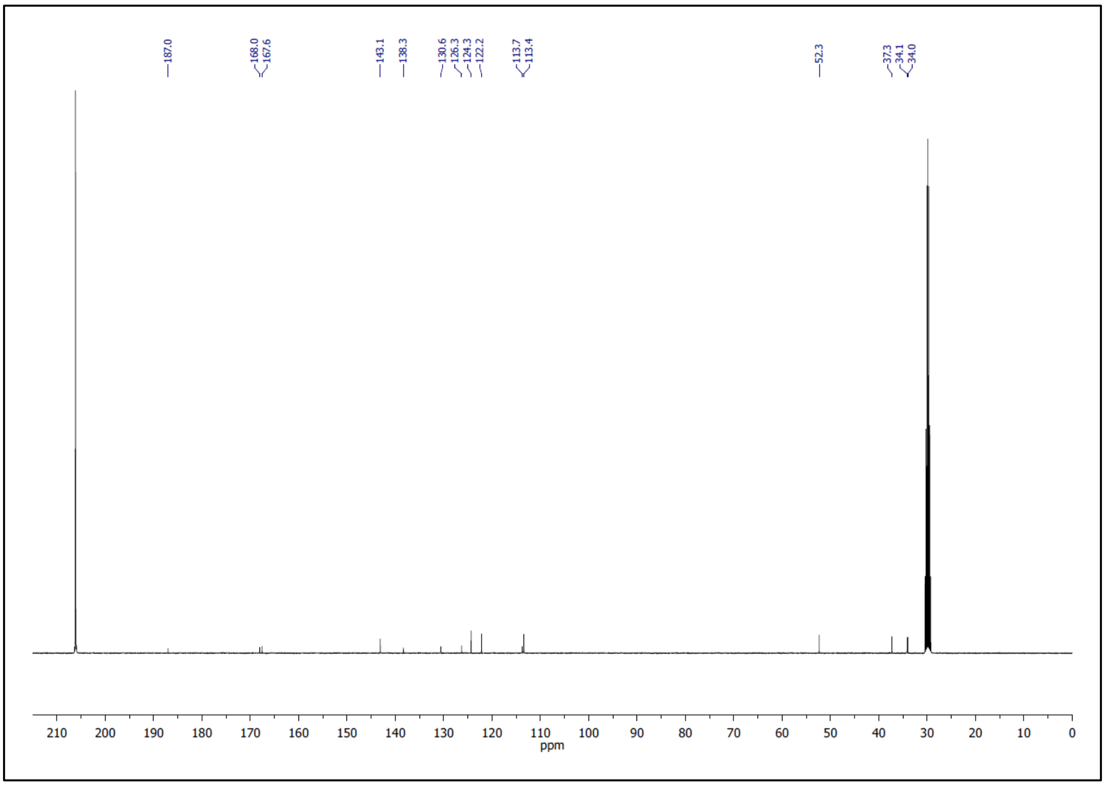

3-(2-(dimethylamino)-2-oxoacetyl)-1-methyl-1H-indole-6-carboxylic acid (**4d**)

^{1}H NMR ($(CD_3)_2CO$)

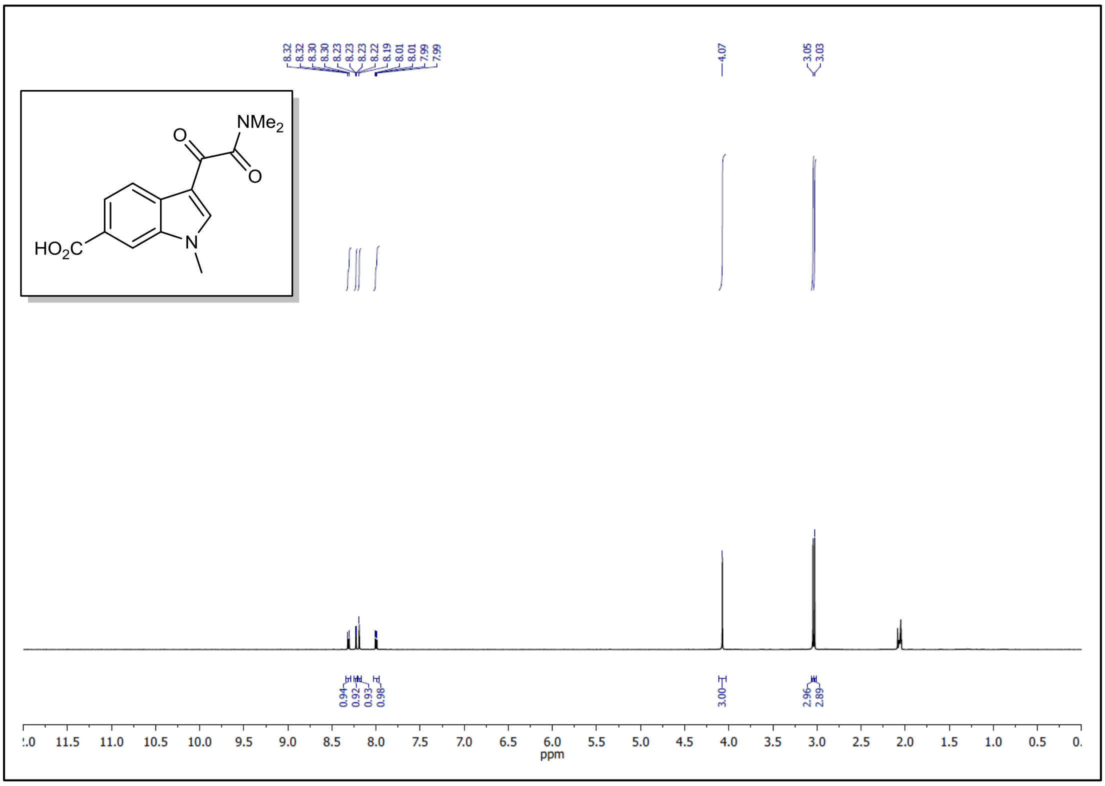

^{13}C NMR ($(CD_3)_2CO$)

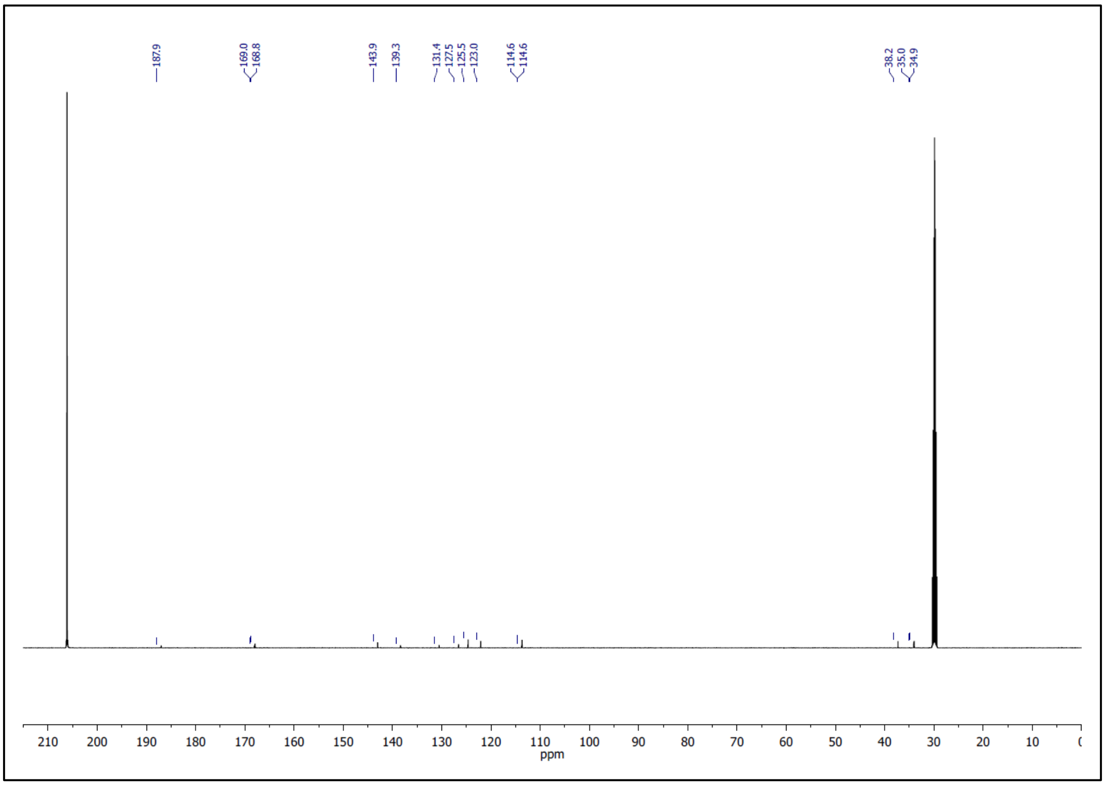

potassium 1,1,1,3,3,3-hexafluoro-2-(trifluoromethyl)propan-2-olate

^{13}C NMR ($(CD_3)_2CO$)

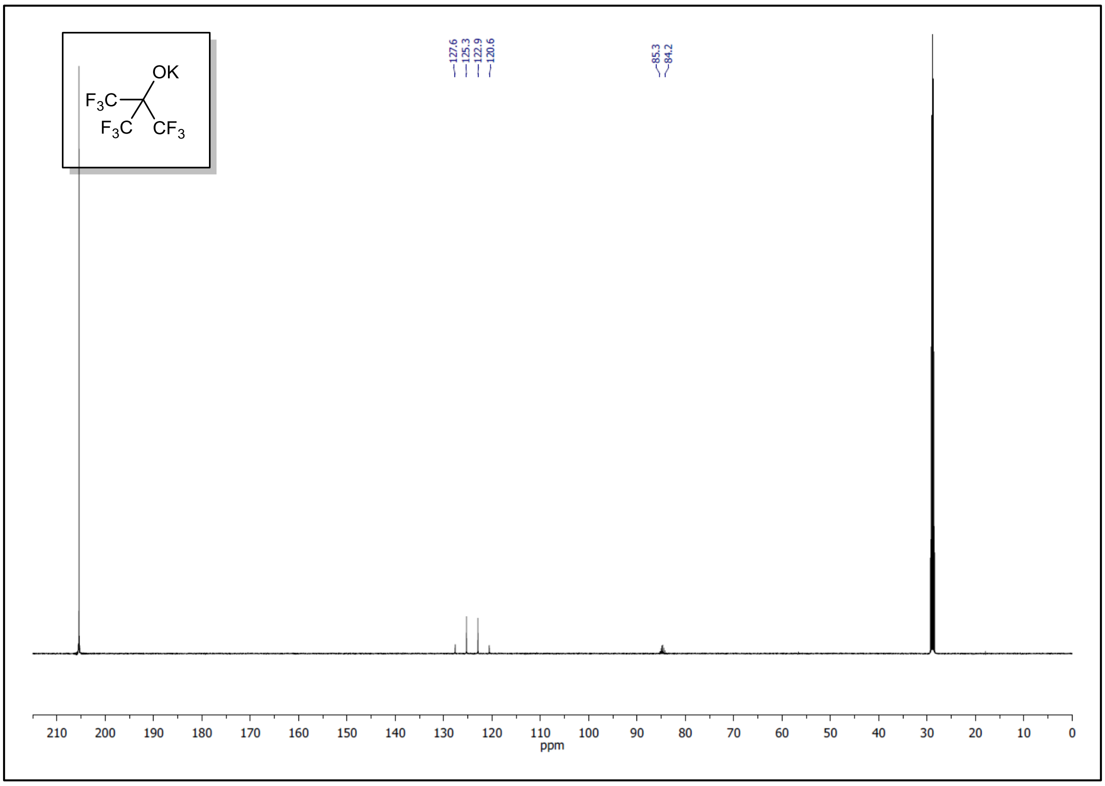

^{19}F NMR ($(CD_3)_2CO$)

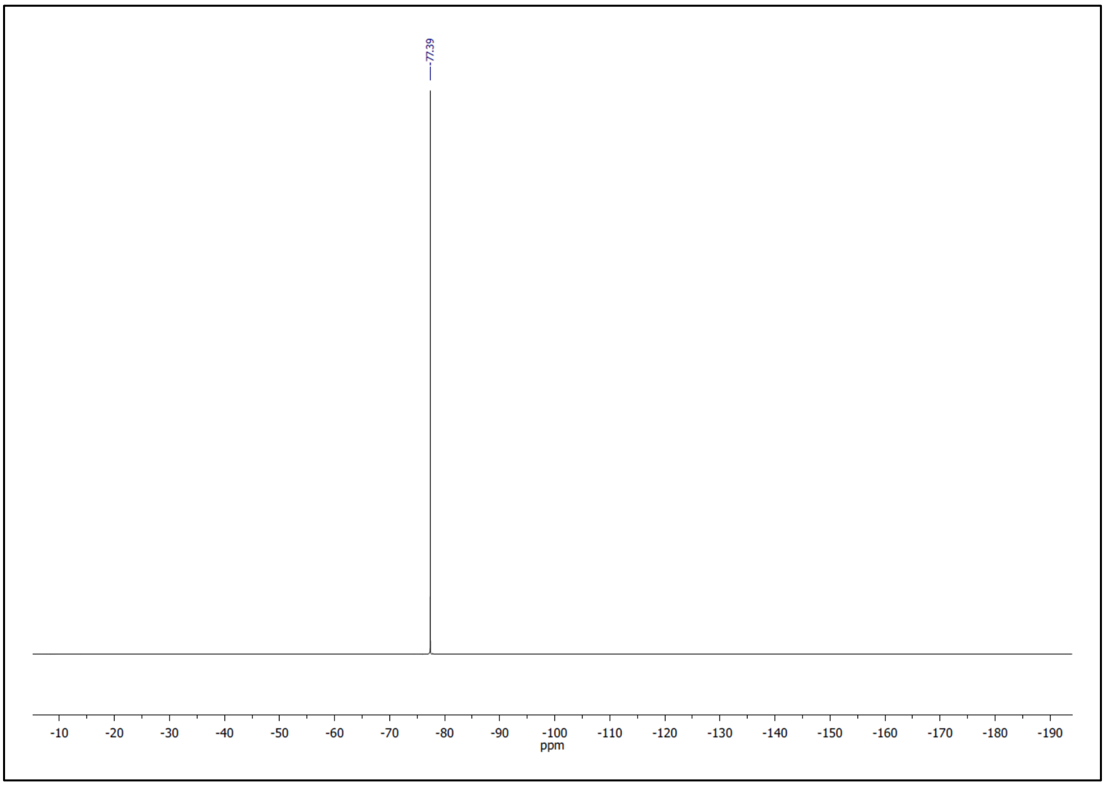

References

[1] M. Simonetti, G. J. P. Perry, X. C. Cambeiro, F. Juliá-Hernández, J. N. Arokianathar, I. Larrosa, *J. Am. Chem. Soc.* **2016**, *138*, 3596-3606.

[2] H. Shi, D. J. Babinski, T. Ritter, *J. Am. Chem. Soc.* **2015**, *137*, 3775-3778.

[3] M. Winn, E. B. Reilly, G. Liu, J. R. Huth, H.-S. Jae, J. Freeman, Z. Pei, Z. Xin, J. Lynch, J. Kester, T. W. von Geldern, S. Leitza, P. DeVries, R. Dickinson, D. Mussatto, G. F. Okasinski, *J. Med. Chem.* **2001**, *44*, 4393-4403.

[4] U. Laban, D. Kurrasch-Orbaugh, D. Marona-Lewicka, D. E. Nichols, *Bioorg. Med. Chem. Lett.* **2001**, 11, 793-795.

[5] C. Zhu, Y. Zhang, J. Kan., H. Zhao, W. Su, *Org. Lett*. **2015**, *17*, 3418-3421.

[6] L. Huang, D. Hackenberger, L. J. Gooßen, *Angew. Chem. Int. Ed.* **2015**, *54*, 12607-12611.

[7] C. A. Fleckenstein, H. Plenio, *Chem. Eur. J.* **2007**, *13*, 2701-2716.

[8] X. Wang, D. Leow, J-Q. Yu, *J. Am. Chem. Soc.* **2011**, *133*, 13864-13867.

[9] H. A. Chiong, Q.-N. Pham, O. Daugulis, *J. Am. Chem. Soc.* **2007**, *129*, 9879-9884.

[10] L. Huang, D. Hackenberger, L. J. Gooßen, *Angew. Chem. Int. Ed.* **2015**, *54*, 12607-12611.
